# *In vivo* Monitoring of Transcriptional Dynamics After Lower-Limb Muscle Injury Enables Quantitative Classification of Healing

**DOI:** 10.1038/srep13885

**Published:** 2015-09-18

**Authors:** Carlos A. Aguilar, Anna Shcherbina, Darrell O. Ricke, Ramona Pop, Christopher T. Carrigan, Casey A. Gifford, Maria L. Urso, Melissa A. Kottke, Alexander Meissner

**Affiliations:** 1 Massachusetts Institute of Technology - Lincoln Laboratory, Lexington, MA 02127, USA; 2 Broad Institute of MIT and Harvard, Cambridge, MA 02142, Harvard Stem Cell Institute, Cambridge, MA 02138, Dept. of Stem Cell and Regenerative Biology, Harvard University, Cambridge, MA 02138, USA; 3 United States Army Institute of Environmental Medicine - Military Performance Division, Natick, MA 01760, USA.

## Abstract

Traumatic lower-limb musculoskeletal injuries are pervasive amongst athletes and the military and typically an individual returns to activity prior to fully healing, increasing a predisposition for additional injuries and chronic pain. Monitoring healing progression after a musculoskeletal injury typically involves different types of imaging but these approaches suffer from several disadvantages. Isolating and profiling transcripts from the injured site would abrogate these shortcomings and provide enumerative insights into the regenerative potential of an individual’s muscle after injury. In this study, a traumatic injury was administered to a mouse model and healing progression was examined from 3 hours to 1 month using high-throughput RNA-Sequencing (RNA-Seq). Comprehensive dissection of the genome-wide datasets revealed the injured site to be a dynamic, heterogeneous environment composed of multiple cell types and thousands of genes undergoing significant expression changes in highly regulated networks. Four independent approaches were used to determine the set of genes, isoforms, and genetic pathways most characteristic of different time points post-injury and two novel approaches were developed to classify injured tissues at different time points. These results highlight the possibility to quantitatively track healing progression *in situ* via transcript profiling using high- throughput sequencing.

Lower-limb musculoskeletal injuries (LLMIs) are common amongst athletes and military personnel^1^, with hundreds of thousands reported every year from the military alone^2^. As athletes and soldiers are highly motivated to resume physical activities, the risk of re-injury before fully healing is high. Following a traumatic LLMI, tightly controlled intra- and intercellular transcriptional systems are activated and coordinated to ensure intermediate physiological behavior^3^,^4^ while also generating appropriate repair and regeneration. The degree and duration of these various processes^5^,^6^,^7^ operate in a manner that is proportional to the severity of the injury, are coordinated across different cell types^8^,^9^ and generally involve a large number of molecules and interrelated pathways^10^.

Methods to unambiguously determine injury state and healing progression can provide effective treatment decisions and rehabilitative strategies, as well as prevent premature return-to-activity lowering the risk of reinjury. Current approaches for gauging injury severity and healing progress have primarily focused on three-dimensional imaging^11^ (computed topography, magnetic resonance imaging) but these approaches are typically expensive to perform as well as interpret and suffer from poor sensitivity and contrast resolution. Recently, ultrasound imaging has become popular due to its cost and portability^12^, but the approach is still limited by the field of view, and operator’s knowledge of anatomy. Thus, there remains an unmet need to monitor muscle injury severity and healing progression after injury^13^,^14^,^15^.

RNAs extracted from the injured muscle would serve as excellent candidates for monitoring injury severity and permit quantitative insights^16^,^17^ into the different muscle repair and regeneration pathways that are temporally activated after injury. Recently, high-throughput RNA sequencing (RNA-seq) has enabled unbiased, global views of gene expression patterns with high accuracy and reproducibility from small or degraded sample inputs, opening the possibility to quantitatively track global *in-vivo* transcriptional patterns from small tissue samples. Herein, a traumatic injury was administered to the tibialis anterior of a young, healthy mouse model and the tissue was extracted at different times ranging 3 hours to 672 hours (1 month). A portion of the tissue was then processed (<5 mg), mRNA extracted and polyadenylated RNA fractions were prepared into strand-specific sequencing libraries. The libraries were then sequenced, analyzed and the adaptive transcriptional responses and their associated pathways were integrated to construct a comprehensive view of the injury and healing progression through time.

With the goal of developing an unbiased method for tracking healing progression, the generated datasets were utilized as training data and twelve additional RNA-Seq datasets were generated where the time points were blinded to act as test data. Two traditional bioinformatics approaches (principal component analysis (PCA), support vector machine (SVM) multiclass classification) and two novel gene signature methods were used to determine the set of genes and isoforms most characteristic of healing state post-injury. The PCA, SVM, and time point signatures methods were then re-applied to the datasets at the pathway level to identify pathways that were differentially activated over time. Ultimately, the time point signatures method enabled accurate classification of 10 out the 12 test samples, and led to the identification of 370 pathways with activation levels that varied significantly (p < 0.05) at various time points after injury. As traumatic LLMIs present variable healing trajectories for different individuals, using an unbiased transcript-based tracking approach at both the gene and pathway level can enable quantitative classification of injury severity as well as inform therapeutic efficacy.

## Results

### Histological Analysis & Global Transcriptional Dynamics After Traumatic LLMI

**Figure 1:**
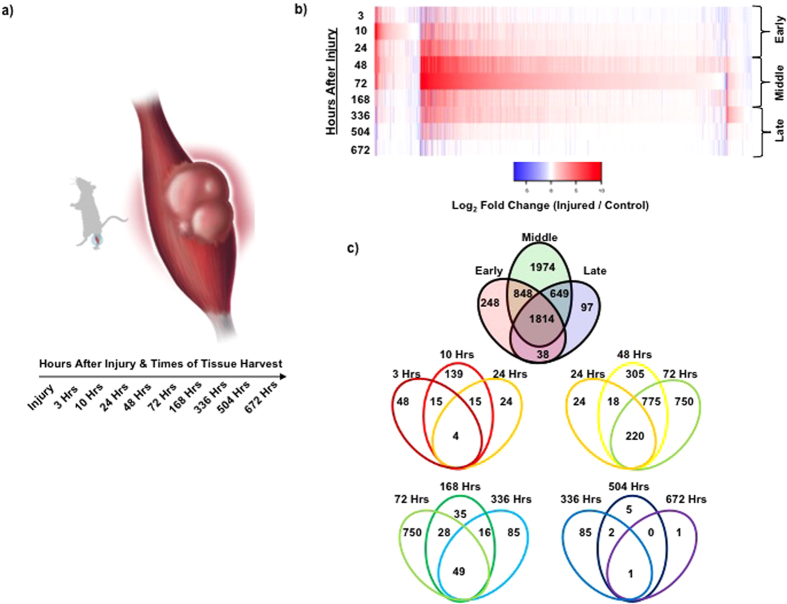
Global transcriptional dynamics after traumatic muscle injury. (**a**) Schematic depicting injury to tibialis anterior (TA) muscle (highlighted in red) and bottom inset shows times after injury when muscles were harvested. (**b**) Heatmap of genes with FPKM >1 at one or more time points categorized into three time periods (early, middle and late). Genes were clustered by their fold change expression profiles in each period. (**c**) The Venn diagram illustrates the number of significant genes at each time period. For example, there were 139 genes with a significant fold change only at 10 h, 23 genes with a significant fold change only at 24 h, 15 genes with a significant fold change at 10 h, 24 h, and nowhere else, and 4 genes with a significant fold change at 3 h, 10 h, 24 h and nowhere else.

Administration of a freeze injury to the tibialis anterior (TA) muscle of ten-week old C57BL/6 mice^18^, provides a well-studied model of tissue repair after an acute LLMI. Briefly, a 6 mm steel probe, cooled to −70 °C, was applied to the exposed TA muscle and the leg was sutured. The injured muscle was then harvested at several time points after the injury (3, 10, 24, 48, 72, 168, 336, 504, 672 h) and the uninjured contralateral TA muscle was also extracted to serve as a control for each time point (Fig. 1a). Both injured and uninjured tissue samples at each time point were used to perform histological analysis (Supp. Fig. 1). Quantitative imaging of the stained (Evans blue dye) injured and uninjured tissues revealed a gradual increase in tissue damage until 48 h (Supp. Fig. 1b–c), indicating secondary damage to the muscle tissue occurred after the initial cryo-injury, which is consistent with previous studies^7^,^18^.

To assess the molecular mechanisms driving healing after the injury, high-resolution RNA-Seq was performed. First, total RNA was isolated from each tissue for each time point and enriched for mRNA. After quality assessment (Agilent 2100 BioAnalyzer), cDNA was then produced and sequencing libraries were prepared (Truseq, Illumina). Each library was then deep sequenced, mapped to the mouse reference genome (mm9) using TopHat and assembled with Cufflinks^19^ (see Methods). A total of 68 training data sets (31 injured, 37 controls) were produced with more than 5.9 billion aligned reads covering more than 448 billion bases. Twelve test datasets were also generated (6 uninjured controls, two injured 3 h datasets, two injured 10 h datasets and two injured 168 h datasets) with 755 million aligned reads covering 57 billion bases. Datasets were grouped into three time periods (early: 3–24 h, middle: 48–168 h, late: 336–672 h). Supplemental Fig. 2 shows two examples of sequence libraries for injured tissue samples obtained from two different mice and extracted at 24 h post-injury, where a high correlation was observed between these two samples (R^2^ > 0.95). The derived mRNAs were also validated using quantitative PCR (qPCR) for multiple genes (n = 25) through three time points and biological replicates from different samples and showed excellent agreement with the RNA-Seq data (Supp. Fig. 2).

To obtain comprehensive, unbiased views of the different gene expression programs and pathways associated with the injury, genes with expression changes as identified by CuffDiff 2 were examined^19^. In total, 12,544 mRNAs were detected with FPKM greater than 1 for at least one time point; and 11,900 genes were expressed (FPKM >1) at one or more time points (Fig. 1b). Of these, 5,668 genes exhibited dynamic behavior (Supp. Fig. 3), with the middle time period possessing the highest number of genes undergoing dynamic changes (5,285), compared with 2,910 genes for early, and 2,560 genes for late (Fig. 1c). 1,814 genes exhibited dynamic behavior at all three time intervals, while 248 genes exhibited dynamic behavior only in the early phase, 1,974 genes only in the middle phase, and 97 genes only in the late phase. The differentially expressed genes were grouped into functional categories using GSEA reactome datasets and further characterized via GO and KEGG annotation through the DAVID Bioinformatics database and the GO Toolkit. GO terms and KEGG pathways with high ranks were selected within the set of terms and pathways identified (Supplementary Tables 1 and 2). Categories that appeared with the lowest FDR and across multiple highly ranked clusters were analyzed further. A total of 597 genes were detected across all samples that underwent alternative splicing (FDR < 5%); with the middle time period exhibiting the highest number of splicing events with 391, and 141 genes exhibiting alternative promoter usage^19^.

### Pro-Inflammatory and Immune System Transcriptional Programs Control the Early Response After a Traumatic LLMI and are Tightly Regulated

Comparison of the injured samples against the controls for the early time points (3 h, 10 h, 24 h) showed a variety of gene expression profiles (Fig. 1b) with several sub-groups of genes changing levels immediately after the injury and rapidly subsiding, while others sustained activation over multiple weeks. The observed early waves of expression were partitioned into several sub-groups and genes within functional categories with statistical significance (p-value > 1e-6) were studied further.

**Figure 2:**
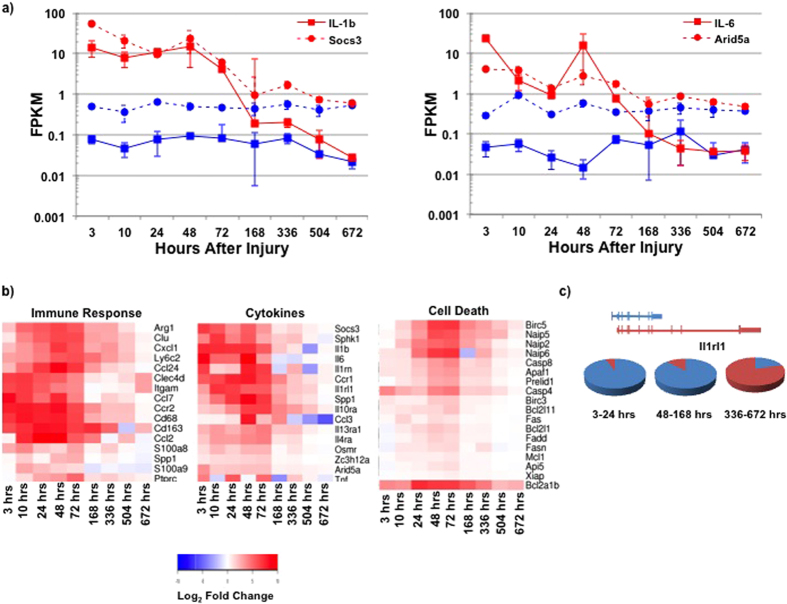
Inflammatory and immune response transcriptional programs activated after traumatic muscle injury. (**a**) Gene expression profiles of pro- and anti-inflammatory genes (IL-1b & Socs3 and Il-6 & Arid5a), which show similar activation profiles and are part of networks with opposing function, (red – injured samples, blue – uninjured samples, IL-1b – squares & solid line, Socs3 – circles & dashed line, IL-6 – squares & solid line, Arid5a – circles & dashed line). Arid5a operates to reduce IL-6 stability, indicating the inflammatory response to injury is transcriptionally regulated on multiple levels. (**b**) Heatmaps of significantly up-regulated (red) or down-regulated (blue) genes for different functional categories. (**c**) Example of alternative splicing detected during early time period. Il1rl1 (ST2) undergoes an increase expression in the ST2L isoform (blue), which has previously been shown to promote proliferation and activation of anti-inflammatory macrophages.

Pro-inflammatory^20^,^21^ and chemotactic protein members^22^,^23^ such as IL-6, IL-1β and CCl2 were, as expected, rapidly upregulated in the early time period (Supp. Info S1 & Supp. Fig. 4). Anti- inflammatory genes^24^ such as Socs3, CD24 and IL-10rα and posttranscriptional regulators such as AT-rich interactive domain-containing protein 5a (Arid5a)^25^ and Regnase-1 (Zc3h12a)^26^ were also upregulated (Fig. 2a and Supp. Info S1). Arid5a and Regnase-1 have previously been shown to regulate inflammatory mRNA stability (such as IL-6), highlighting tight control of the inflammatory response and sensitivity of the RNA-Seq data. Transcripts encompassing a family of pro-apoptotic loci and anti-apoptotic loci were also detected in the early time period (Fig. 2b), which are likely the result of invading immune cells release of oxygen free radicals and other reactive oxygen species that induce secondary tissue damage and cell death. This observation of secondary tissue damage also agrees well with the histological analysis (Supp. Fig. 1). Alternative promoter usage and isoform switching events were also identified within the early period (Supp. Fig. 5). A novel example is the receptor for the “alarmin” gene^27^ (IL-1rl1 or St2), which was previously shown to activate upon tissue damage and restrain inflammation^28^. Figure 2c shows an increase in expression of the St2l isoform in the injured samples (blue isoform), which peaked at 10 h and remained elevated until 336 h. The St2l isoform has also previously been shown to promote proliferation and activation of anti-inflammatory macrophages^29^ and regulatory T-cells^8^, both of which critically restrain inflammation and influence various muscle repair and regeneration pathways. Collectively, these observations are consistent with previous studies of muscle tissue injury^5^,^6^,^7^,^18^,^20^,^21^,^22^,^23^,^27^, whereby transcripts associated with inflammation, invading immune cells, cytokine signaling, apoptosis, anoikis, and proliferation were observed immediately after injury. Detection of these transcripts also serve as excellent indicators to determine injury severity by observing shifts in the balance of pro- and anti-inflammatory molecules (such as CD24 and CCl2), which influence the degree of secondary damage^10^,^20^,^21^.

### Traumatic LLMI Generates Sequential Initiation of Complement, Notch and Wnt Signaling

**Figure 3:**
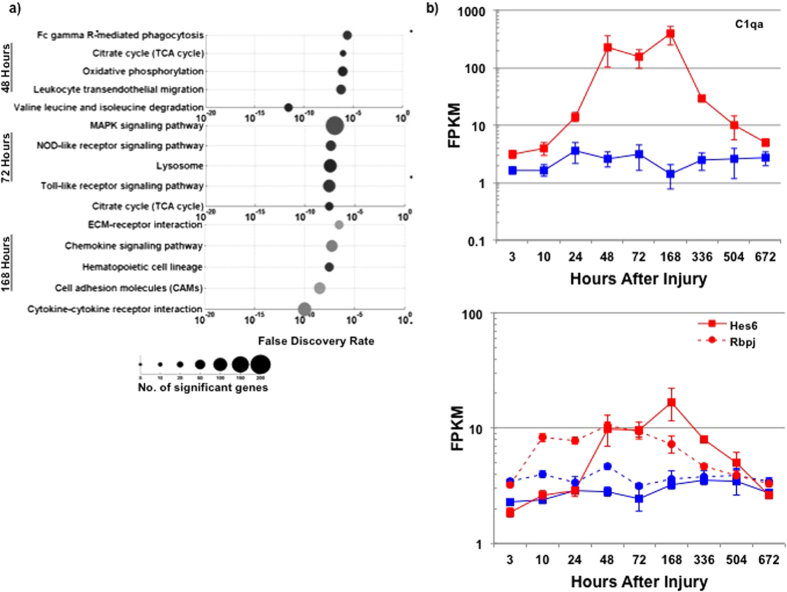
Dynamics of injured muscle tissue activated several days after injury. (**a**) Enriched KEGG pathways from differentially expressed genes for the middle time points (48–168 h). The size of the circle corresponds to the number of significant genes with each enriched pathway. Categories associated with growth emerge in contrast to the early period, which was characterized by inflammation and cell death. (**b**) Gene expression profiles of complement cascade trigger (C1qa) and two genes associated with different signaling pathways (Rbpj – Notch signaling, Hes6 – myoblast commitment and differentiation). The temporal activation of these different genes (and their associated networks and pathways) illustrates a progression of Complement and Notch activation, followed by Wnt signaling and myogenic differentiation. (red – injured samples, blue – uninjured samples, Rbpj – circles & dashed line, Hes6 – squares & solid line).

A significant fraction of the upregulated genes in the early and middle time periods can be ascribed to invading immune cells (Fig. 3a), which in part act to phagocytize debris from the injured site. Concordantly, dramatic increases in expression of phagocytic and complement cascade genes were detected (Supp. Fig. 6) and Fig. 3b illustrates the expression profile of the C1qa gene, a complement cascade trigger. C1qa has previously been shown to inhibit muscle regeneration and stimulate the Wnt signaling pathway^30^, as well as induce expression of fibrotic genes and collagen production^31^ (Supp. Info S2 & Supp. Fig. 7). As Wnt signaling is viewed to increase in the middle time period, signatures of proliferating progenitors (Notch signaling^32^,^33^, bone morphogenetic proteins^34^, secreted frizzled proteins^35^), which were upregulated in the early time period, begin to decline in expression. This temporal switch from Notch to Wnt^36^ is also accompanied by increases in expression of multiple genes associated with myogenic differentiation (Hes6 and Myod1, Myog, Myf6). Figure 3b demonstrates the expression profiles of RbpJ, the primary mediator of Notch signaling^37^, and HES6, a transcription factor that modulates myoblast commitment and differentiation^38^. As RbpJ and Notch signaling declines in the middle time period, Hes6 and Wnt signaling increase to promote myoblast differentiation. The temporal activation of these new sets of genes suggests their detection can assist to identify the onset of healing as well as establish the regenerative competence of a given individual after an acute traumatic LLMI.

### Traumatic LLMI Induces Migratory Fibroblasts to Adopt a Contractile Phenotype

**Figure 4:**
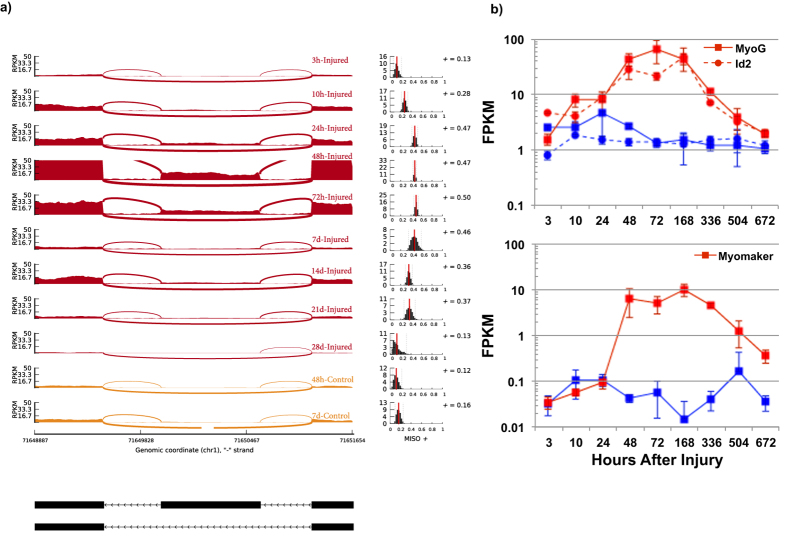
Muscle tissue microenvironment signaling after traumatic muscle injury. (**a**) The left side illustrates the RNA-Seq read coverage for the fibronectin (Fn1) gene for the EDA exon during different time points after the injury. The MISO + (percent spliced in) values are on the right and show a shift in the EDA exon for the middle time points, indicating an increased detection of the ED-A splice variant. Detection of the splice variant decreases back to control values at the 672 h time point. (**b**) Top—Gene expression profile of Myogenin (MyoG), a transcription factor that regulates terminal differentiation of the myogenic program, and Id2, a helix-loop-helix protein that inhibits myogenic factor activity and modulates the terminal myogenic differentiation program. Bottom—Gene expression profile of Myomaker (Tmem8c), a transmembrane protein that fuses adjacent myoblasts, which remained upregulated until 672 h after the injury.

Migrating fibroblasts play an essential role in tissue remodeling after muscle injury, through production of new extracellular matrix (ECM) components and development of a phenotype that contracts the surrounding matrix^31^,^39^. The increased contractional forces are permitted by altered interactions between integrins and cell binding domains that modulate cell adhesion. These modified interactions are orchestrated through splicing changes to produced fibronectin transcripts such as the ED-A and ED-B exons^40^. Figure 4a demonstrates detection of the ED-A splice variant of fibronectin^41^ beginning at 24 h after the injury and shifting back at approximately 672 h. Detection of the ED-A splice variant indicates formation of new ECM and altered niche stiffness^42^, which in addition to the soluble factors emitted by invading immune cells, has previously been shown to activate satellite cells^43^.

### Activation of Muscle Repair Machinery

The changes to the physical microenvironment and cytokines from resident and invading cells along with muscle regulatory factors (Myod1, Myog and Myf6), numerous transcription factors^44^,^45^ and IGF signaling^46^ direct the cells in the injured site toward the skeletal muscle program and regeneration of the tissue. Many of the new expression programs showed overlapping kinetics and opposing functions such as Myod1 and Myog with inhibitor of DNA binding genes (Id1, Id2, Id3, Fig. 4b). A unique feature of differentiating myogenic cells in response to injury is the ability to fuse to existing damaged fibers, which we view through a group of genes promoting fusion^47^ of cells to each other and existing muscle fibers^48^. Figure 4b illustrates the gene expression profile of Myomaker (Tmem8c), a transmembrane protein that enhances fusion of myoblasts, which increased in expression during the middle time period and remained upregulated until 672 h. In aggregate, the upregulation of satellite cell markers, transcription factors and myoblast fusion genes indicates the nascent stages of muscle remodeling. The initiation of these different gene networks can be utilized to monitor healing progression of the injured muscle as well as offer insights into the signaling cascades that control healing timelines.

### Systems-Level Perspective of Transcriptional Networks

**Figure 5:**
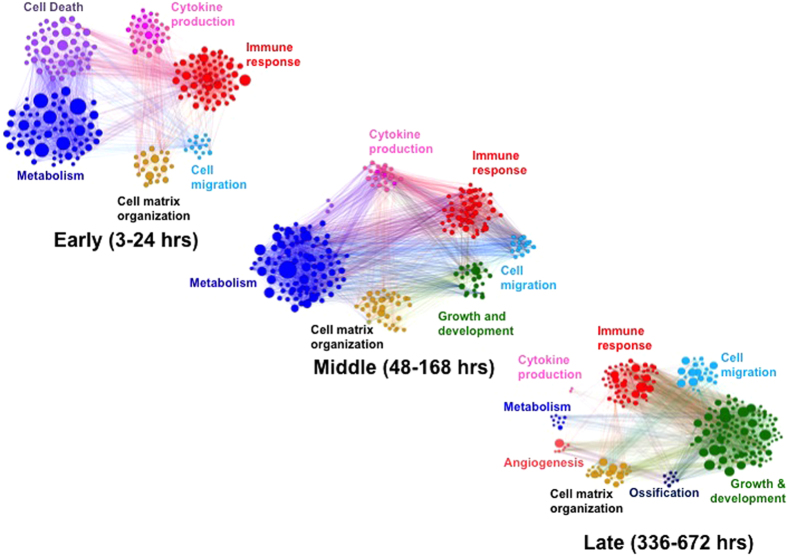
Temporal evolution of transcriptional coregulated networks organized by function after traumatic injury. Each network diagram is composed of statistically significant functional enrichments, where Gene Ontology (GO) terms are clustered by functional category such that all terms with a common ancestor term are the same color. The size of each circle corresponds to the corrected P-value of the associated GO term, and edges in the graph represent interactions between associated GO terms.

Summation of the different transcriptional networks for all of the time points shows the injury site is a complex environment with multiple cell types executing a wide variety of functions. Figure 5 illustrates the temporal transcriptome dynamics organized into three time periods, whereby co-regulated networks are categorized by Gene Ontology (GO) terms. The resulting network diagram captures the evolution of different transcriptional groups such as the immune network and cell-death program in the early time period, cytokines and growth and development in the middle and late periods, both of which were described above. The diagram also highlights combinatorial regulation of the injured site and healing progression. For example, in the middle time period, cytokines, immune cell genes and elements of the ECM are observed to interact with genes involved with growth and development. As illustrated above, these collective interactions drove satellite cell proliferation through the Complement and Notch signaling pathways followed by differentiation and active Wnt signaling, all of which have previously been shown to influence satellite cell activation and differentiation^5^,^8^,^22^,^30^,^31^,^32^,^36^,^43^,^44^. Consequently, the collective interaction of many transcriptional programs such as inflammation, cytokine signaling, immunity, ECM remodeling, metabolism, and myogenic differentiation converge to influence the dynamics of satellite cells and muscle repair and regeneration (Supp. Fig. 7). The observed transcriptional patterns suggest the possibility that their detection can be utilized as bioinformatics classifiers^16^,^17^ to track healing progression after injury.

### Development of Transcriptional Signature Classification Schemas

As the observed gene expression dynamics displayed excellent agreement with previous muscle injury studies and showed unique temporal kinetics, an unbiased bioinformatics strategy for tracking healing progression using gene expression data after an LLMI was developed. The previously generated 68 datasets were utilized as training data and twelve additional RNA-Seq datasets were generated where the time points were blinded to act as test data. The twelve test datasets corresponded to three different time points and represented a mix of injured and uninjured samples (six uninjured control samples, two injured 3 h samples, two injured 10 h samples, and two injured 168 h samples). Four methods were developed for evaluation of the test datasets: Support Vector Machines (SVM), Principal Component Analysis (PCA), and two time point signatures methods. A neural network approach was considered, but insufficient training datasets were available for proper training^49^.

### Support Vector Machine Classifier Performance

**Figure 6:**
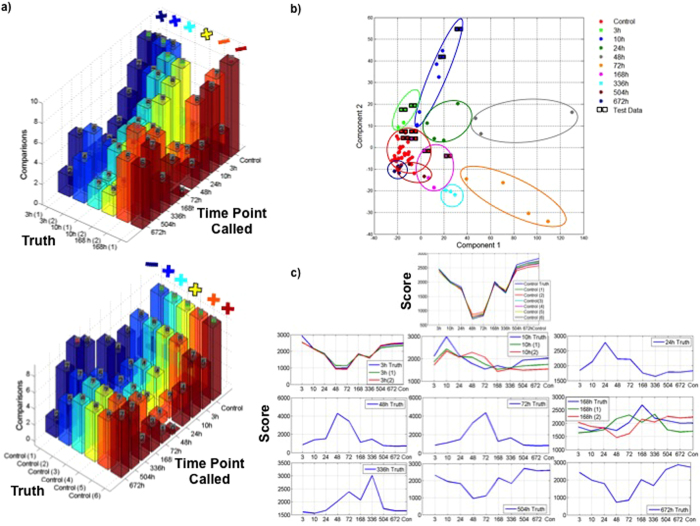
Results from various bioinformatics classification schemes utilized to analyze transcriptomic datasets show accurate categorization of injured and uninjured samples. (**a**) One-versus-one support vector classification results for test samples. A test sample was analyzed with 45 classifiers, each of which assigned the sample to one of two time points. Voting was used to group classifier results. The height of the bars indicates the number of votes given to each time point for a given sample. Top graph displays classification results for injured samples – 2 samples from 3h after injury, 2 samples for 10 h, 2 samples for 168 h. Bottom graph displays classification of results for 6 control samples. (**b**) Principal component analysis clustering of 12 test samples at the gene level. 66 training samples and 12 test samples are plotted in the space of principal components 1 and 2. Labels specify the time point of the nearest training sample for each of the test samples. Misclassified samples are circled in red. All other sample classifications were correct. (**c**) Similarity profiles of training and test samples to the control data and each of the 9 injured time points. Truth sample profiles are indicated in blue. If a scored sample and a truth sample for a given time point both exhibit a fold change for a gene, or if both exhibit no fold change for the gene, the score is incremented. The score increment is equal to the normalized fold change (on a scale from 0 to 1) in the truth sample relative to a control, or 0.5 if both sample exhibit no fold change.

An SVM classifier was developed and the best performance was obtained when the data was filtered to include all significant genes (see Methods). The weighted SVM calls from each pair of classifiers were summed, and the time point with the highest number of weighted votes was designated as the final classification call. The performance of the SVM classifier is illustrated in Fig. 6a, whereby the positive or negative symbol over each graph represents if the SVM call was accurate (positive symbol) or inaccurate (negative symbol). The relative height of the bars can be analyzed further to uncover generalizable patterns of performance. As can be seen for the uninjured control datasets, the height of the 10 h bars is slightly lower than the height of the control bars for four datasets. Similarly, for the injured 3 h datasets, the height of the uninjured control bars is slightly lower than the height of the injured 3 h bars. This result demonstrates that the SVM call possessed high confidence since adjacent time points both have a high number of votes. Overall, these results demonstrate that the SVM classifier could accurately identify 75% of the test datasets, but other bioinformatics techniques were evaluated to determine if higher classification accuracy could be obtained.

### Principal Component Analysis Results

Principal component analysis (PCA) was performed on both the training and test datasets. Figure 6b depicts the training and test datasets plotted in the space of principal components 1 and 2, whereby these two components account for nearly 60% of the variance observed between the datasets. The circles are results from the training datasets and squares indicate the test datasets, where the left square indicates the time point the dataset was created from and the right square indicates the time point the dataset was classified as. The samples that were misclassified by PCA are uniquely similar to the errors observed from the SVM classifier. Figure 6b shows a gradual shift of expression signatures away from the uninjured controls from 3 h until 72 h. Beginning at 168 h after injury, the samples cluster progressively closer to the uninjured controls, such that the 672 h injured samples are nearly indistinguishable from the uninjured control datasets. The scatterplot also indicates that the three samples most likely to be misclassified – the two injured 168 h samples and one of the six uninjured control samples, do not cluster with other samples from the same time point.

The PCA results suggest several additional rules for evaluating the confidence of sample classification decisions. For both the PCA and SVM approaches, samples at 3 h and 504 h cluster near the controls. Similarly, adjacent time points are near to each other in the multi-dimensional feature space (in the case of PCA), and kernel space (in the case of SVM).

### Two Time Point Signatures Methods

In the time point-weighted signatures method, genes that underwent a dynamic change as a result of the injury were assigned a score for each time point (see Methods). Figure 6c illustrates the results of the Time point—Weighted—Method approach, where the dark blue graphs in each subplot indicate the training dataset profiles for the different times after injury. A time point signatures score reflects the number of genes for which the test sample gene profile matches a training sample—both were upregulated, downregulated, or unchanged relative to the controls. This number of matching genes is weighted by the classification power of the genes—i.e. a gene that is upregulated at only two time points has greater power for classification compared to a gene that is upregulated at 7 of the time points. For the resulting scores, a difference of over 100 weighted genes between adjacent time points indicates a high confidence algorithm call. A score difference of 50 to 100 indicates a medium confidence call and score differences are noted along the y-axis of Fig. 6c. Figure 6c also shows the profiles for the injured 3 hours, 672 h, and to a lesser extent 504 h are highly similar to the uninjured control profiles. A Pearson correlation of 0.931 was observed between the injured 3 h training profiles and the uninjured control, 0.98 between the 504 h profiles and the uninjured controls, and 0.996 between the 672 h profiles and the uninjured controls, respectively. In the test data, the uninjured control and injured 3 h datasets follow the training data closely, while the 10 h and 168 h samples deviate from the training data.

The time point-weighted signatures method assigns normalized weights to the magnitude of the fold change between the injured and control samples and used the calculated weights to match gene profiles between training and blinded samples (see Methods). Using this approach, specific genes whose changes in expression are useful for classifying a blinded time point could be determined. Examples of the representative genes include TCDD-Inducible Poly(ADP-Ribose) Polymerase-Tiparp (fold change = 3, P-value = 0.011) at 3 h, FOS-Like Antigen 1-Fosl1 (fold change = 13.06, P-value = 0.0166) at 10 h, Nicotinamide Riboside Kinase 2-Nmrk2 (fold change = 3.01,P-value = 7.05e-5) at 24 h, Insulin-like Growth Factor 2 Receptor-Igf2r (fold change = 3.18, P-value = 2.34e-4) at 48 h, Interferon-Induced Protein 35-Ifi35 (fold change = 4.31, P-value = 1.44e-4) at 72 h, Phosphoglucomutase 5-Pgm5 (fold change = 2.90, P-value = 1.88e-3) at 168 h, Collagen Type VI Alpha 6-Col6a6 (fold change = 8.02, P-value = 4.95e-5) at 336 h, and Myosin Light Chain 10-Myl10 (fold change = −3.68, P-value = 1.72e-2) at 672 h. These genes had a high fold change for a single time point, and low fold changes of injured versus control for each of the other time points. Genes such as these were then selected to differentiate between time points because pairwise profile comparisons at adjacent time points are of greater interest than the global expression profile of a gene across multiple time points.

### Overall Gene Classification Schema Performance

**Figure 7:**
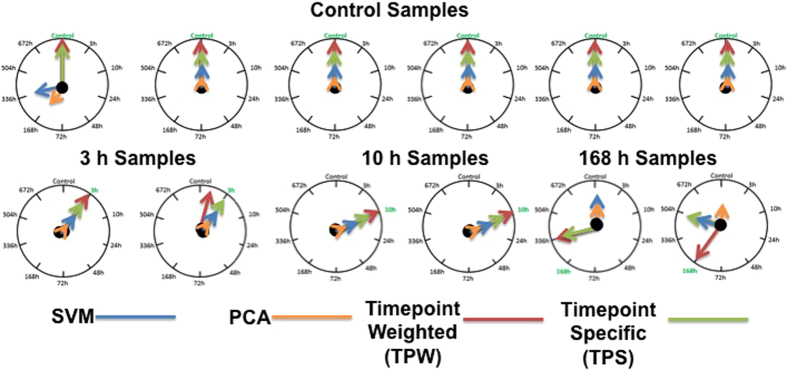
Sample classification results from four bioinformatics classification methods—support vector machine with linear kernel (blue arrows), principal component analysis (orange arrows), time point weighted signatures method (red arrows), time point-specific signatures method (green arrows). The arrows indicate the time point reported by each of four methods with highest confidence. Twelve blinded samples, corresponding to four time points, were analyzed: 6 control samples, two 3 h samples, two 10 h samples, and two 168 h samples.

The overall performance of the four sample classification methods is illustrated in Fig. 7. All of the methods incorrectly predicted one of the injured 168 h samples as either injured 336 h (both the Time point-Weighted and Time point-Specific methods) or as an uninjured control (PCA and SVM methods). The other injured 168 h test sample was also incorrectly predicted by 3 of the methods. This result is due to variability in the training data from the 168 h injured samples. Overall, the results from these classification schemas suggest that the time point signature approaches outperformed the SVM and PCA classifiers by 10 percent, with time point weighted signatures method performing better than time point- specific signatures method.

To further probe into the origin of the variability observed from the RNA-Seq data at 168 h, quantitative PCR (qPCR) for multiple genes (n = 25) and biological replicates from different samples was performed (Supp. Fig. 2). Comparison of four biological replicate experiments with qPCR and the RNA-Seq results showed that the determined expression values were strongly correlated (R^2^ = 0.88), indicating the observed variation at 168 h is biologically representative. Further inspection of the genes from the 168 h time point that contributed the highest loadings to the overall variance showed enrichments in several pathways such as angiogenesis, ECM remodeling, immune response and endocytosis (Supp. Table 3). These pathways are consistent with neighboring time points (72 h and 336 h), as well as reinforce the observation of proliferating myogenic precursors remodeling their niche and myoblasts undergoing differentiation since differentiating myoblasts promote angiogenesis^50^. Collectively, these findings support the conclusion that the data observed from the 168 h time point is biologically representative even though the training and test data replicates for the 168 h time points exhibited a slightly lower Pearson correlation of 0.90, compared to a correlation over 0.95 for replicates from the other time points. One possible explanation for the anomalous behavior from the 168 h time point is that the collection of profiled cells may be in multiple states. In support of this hypothesis, profiling single cells through myogenic differentiation under homogenous conditions revealed high cell-to-cell variation and transcriptional changes over variable time scales^51^. Since the RNA-Seq profiles represent an average of measurements, with the most abundant cell type contributing the largest component of the composite expression value, variations in expression through time from single cells will add heterogeneity. Furthermore, as discussed above, at the 168 h time point we view signatures of proliferating myogenic precursors and differentiating myoblasts, both of which contribute differently to the merged expression value. Thus, the variability from the 168 h may be due to sampled myogenic cells at different stages of differentiation.

The four classification methods gave inconsistent results for the injured 168 h samples, suggesting that cells at that time may be undergoing transition states that lead to high variability between replicate samples. Though the injured 168 h samples were challenging to classify, the successful classification of multiple uninjured control samples, injured 3 h, and injured 10 h datasets advances the overarching goal of identifying easily accessible biomarkers for healing status and early triage. The ability to classify a small volume of tissue such as a muscle biopsy from a fine-needle aspirate to the correct post-injury time point serves as a step toward the eventual goal of translating molecular ontology networks into quantitative diagnostics.

### Classification Analysis at the Pathway Level

The time point classification analysis with the PCA, SVM, and time point signature methods was repeated at the pathway level to identify if gene pathways could differentiate time points after injury with greater accuracy than at the gene level. Generally, analysis at the pathway level carries less statistical power than analysis at the gene level, as pathway expression values are the mean of the expression values of individual genes, and consequently have a greater amount of noise. The SVM approach (results not shown) led to a number of samples misclassified at the pathway level. Supplemental Figure 10 shows PCA also did not perform as well at the pathway level, however, the errors that were made by the classifier were generally for datasets from adjacent time points (injured 10 h datasets classified as 24 h, injured 3 h datasets classified as an uninjured control). This enables classification of samples to an “early”, “middle”, or “late” category, as delineated in Supplemental Figure 10a.

**Figure 8:**
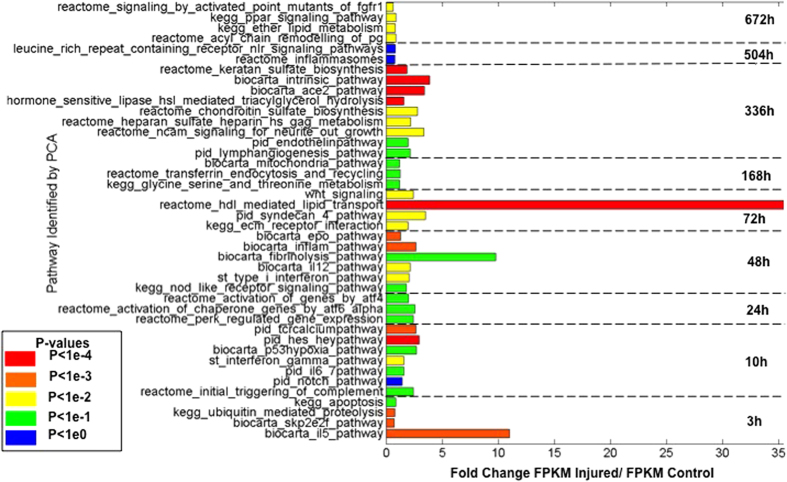
Pathways for each time point with the highest PCA loadings. The length of each bar denotes the fold change of the injured sample over the control at the time point. The color of each bar denotes the p-value for change in pathway activation level. Data derived from 12 test samples.

The Time Point-Specific and Time Point-Weighted signatures method at the pathway level were able to classify 10 of the 12 test samples correctly (Supp. Figure 10b), with an uninjured control dataset misclassified as an injured 168 h dataset, and an injured 168 h dataset misclassified as an uninjured control dataset (Supp. Figure 10b). These were the same samples misclassified by performing the analysis at the gene level. The weighted loadings of the pathways in component space were used to identify a set of pathways that contribute the most to variation across the time points (Fig. 8 and Supplementary Figure 11). A number of these are associated with inflammation, the immune response and cell death for the early time period (IL-6 and Interferon- Gamma signaling, monocyte activation, triggering of coagulation and complement, apoptosis and hypoxia). In the middle phase, elements that regulate the immune system are still active (Nod-Like Receptors, Type 1 interferons, IL-12 signaling), while fibrinolysis and ECM remodeling (cytoskeletal protein cleavage, cell junction organization), cellular differentiation (Wnt signaling and syndecan-4 pathway) in addition to numerous metabolic pathways (HDL- mediated lipid transport pathways, threonine metabolism pathway) become significantly over- expressed. In the late phase, angiogenesis and endothelin pathways are activated in addition to neural regeneration pathways (NCAM signaling) as well as pathways associated with cellular adhesion and myoblast fusion, ECM remodeling and metabolism (integrin-cell surface interactions, GAG metabolism, chondroitin sulfate – dermatan sulfate metabolism). Many of the identified time point specific pathways matched the pathways discovered during the GSEA and DAVID analysis on the training RNA-seq data highlighted above.

## Discussion

Multiple gene expression programs and pathways have been linked to muscle repair and regeneration, each of which acts with differing kinetics and degrees of activation for different individuals. This feature, in conjunction with limitations of available imaging tools, has prevented quantitative classification of healing progression for individuals who sustain a LLMI. Herein, high-resolution RNA-Seq was utilized to track the different stages of healing after a traumatic injury and using this approach, multiple types of cellular programs that confer different properties to the muscle repair and regeneration system were able to be monitored through time. In contrast to imaging modalities that provide low resolution and little information on the various gene expression programs, accurate classification of uninjured and injured tissues was carried out without a priori knowledge. Several different bioinformatics classification methods were utilized to dissect the genomic datasets and metrics of performance for each schema were assessed. This methodology may help clarify or further enable diagnosis of how a given patient is progressing towards healing after a traumatic injury as well as enable a clinician to determine the relative timing of different muscle repair and regeneration networks that potentiate a return- to-activity decision. Moreover, the approach can be coupled to guide treatments and evaluate therapeutic efficacies.

The RNA-Seq results demonstrated that the injury site is highly dynamic with multiple gene expression programs contributing to healing progression, including several with antagonistic behavior. To gain further insight into the transcriptional networks, pathway analysis was performed and showed the networks progressively migrating from a pro-inflammatory protective state in the early period after the injury to an anti-inflammatory, supportive state in the middle and late time periods. The observed networks are consistent with previous studies and highlight the possibility to quantitatively track healing progression via transcript profiling using high- throughput sequencing. To test the robustness of the generated datasets and the ability to classify a given sample, additional datasets were generated and the corresponding time points after the injury were blinded. Four different bioinformatics techniques were then utilized to classify the blinded samples and the performance of the classification schemas was quantified. The novel time point signature approaches outperformed the SVM and PCA classifiers and the difference in performance of the two time point signatures approaches suggests the need for an optimized model to weight the dot products across pairwise sample comparisons. The model for time point weighted signatures method performed better because it accounted for FPKM expression levels for different genes in addition to the number of time points for which a given gene exhibited a fold change. A future direction of research might aim to improve this model via a grid search algorithm designed to determine the optimal set of weights for a given gene profile^52^.

The four classification methods gave inconsistent results for the injured 168 h samples, suggesting that cells at that time may be undergoing transition states that lead to high variability between replicate samples. The variability from these states makes a single profile more difficult to determine for later stages of muscle repair and regeneration. To further develop predictive power for these time points, single cell transcriptomic profiling^51^ or sampling of multiple locations from the injury site may enable better fidelity at predicting healing trajectories and outcomes during the regenerative phase after injury. Though the injured 168 h samples were challenging to classify, the successful classification of multiple uninjured control samples, injured 3 h, and injured 10 h datasets advances the overarching goal of identifying easily accessible biomarkers for healing status and early triage. The ability to classify a small volume of tissue such as a muscle biopsy from a fine-needle aspirate to the correct post-injury time point serves as a step toward the eventual goal of translating molecular ontology networks into quantitative diagnostics.

## Materials and Methods

All experimental protocols were approved by the USARIEM Institutional Animal Care and Use Committee (IACUC).

### Animals & Traumatic Injury Model

Male C57BL/6J mice (10 weeks of age, 24–27 grams) were obtained from The Jackson Laboratory (Bar Harbor, ME). Mice were housed one per cage (shoebox cage, 7″ × 11″ × 5″ h) in the USARIEM animal facility at a constant Ta = 24 ± 1 °C, 50% relative humidity, with a 12 h/ 12 h (0600–1800 h) light/dark cycle. Standard laboratory rodent chow and water were provided ad libitum. Cages were supplied with Alpha-dri and cob blend bedding for nesting and enrichment and plastic houses for warmth and comfort. Food intake and body mass were recorded daily. Mice were cared for in accordance with the Guide for the Care and Use of Laboratory Animals in a facility accredited by the Association for the Assessment and Accreditation of Laboratory Animal Care (AAALAC).

Prior to administration of the freeze injury, mice were anesthetized with a combination of fentanyl (0.33 mg/kg), droperidol (16.7 mg/kg), and diazepam (5 mg/kg). The TA muscle was exposed via a 1 cm long incision in the aseptically prepared skin overlying the TA muscle. Freeze injury was performed in the left, hind limb. The non-injured contralateral leg served as one control. Freeze injury was induced by applying a 6 mm diameter steel probe (cooled to the temperature of dry ice, −70C) to the belly of the TA muscle (directly below incision site) for 10 seconds. Following injury, the skin incision was closed using 6-0 plain gut absorbable suture(Ethicon, Piscataway, NJ). The analgesic, Buprenorphine (0.1 mg/kg SQ) was administered using a 25–27 gauge needle prior to recovery from anesthesia.

Mice were euthanized at each time-point post-injury (3, 10, 24, 48, 72, 168, 336, 504, 672 h) via CO_2_ inhalation (2 liters/min), thoracotomy and exsanguination. TA muscles were removed from the injured and contralateral limb; weighed, and a portion of the tissue was homogenized in Trizol, snap frozen in liquid N_2_, and stored at −80 °C.

### RNA-Isolation and Sequencing Library Preparation

Total RNA was isolated from the homogenized tissue in Trizol using the miRNeasy Mini Kit (Qiagen) as per the manufacturer’s instructions. RNA concentration and integrity were measured with a Nanodrop spectrophotometer (Nanodrop 2000c) and Bioanalyzer (Agilent 2100). If a sample did not pass quality metrics for further processing (RIN >7), the samples were omitted from further processing. This quality check resulted in several time points that only had two tissues, such as the 3 hour and 168 hour time points, or the 48, 336, 504 and 672 hour time points, which only had three tissues. At least 1 μg of isolated total RNA was used to produce strand-specific cDNA libraries using the Truseq protocol, as per the manufacturer’s instructions and previously described^53^. Individual libraries were pooled and sequenced using twelve lanes of 76-base paired reads on an Illumina Genome Analyzer IIx.

The RNA-seq datasets were separated into a training dataset and a test dataset. The training datasets consisted of 37 control samples, two injured 3 h samples, five injured 10 h samples, four injured 24 h samples, three injured 48 h samples, four injured 72 h samples, two injured 168 h samples, three injured 336 h samples, three injured 504 h samples, and four injured 672 h samples.

### RNA-Seq Data Processing

RNA data in BAM format was aligned to the reference mouse genome (mm9) using the TopHat aligner. All analysis was performed using the mm9 mouse assembly and annotation as reference. The aligned reads were then analyzed with the Cufflinks 21 software suite (v2.1.1). The Cufflinks tool was first used to assemble transcripts for each replicate and time point. Separate assemblies were generated for injured and controlled conditions. Next, Cuffmerge was applied to the assembled transcripts to create a single merged transcriptome annotation for each condition (injured or control). Third, Cuffdiff was used to find differentially expressed genes and isoforms across time points and conditions, as well as detect differential splicing and alternative promoter usage. Cuffdiff was executed using the merged transcriptome assembly along with BAM files from the TopHat tool for each individual replicate. Last, the CummeRBund R package was used to compute statistics on differentially expressed genes and isoforms. All reference information for Mus musculus was downloaded from the Illumina iGenome site: http://cufflinks.cbcb.umd.edu/igenomes.html. The UCSC mm9 build was utilized with Cufflinks and Cuffmerge.

### Data Filtering

Gene and isoform FPKM values derived via the Cufflinks analysis were filtered to remove uninformative replicates. A pairwise Pearson correlation was computed across replicates for a time point (3 h – 672 h) for each condition (control, injured). Any replicate that did not correlate with all other replicates with R^2^ ≥ 0.95 was excluded from the analysis. Replicates were merged into aggregate gene and isoform expression values. The median FPKM was computed across each set of replicates. If this value was 0, the aggregate FPKM for the time point/condition was set to 0. Otherwise, the mean FPKM was computed across the replicates. The data was further filtered to limit the analysis to genes and isoforms with a significant change in expression. To meet this requirement, a gene/isoform was required to exhibit FPKM >=1 at one or more of the 9 time points with a q value less than 0.05. Additionally, the gene/isoform must have undergone a two-fold (or higher) fold change in FPKM at one or more time points. These criteria resulted in 5,668 significant genes, as described in the “Global Transcriptional Dynamics” section, as well as 7,258 significant isoforms.

### Gene Set Enrichment Analysis

Filtered replicates were analyzed with the standalone version of the GSEA^54^ (Gene Set Enrichment Analysis) tool (v. 2.0.8). Candidate gene sets were selected from the Molecular Signatures Database version 4.0 (MSigDB), filtering by “Mus musculus” organism and “MOUSE_GENE_SYMBOL” chip. The analysis was refined by focusing on gene sets associated with the reactome. The analysis was performed using a categorical phenotype .cls input file, comparing all 31 injured replicates to all 37 control replicates. This was followed by a time series analysis using a continuous .cls input phenotype. Each profile in this phenotype corresponded to gene upregulation at a single time point. GSEA identifies gene sets that are correlated and anticorrelated with a continuous profile, and the anti-correlated gene sets were interpreted as down-regulated for the given time point. GSEA was executed with 1000 permutations, no collapsing of datasets to gene symbols, since the input data file had been filtered as described above in the “Data Filtering” section to avoid multiple probes per gene, and the gene set permutation type. For the categorical phenotype, genes were ranked using the Signal2Noise metric, whereas for the continuous phenotype genes were ranked via the Pearson correlation metric. Gene sets with more than 500 genes and fewer than 15 genes were excluded from the analysis. The resulting gene sets were filtered by FDR value – a cutoff of 0.05 was used in determining significant gene sets.

### Functional Annotation of Differentially Expressed Genes

Differentially expressed genes, as found by the Cuffdiff analysis described above, were grouped by time point. These gene groups were than analyzed with the DAVID Functional Annotation Tool^55^. The “GOTERM_BP_FAT”, “GOTERM_MF_FAT”, and “KEGG_PATHWAY” annotation criteria were selected with Bonferonni multiple testing correction. Differentially expressed genes were also analyzed with the Generic GO Term Finder tool from the Lewis- Sigler Institute for Integrative Genomics^55^,^56^. Significantly enriched GO Terms (FDR < 0.05) were clustered with the Gephi software tool (v.0.8.2) to discover major functional groups.

### Alternative Splicing Analysis

The MISO software (0.5.2) was used to identify genes that underwent alternative splicing^57^. The mouse mm9 alternative event annotation file (v 1.0) was downloaded from the MISO Web site for alternative splicing analysis. Results were filtered by significance and order or magnitude of expression change, using cutoff values suggested in the MISO documentation. The set of differentially expressed events generated by MISO was filtered such that 1) events have at least 1 inclusion read, 2) events have at least one exclusion read, 3) the sum of inclusion and exclusion reads is at least 10, 4) the delta Psi is at least 0.20, 5) the Bayes factor is at least 10. The set of filtered events was visualized using the Sashimi software, using the number of reads in each sample to normalize the read density for RPKM calculation.

### Sample Classification

For each of the four methods, the RNA-seq data was filtered in several ways to determine the optimal subset of data to use for correctly predicting the unknown time point^58^. In the first approach, the full set of 23,811 mouse genes was reduced to the 5,794 genes that were differentially expressed at one or more time points. A log_2_ fold change in FPKM between an injured time point sample and the corresponding control, as well as a minimum FPKM of 0.5 at one of the 10 time points was used as the criteria to determine differential expression. As an alternative filtering technique, only genes that were differentially expressed at a single time point (2,319 genes) were used for the analysis. In the third filtering approach, isoforms rather than genes were examined, and an initial set of 30,564 isoforms was filtered to a set of 7,541 isoforms with differential expression. In a fourth approach, this set of 7,541 isoforms was further filtered to a set of 1,908 isoforms that exhibited differential expression at only a single time point. For each of these filtering approaches, the four classification techniques discussed below were applied to both the raw FPKM values in the set of genes/isoforms, and to the log_2_ fold change of injured/control.

### Support Vector Machine

The “one vs. one” and “one vs. all” approaches were used to train a cascade of support vector machines to classify the blinded RNA-Seq time points^59^. In the “one vs. one” approach, the support vector machine functionality of the MATLAB (v2013a) Machine Learning Toolkit was utilized to train a support vector machine classifier with a linear kernel to distinguish between each pair of time points. A sample was analyzed with the full set of 45 SVM classifiers. Each SVM call was weighted by the distance of the support vectors from the separating hyperplane. This distance was computed with Equation 1^60^, whereby a decision hyperplane is defined by an intercept term b and a decision hyperplane normal vector 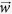 that is perpendicular to the hyperplane. The set of training data is represented as 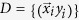, where each member is a pair of a vector 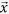 and a class label y_i_ assigned by the support vector machine. 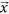 is the vector composed of all gene expression values corresponding to a single sample. R represents the distance of 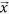 from the separating hyperplane.


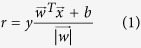


The weighted SVM calls from each pair of classifiers were summed, and the time point with the highest number of weighted votes was designated as the final classification call^61^,^62^. In the contrasting “one vs. all” approach, the control samples formed one class and the nine injured time point samples formed a second class^63^. An SVM was used to determine whether a blinded sample was closer to a control or an injured sample. If closer to an injured sample, the process was repeated—each time point was compared to the full set of remaining time points, excluding time points one by one until the most likely time point was determined. This approach did not perform as well as the one vs. one approach.

### Principal Component Analysis

Principal component analysis was performed on the data using the Statistics Toolbox in MATLAB (v.2013a)^64^. A matrix was formulated such that the each row contained the FPKM values for the full set of genes for each sample. Equivalently, each column contained the FPKM expression profile for a single gene across all the samples. The z-score of each expression profile was computed to normalize the data. The original data were transformed to the space of principal components. For each test sample, the distance formula in N dimensions (N = number of principle components) was applied to determine the nearest training dataset in PCA space. The test dataset was classified to belong to the same time point (or control) as the nearest sample^65^. PCA analysis was also used to determine the set of genes/isoforms that contribute the most to the variance between expression profiles for the time points^66^. A dot product was computed between the principal component coefficients (loadings) for each component and the eigenvalues of the covariance matrix of the data. The elements with the highest weights in the resulting matrix correspond to the genes that contribute the most to the variance between the time points^67^.

### Comparison of Weighted Gene Expression Profiles

Replicates for each time point in the training dataset were combined. If the median FPKM value across the replicates was zero, the combined FPKM was set to 0. Otherwise, replicate FPKM values were averaged for each time point. Two approaches were implemented to characterize the training data expression profiles.

### Time Point-Specific Signatures Method

In the time point-weighted signatures method, any gene with FPKM greater than or equal to 0.5 and a logfold change in expression greater than 2 was assigned a score equal to one over the number of time points where the gene exhibited a significant change in expression. The number of time points with a significant change in expression was calculated separately for upregulated (injured/control) and downregulated genes. For example, if a gene was upregulated at 3 h and 10 h after injury and downregulated 504 h after injury, the weighting would be ½ for the upregulated time points and 1 for the downregulated time point. The gene expression profile of a test dataset was then compared to each of the profiles for the training dataset. A match score of zero was initialized for each comparison training dataset. In the case of a match (i.e. a gene was upregulated in both the training and test dataset, downregulated in both datasets, or did not exhibit a significant change in expression in either dataset), the score was incremented by the upregulated or downregulated weight of the gene. If a gene did not exhibit a significant expression fold change in either dataset, the match score was incremented by 0.5. This number was determined via least squares regression as the optimal weight to assign in the case of no fold change, based on the composite profile of the training data. Ultimately, the test dataset was classified as the time point with the highest match score in the training data.

### Time Point-Weighted Signatures Method

The time point-weighted signatures method differs from the time point-specific signature method in how weights are assigned to the dot product of gene expression vectors. Rather than weighting a gene in a training dataset based on the number of time points where the gene was upregulated or downregulated, weights were assigned based on the magnitude of the fold change between the injured and control samples. The maximum fold change in expression for the gene across the nine time points was determined. The fold change for all remaining time points (injured/control) was computed and normalized as a fraction of the maximum fold change. This normalized value was used to weight a match between a training and blinded sample at the gene of interest.

A set of genes was next identified where the training data profiles were upregulated with a fold change greater than 2 or downregulated with a fold change greater than −2 at a single injured time point. The genes were assigned a score of 1 (if upregulated) or −1 (if downregulted) at that time point, and a score of 0 at all other time points. This produced a matrix of scores (0 at time points with no significant change, 1 at time points with upregulated genes, and −1 at time points with downregulated genes). Subsequently, a similar matrix was computed for the testing datasets. Analysis was restricted to the subset of genes with a significant fold change at a single time point, as observed in the training data. Time points with a positive fold change greater than two were assigned a score of 1; time points with a negative fold change were assigned a score of −1; and the remaining time points were assigned a score of zero. The element-wise difference between the training and test score matrix was computed, and the time point in the test data with the smallest absolute distance from the training data was selected.

### Significant Gene Selection

The time point-specific signatures method was utilized to identify the set of genes most responsible between gene expression profiles across time points. In this method, genes with the highest weights are differentially expressed at only one of the nine time points, and are consequently most informative for distinguishing which time point a blinded sample belongs to.

### Gene Pathway Classification

Mus musculus pathways were selected from version 4.0 of the Broad Molecular Signatures Database (MSIGDB). In total 1320 pathways were examined. The mean pathway expression score was calculated by taking the mean FPKM expression value for all member genes in the pathway. For each pathway, a two-tailed T test was performed to determine the Z-score across the 9 time points. 1189 pathways with Z-scores greater than 2 or less than −2 (P-value < 0.05) were identified.

To find the pathways that account for the majority of the variance between the time points, Principal Component Analysis was performed on the pathway Z-scores following the approach described above for gene-level analysis. The pathways with the highest loadings for each time point were identified and plotted in Supplemental Figure 10a.

The normalized time points signatures algorithm was applied to the significant pathways. Pathways with a Z-score less than −2 or greater than 2, in conjunction with a fold change greater than 2 at one or more time points, were identified. The 370 pathways that met these criteria are illustrated in Supplementary Figure 11.

### Data Visualization

The normalized FPKM values computed by Cufflinks were used to generate heatmaps of upregulated and downregulated genes for the early (3 h – 24 h), middle (48 h – 168 h) and late period (336 h – 672 h) phases. The data was filtered to include only genes with FPKM >1 at one of the time points, and significant genes were identified. These had a fold change of 2 or higher at one of the time points in the early, middle, and late phases. The log_2_ (fold change) values were clustered into heatmaps using the R heatmap.2 library.

### Histological Tissue Analysis

For histological analysis of damage to the tibialis anterior muscle, an additional cohort of C57BL/6J mice (n = 3 for each injury time point and Sham) were administered Evans blue dye (EBD) (1% weight/volume solution to yield a 1 mg EBD/10 g of body weight) intraperitoneally two hours prior to experimental injury or sham treatments. At each collection time point muscles from injured and non-injured limbs were collected for analysis as described above. The removed tibialis anterior muscles were then fixed in 4% paraformaldehyde phosphate buffer solution (PBS) for 2 hours and then washed three times in ice-cold PBS. The fixed samples were then placed in cryomolds (tissue tek) and covered in Optimal cutting temperature compound, (OCT: Tissue Tek, Sakura Finetek U.S.A. Torrance, CA). The OCT covered samples were then frozen in semi-frozen isopentane before storage at −80 °C before processing. Muscle samples were processed into 8-μM thick sections on a cryostat (Leica 1850uv, Leica Microsystems Buffalo Grove, IL) and adhered to slides (Colorfrost plus, Fisher scientific Hampton, NH). Each section was stained with skeletal muscle actin antibodies (15265 Abcam, Cambridge, MA) and goat polyclonal anti-rabbit IgG conjugated with fluorescein isothiocynate (FITC) (life technologies, Carlsbad, CA) and the nuclear stain 4′6-diamidino-2-phenylindole (DAPI) (life technologies, Carlsbad, CA). Sections were visualized on a LSM 700 (Carl Zeiss, Oberkochen, Germany). EBD was detected with laser excitation provided at a wavelength of 540 nm and emission collections at 590 nm. EBD positive cells were counted at 20X magnification. The percentage of EBD-positive areas was calculated by dividing the area of EBD staining by the total skeletal muscle area as defined by skeletal muscle actin. The fraction of skeletal muscle staining positive for EBD was used to calculate the percentage of muscle that was injured.

## Additional Information

**How to cite this article**: Aguilar, C. A. *et al*. *In vivo* Monitoring of Transcriptional Dynamics After Lower-Limb Muscle Injury Enables Quantitative Classification of Healing. *Sci. Rep*. **5**, 13885; doi: 10.1038/srep13885 (2015).

## Supplementary Material

Supplementary Information
