# Supplementary material for: In vivo Monitoring of Transcriptional Dynamics After Lower-Limb Muscle Injury Enables Quantitative Classification of Healing
Source: Sci Rep. 2015 Sep 18;5:13885. doi: 10.1038/srep13885 (PMC4585378; doi:10.1038/srep13885)
Supplement: Supplementary Information [file srep13885-s1.pdf]

In vivo Monitoring of Transcriptional Dynamics After Lower-Limb Muscle Injury Enables Quantitative Classification of Healing

Carlos A. Aguilar[1]*, Anna Shcherbina[1], Darrell Ricke[1], Ramona Pop[2], Christopher T. Carrigan[3], Casey A. Gifford[2], Maria L. Urso[3,†], Melissa A. Kottke[3], Alexander Meissner[2]

Supplementary Materials

Text S1: Transcriptional Signatures of Inflammation and Immune System Detected in Early Period

Text S2: Transcriptional Signatures Associated With Injured Muscle Tissue Extracellular Matrix

Text S3: Injured Muscle Tissue Microenvironment Regulates Existing and Invading Cellular Migration, Proliferation, Differentiation and Phenotype

Text S4: Activation of Muscle Repair Machinery

Text S5: Late Transcriptional Programs Activated in Response to Traumatic LLMI

Fig. S1. Immuno-histological analysis of muscle after cryo-injury.

Fig. S2. a) Example of reproducibility from RNA-Seq datasets. Biological replicates of *in vivo* isolated RNA samples from 24 hours after injury. Sequencing libraries were prepared from the isolated RNA and a strong correlation (R^2 = 0.95) was observed for two representative examples. b) Validation of RNA-Seq data with qPCR comparing the injured versus uninjured samples for the 3 hour time point. c) Validation of RNA-Seq data with qPCR comparing the injured versus uninjured samples for the 10 hour time point. d) Validation of RNA-Seq data with qPCR comparing the injured versus uninjured samples for the 24 hour time point.

Fig. S3. Number of differentially expressed genes.

Fig. S4. Gene Sets Activated Early After Injury (3-24 hrs) a) Enriched KEGG pathways from differentially expressed genes for the early time points (3-24 hrs). The size of the circle corresponds to the number of significant genes with each enriched pathway. b) Heatmap of genes associated metabolism expressed in the early time period. c) Gene expression profile of Arginase 1 (Arg1), which is a molecule emitted by type 2 macrophages to balance inflammatory reactive-oxygen species present in the injured tissue. Red-Injured, Blue-Uninjured

Fig. S5. Examples of alternative splicing in immune network of genes.

Fig. S6. Genes associated with hematopoietic activation, proliferation and anti-inflammatory cytokines activated in early & middle periods.

Fig. S7. Gene sets associated with extra-cellular matrix remodeling & satellite cell activation.

Fig. S8. a) Enriched KEGG pathways from differentially expressed genes for the late time points. The size of the circle corresponds to the number of significant genes with each enriched pathway. b) Heatmaps of gene sets activated in late period categorized by function.

Fig. S9. Area plots of FDR values for Gene Annotation (GO) clusters over time. GO terms were separated into major functional categories, and the IQR was computed for the terms in each group. The grey lines indicate the 25th and 75th percentiles, red lines denote the 25% of gene FDR values, the blue line denotes the median FDR value, and the green line denotes the 75% for FDR.

Fig. S10. Pathway-level scoring results for test datasets from PCA and the timepoint signatures methods. a). Principal component analysis. b) Normalized time point signatures method applied to mean pathway expression values. Timepoint signatures for 6 test samples and a truth training sample. The green curve indicates the control sample that was misclassified by the algorithm at the pathway level. Time point signatures for the 9 time points (blue lines) as well as 6 test samples (red and green lines).

Fig. S11. Differentially expressed gene pathways in injured samples over time.

Table S1. Significant GO clusters (FDR <0.05) identified for differentially expressed genes through the DAVID annotation tool. Each tab represents the annotations derived for each timepoint, clustered by function and sorted by FDR value from most to least significant.

Table S2: KEGG pathways identified for differentially expressed genes (FDR < 0.05). Pathways are listed for each timepoint and sorted by FDR value from most to least significant.

Table S3. Significant GO clusters identified from the 168h time point that were obtained from differentially expressed genes that were determined to contribute the largest variance at that time point.

Text S1: Transcriptional Signatures of Inflammation and Immune System Detected in Early Period

Pro-inflammatory cytokines and chemotactic protein members: IL-1β, IL-6, IL-18, Tnfα, Osm, Ccl2, Ccr2, Cxcr2, Trem2, S100a8, FDR=2.21e-22 - GO:0034097, response to cytokine

Anti-inflammatory genes: Socs3, IL-1rn, IL-4rα, IL-10rα, IL-13rα1, FDR=4.31e-10 - GO:0050728, negative regulation of inflammatory response

Invading immune cell genes: Cd68, Ly6c, Cd14, Cd163, Mrc1, Ptprc, Cd24a, Cd63, Itgam, Clec7a, Ltf, Irf7, Hp, FDR=7.6e-7 - GO:0050900, leukocyte migration involved in inflammatory response

Cytokines and chemokines: Ym1, S100a9, Spp1, Ccr1, Ccl3, Ccl11, Lcn2, Cxcl5, Ccl12, FDR=3.8e-44 - GO:0001816, cytokine production, FDR=9.05e-14 - GO:0032602, chemokine production

Signaling lymphocytic activation molecule family: Slamf7, Slamf8, Ly9, FDR=3.44e-16 - GO:0046649, lymphocyte activation

Pro-apoptotic loci: Casp1, Casp4, Casp8, Apaf1, Fas, FDR=1.40e-9 - GO:2001235, positive regulation of apoptotic signaling pathway

Anti-apoptotic loci: Xiap, Birc3, Birc5, Api5, Bax, Mcl1, Bcl2l1, FDR=2.17e-23 - GO:0043066, negative regulation of apoptotic process

Text S2: Transcriptional Signatures Associated With Injured Muscle Tissue Extracellular Matrix

Structural components of extra-cellular matrix (ECM) remodeling: Col1a1, Col1a2, Col3a1, Col5a1, Col5a2, Col5a3, Col6a1, Col6a2, Col6a3, Eln, FDR=4.19e-111 - GO:0016043, cellular component organization

Connective tissue: Fbn1, Ctgf, Has1, Hbegf, Fn1, Sparc, Tnc, FDR=6.65e-14 - GO:0061448, connective tissue development

ECM cross-linking enzymes: Lox, Loxl1, Loxl2, Plod3, Leprel2, P4hb

Small leucine-rich proteoglycans: Dcn, Bgn, Kera, Vcan, Lum, Ogn, FDR=3.69e-28 - GO:0036211, protein modification process

Adhesive glycoproteins: Pcolce, Itbpbp1, Lgals1, Dpt, Postn, Emilin2, Efemp2, Igfbp7, FDR=3.36e-16 - GO:0043062, extracellular structure organization

Tgf-β superfamily: Tgf-βi, Tgf-βr1, Tgf-βr2, Tgf-β1

Formation and stabilization of blood vessels and arterial repair: Ang, Angpt2, Angptl1, Angptl4, Angptl7, Cthrc1, Aif1, FDR=3.12e-10 - GO:0001525, angiogenesis

Contractile fibers: ActB, Actg, Capg, Arpc3, Cotl1, Tln1, Tagln, Vcl, RhoA, FDR=7.8e-5 - GO:0030833, regulate actin filament polymerization, FDR=1.5e-3 - GO:0032273, positive regulation of protein polymerization

Integrin-associated genes: Itga5, Itga7, Itgam, Itgav, Itgb1, Itgb2, FDR=3.34e-5 - GO:0007229, integrin-mediated signaling pathway

Mmps, Adams and Timps: Mmp2, Mmp3, Mmp8, Mmp14, Mmp19, Adam8, Adam15, Adam19, Timp1, Timp2, FDR=6.83e-8 - GO:0048771, tissue remodeling

Text S3: Injured Muscle Tissue Changes from Pro- to Anti-Inflammatory States Several Days After Injury

Innate immunity and microbial recognition: Tlr1, Tlr7, Tlr8, FDR=0.003 - GO:0034121, regulation of toll-like receptor signaling pathway

Antigen presentation and hematopoietic activation and proliferation: Ptprc, IL-21r, IL-17ra, Vav1, Lyz2, Csf1r, Csf2rb, Csf2ra, Il3ra, Il5ra, Ifi204, Aif1, FDR=1.4e-8 - GO:0042110, T cell activation, FDR=8.18e-19 - GO:0030097, Haematopoiesis

Anti-inflammatory cytokines: IL-1rl1, Il-4ra, IL-10rα, Msr1, IL-13ra1, S100a9, FDR=2.9e-10 - KEGG mmu04630, cytokine-cytokine receptor interaction, FDR=9e-3 - GO:0010934, macrophage cytokine production

Phagocytic and complement cascade genes: Cyba, Ncf1, Ncf2, Ncf4, C1qa, C1qb, C1qc, C1ra, C1s, FDR=3.37e-8 - GO:0006956, complement activation, FDR=7e-6 - GO:0006909, phagocytosis

Notch signaling: Dll1, Notch2, Myc, Rbpj, Cdkn1a, FDR = 3.69e-18, GO:0002684, positive regulation of immune system process

Bone morphogenetic proteins: Bmp1, Bmp4, FDR = 0.08, GO:0005125, cytokine activity

Actively proliferating cells: Cdk1, FDR = 9.63e-9, GO:0007049, cell cycle

Text S4: Activation of Muscle Repair Machinery

Satellite cell markers: CD34, Itga7, Itgb1, Sdc3, Sdc4, Sdcbp, Cav1, Cxcr4, Cdh15, Ly6a, FDR=4.933e-5 - GO:0007519, muscle tissue development

Muscle-fusion genes: Tmem8c, Cdh15, Capn2, Capns1, Myof, Cav3, Itgb1, Vcam1, Itga4, FDR=2.5e-6 - GO:0098602, single organism cell adhesion

Basement membrane genes: Col4a1, Col4a2, Dysf, Trim72, Lamc2, Cav3, Ahnak, Anxa1, Anxa2, Anxa5, FDR=4.78e-9 - GO:0061024, membrane organization

Muscle-specific actins, myosins, troponins, and tropomyosins: Actl9, Clrn1, Myh3, Myl4, Myl6b, Cald1, Tpm4, Tnni1, Tnni3, Tnnt1, Tnnt2, Tnnc1, Tpm3, Des, Vim

Transcription factors: Itgb2, Rrad, Fos, JunB, FosB, Pbx2, Pbx3, Dusp5, MyC, BMyc, Mt3, Egr1, Egr2, Sgms2, Runx1, Grn, Pdlim3, Csrp3, Usf1, Arid5b, Naca, Ankrd1, Ankrd2, Atf3, Atf4, Atf6, Xbp1, Tead4, Nfe2l2, FDR=2.46e-10 - GO:0006351, transcription, DNA-templated

Text S5: Late Transcriptional Programs Activated in Response to Traumatic LLMI

Chemotaxis genes: Ccl6, Ccl8, Ccl9, Cxcl16, Fcer1g, Fcgr3, FDR=1.9e-5

Mast cell activation: Fyb, Fcer1g, Fcgr3, Fcgr2b, Lat2, Lcp2, FDR=3.33e-5 - GO:0045576, mast cell activation

Fc receptors: Fcer1g, Fcgr1, Fcgr3, Fcgr2b, Clec7a, Colec12, Hck, Sirpa, Slc11a1, Vav1, FDR=0.035 for KEGG mmu04664, Fc epsilon RI signaling pathway.

Lectins: Clec12a, Clec4a1, Clec4a2, Clec4a3, Clec4n, Clec7a, Clec10a.

Collagen fibril organization: Adamts14, Col3a1, Col5a1, Col5a2, Dpt, Lox, and Anxa2, FDR = 1e-7)

ECM receptors: VLA proteins (α2, α5, α8, α9, α10, α11), cytoadhesins (β3, AlI-β), and proteoglycans (Cd44, Sdc3, Sv2), KEGG ECM-receptor interaction pathway, FDR = 5.7e-7

Angiogenesis genes: Col4a1, Col4a2, Robo4, Vash1, Sema5a, Sox18, GO:0001525, FDR = 3.24e-14

Muscle contraction and muscle system processes: Cacna1s, Myom1, Myh2, Myh4, Myh7, Nos1, Kcnma1, Ryr1, Trim63, FDR=6.7e-4 and 1.6e-3, respectively.

Ossification regulation: Gpnmb, Bglap, Sparc, Spp1, Runx2, Bmp1, Bmp5, Smad1

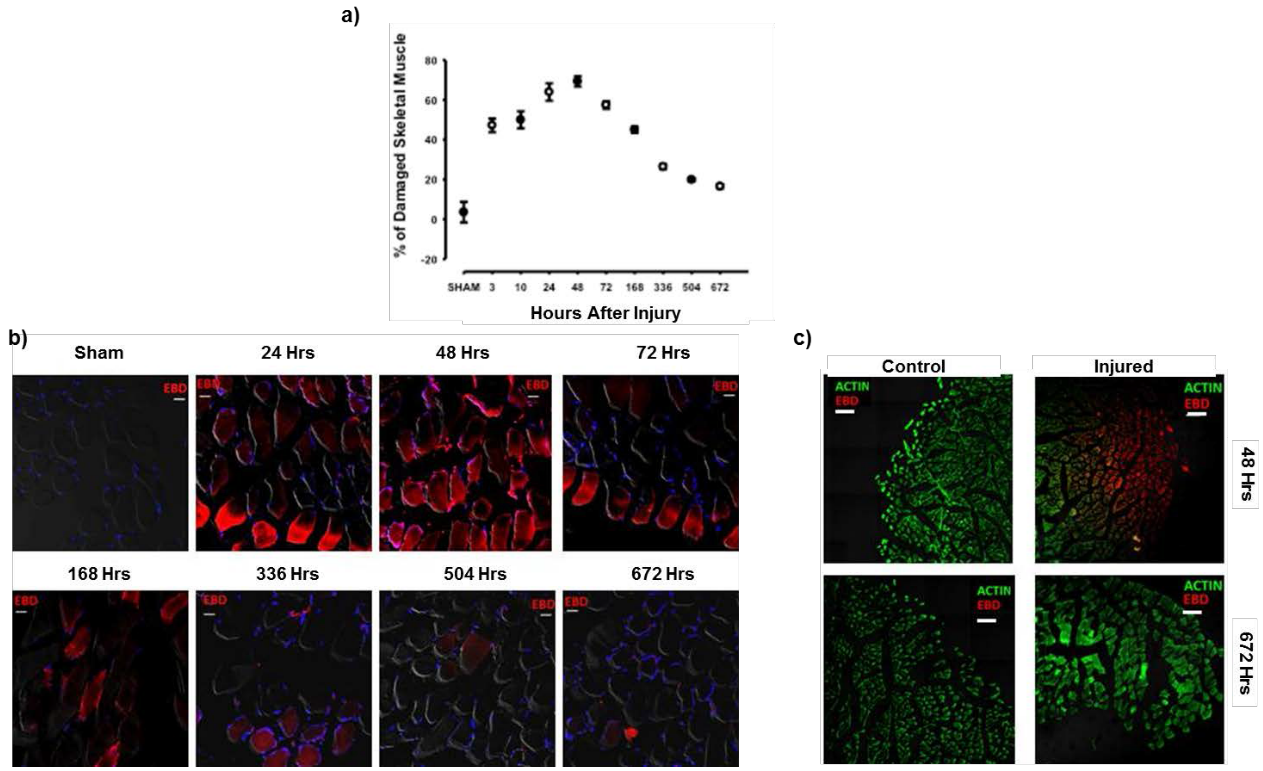

Fig. S1. Immuno-histological analysis of muscle after cryo-injury. a) Quantitative analysis of immuno-histological images revealed a gradual increase in tissue damage until 48 hours, indicating secondary damage to the muscle tissue occurred after the initial cryo-injury. b) Representative histological images of the injured TA stained with Evans Blue Dye (EBD – red) and DNA (blue) after several time points. c) Representative immuno-histological images at 48 hours and 672 hours after injury, whereby the green is stained for actin and red is stained for evans blue dye.

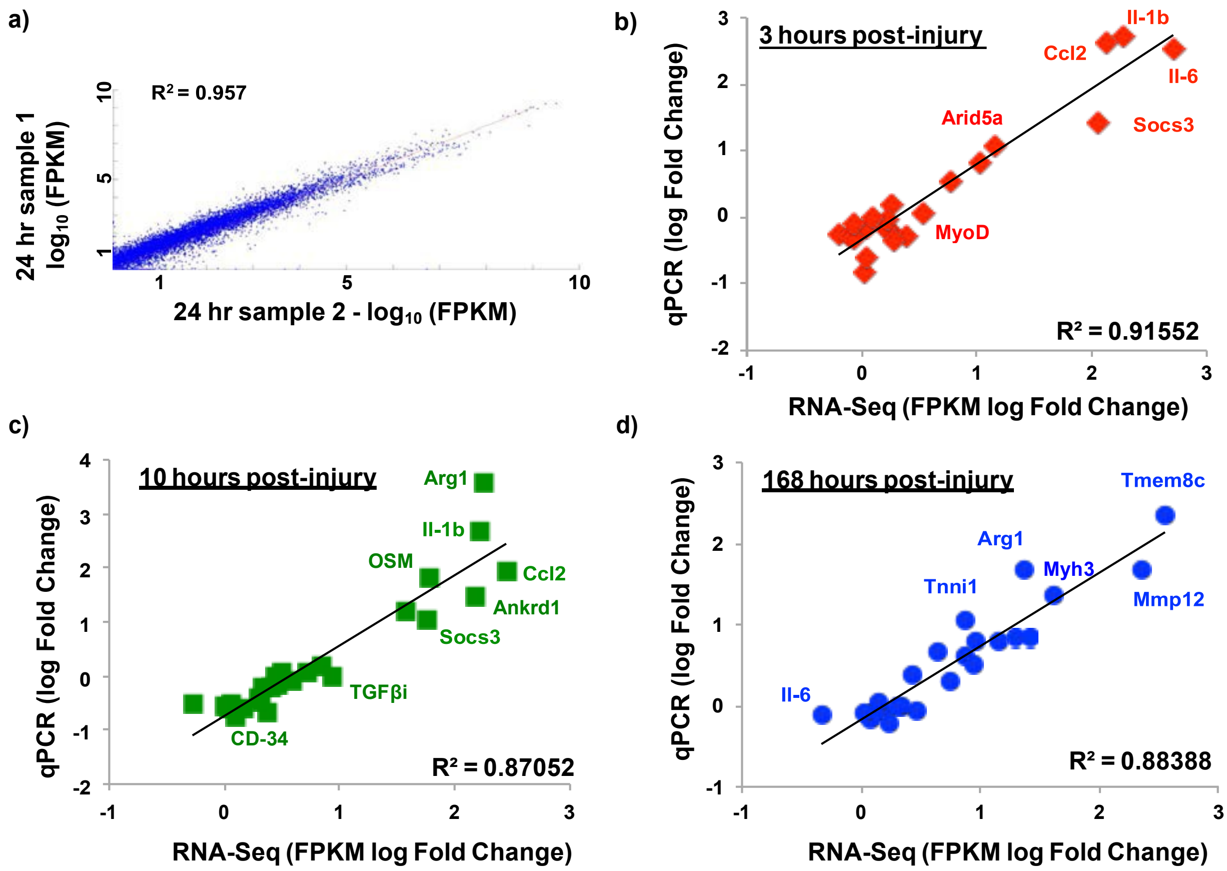

Fig. S2. a) Example of reproducibility from RNA-Seq datasets. Biological replicates of *in vivo* isolated RNA samples from 24 hours after injury. Sequencing libraries were prepared from the isolated RNA and a strong correlation ($R^2 = 0.95$) was observed for two representative examples. b) Validation of RNA-Seq data with qPCR comparing the injured versus uninjured samples for the 3 hour time point. c) Validation of RNA-Seq data with qPCR comparing the injured versus uninjured samples for the 10 hour time point. d) Validation of RNA-Seq data with qPCR comparing the injured versus uninjured samples for the 168 hr time point.

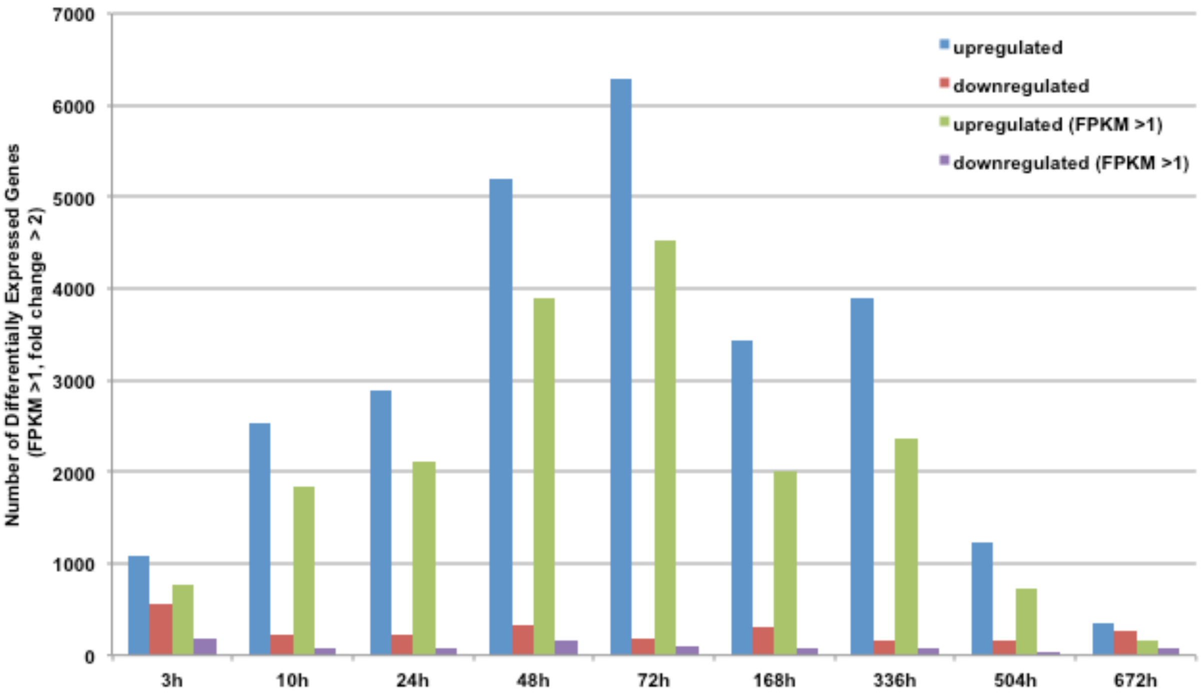

Fig. S3. Number of differentially expressed genes

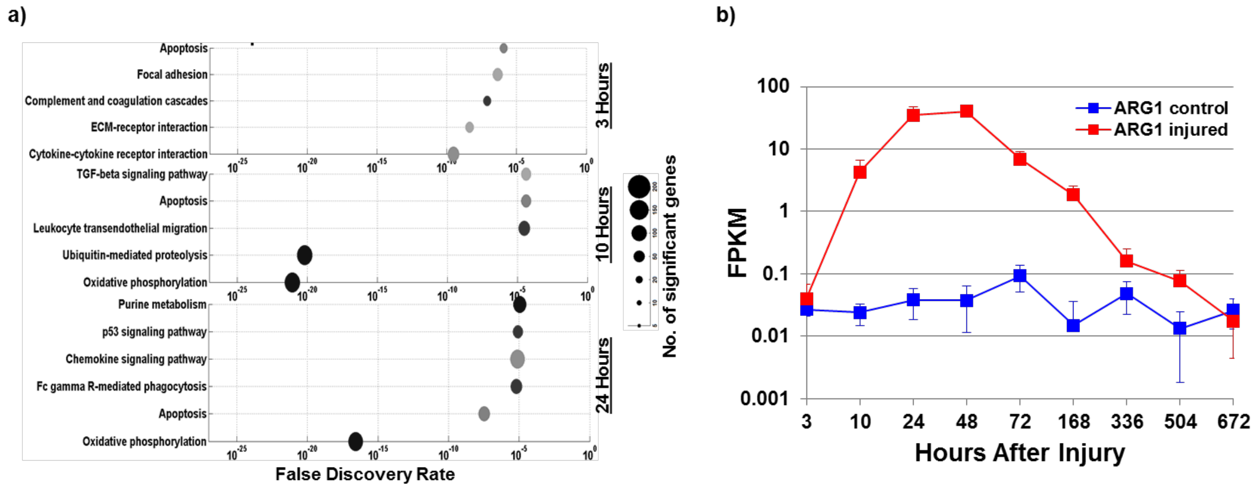

Fig. S4. Gene Sets Activated Early After Injury (3-24 hrs) a) Enriched KEGG pathways from differentially expressed genes for the early time points (3-24 hrs). The size of the circle corresponds to the number of significant genes with each enriched pathway. b) Heatmap of genes associated metabolism expressed in the early time period. c) Gene expression profile of Arginase 1 (Arg1), which is a molecule emitted by type 2 macrophages to balance inflammatory reactive-oxygen species present in the injured tissue. Red-Injured, Blue-Uninjured.

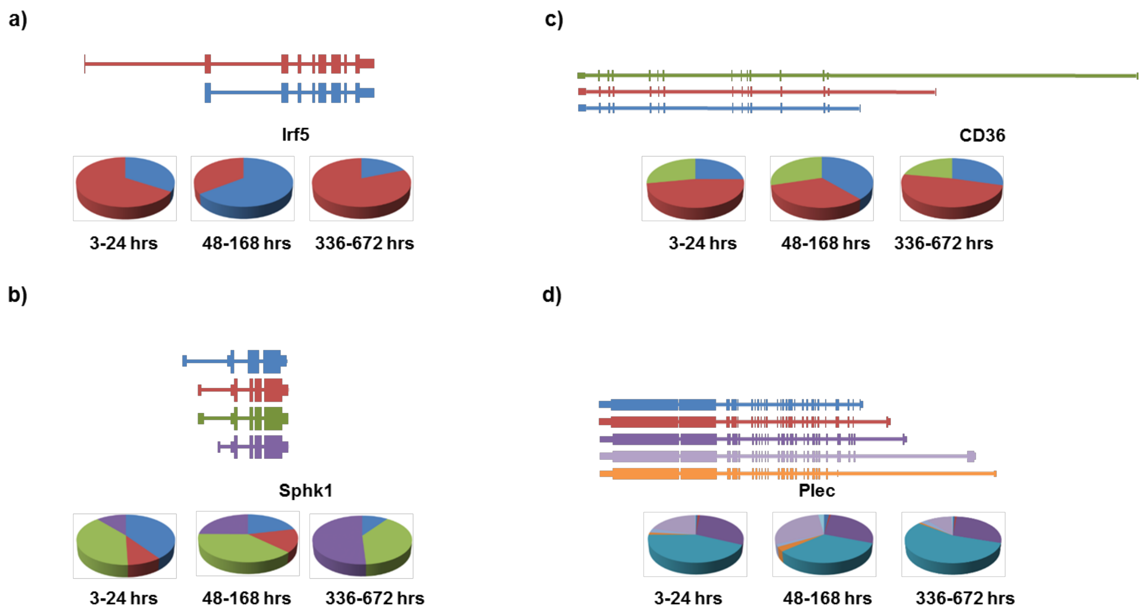

Fig. S5. Examples of alternative splicing in immune network of genes as determined by Cufflinks.

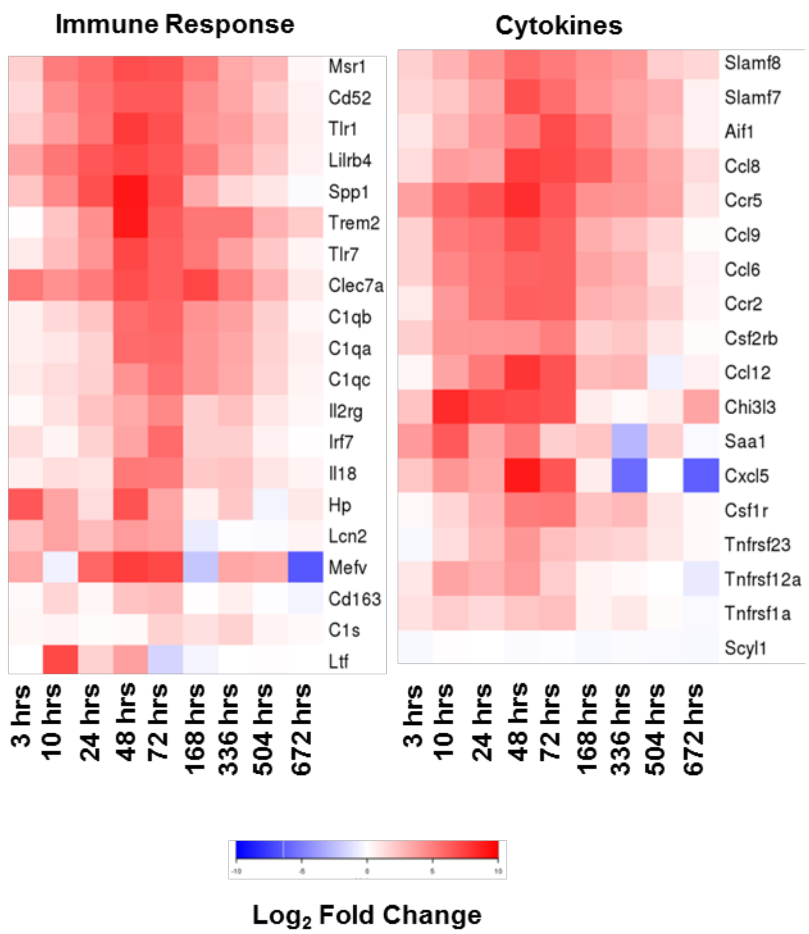

Fig. S6. Genes associated with hematopoietic activation, proliferation and anti-inflammatory cytokines activated in early & middle periods.

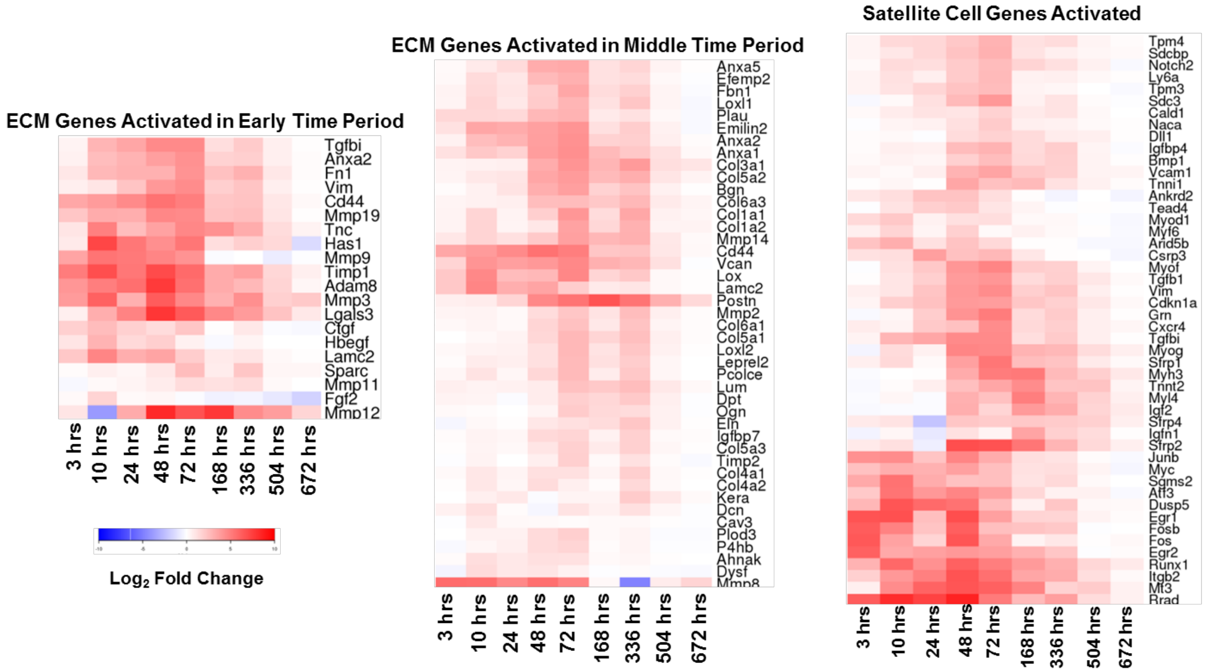

Fig. S7. Gene sets associated with extra-cellular matrix remodeling & satellite cell activation

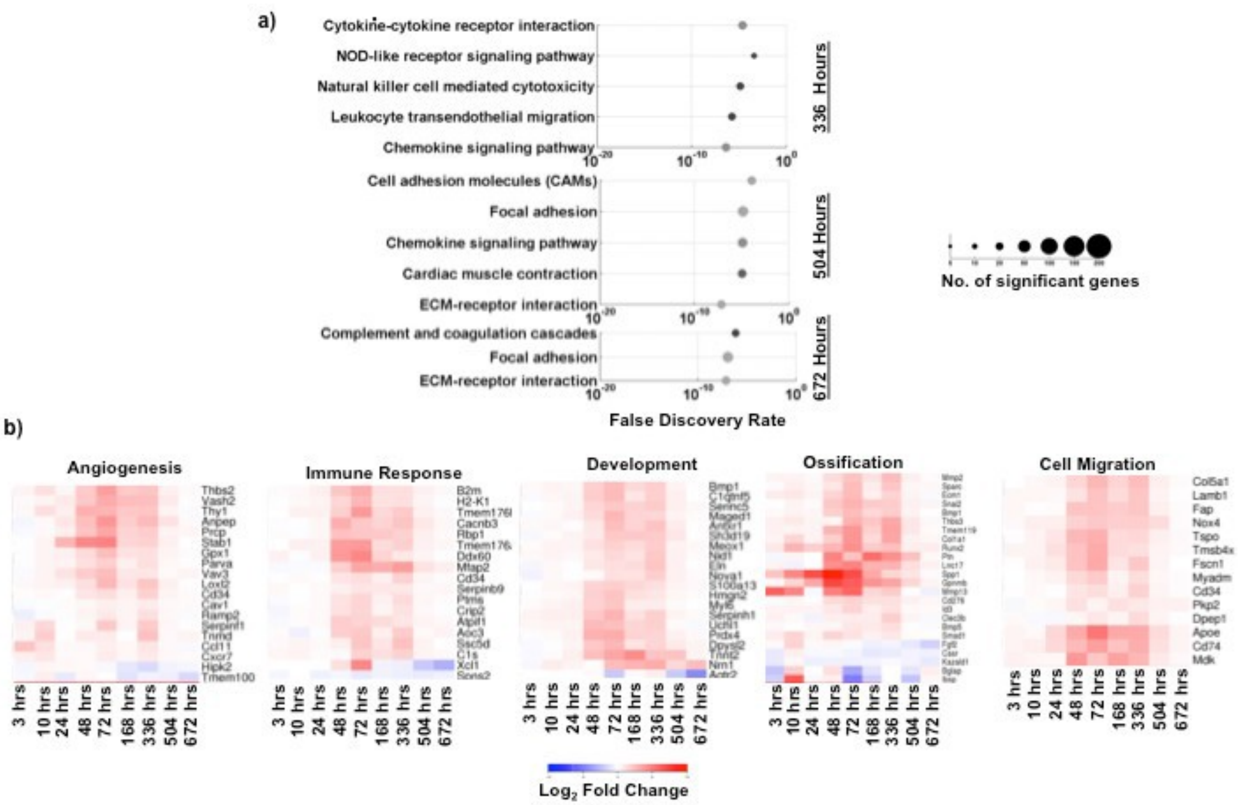

Fig. S8. a) Enriched KEGG pathways from differentially expressed genes for the late time points. The size of the circle corresponds to the number of significant genes with each enriched pathway. b) Heatmaps of gene sets activated in late period categorized by function.

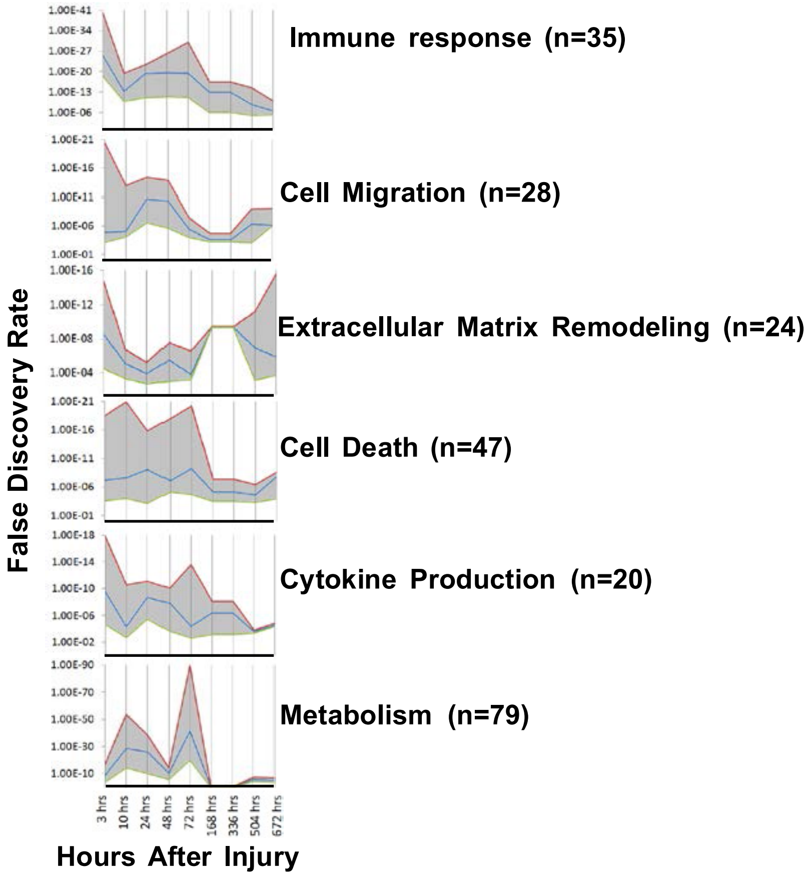

Fig. S9. Area plots of FDR values for Gene Annotation (GO) clusters over time. GO terms were separated into major functional categories, and the IQR was computed for the terms in each group. The grey lines indicate the 25th and 75th percentiles, red lines denote the 25% of gene FDR values, the blue line denotes the median FDR value, and the green line denotes the 75% for FDR.

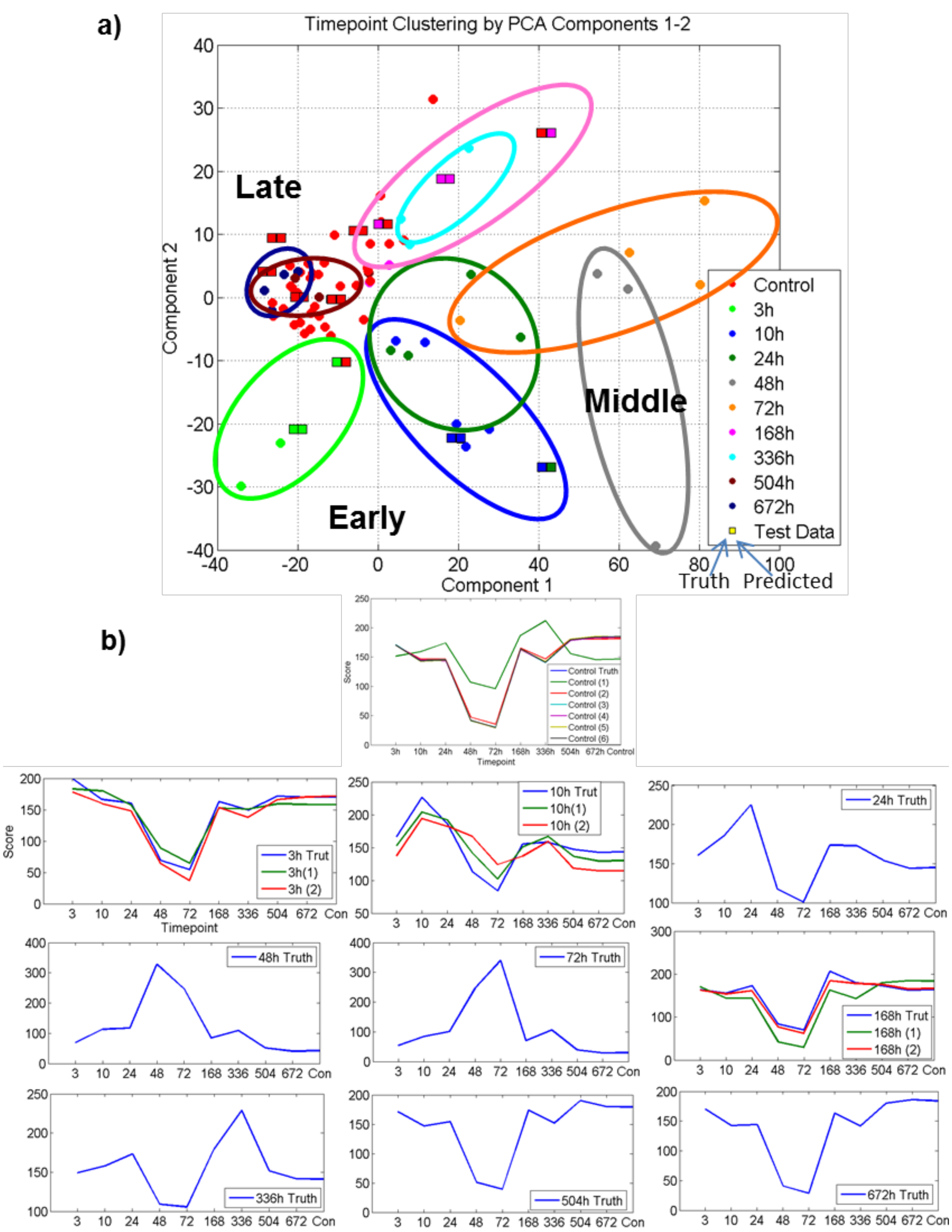

Fig. S10. Pathway-level scoring results for test datasets from PCA and the timepoint signatures methods. a) Principal component analysis. b) Normalized time point signatures method applied to mean pathway expression values. The top panel represents the timepoint signatures obtained for the uninjured control datasets for 6 test samples and a truth training sample. The green curve indicates the control sample that was misclassified by the algorithm at the pathway level. The next panels are derived from time point signatures for the 9 time points (blue lines) as well as 6 test samples (red and green lines).

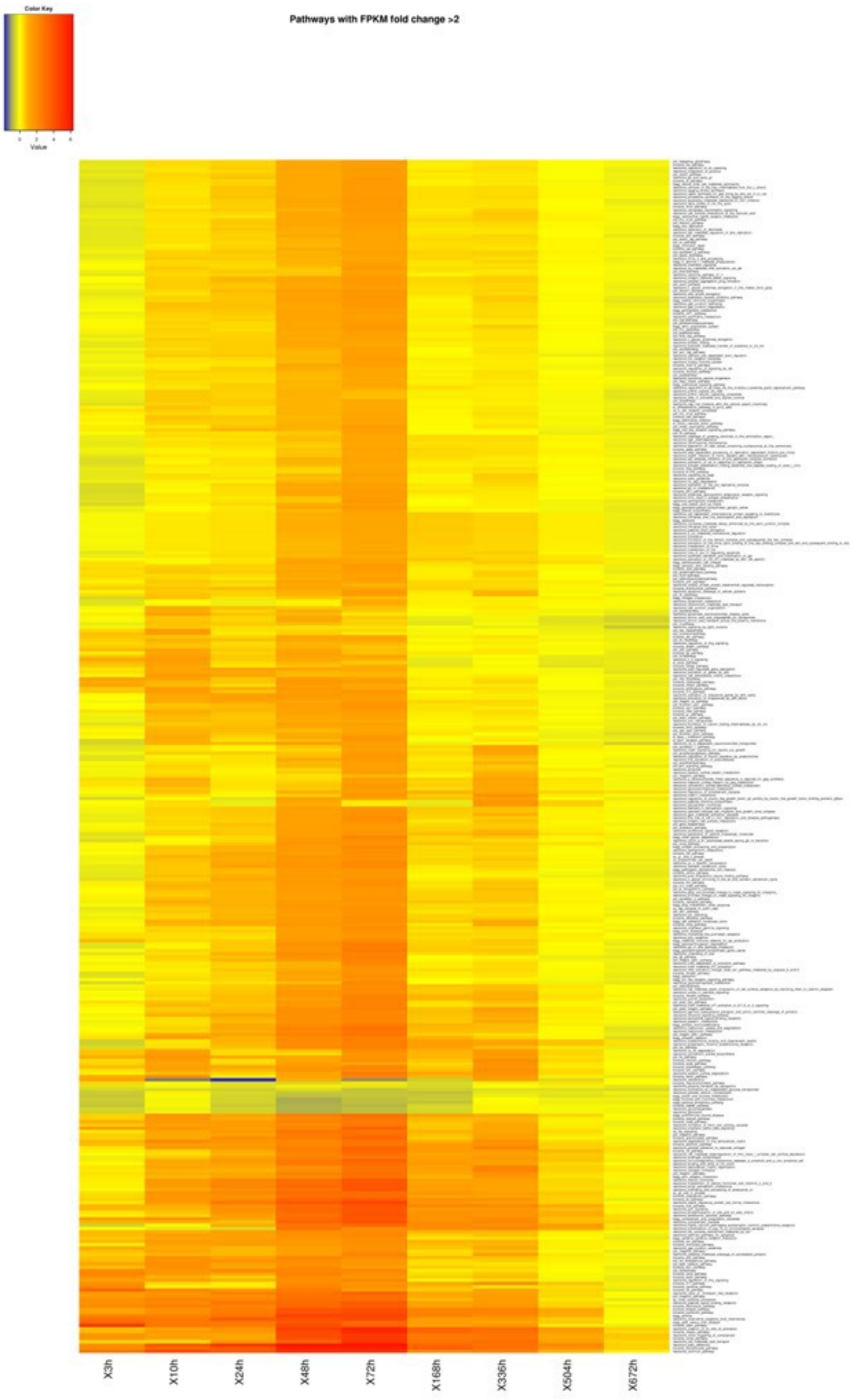

Fig. S11. Differentially expressed gene pathways in injured samples over time.

Table S1.A GO Terms upregulated 3 hours post injury

Annotation Cluster 1 — Enrichment Score: 22.19983472566586

Category	Term	Count	%	PValue	Genes	List Total	Pop Hits	Pop Total	Fold Enrichment	Bonferroni	Benjamini	FDR
GOTERM_BP_FAT	GO:0009611~response to wounding	82	7.56	4.57E-27	GNA13, NOG, MASP1, F13A1, TLR2, GJA1, TLR4, C1QC, TGFB2, CFP, S1PR3, C1RA, MYD88, SLC1A3, CD44, CFH, IL1B, LBP, CFD, TFPI2, NFKBIZ, C4B, SAA3, SERPING1, NLRP3, PLAUR, C1QA, C1QB, SERPINA3N, HIF1A, THBD, DARC, F3, CCR2, PLA2G7, PROS1, LCP1, KDM6B, PTAFR, CXCL1, DCBLD2, CCL3, CCL2, C3, CCR1, CXCL2, CXCL9, ITGB2, PF4, C1S, CCL7, TRF, CCL24, SLC11A1, TNFRSF1A, TNFRSF1B, PROCR, MAP3K1, ENTPD1, THBS1, FN1, PTPN6, SELP, KLF6, BMP2, PLEK, CFB, TGFBR2, SPHK1, TLR13, SMAD1, ANXA5, STAT3, FCGR3, CCL11, NOTCH2, NUPR1, HBEGF, ID3, CLEC7A, BMPR1B, CD14	823	347	13588	3.90	1.38E-23	0.00	8.23E-24
GOTERM_BP_FAT	GO:0006952~defense response	85	7.84	2.72E-21	MASP1, TLR2, H2-D1, TLR4, C1QC, CFP, S1PR3, C1RA, TMEM173, MYD88, CD44, IL1RAP, CFH, IL1B, LBP, CFD, MX2, NFKBIZ, BCL10, C5AR1, C4B, RELA, SAA3, IFI47, SERPING1, CLIC1, NLRP3, C1QA, C1QB, SERPINA3N, HIF1A, DARC, CCR2, PLA2G7, H2-AA, KDM6B, PTAFR, CXCL1, IFIH1, IL1R1, CCL3, CCL2, C3, CCR1, CXCL2, CXCL9, RSAD2, ITGB2, C1S, CD74, CCL7, TRF, CCL24, SLC11A1, TNFRSF1A, TNFRSF1B, TAP1, FCER1G, BCL3, THBS1, FN1, PTPRC, PTPN6, SELP, BMP2, LYZ2, IRGM1, CFB, IL1RL1, IL1RL2, SPHK1, TLR13, SMAD1, COTL1, STAT3, FCGR3, CCL11, DDX58, FCGR2B, NUPR1, IRF8, APAF1, CLEC7A, BMPR1B, CD14	823	448	13588	3.13	8.20E-18	0.00	4.90E-18
GOTERM_BP_FAT	GO:0006954~inflammatory response	57	5.26	2.02E-20	MASP1, TLR2, TLR4, C1QC, CFP, S1PR3, C1RA, MYD88, CD44, CFH, IL1B, LBP, CFD, NFKBIZ, C4B, SAA3, SERPING1, NLRP3, C1QA, C1QB, SERPINA3N, HIF1A, DARC, CCR2, PLA2G7, PTAFR, KDM6B, CXCL1, CCL3, CCL2, C3, CCR1, CXCL2, CXCL9, ITGB2, C1S, CCL7, TRF, CCL24, TNFRSF1A, SLC11A1, TNFRSF1B, THBS1, FN1, SELP, BMP2, CFB, SPHK1, TLR13, SMAD1, STAT3, FCGR3, CCL11, NUPR1, CLEC7A, BMPR1B, CD14	823	225	13588	4.18	6.11E-17	0.00	3.65E-17

Annotation Cluster 2 — Enrichment Score: 11.413810308144937

Category	Term	Count	%	PValue	Genes	List Total	Pop Hits	Pop Total	Fold Enrichment	Bonferroni	Benjamini	FDR
GOTERM_MF_FAT	GO:0001871~pattern binding	35	3.23	5.36E-14	ENPP2, POSTN, PF4, FSTL1, DCN, CCL7, ITGAM, ABI3BP, SMOC2, CD44, APOE, CTGF, CRISPLD2, COMP, CFH, PTX3, THBS1, THBS2, CYR61, FN1, BMP4, PTPRC, TNXB, FBN1, CCDC80, CHODL, TNFAIP6, ADAMTS8, EGFLAM, LAYN, HBEGF, LAMC2, ADAMTS1, VCAN, CLEC7A	788	128	13288	4.61	4.59E-11	0.00	8.30E-11
GOTERM_MF_FAT	GO:0030247~polysaccharide binding	35	3.23	5.36E-14	ENPP2, POSTN, PF4, FSTL1, DCN, CCL7, ITGAM, ABI3BP, SMOC2, CD44, APOE, CTGF, CRISPLD2, COMP, CFH, PTX3, THBS1, THBS2, CYR61, FN1, BMP4, PTPRC, TNXB, FBN1, CCDC80, CHODL, TNFAIP6, ADAMTS8, EGFLAM, LAYN, HBEGF, LAMC2, ADAMTS1, VCAN, CLEC7A	788	128	13288	4.61	4.59E-11	0.00	8.30E-11
GOTERM_MF_FAT	GO:0005539~glycosaminoglycan binding	31	2.86	2.16E-12	POSTN, PF4, FSTL1, DCN, CCL7, ITGAM, ABI3BP, SMOC2, CD44, APOE, CTGF, CRISPLD2, COMP, CFH, THBS1, THBS2, CYR61, FN1, BMP4, PTPRC, TNXB, CCDC80, CHODL, TNFAIP6, ADAMTS8, EGFLAM, LAYN, HBEGF, LAMC2, ADAMTS1, VCAN	788	114	13288	4.59	1.85E-09	0.00	3.34E-09
GOTERM_MF_FAT	GO:0008201~heparin binding	24	2.21	2.64E-10	BMP4, PTPRC, TNXB, CCDC80, POSTN, PF4, FSTL1, CCL7, ITGAM, ABI3BP, SMOC2, ADAMTS8, APOE, CTGF, CRISPLD2, COMP, CFH, HBEGF, ADAMTS1, LAMC2, THBS1, THBS2, CYR61, FN1	788	83	13288	4.88	2.26E-07	0.00	4.08E-07
GOTERM_MF_FAT	GO:0030246~carbohydrate binding	50	4.61	5.22E-10	CD209A, POSTN, FSTL1, CD93, CD44, CTGF, APOE, CFH, CLEC4D, CYR61, CHODL, CLEC4N, TNFAIP6, ADAMTS8, EGFLAM, CD33, VCAN, ADAMTS1, LAMC2, ENPP2, PF4, DCN, CLEC10A, CCL7, ABI3BP, ITGAM, SMOC2, CRISPLD2, COMP, PTX3, THBS1, THBS2, SELPLG, FN1, BMP4, MRC1, SELP, PTPRC, TNXB, LGALS3, SELL, FBN1, CCDC80, LGALS9, CLEC3B, LAYN, GFPT2, HBEGF, CLEC7A, CD302	788	317	13288	2.66	4.46E-07	0.00	8.08E-07

Annotation Cluster 3 — Enrichment Score: 10.402160676764138

Category	Term	Count	%	PValue	Genes	List Total	Pop Hits	Pop Total	Fold Enrichment	Bonferroni	Benjamini	FDR
GOTERM_BP_FAT	GO:0006935~chemotaxis	35	3.23	5.42E-16	NRP2, CCL3, CCL2, S100A8, ENPP2, CCR1, SLC37A4, S100A9, CXCL2, FPR1, CCL9, CXCR2, PF4, ITGB2, FPR2, CCL7, ITGAM, CCL6, TGFB2, CCL24, RAC2, SEMA3F, CSF3R, IL1B, FCER1G, LBP, CYR61, C5AR1, FCGR3, CCL11, CORO1A, CXCL16, ECSCR, CMTM6, PTAFR	823	109	13588	5.30	1.67E-12	0.00	9.99E-13
GOTERM_BP_FAT	GO:0042330~taxis	35	3.23	5.42E-16	NRP2, CCL3, CCL2, S100A8, ENPP2, CCR1, SLC37A4, S100A9, CXCL2, FPR1, CCL9, CXCR2, PF4, ITGB2, FPR2, CCL7, ITGAM, CCL6, TGFB2, CCL24, RAC2, SEMA3F, CSF3R, IL1B, FCER1G, LBP, CYR61, C5AR1, FCGR3, CCL11, CORO1A, CXCL16, ECSCR, CMTM6, PTAFR	823	109	13588	5.30	1.67E-12	0.00	9.99E-13
GOTERM_BP_FAT	GO:0030595~leukocyte chemotaxis	15	1.38	7.56E-11	C5AR1, CCR1, CXCL2, S100A9, SLC37A4, ITGB2, PF4, ITGAM, TGFB2, FCGR3, CORO1A, FCER1G, IL1B, CSF3R, LBP	823	27	13588	9.17	2.28E-07	0.00	1.36E-07
GOTERM_BP_FAT	GO:0060326~cell chemotaxis	15	1.38	7.56E-11	C5AR1, CCR1, CXCL2, S100A9, SLC37A4, ITGB2, PF4, ITGAM, TGFB2, FCGR3, CORO1A, FCER1G, IL1B, CSF3R, LBP	823	27	13588	9.17	2.28E-07	0.00	1.36E-07
GOTERM_BP_FAT	GO:0007626~locomotory behavior	42	3.87	1.12E-09	NRP2, CCL3, CCL2, S100A8, ENPP2, CCR1, SLC37A4, S100A9, CXCL2, FPR1, CCL9, CXCR2, PF4, ITGB2, FPR2, ITGAM, CCL7, CCL6, TGFB2, CCL24, RAC2, PDE1B, SEMA3F, CSF3R, IL1B, FCER1G, LBP, CYR61, KCNMA1, C5AR1, NR4A2, NPY1R, FCGR3, CCL11, CORO1A, EPS8, CXCL16, ECSCR, CSTB, RASD2, CMTM6, PTAFR	823	239	13588	2.90	3.37E-06	0.00	2.01E-06
GOTERM_BP_FAT	GO:0050900~leukocyte migration	17	1.57	1.58E-09	SELP, C5AR1, CCR1, CXCL2, S100A9, SLC37A4, ITGB2, PF4, ITGAM, TGFB2, FCGR3, CORO1A, FCER1G, IL1B, CSF3R, LBP, SELPLG	823	43	13588	6.53	4.76E-06	0.00	2.84E-06
GOTERM_BP_FAT	GO:0007610~behavior	56	5.17	1.26E-08	NRP2, S100A8, S100A9, CXCR2, TGFB2, PRKAR2B, BDNF, SLC1A3, SEMA3F, CSF3R, IL1B, LBP, CYR61, KCNMA1, EGR1, C5AR1, EGR2, OSM, CSTB, RASD2, PTAFR, CCL3, CCL2, ENPP2, CCR1, SLC37A4, CXCL2, FPR1, CCL9, PF4, ITGB2, FPR2, CCL7, ITGAM, CCL6, CCL24, RAC2, PDE1B, AGT, FCER1G, RUNX1, NR4A2, ITGA3, NPY1R, NR4A3, STAT3, FCGR3, CCL11, CORO1A, EPS8, ITGA5, CXCL16, ECSCR, NTRK2, AVPR1A, CMTM6	823	405	13588	2.28	3.81E-05	0.00	2.28E-05
GOTERM_BP_FAT	GO:0030593~neutrophil chemotaxis	10	0.92	1.63E-07	C5AR1, CXCL2, SLC37A4, FCER1G, IL1B, CSF3R, ITGB2, ITGAM, FCGR3, TGFB2	823	17	13588	9.71	4.91E-04	0.00	2.93E-04

Annotation Cluster 4 — Enrichment Score: 10.278554654758812

Category	Term	Count	%	PValue	Genes	List Total	Pop Hits	Pop Total	Fold Enrichment	Bonferroni	Benjamini	FDR
GOTERM_BP_FAT	GO:0001944~vasculature development	50	4.61	1.53E-13	GNA13, PGF, GJA1, FOXO1, MMP2, TGFB2, SHB, CTGF, CXCR4, APOE, HMOX1, CASP8, IL1B, LOX, NR2F2, ANGPT2, CYR61, PTPRJ, SOCS3, PDPN, MMP19, MYH9, MMP14, JUNB, HIF1A, NUS1, ZFP697, COL1A1, TNFAIP2, TNFRSF12A, TIPARP, COL3A1, ZFP36L1, ALDH1A2, AGT, ZC3H12A, COL18A1, BMP4, KLF5, TGFBR1, SPHK1, TGFBR2, ITGA4, FZD5, EPHA2, ANXA2, LAMA4, ECSCR, NTRK2, HBEGF	823	250	13588	3.30	4.62E-10	0.00	2.76E-10
GOTERM_BP_FAT	GO:0001568~blood vessel development	49	4.52	2.36E-13	GNA13, PGF, GJA1, FOXO1, MMP2, TGFB2, SHB, CTGF, CXCR4, APOE, HMOX1, CASP8, IL1B, LOX, NR2F2, ANGPT2, CYR61, PTPRJ, SOCS3, MMP19, MMP14, MYH9, JUNB, HIF1A, NUS1, ZFP697, COL1A1, TNFAIP2, TNFRSF12A, TIPARP, COL3A1, ZFP36L1, ALDH1A2, AGT, ZC3H12A, COL18A1, BMP4, KLF5, TGFBR1, SPHK1, TGFBR2, ITGA4, FZD5, EPHA2, ANXA2, LAMA4, ECSCR, NTRK2, HBEGF	823	244	13588	3.32	7.12E-10	0.00	4.25E-10
GOTERM_BP_FAT	GO:0048514~blood vessel morphogenesis	38	3.51	6.29E-10	GNA13, PGF, TNFRSF12A, TIPARP, GJA1, TGFB2, ZFP36L1, SHB, APOE, CTGF, CXCR4, HMOX1, CASP8, IL1B, ZC3H12A, NR2F2, ANGPT2, CYR61, COL18A1, PTPRJ, BMP4, KLF5, TGFBR1, MMP19, TGFBR2, ITGA4, MYH9, FZD5, MMP14, JUNB, EPHA2, ANXA2, HIF1A, NUS1, NTRK2, ECSCR, HBEGF, TNFAIP2	823	198	13588	3.17	1.90E-06	0.00	1.13E-06
GOTERM_BP_FAT	GO:0001525~angiogenesis	26	2.40	3.38E-07	GNA13, TNFRSF12A, PGF, SHB, CXCR4, CTGF, HMOX1, CASP8, IL1B, ZC3H12A, ANGPT2, CYR61, COL18A1, KLF5, BMP4, TGFBR2, MMP19, FZD5, MMP14, MYH9, ANXA2, HIF1A, NUS1, ECSCR, HBEGF, TNFAIP2	823	133	13588	3.23	0.001019867	0.00	6.09E-04

Annotation Cluster 5 — Enrichment Score: 9.121149951246146

Category	Term	Count	%	PValue	Genes	List Total	Pop Hits	Pop Total	Fold Enrichment	Bonferroni	Benjamini	FDR
GOTERM_BP_FAT	GO:0016477~cell migration	45	4.15	2.87E-11	PVR, NRP2, TNFRSF12A, CCR1, SLC37A4, S100A9, CXCL2, GJA1, ZEB2, PF4, ITGB2, PRKG1, ITGAM, TGFB2, CTGF, CXCR4, SEMA3F, CSF3R, IL1B, FCER1G, CAP1, LBP, NR2F2, SELPLG, DCLK1, FMNL1, PLAT, EGFR, SELP, C5AR1, ARID5B, TGFBR1, NR4A2, ITGA3, ITGA4, MYH9, MMP14, FCGR3, CORO1A, HIF1A, NAV1, FYN, CFL1, HBEGF, PLAU	823	240	13588	3.10	8.65E-08	0.00	5.16E-08
GOTERM_BP_FAT	GO:0006928~cell motion	55	5.07	9.67E-10	PVR, NRP2, S100A9, GJA1, ZEB2, PRKG1, TGFB2, BDNF, CTGF, CXCR4, SEMA3F, CSF3R, IL1B, LBP, CAP1, NR2F2, BOC, FMNL1, EGFR, C5AR1, EGR2, PDPN, ARID5B, MYH9, MMP14, SIRPA, HIF1A, NAV1, CFL1, TNFRSF12A, CCR1, SLC37A4, CXCL2, PF4, ITGB2, CDH4, ITGAM, CD9, DNAJA1, FCER1G, B3GNT2, SELPLG, DCLK1, PLAT, SELP, TGFBR1, NR4A2, ITGA3, ITGA4, FCGR3, CORO1A, FYN, HBEGF, BMPR1B, PLAU	823	367	13588	2.47	2.92E-06	0.00	1.74E-06
GOTERM_BP_FAT	GO:0050900~leukocyte migration	17	1.57	1.58E-09	SELP, C5AR1, CCR1, CXCL2, S100A9, SLC37A4, ITGB2, PF4, ITGAM, TGFB2, FCGR3, CORO1A, FCER1G, IL1B, CSF3R, LBP, SELPLG	823	43	13588	6.53	4.76E-06	0.00	2.84E-06
GOTERM_BP_FAT	GO:0051674~localization of cell	46	4.24	2.38E-09	PVR, NRP2, TNFRSF12A, CCR1, SLC37A4, S100A9, CXCL2, GJA1, ZEB2, PF4, ITGB2, PRKG1, ITGAM, TGFB2, CTGF, CXCR4, SEMA3F, DNAJA1, CSF3R, IL1B, FCER1G, CAP1, LBP, NR2F2, SELPLG, DCLK1, FMNL1, PLAT, EGFR, SELP, C5AR1, ARID5B, TGFBR1, NR4A2, ITGA3, ITGA4, MYH9, MMP14, FCGR3, CORO1A, HIF1A, NAV1, FYN, CFL1, HBEGF, PLAU	823	284	13588	2.67	7.18E-06	0.00	4.29E-06
GOTERM_BP_FAT	GO:0048870~cell motility	46	4.24	2.38E-09	PVR, NRP2, TNFRSF12A, CCR1, SLC37A4, S100A9, CXCL2, GJA1, ZEB2, PF4, ITGB2, PRKG1, ITGAM, TGFB2, CTGF, CXCR4, SEMA3F, DNAJA1, CSF3R, IL1B, FCER1G, CAP1, LBP, NR2F2, SELPLG, DCLK1, FMNL1, PLAT, EGFR, SELP, C5AR1, ARID5B, TGFBR1, NR4A2, ITGA3, ITGA4, MYH9, MMP14, FCGR3, CORO1A, HIF1A, NAV1, FYN, CFL1, HBEGF, PLAU	823	284	13588	2.67	7.18E-06	0.00	4.29E-06

Annotation Cluster 6 — Enrichment Score: 8.28576005027683

Category	Term	Count	%	PValue	Genes	List Total	Pop Hits	Pop Total	Fold Enrichment	Bonferroni	Benjamini	FDR
GOTERM_BP_FAT	GO:0002684~positive regulation of immune system process	46	4.24	2.60E-14	ITGAL, ADORA2B, MASP1, C3, IL4RA, TLR2, NFKBIA, TLR4, C1S, C1QC, PNP, CD74, ADA, TGFB2, CFP, IRAK3, C1RA, SLC11A1, MYD88, CFH, FCER1G, INPP5D, LBP, CFD, THBS1, PTPRC, BCL10, PTPN6, SELP, ICOSL, HSP90AA1, LYN, C4B, CFB, RELA, TGFBR2, SERPING1, NFAM1, FCGR3, C1QA, C1QB, CD83, CORO1A, CDKN1A, H2-AA, CLEC7A	823	206	13588	3.69	7.84E-11	0.00	4.68E-11
GOTERM_BP_FAT	GO:0002252~immune effector process	35	3.23	6.04E-14	MASP1, C3, RSAD2, C1S, C1QC, CD74, CFP, C1RA, SLC11A1, MYD88, CFH, FCER1G, BCL3, FAS, INPP5D, LBP, CFD, PTX3, PTPRC, PTPN6, BCL10, ICAM1, ICOSL, C4B, CFB, SWAP70, SERPING1, NLRP3, FCGR3, C1QA, C1QB, FCGR2B, LILRB3, IRF7, H2-AA	823	126	13588	4.59	1.82E-10	0.00	1.09E-10
GOTERM_BP_FAT	GO:0045087~innate immune response	31	2.86	6.16E-13	IFIH1, IL1R1, MASP1, C3, TLR2, TLR4, C1S, C1QC, CFP, C1RA, SLC11A1, TMEM173, MYD88, IL1RAP, CFH, LBP, CFD, MX2, PTPN6, BCL10, IRGM1, C4B, IL1RL1, CFB, IL1RL2, TLR13, SERPING1, C1QA, DDX58, C1QB, CLEC7A	823	107	13588	4.78	1.86E-09	0.00	1.11E-09
GOTERM_BP_FAT	GO:0002449~lymphocyte mediated immunity	26	2.40	9.98E-13	C3, C1S, C1QC, CD74, C1RA, SLC11A1, MYD88, FCER1G, BCL3, INPP5D, FAS, PTPN6, BCL10, ICAM1, ICOSL, SWAP70, CFB, C4B, SERPING1, FCGR3, C1QA, C1QB, FCGR2B, LILRB3, IRF7, H2-AA	823	76	13588	5.65	3.01E-09	0.00	1.80E-09
GOTERM_BP_FAT	GO:0019724~B cell mediated immunity	23	2.12	1.21E-11	BCL10, ICOSL, C3, C4B, CFB, SWAP70, SERPING1, C1S, C1QC, CD74, FCGR3, C1QA, C1QB, C1RA, MYD88, FCGR2B, LILRB3, IRF7, FCER1G, BCL3, H2-AA, FAS, INPP5D	823	65	13588	5.84	3.66E-08	0.00	2.18E-08
GOTERM_BP_FAT	GO:0002250~adaptive immune response	26	2.40	1.23E-11	C3, C1S, C1QC, CD74, C1RA, SLC11A1, MYD88, FCER1G, BCL3, INPP5D, FAS, BCL10, ICAM1, ICOSL, SWAP70, CFB, C4B, SERPING1, FCGR3, C1QA, C1QB, FCGR2B, LILRB3, IRF7, GADD45G, H2-AA	823	84	13588	5.11	3.72E-08	0.00	2.22E-08
GOTERM_BP_FAT	GO:0002460~adaptive immune response based on somatic recombination of immune receptors built fr	26	2.40	1.23E-11	C3, C1S, C1QC, CD74, C1RA, SLC11A1, MYD88, FCER1G, BCL3, INPP5D, FAS, BCL10, ICAM1, ICOSL, SWAP70, CFB, C4B, SERPING1, FCGR3, C1QA, C1QB, FCGR2B, LILRB3, IRF7, GADD45G, H2-AA	823	84	13588	5.11	3.72E-08	0.00	2.22E-08
GOTERM_BP_FAT	GO:0002443~leukocyte mediated immunity	26	2.40	5.02E-11	C3, C1S, C1QC, CD74, C1RA, SLC11A1, MYD88, FCER1G, BCL3, INPP5D, FAS, PTPN6, BCL10, ICAM1, ICOSL, SWAP70, CFB, C4B, SERPING1, FCGR3, C1QA, C1QB, FCGR2B, LILRB3, IRF7, H2-AA	823	89	13588	4.82	1.52E-07	0.00	9.05E-08
GOTERM_BP_FAT	GO:0050778~positive regulation of immune response	32	2.95	9.12E-11	MASP1, C3, TLR2, NFKBIA, TLR4, C1S, C1QC, PNP, ADA, TGFB2, CFP, C1RA, SLC11A1, MYD88, CFH, FCER1G, LBP, CFD, PTPN6, BCL10, PTPRC, LYN, C4B, CFB, RELA, SERPING1, NFAM1, FCGR3, C1QA, C1QB, H2-AA, CLEC7A	823	136	13588	3.88	2.75E-07	0.00	1.64E-07
GOTERM_BP_FAT	GO:0002253~activation of immune response	25	2.31	1.42E-10	MASP1, C3, TLR2, NFKBIA, TLR4, C1S, C1QC, CFP, C1RA, MYD88, CFH, FCER1G, CFD, BCL10, PTPN6, PTPRC, LYN, CFB, C4B, RELA, SERPING1, NFAM1, C1QA, C1QB, CLEC7A	823	86	13588	4.80	4.29E-07	0.00	2.56E-07
GOTERM_BP_FAT	GO:0016064~immunoglobulin mediated immune response	21	1.94	3.88E-10	BCL10, ICOSL, C3, C4B, CFB, SWAP70, SERPING1, C1S, C1QC, CD74, FCGR3, C1QA, C1QB, C1RA, MYD88, FCGR2B, IRF7, FCER1G, BCL3, H2-AA, INPP5D	823	63	13588	5.50	1.17E-06	0.00	6.99E-07
GOTERM_BP_FAT	GO:0048584~positive regulation of response to stimulus	37	3.41	3.91E-10	ADORA2B, MASP1, C3, TLR2, NFKBIA, TLR4, C1S, C1QC, PNP, ADA, TGFB2, CFP, TNFRSF1A, C1RA, SLC11A1, MYD88, CFH, FCER1G, IL1B, LBP, THBS1, CFD, PTPRC, PTPN6, BCL10, LYN, C4B, CFB, RELA, SERPING1, NFAM1, FCGR3, C1QA, C1QB, H2-AA, FABP4, CLEC7A	823	186	13588	3.28	1.18E-06	0.00	7.04E-07
GOTERM_BP_FAT	GO:0002526~acute inflammatory response	23	2.12	1.47E-09	MASP1, C3, C4B, CFB, SAA3, TLR4, SERPING1, C1S, C1QC, TRF, STAT3, FCGR3, CFP, C1QA, C1QB, C1RA, SERPINA3N, NUPR1, CFH, IL1B, LBP, CFD, FN1	823	81	13588	4.69	4.43E-06	0.00	2.65E-06
GOTERM_BP_FAT	GO:0002541~activation of plasma proteins involved in acute inflammatory response	13	1.20	7.19E-07	MASP1, CFB, C4B, C3, SERPING1, C1S, C1QC, C1QA, CFP, C1RA, C1QB, CFH, CFD	823	36	13588	5.96	0.002167186	0.00	0.001295678
GOTERM_BP_FAT	GO:0006956~complement activation	13	1.20	7.19E-07	MASP1, CFB, C4B, C3, SERPING1, C1S, C1QC, C1QA, CFP, C1RA, C1QB, CFH, CFD	823	36	13588	5.96	0.002167186	0.00	0.001295678
GOTERM_BP_FAT	GO:0006959~humoral immune response	15	1.38	2.64E-06	MASP1, CFB, C4B, C3, SERPING1, C1S, C1QC, C1QA, CFP, C1QB, C1RA, CCR2, CFH, BCL3, CFD	823	54	13588	4.59	0.007926493	0.00	0.004752592
GOTERM_BP_FAT	GO:0051605~protein maturation by peptide bond cleavage	15	1.38	2.66E-05	MASP1, CFB, C4B, C3, SERPING1, C1S, MMP14, C1QC, C1QA, CFP, C1QB, C1RA, CFH, CFD, SRGN	823	65	13588	3.81	0.077170232	0.00	0.047951365

Annotation Cluster 7 — Enrichment Score: 8.066260037968595

Category	Term	Count	%	PValue	Genes	List Total	Pop Hits	Pop Total	Fold Enrichment	Bonferroni	Benjamini	FDR

Category	Term	Count	%	PValue	Genes	List Total	Pop Hits	Pop Total	Fold Enrichment	Bonferroni	Benjamini	FDR
GOTERM_BP_FAT	GO:0007155~cell adhesion	76	7.01	5.20E-11	PVR, NRP2, CADM3, ATP1B2, CLDN5, POSTN, DDR2, TGFB2, CGREF1, CD44, CD93, CTGF, TGFBI, CSF3R, ZYX, ADAM8, COL11A2, BOC, CYR61, ICAM1, PDPN, PCDH7, MYH9, SIRPA, BCL2L11, CD84, HES1, TNFAIP6, CD33, VCAN, LAMC2, DCBLD2, ITGAL, TNFRSF12A, TNC, NEDD9, ITGB2, SOX9, CDH4, ITGAM, ITGBL1, VCAM1, CD9, PVRL3, AGT, COMP, MYBPH, COL6A2, COL6A1, THBS1, SELPLG, THBS2, THBS3, FN1, THBS4, DPT, SPP1, COL18A1, PTPRC, SELP, SVEP1, TNXB, PLEK, SELL, PCDH12, NID1, ITGA3, ITGA4, EMILIN2, COL16A1, LAMA4, COL14A1, SNED1, ITGA5, CLEC7A, BMPR1B	823	561	13588	2.24	1.57E-07	0.00	9.36E-08
GOTERM_BP_FAT	GO:0022610~biological adhesion	76	7.01	5.65E-11	PVR, NRP2, CADM3, ATP1B2, CLDN5, POSTN, DDR2, TGFB2, CGREF1, CD44, CD93, CTGF, TGFBI, CSF3R, ZYX, ADAM8, COL11A2, BOC, CYR61, ICAM1, PDPN, PCDH7, MYH9, SIRPA, BCL2L11, CD84, HES1, TNFAIP6, CD33, VCAN, LAMC2, DCBLD2, ITGAL, TNFRSF12A, TNC, NEDD9, ITGB2, SOX9, CDH4, ITGAM, ITGBL1, VCAM1, CD9, PVRL3, AGT, COMP, MYBPH, COL6A2, COL6A1, THBS1, SELPLG, THBS2, THBS3, FN1, THBS4, DPT, SPP1, COL18A1, PTPRC, SELP, SVEP1, TNXB, PLEK, SELL, PCDH12, NID1, ITGA3, ITGA4, EMILIN2, COL16A1, LAMA4, COL14A1, SNED1, ITGA5, CLEC7A, BMPR1B	823	562	13588	2.23	1.70E-07	0.00	1.02E-07
Annotation Cluster 8	Enrichment Score: 5.794336180757243											
Category	Term	Count	%	PValue	Genes	List Total	Pop Hits	Pop Total	Fold Enrichment	Bonferroni	Benjamini	FDR
GOTERM_MF_FAT	GO:0008009~chemokine activity	14	1.29	1.36E-07	CXCL1, CCL3, CCL2, CXCL2, CXCL9, CCL9, PF4, CCL7, CCL6, CCL24, CCL11, CXCL14, PPBP, CXCL16	788	38	13288	6.21	1.16E-04	0.00	2.10E-04
GOTERM_MF_FAT	GO:0042379~chemokine receptor binding	14	1.29	1.93E-07	CXCL1, CCL3, CCL2, CXCL2, CXCL9, CCL9, PF4, CCL7, CCL6, CCL24, CCL11, CXCL14, PPBP, CXCL16	788	39	13288	6.05	1.65E-04	0.00	2.99E-04
Annotation Cluster 9	Enrichment Score: 5.746125904859628											
Category	Term	Count	%	PValue	Genes	List Total	Pop Hits	Pop Total	Fold Enrichment	Bonferroni	Benjamini	FDR
GOTERM_BP_FAT	GO:0006915~apoptosis	56	5.17	1.28E-06	NUAK2, NFKB1, CXCR2, TGFB2, SHB, TMEM173, CASP4, BAG3, CASP8, FAS, CASP1, MYC, BCL10, LTBR, NLRP3, BCL2L11, DDIT4, OSM, TNFRSF10B, RIPK1, GADD45G, RIPK3, TNFAIP3, EIF2AK2, GADD45B, PPP1R15A, PEG3, MCL1, LITAF, TNFRSF12A, PML, STK17B, ARF6, TNFRSF1A, PRUNE2, IRAK3, PEG10, COMP, MAP3K1, SHISA5, ZC3H12A, INPP5D, SRGN, PHLDA1, CFLAR, ACTC1, BIRC3, NFKBIL1, CIDEC, RASSF5, CSRNP1, CASP12, SULF1, APAF1, DRAM1, PDCD7	823	465	13588	1.99	0.003849547	0.00	0.002303428
GOTERM_BP_FAT	GO:0016265~death	60	5.54	1.93E-06	NUAK2, NFKB1, CXCR2, TGFB2, SHB, TMEM173, CASP4, BAG3, CASP8, FAS, CASP1, MYC, BCL10, LTBR, NLRP3, BCL2L11, DDIT4, OSM, TNFRSF10B, RIPK1, GADD45G, RIPK3, TNFAIP3, EIF2AK2, GADD45B, PPP1R15A, PEG3, MCL1, LITAF, TNFRSF12A, PML, STK17B, ARF6, IRAK3, TNFRSF1A, PRUNE2, PEG10, TNFRSF1B, COMP, MAP3K1, SHISA5, ZC3H12A, FGL2, INPP5D, SRGN, PHLDA1, CFLAR, ACTC1, LYZ2, NR4A2, BIRC3, NFKBIL1, CIDEC, RASSF5, CSRNP1, SULF1, CASP12, APAF1, DRAM1, PDCD7	823	519	13588	1.91	0.005805099	0.00	0.003476951
GOTERM_BP_FAT	GO:0008219~cell death	59	5.44	1.93E-06	NUAK2, NFKB1, CXCR2, TGFB2, SHB, TMEM173, CASP4, BAG3, CASP8, FAS, CASP1, MYC, BCL10, LTBR, NLRP3, BCL2L11, DDIT4, OSM, TNFRSF10B, RIPK1, GADD45G, RIPK3, TNFAIP3, EIF2AK2, GADD45B, PPP1R15A, PEG3, MCL1, LITAF, TNFRSF12A, PML, STK17B, ARF6, IRAK3, TNFRSF1A, PRUNE2, PEG10, TNFRSF1B, COMP, MAP3K1, SHISA5, ZC3H12A, FGL2, INPP5D, SRGN, PHLDA1, CFLAR, LYZ2, ACTC1, BIRC3, NFKBIL1, CIDEC, RASSF5, CSRNP1, SULF1, CASP12, APAF1, DRAM1, PDCD7	823	507	13588	1.92	0.005808084	0.00	0.003478744
GOTERM_BP_FAT	GO:0012501~programmed cell death	56	5.17	2.18E-06	NUAK2, NFKB1, CXCR2, TGFB2, SHB, TMEM173, CASP4, BAG3, CASP8, FAS, CASP1, MYC, BCL10, LTBR, NLRP3, BCL2L11, DDIT4, OSM, TNFRSF10B, RIPK1, GADD45G, RIPK3, TNFAIP3, EIF2AK2, GADD45B, PPP1R15A, PEG3, MCL1, LITAF, TNFRSF12A, PML, STK17B, ARF6, TNFRSF1A, PRUNE2, IRAK3, PEG10, COMP, MAP3K1, SHISA5, ZC3H12A, INPP5D, SRGN, PHLDA1, CFLAR, ACTC1, BIRC3, NFKBIL1, CIDEC, RASSF5, CSRNP1, CASP12, SULF1, APAF1, DRAM1, PDCD7	823	473	13588	1.95	0.006542704	0.00	0.003920182
Annotation Cluster 10	Enrichment Score: 4.535473351581223											
Category	Term	Count	%	PValue	Genes	List Total	Pop Hits	Pop Total	Fold Enrichment	Bonferroni	Benjamini	FDR
GOTERM_BP_FAT	GO:0010941~regulation of cell death	66	6.09	3.28E-07	NUAK2, FOXO1, PNP, TGFB2, BDNF, CASP4, APOE, BAG3, HMOX1, CHST11, CASP8, FAS, CASP1, MYC, BCL10, PIM1, NLRP3, BCL2L11, ADRB2, HIF1A, TNFRSF10B, BTG2, BTG1, RIPK3, IFIH1, FGFR3, MCL1, PML, STK17B, HSPA1A, HSPA1B, NR3C1, SOX9, CD74, ADA, GCH1, IRAK3, ALDH1A3, AGT, SHISA5, BCL3, FCER1G, INPP5D, SKIL, ANGPTL4, SPP1, BMP4, COL18A1, CFLAR, PTPRC, PTPN6, KLF10, TGFBR1, SPHK1, NR4A2, BIRC3, NFKBIL1, FCGR3, CIDEC, NOTCH2, CDKN1A, NUPR1, CASP12, APAF1, ID3, PDCD7, IFI204	823	563	13588	1.94	9.88E-04	0.00	5.91E-04
GOTERM_BP_FAT	GO:0042981~regulation of apoptosis	65	6.00	3.70E-07	NUAK2, FOXO1, PNP, TGFB2, BDNF, CASP4, APOE, BAG3, HMOX1, CHST11, CASP8, FAS, CASP1, MYC, BCL10, PIM1, NLRP3, BCL2L11, ADRB2, HIF1A, TNFRSF10B, BTG2, BTG1, RIPK3, IFIH1, FGFR3, MCL1, PML, STK17B, HSPA1A, HSPA1B, NR3C1, SOX9, CD74, ADA, GCH1, IRAK3, ALDH1A3, AGT, SHISA5, BCL3, FCER1G, INPP5D, SKIL, ANGPTL4, SPP1, COL18A1, CFLAR, PTPRC, PTPN6, KLF10, TGFBR1, SPHK1, NR4A2, BIRC3, NFKBIL1, FCGR3, CIDEC, NOTCH2, CDKN1A, NUPR1, CASP12, APAF1, ID3, PDCD7, IFI204	823	553	13588	1.94	0.001115723	0.00	6.67E-04

Table S1.B GO Terms upregulated 10 hours post injury

Annotation Cluster 1 | Enrichment Score: 11.839159662001325

Category	Term	Count	%	PValue	Genes	List Total	Pop Hits	Pop Total	Fold Enrichment	Bonferroni	Benjamini	FDR
GOTERM_BP_FAT	GO:0009611~response to wounding	115	4.01	7.27E-17	GNA13, NOG, F13A1, TLR1, TLR2, TLR4, C1QC, TGFB1, TGFB2, CFP, C1RA, MYD88, DYSF, CD44, CFH, CFD, TFPI2, NCF1, C4B, SERPING1, PLAUR, C1QA, C1QB, HIF1A, THBD, CCR5, DARC, F3, LYST, CCR2, TFPI, CCL2, NINJ1, CCL8, ITGB2, TRIM72, CCL7, ARG1, SLC11A1, MTPN, ITGB6, ENTPD1, FN1, PTPN6, KLF6, BMP2, CFB, EFEMP2, TLR13, SPHK1, EPHX2, CHI3L3, SMAD1, FCGR1, FCGR3, NOTCH2, SLC7A2, CXCL13, CLEC7A, HDAC9, BMP6, PPARA, PPARD, TOLLIP, LY86, HPS1, GJA1, CASP3, SLC1A3, SAA1, PAX7, LBP, NFKBIZ, GAL, NLRP3, CD163, CHST1, SERPINA3N, NPPB, NGFR, PROS1, LCP1, PTAFR, KDM6B, CXCL1, CXCL5, C3, CXCL3, CCR1, CXCL2, PF4, ABHD2, CD24A, CCL24, TNFRSF1A, ANXA8, TNFRSF1B, PYCARD, THBS1, SELP, IL6, PLEK, ANXA5, STAT3, CCL11, CCL12, ORM1, NUPR1, BAX, HBEGF, ALOX5, BMPR1B, ORM2, CD14, IGFBP4	2080	347	13588	2.17	4.59E-13	4.59E-13	2.11E-13
GOTERM_BP_FAT	GO:0006954~inflammatory response	78	2.72	1.08E-12	TOLLIP, LY86, TLR1, TLR2, TLR4, C1QC, TGFB1, CFP, C1RA, MYD88, CD44, SAA1, CFH, LBP, CFD, NFKBIZ, C4B, NCF1, SERPING1, GAL, NLRP3, CD163, CHST1, C1QA, C1QB, SERPINA3N, HIF1A, CCR5, DARC, CCR2, NPPB, PTAFR, KDM6B, CXCL1, CCL2, CXCL5, C3, CCR1, CXCL3, CXCL2, CCL8, ITGB2, CD24A, CCL7, CCL24, SLC11A1, TNFRSF1A, TNFRSF1B, ITGB6, PYCARD, THBS1, FN1, SELP, IL6, BMP2, CFB, SPHK1, TLR13, EPHX2, CHI3L3, SMAD1, FCGR1, STAT3, FCGR3, CCL11, ORM1, CCL12, NUPR1, SLC7A2, CXCL13, ALOX5, CLEC7A, HDAC9, BMPR1B, CD14, ORM2, IGFBP4, BMP6	2080	225	13588	2.26	4.45E-09	2.23E-09	2.01E-09
GOTERM_BP_FAT	GO:0006952~defense response	113	3.94	3.88E-08	TBK1, TLR1, PGLYRP1, TLR2, TLR4, C1QC, TGFB1, CFP, C1RA, MYD88, CD44, CFH, CFD, BCL10, C5AR1, NGP, NCF1, C4B, RELA, SERPING1, C1QA, C1QB, HIF1A, CCR5, DARC, LYST, CCR2, MAVS, IL1R1, CCL2, FGR, CCL8, ITGB2, CCL7, CD74, SLC11A1, ITGB6, FCER1G, DHX58, FN1, PTPN6, BMP2, CARD9, CFB, IL1RL1, IL1RL2, TLR13, SPHK1, EPHX2, MYO1F, CHI3L3, SMAD1, FCGR1, FCGR3, KCNJ8, SLC7A2, CXCL13, CLEC7A, HDAC9, RBPJ, BMP6, TOLLIP, LY86, H2-D1, TMEM173, SAA1, IL1RAP, LBP, NFKBIZ, ELANE, CLIC1, GAL, NLRP3, CD163, CHST1, SERPINA3N, NPPB, H2-AA, PTAFR, CTSG, KDM6B, CXCL1, CXCL5, C3, CXCL3, CCR1, CXCL2, CD24A, CCL24, TNFRSF1A, TNFRSF1B, PYCARD, BCL3, THBS1, PTPRC, SELP, IL6, LYZ2, HCK, COTL1, STAT3, CCL11, CCL12, ORM1, FCGR2B, NUPR1, IRF8, APAF1, ALOX5, BMPR1B, ORM2, CD14, IGFBP4	2080	448	13588	1.65	1.60E-04	1.23E-05	7.23E-05

Annotation Cluster 2 | Enrichment Score: 6.921603726439316

Category	Term	Count	%	PValue	Genes	List Total	Pop Hits	Pop Total	Fold Enrichment	Bonferroni	Benjamini	FDR
GOTERM_MF_FAT	GO:0001871~pattern binding	47	1.64	4.26E-09	HSD17B12, PGLYRP1, CCL8, POSTN, FSTL1, PF4, DCN, VIT, ABI3BP, ITGAM, CCL7, CD44, CTGF, COMP, CFH, LTF, PTX3, THBS1, GPNMB, FGF2, THBS2, CYR61, FN1, BMP4, PTPRC, TNXB, PRG4, FBN1, ELANE, CHI3L3, STAB2, TINAGL1, PCOLCE2, VEGFB, TNFAIP6, LYVE1, EGFLAM, BGN, ADAMTS8, LAYN, VEGFA, HBEGF, VCAN, ADAMTS1, LAMC2, CLEC7A, CTSG	2006	128	13288	2.43	6.04E-06	2.01E-06	7.03E-06
GOTERM_MF_FAT	GO:0030247~polysaccharide binding	47	1.64	4.26E-09	HSD17B12, PGLYRP1, CCL8, POSTN, FSTL1, PF4, DCN, VIT, ABI3BP, ITGAM, CCL7, CD44, CTGF, COMP, CFH, LTF, PTX3, THBS1, GPNMB, FGF2, THBS2, CYR61, FN1, BMP4, PTPRC, TNXB, PRG4, FBN1, ELANE, CHI3L3, STAB2, TINAGL1, PCOLCE2, VEGFB, TNFAIP6, LYVE1, EGFLAM, BGN, ADAMTS8, LAYN, VEGFA, HBEGF, VCAN, ADAMTS1, LAMC2, CLEC7A, CTSG	2006	128	13288	2.43	6.04E-06	2.01E-06	7.03E-06
GOTERM_MF_FAT	GO:0005539~glycosaminoglycan binding	41	1.43	9.18E-08	HSD17B12, PGLYRP1, CCL8, POSTN, PF4, FSTL1, DCN, VIT, ITGAM, ABI3BP, CCL7, CD44, CTGF, COMP, CFH, LTF, THBS1, GPNMB, FGF2, THBS2, CYR61, FN1, BMP4, PTPRC, TNXB, ELANE, STAB2, PCOLCE2, VEGFB, TNFAIP6, LYVE1, EGFLAM, BGN, ADAMTS8, LAYN, VEGFA, HBEGF, VCAN, LAMC2, ADAMTS1, CTSG	2006	114	13288	2.38	1.30E-04	2.60E-05	1.51E-04
GOTERM_MF_FAT	GO:0030246~carbohydrate binding	80	2.79	2.57E-06	CD209A, PGLYRP1, FSTL1, POSTN, CD209D, CD44, CTGF, CFH, CLEC4A1, LTF, CLEC4A2, FGF2, CYR61, ATRNL1, WBSCR17, PRG4, ELANE, COLEC12, CLEC4N, PCOLCE2, VEGFB, MAN2A1, TNFAIP6, EGFLAM, ADAMTS8, BGN, CLEC12A, CD33, VEGFA, LAMC2, ADAMTS1, VCAN, STBD1, CLEC5A, CTSG, SIGLECE, GALNT7, CD248, HSD17B12, GALNT4, CCL8, PF4, DCN, VIT, CLEC10A, CD24A, ITGAM, CCL7, ABI3BP, GALM, COMP, GPNMB, THBS1, PTX3, SELPLG, THBS2, FN1, MRC1, BMP4, PTPRC, SELP, TNXB, LGALS3, SELL, LGALS4, FBN1, CHI3L3, STAB2, TINAGL1, CLEC11A, LGALS9, LYVE1, GCK, LAYN, CLEC3A, GFPT2, HBEGF, CLEC7A, CD302, CLEC14A	2006	317	13288	1.67	3.63E-03	2.60E-04	4.24E-03
GOTERM_MF_FAT	GO:0008201~heparin binding	30	1.05	5.76E-06	HSD17B12, CCL8, POSTN, PF4, FSTL1, CCL7, ITGAM, ABI3BP, CTGF, COMP, CFH, LTF, GPNMB, THBS1, FGF2, THBS2, CYR61, FN1, BMP4, PTPRC, TNXB, ELANE, PCOLCE2, VEGFB, ADAMTS8, VEGFA, HBEGF, LAMC2, ADAMTS1, CTSG	2006	83	13288	2.39	8.12E-03	5.10E-04	9.50E-03

Annotation Cluster 3 | Enrichment Score: 6.534967767280406

Category	Term	Count	%	PValue	Genes	List Total	Pop Hits	Pop Total	Fold Enrichment	Bonferroni	Benjamini	FDR
GOTERM_BP_FAT	GO:0030029~actin filament-based process	60	2.09	1.18E-09	RHOJ, ENAH, LIMA1, PDGFB, NUAK2, PDGFA, AIF1, BCAR1, S100A9, PDLIM3, ACTG1, GSN, CAP1, PLS3, RHOG, FMNL1, ACTN4, ROCK2, PRKCI, ACTN1, TMSB10, MYH9, FLNC, SIRPA, WAS, FLNA, VASP, MURC, XIRP1, CFL1, AKAP2, DBN1, LCP1, MYO5A, SHROOM3, MYL2, TCAP, CNN3, MYO9B, DAAM1, ITGB1, EZR, RAC2, FAT1, SH2B2, BCL6, CNN2, WIPF1, CNN1, ACTC1, TNXB, GAS7, CORO1C, NRAS, CORO1A, EPB4.1L1, EPS8, MYH11, ABL2, DIAP1	2080	176	13588	2.23	4.89E-06	6.11E-07	2.21E-06
GOTERM_BP_FAT	GO:0030036~actin cytoskeleton organization	56	1.95	5.40E-09	RHOJ, ENAH, LIMA1, PDGFB, NUAK2, PDGFA, AIF1, BCAR1, S100A9, PDLIM3, ACTG1, GSN, CAP1, PLS3, RHOG, FMNL1, ACTN4, ROCK2, PRKCI, ACTN1, TMSB10, MYH9, SIRPA, WAS, VASP, FLNA, MURC, XIRP1, CFL1, AKAP2, DBN1, LCP1, SHROOM3, MYL2, TCAP, CNN3, DAAM1, ITGB1, EZR, RAC2, FAT1, SH2B2, BCL6, CNN2, CNN1, ACTC1, TNXB, GAS7, CORO1C, NRAS, CORO1A, EPB4.1L1, EPS8, MYH11, ABL2, DIAP1	2080	165	13588	2.22	2.23E-05	2.23E-06	1.01E-05
GOTERM_BP_FAT	GO:0007015~actin filament organization	24	0.84	2.92E-06	LIMA1, ACTC1, ENAH, ACTN4, AIF1, BCAR1, PDLIM3, PRKCI, ACTN1, GAS7, SIRPA, WAS, FLNA, CORO1A, EZR, GSN, FAT1, CFL1, AKAP2, DBN1, ABL2, LCP1, PLS3, DIAP1	2080	56	13588	2.80	1.20E-02	3.01E-04	5.44E-03

Annotation Cluster 4 | Enrichment Score: 6.498040298543319

Category	Term	Count	%	PValue	Genes	List Total	Pop Hits	Pop Total	Fold Enrichment	Bonferroni	Benjamini	FDR
GOTERM_MF_FAT	GO:0000166~nucleotide binding	428	14.91	3.23E-10	MAP3K8, VPS4B, DHX33, PTBP2, MAP2K6, DHX32, GBP5, MAGI1, ROCK2, MYLK4, GBP9, PTBP1, SPAG1, MYH7, GEM, MYH9, GLUL, MAPK4, ARL8A, MAPK7, EIF2AK3, EIF2AK4, GNAI3, MYEF2, HSPA1A, ARF6, HSPA1B, MTIF2, MUSK, RAC2, PABPC1, DHX58, DHX8, TGFBR1, SPHK1, UBE2L6, ACACB, NUBPL, KCNJ8, ARF3, ARF4, PPRC1, RAD54L2, TNK2, ACAD11, ACAD10, NUAK2, ATP10A, ADCK1, SLC22A4, EEF2K, GUCY1A3, SLC22A5, SIK1, RAP2B, SRPK2, RAP2A, AIFM2, LYN, AIFM1, PKDCC, 1700017B05RIK, NDUFA10, BBS10, MAST4, SQLE, RIPK1, EIF4A1, ARL4C, RASD2, ALDH18A1, UGDH, HK1, SF3B4, ACSL1, DGKE, SNRK, PARS2, FMO1, PTK2B, CKMT2, FMO2, HK3, CERK, ETFA, ACSL5, ACTC1, SWAP70, PDK4, AXL, NADK, ACLY, NIACR1, RPS6KA5, NRAS, RPS6KA3, RPS6KA4, SFPQ, POLDIP3, DGKZ, AACS, KATNAL2, GNA13, RNASEL, D5ERTD579E, HMGCR, RBM3, RBM15B, EIF5, INO80, RRAD, MTHFD1L, DDX27, KDM1A, RALB, DDX21, STK39, EGFR, RET, TWF2, RPS6KC1, ATP9A, BMP2K, REM1, OAS1A, LRRK1, SRXN1, NMNAT1, ABCA9, FGR, TIMP4, ABCA2, P2RY6, DDX3X, HNRNPF, TEC, TRP53, ABCB9, RAB8B, MYO1E, MET, MYO1D, MYO1G, MYO1F, DOCK7, DOCK8, DOCK6, IDH3A, IKBKE, P2RX4, DDX56, P2RX6, PLK3, PLK2, PRKAR1B, PRKAR1A, NDUFV2, MYH11, ABL2, RHOJ, KIF22, VARS2, ATL3, SLFN8, CAD, PIP5K1A, FES, SLFN4, SLFN1, SLFN2, LACE1, RHOC, TIE1, RHOG, ACADM, ACOXL, ACADS, ERAL1, ACADL, NCL, ACADVL, TOR2A, ATP6V1A, KIF1B, RRAS2, TXNRD1, BTAF1, PGS1, ASNS, IGF1R, HSPA2, ALDH1A3, CHD1, HSPA5, EHD1, DCLK1, EHD4, NOX4, AMHR2, ALPK2, HCK, ABCA8A, GRSF1, DACH1, CYBB, HSP90B1, GCK, DACH2, JAK1, ZRANB3, APAF1, IPPK, H2-DMA, ADCY4, FASTKD1, ADCY7, U2AF2, AURKA, PRKG1, 2310057M21RIK, PRKX, SULT1A1, OASL2, OASL1, GM4841, DDX10, MRPL39, RAB20, BCR, SUCLG1, POLE, PIM1, NAV3, WNK2, PPARGC1B, CARNS1, RFC5, RND3, RND1, ABCB1A, ACAP1, DLD, PDGFRA, MDH1, FUS, 5430435G22RIK, CHKA, CLCN3, PFKFB4, PFKFB3, FARS2, PFKFB2, PFKFB1, STK17B, MYO9B, IGF2BP2, CHEK2, OAS2, IDH1, SUPV3L1, POLQ, MOCS1, TRIO, TREX1, ATP13A3, ATP13A5, EPHA2, HYOU1, ATP2A2, ATP2A3, NTRK2, GRK5, SLC27A1, STEAP4, ACVRL1, CPEB2, CPEB3, CAMKK1, CAMKK2, CKB, ACTR3, TUBB5, TUBB6, NOS3, DOCK11, NT5E, DHCR24, RBM45, RAB4B, CBR4, ADK, TOP3A, SLC27A3, MAP3K14, MYO5A, RAB3A, KIT, GCH1, RAB44, IGTP, STK40, MORC4, ABCD2, SPATA5, MYO5C, CSNK1A1, IRGM2, NOS1, ATP1A1, MAPK11, RAB32, RAB31, RAB30, ADCY9, PYGL, RAB34, PHGDH, GYK, RBM14, RBM15, GRPEL2, PSTK, TBK1, CTPS, F13A1, PI4K2B, ITPKC, ACSS2, TOP1, ARL11, RBMS3, MLKL, GNL2, RBM12, RBMS1, GNL3, MYO6, CNGA3, POR, STK3, MAP4K4, PI4K2A, MORC2A, RIPK3, CSNK1G3, GBP4, FGFR3, GCLC, ACTR3B, MAP4K2, RIOK1, CMPK1, IRAK4, IRAK3,	2006	2183	13288	1.30	4.58E-07	4.58E-07	5.34E-07
GOTERM_MF_FAT	GO:0017076~purine nucleotide binding	370	12.89	2.76E-09	2310057M21RIK, PRKX, BTK, ACTG1, MAP3K6, MAP3K5, DHX37, OASL2, OASL1, MAP3K8, GM4841, VPS4B, DHX33, DDX10, RAB20, MAP2K6, DHX32, BCR, GBP5, MAGI1, ROCK2, SUCLG1, MYLK4, GBP9, SPAG1, PIM1, MYH7, WNK2, GEM, MYH9, CARNS1, RFC5, RND3, GLUL, RND1, ABCB1A, ACAP1, MAPK4, DLD, PDGFRA, ARL8A, MAPK7, EIF2AK3, EIF2AK4, 5430435G22RIK, CHKA, CLCN3, GNAI3, PFKFB4, PFKFB3, FARS2, PFKFB2, PFKFB1, STK17B, ARF6, HSPA1A, MYO9B, CHEK2, HSPA1B, OAS2, MTIF2, MUSK, RAC2, SUPV3L1, POLQ, DHX58, MOCS1, DHX8, TGFBR1, SPHK1, TRIO, UBE2L6, TREX1, ACACB, ATP13A3, ATP13A5, EPHA2, NUBPL, HYOU1, ATP2A2, ATP2A3, KCNJ8, ARF3, NTRK2, ARF4, RAD54L2, TNK2, GRK5, ACAD11, ACAD10, STEAP4, ACVRL1, NUAK2, ATP10A, CAMKK1, CAMKK2, CKB, ACTR3, ADCK1, SLC22A4, EEF2K, TUBB5, TUBB6, GUCY1A3, NOS3, SLC22A5, SIK1, DOCK11, DHCR24, RAP2B, SRPK2, RAP2A, AIFM2, LYN, AIFM1, RAB4B, PKDCC, 1700017B05RIK, NDUFA10, BBS10, MAST4, SQLE, ADK, RIPK1, EIF4A1, TOP3A, MAP3K14, ARL4C, RASD2, MYO5A, RAB3A, ALDH18A1, HK1, KIT, GCH1, RAB44, ACSL1, IGTP, SNRK, DGKE, STK40, MORC4, PARS2, FMO1, PTK2B, CKMT2, FMO2, HK3, ABCD2, CERK, SPATA5, ETFA, ACSL5, MYO5C, CSNK1A1, ACTC1, NOS1, IRGM2, SWAP70, PDK4, AXL, ACLY, NADK, MAPK11, ATP1A1, NIACR1, RPS6KA5, RAB32, NRAS, RAB31, RPS6KA3, RAB30, RPS6KA4, ADCY9, RAB34, DGKZ, GYK, AACS, KATNAL2, GNA13, GRPEL2, RNASEL, D5ERTD579E, PSTK, TBK1, CTPS, F13A1, EIF5, INO80, RRAD, ITPKC, PI4K2B, ACSS2, MTHFD1L, DDX27, TOP1, KDM1A, ARL11, RALB, MLKL, DDX21, STK39, GNL2, GNL3, EGFR, RET, TWF2, MYO6, RPS6KC1, CNGA3, STK3, MAP4K4, ATP9A, PI4K2A, MORC2A, BMP2K, RIPK3, REM1, OAS1A, CSNK1G3, GBP4, LRRK1, SRXN1, NMNAT1, FGFR3, ABCA9, GCLC, FGR, ACTR3B, MAP4K2, TIMP4, ABCA2, RIOK1, CMPK1, IRAK4, IRAK3, DDX3X, RHOBTB2, UBE2D1, RUNX1, ENTPD1, RUNX2, CSF1R, TEC, RHOBTB3, TRP53, EEF1A1, ABCB9, RAB8B, MYO1E, MET, MYO1D, MYO1G, MYO1F, DOCK7, DOCK8, DOCK6, IKBKE, P2RX4, DDX56, P2RX6, PLK3, PLK2, PRKAR1B, PRKAR1A, MYH11, RHOT2, RAP1B, SMC1A, PIP4K2A, ABL2, UBE2E2, RHOJ, KIF22, CDK17, VARS2, CDK18, ATL3, SLFN8, CAD, PIP5K1A, FES, DDR2, SLFN4, SLFN1, SLFN2, GSR, PAK2, LACE1, PAK4, ATP8B1, CDK12, MKKS, RHOC, TIE1, CSK, TUBA1A, TUBA1B, MDN1, TUBA1C, RHOG, CDK14, IRAK2, ACADM, HSP90AA1, ACOXL, ACADS, PRKCI, ERAL1, MCM3, UBE2C, NLRP3, ACADL, PRKCD, MCM4, TIMM44, MCM6, TOR2A, PANK4, ACADVL, ATP6V1A, KIF1B, PANK1, RRAS2, H2-AA, TXNRD1, NKIRAS1, BTAF1, PGS1, UBE2Z, TRIB3, ASNS, TRIB1, IGF1R, HSPA2, MAP3K2, TGM2, CHD1, UCK2, HSPA5, EHD1, DCLK1, EHD4, NOX4, AMHR2, ACTB, ALPK2, MSH2, HCK, ABCA8A, HSP90B1, CYBB, GCK, ATP8A2,	2006	1871	13288	1.31	3.91E-06	1.95E-06	4.55E-06

GOTERM_MF_FAT	GO:0032553~ribonucleotide binding	345	12.02	2.77E-07	KIFC2, ADCY4, FASTKD1, XRCC2, ADCY7, TUBB2B, TUBB2A, AURKA, PRKG1, C230081A13RIK, 2310057M21RIK, PRKX, BTK, ACTG1, MAP3K6, MAP3K5, DHX37, OASL2, OASL1, MAP3K8, GM4841, VPS4B, DHX33, DDX10, RAB20, MAP2K6, DHX32, BCR, GBP5, MAGI1, ROCK2, SUCLG1, MYLK4, GBP9, SPAG1, PIM1, MYH7, WNK2, GEM, MYH9, CARNS1, RFC5, RND3, GLUL, RND1, ABCB1A, ACAP1, MAPK4, PDGFRA, ARL8A, MAPK7, EIF2AK3, EIF2AK4, 5430435G22RIK, CHKA, CLCN3, GNAI3, PFKFB4, PFKFB3, FARS2, PFKFB2, PFKFB1, STK17B, ARF6, HSPA1A, MYO9B, CHEK2, HSPA1B, OAS2, MTIF2, MUSK, RAC2, SUPV3L1, POLQ, DHX58, MOCS1, DHX8, TGFBR1, SPHK1, TRIO, UBE2L6, ACACB, ATP13A3, ATP13A5, EPHA2, NUBPL, HYOU1, ATP2A2, ATP2A3, KCNJ8, ARF3, NTRK2, ARF4, RAD54L2, TNK2, GRK5, ACVRL1, NUAK2, ATP10A, CAMKK1, CAMKK2, CKB, ACTR3, ADCK1, SLC22A4, EEF2K, TUBB5, TUBB6, GUCY1A3, SLC22A5, SIK1, DOCK11, RAP2B, SRPK2, RAP2A, LYN, RAB4B, PKDCC, 1700017B05RIK, NDUFA10, BBS10, MAST4, ADK, RIPK1, EIF4A1, TOP3A, MAP3K14, ARL4C, RASD2, MYO5A, RAB3A, ALDH18A1, HK1, KIT, GCH1, RAB44, ACSL1, IGTP, DGKE, STK40, MORC4, SNRK, PARS2, PTK2B, CKMT2, HK3, ABCD2, CERK, SPATA5, ACSL5, MYO5C, CSNK1A1, ACTC1, IRGM2, SWAP70, PDK4, AXL, ACLY, NADK, MAPK11, ATP1A1, NIACR1, RPS6KA5, RAB32, NRAS, RAB31, RPS6KA3, RAB30, RPS6KA4, ADCY9, RAB34, DGKZ, GYK, AACS, KATNAL2, GNA13, RNASEL, D5ERTD579E, PSTK, TBK1, CTPS, F13A1, EIF5, INO80, RRAD, ITPKC, PI4K2B, ACSS2, MTHFD1L, DDX27, TOP1, ARL11, RALB, MLKL, STK39, DDX21, GNL2, GNL3, EGFR, RET, TWF2, MYO6, RPS6KC1, CNGA3, STK3, MAP4K4, ATP9A, PI4K2A, MORC2A, BMP2K, RIPK3, REM1, OAS1A, CSNK1G3, GBP4, LRRK1, SRXN1, NMNAT1, FGFR3, ABCA9, GCLC, FGR, ACTR3B, MAP4K2, TIMP4, ABCA2, RIOK1, CMPK1, IRAK4, IRAK3, DDX3X, RHOBTB2, UBE2D1, ENTPD1, RUNX1, RUNX2, CSF1R, TEC, RHOBTB3, TRP53, EEF1A1, ABCB9, RAB8B, MYO1E, MET, MYO1D, MYO1G, MYO1F, DOCK7, DOCK8, DOCK6, IKBKE, P2RX4, DDX56, P2RX6, PLK3, PLK2, PRKAR1B, PRKAR1A, MYH11, RHOT2, RAP1B, SMC1A, PIP4K2A, ABL2, UBE2E2, RHOJ, KIF22, CDK17, VARS2, CDK18, ATL3, SLFN8, CAD, PIP5K1A, FES, DDR2, SLFN4, SLFN1, SLFN2, PAK2, LACE1, PAK4, ATP8B1, CDK12, MKKS, RHOC, TIE1, CSK, TUBA1A, TUBA1B, MDN1, TUBA1C, RHOG, CDK14, IRAK2, HSP90AA1, PRKCI, ERAL1, MCM3, UBE2C, NLRP3, PRKCD, MCM4, TIMM44, MCM6, TOR2A, PANK4, ATP6V1A, KIF1B, PANK1, RRAS2, H2-AA, NKIRAS1, BTAF1, PGS1, UBE2Z, TRIB3, ASNS, TRIB1, IGF1R, HSPA2, MAP3K2, TGM2, CHD1, UCK2, HSPA5, EHD1, DCLK1, EHD4, AMHR2, ACTB, ALPK2, MSH2, HCK, ABCA8A, HSP90B1, GCK, ATP8A2, GRLF1, JAK1, APAF1, ZRANB3, IPPK, BMPR1B, H2-DMA	2006	1796	13288	1.27	3.92E-04	6.53E-05	4.56E-04
GOTERM_MF_FAT	GO:0032555~purine ribonucleotide binding	345	12.02	2.77E-07	KIFC2, ADCY4, FASTKD1, XRCC2, ADCY7, TUBB2B, TUBB2A, AURKA, PRKG1, C230081A13RIK, 2310057M21RIK, PRKX, BTK, ACTG1, MAP3K6, MAP3K5, DHX37, OASL2, OASL1, MAP3K8, GM4841, VPS4B, DHX33, DDX10, RAB20, MAP2K6, DHX32, BCR, GBP5, MAGI1, ROCK2, SUCLG1, MYLK4, GBP9, SPAG1, PIM1, MYH7, WNK2, GEM, MYH9, CARNS1, RFC5, RND3, GLUL, RND1, ABCB1A, ACAP1, MAPK4, PDGFRA, ARL8A, MAPK7, EIF2AK3, EIF2AK4, 5430435G22RIK, CHKA, CLCN3, GNAI3, PFKFB4, PFKFB3, FARS2, PFKFB2, PFKFB1, STK17B, ARF6, HSPA1A, MYO9B, CHEK2, HSPA1B, OAS2, MTIF2, MUSK, RAC2, SUPV3L1, POLQ, DHX58, MOCS1, DHX8, TGFBR1, SPHK1, TRIO, UBE2L6, ACACB, ATP13A3, ATP13A5, EPHA2, NUBPL, HYOU1, ATP2A2, ATP2A3, KCNJ8, ARF3, NTRK2, ARF4, RAD54L2, TNK2, GRK5, ACVRL1, NUAK2, ATP10A, CAMKK1, CAMKK2, CKB, ACTR3, ADCK1, SLC22A4, EEF2K, TUBB5, TUBB6, GUCY1A3, SLC22A5, SIK1, DOCK11, RAP2B, SRPK2, RAP2A, LYN, RAB4B, PKDCC, 1700017B05RIK, NDUFA10, BBS10, MAST4, ADK, RIPK1, EIF4A1, TOP3A, MAP3K14, ARL4C, RASD2, MYO5A, RAB3A, ALDH18A1, HK1, KIT, GCH1, RAB44, ACSL1, IGTP, DGKE, STK40, MORC4, SNRK, PARS2, PTK2B, CKMT2, HK3, ABCD2, CERK, SPATA5, ACSL5, MYO5C, CSNK1A1, ACTC1, IRGM2, SWAP70, PDK4, AXL, ACLY, NADK, MAPK11, ATP1A1, NIACR1, RPS6KA5, RAB32, NRAS, RAB31, RPS6KA3, RAB30, RPS6KA4, ADCY9, RAB34, DGKZ, GYK, AACS, KATNAL2, GNA13, RNASEL, D5ERTD579E, PSTK, TBK1, CTPS, F13A1, EIF5, INO80, RRAD, ITPKC, PI4K2B, ACSS2, MTHFD1L, DDX27, TOP1, ARL11, RALB, MLKL, STK39, DDX21, GNL2, GNL3, EGFR, RET, TWF2, MYO6, RPS6KC1, CNGA3, STK3, MAP4K4, ATP9A, PI4K2A, MORC2A, BMP2K, RIPK3, REM1, OAS1A, CSNK1G3, GBP4, LRRK1, SRXN1, NMNAT1, FGFR3, ABCA9, GCLC, FGR, ACTR3B, MAP4K2, TIMP4, ABCA2, RIOK1, CMPK1, IRAK4, IRAK3, DDX3X, RHOBTB2, UBE2D1, ENTPD1, RUNX1, RUNX2, CSF1R, TEC, RHOBTB3, TRP53, EEF1A1, ABCB9, RAB8B, MYO1E, MET, MYO1D, MYO1G, MYO1F, DOCK7, DOCK8, DOCK6, IKBKE, P2RX4, DDX56, P2RX6, PLK3, PLK2, PRKAR1B, PRKAR1A, MYH11, RHOT2, RAP1B, SMC1A, PIP4K2A, ABL2, UBE2E2, RHOJ, KIF22, CDK17, VARS2, CDK18, ATL3, SLFN8, CAD, PIP5K1A, FES, DDR2, SLFN4, SLFN1, SLFN2, PAK2, LACE1, PAK4, ATP8B1, CDK12, MKKS, RHOC, TIE1, CSK, TUBA1A, TUBA1B, MDN1, TUBA1C, RHOG, CDK14, IRAK2, HSP90AA1, PRKCI, ERAL1, MCM3, UBE2C, NLRP3, PRKCD, MCM4, TIMM44, MCM6, TOR2A, PANK4, ATP6V1A, KIF1B, PANK1, RRAS2, H2-AA, NKIRAS1, BTAF1, PGS1, UBE2Z, TRIB3, ASNS, TRIB1, IGF1R, HSPA2, MAP3K2, TGM2, CHD1, UCK2, HSPA5, EHD1, DCLK1, EHD4, AMHR2, ACTB, ALPK2, MSH2, HCK, ABCA8A, HSP90B1, GCK, ATP8A2, GRLF1, JAK1, APAF1, ZRANB3, IPPK, BMPR1B, H2-DMA	2006	1796	13288	1.27	3.92E-04	6.53E-05	4.56E-04
GOTERM_MF_FAT	GO:0030554~adenyl nucleotide binding	299	10.42	6.00E-07	KIFC2, ADCY4, FASTKD1, XRCC2, ADCY7, AURKA, PRKG1, C230081A13RIK, 2310057M21RIK, PRKX, BTK, ACTG1, MAP3K6, MAP3K5, DHX37, OASL2, OASL1, MAP3K8, VPS4B, DHX33, DDX10, MAP2K6, DHX32, BCR, MAGI1, ROCK2, MYLK4, PIM1, MYH7, WNK2, MYH9, CARNS1, RFC5, GLUL, ABCB1A, ACAP1, MAPK4, DLD, PDGFRA, MAPK7, EIF2AK3, EIF2AK4, CHKA, CLCN3, PFKFB4, PFKFB3, FARS2, PFKFB2, PFKFB1, STK17B, MYO9B, HSPA1A, CHEK2, OAS2, HSPA1B, MUSK, SUPV3L1, POLQ, DHX58, DHX8, TGFBR1, SPHK1, TRIO, UBE2L6, TREX1, ACACB, ATP13A3, ATP13A5, EPHA2, NUBPL, HYOU1, ATP2A2, ATP2A3, KCNJ8, NTRK2, RAD54L2, TNK2, GRK5, ACAD11, ACAD10, STEAP4, ACVRL1, NUAK2, ATP10A, CAMKK1, CAMKK2, CKB, ACTR3, ADCK1, SLC22A4, EEF2K, NOS3, SLC22A5, SIK1, DHCR24, SRPK2, AIFM2, LYN, AIFM1, PKDCC, NDUFA10, BBS10, MAST4, SQLE, ADK, RIPK1, EIF4A1, TOP3A, MAP3K14, MYO5A, ALDH18A1, HK1, KIT, ACSL1, DGKE, STK40, MORC4, SNRK, FMO1, PTK2B, PARS2, CKMT2, FMO2, HK3, ABCD2, CERK, SPATA5, ETFA, ACSL5, MYO5C, CSNK1A1, ACTC1, NOS1, SWAP70, PDK4, AXL, ACLY, NADK, MAPK11, ATP1A1, RPS6KA5, RPS6KA3, RPS6KA4, ADCY9, DGKZ, GYK, AACS, KATNAL2, GRPEL2, RNASEL, D5ERTD579E, PSTK, TBK1, CTPS, F13A1, INO80, ITPKC, PI4K2B, ACSS2, MTHFD1L, DDX27, TOP1, KDM1A, MLKL, STK39, DDX21, EGFR, RET, TWF2, MYO6, RPS6KC1, STK3, MAP4K4, ATP9A, PI4K2A, MORC2A, BMP2K, RIPK3, CSNK1G3, OAS1A, LRRK1, SRXN1, NMNAT1, FGFR3, ABCA9, GCLC, FGR, ACTR3B, MAP4K2, TIMP4, ABCA2, RIOK1, CMPK1, IRAK4, IRAK3, DDX3X, UBE2D1, ENTPD1, RUNX1, RUNX2, CSF1R, TEC, RHOBTB3, TRP53, ABCB9, MYO1E, MET, MYO1D, MYO1G, MYO1F, IKBKE, P2RX4, DDX56, P2RX6, PLK3, PLK2, PRKAR1B, PRKAR1A, MYH11, PIP4K2A, SMC1A, ABL2, UBE2E2, KIF22, CDK17, CDK18, VARS2, SLFN8, CAD, PIP5K1A, FES, DDR2, SLFN4, SLFN1, SLFN2, GSR, PAK2, LACE1, PAK4, ATP8B1, CDK12, MKKS, TIE1, CSK, MDN1, CDK14, IRAK2, ACADM, HSP90AA1, ACOXL, ACADS, PRKCI, MCM3, UBE2C, NLRP3, ACADL, PRKCD, MCM4, TIMM44, MCM6, PANK4, ACADVL, TOR2A, ATP6V1A, KIF1B, PANK1, H2-AA, TXNRD1, BTAF1, PGS1, UBE2Z, TRIB3, ASNS, TRIB1, IGF1R, HSPA2, MAP3K2, CHD1, UCK2, HSPA5, EHD1, DCLK1, EHD4, NOX4, AMHR2, ACTB, ALPK2, MSH2, HCK, ABCA8A, HSP90B1, CYBB, GCK, ATP8A2, JAK1, APAF1, ZRANB3, IPPK, BMPR1B, H2-DMA	2006	1535	13288	1.29	8.49E-04	1.06E-04	9.89E-04
GOTERM_MF_FAT	GO:0001882~nucleoside binding	302	10.52	7.86E-07	KIFC2, ADCY4, FASTKD1, XRCC2, ADCY7, AURKA, PRKG1, C230081A13RIK, 2310057M21RIK, PRKX, BTK, ACTG1, MAP3K6, MAP3K5, DHX37, OASL2, OASL1, MAP3K8, VPS4B, DHX33, DDX10, MAP2K6, DHX32, BCR, MAGI1, ROCK2, MYLK4, PIM1, MYH7, WNK2, MYH9, CARNS1, RFC5, GLUL, ABCB1A, ACAP1, MAPK4, DLD, PDGFRA, MAPK7, EIF2AK3, EIF2AK4, CHKA, CLCN3, PFKFB4, PFKFB3, FARS2, PFKFB2, PFKFB1, STK17B, MYO9B, HSPA1A, CHEK2, OAS2, HSPA1B, MUSK, SUPV3L1, POLQ, SLC28A2, SLC28A1, DHX58, DHX8, TGFBR1, SPHK1, TRIO, UBE2L6, TREX1, ACACB, ATP13A3, ATP13A5, EPHA2, NUBPL, HYOU1, ATP2A2, ATP2A3, KCNJ8, NTRK2, RAD54L2, TNK2, GRK5, ACAD11, ACAD10, STEAP4, ACVRL1, NUAK2, ATP10A, CAMKK1, CAMKK2, CKB, ACTR3, ADCK1, SLC22A4, EEF2K, NOS3, SLC22A5, SIK1, DHCR24, SRPK2, AIFM2, LYN, AIFM1, PKDCC, NDUFA10, BBS10, MAST4, SQLE, ADK, RIPK1, EIF4A1, TOP3A, MAP3K14, MYO5A, ALDH18A1, HK1, KIT, ACSL1, DGKE, STK40, MORC4, SNRK, FMO1, PTK2B, PARS2, CKMT2, FMO2, HK3, ABCD2, CERK, SPATA5, ETFA, ACSL5, MYO5C, CSNK1A1, ACTC1, NOS1, SWAP70, PDK4, AXL, ACLY, NADK, MAPK11, ATP1A1, RPS6KA5, RPS6KA3, RPS6KA4, ADCY9, DGKZ, GYK, AACS, KATNAL2, GRPEL2, RNASEL, D5ERTD579E, PSTK, TBK1, CTPS, F13A1, INO80, ITPKC, PI4K2B, ACSS2, MTHFD1L, DDX27, TOP1, KDM1A, MLKL, STK39, DDX21, EGFR, RET, TWF2, MYO6, RPS6KC1, CNGA3, STK3, MAP4K4, ATP9A, PI4K2A, MORC2A, BMP2K, RIPK3, CSNK1G3, OAS1A, LRRK1, SRXN1, NMNAT1, FGFR3, ABCA9, GCLC, FGR, ACTR3B, MAP4K2, TIMP4, ABCA2, RIOK1, CMPK1, IRAK4, IRAK3, DDX3X, UBE2D1, ENTPD1, RUNX1, RUNX2, CSF1R, TEC, RHOBTB3, TRP53, ABCB9, MYO1E, MET, MYO1D, MYO1G, MYO1F, IKBKE, P2RX4, DDX56, P2RX6, PLK3, PLK2, PRKAR1B, PRKAR1A, MYH11, PIP4K2A, SMC1A, ABL2, UBE2E2, KIF22, CDK17, CDK18, VARS2, SLFN8, CAD, PIP5K1A, FES, DDR2, SLFN4, SLFN1, SLFN2, GSR, PAK2, LACE1, PAK4, ATP8B1, CDK12, MKKS, TIE1, CSK, MDN1, CDK14, IRAK2, ACADM, HSP90AA1, ACOXL, ACADS, PRKCI, MCM3, UBE2C, NLRP3, ACADL, PRKCD, MCM4, TIMM44, MCM6, TOR2A, PANK4, ACADVL, ATP6V1A, KIF1B, PANK1, H2-AA, TXNRD1, BTAF1, PGS1, UBE2Z, TRIB3, ASNS, TRIB1, IGF1R, HSPA2, MAP3K2, CHD1, UCK2, HSPA5, EHD1, DCLK1, EHD4, NOX4, AMHR2, ACTB, ALPK2, MSH2, HCK, ABCA8A, HSP90B1, CYBB, GCK, ATP8A2, JAK1, APAF1, ZRANB3, IPPK, BMPR1B, H2-DMA	2006	1558	13288	1.28	1.11E-03	1.11E-04	1.30E-03
GOTERM_MF_FAT	GO:0001883~purine nucleoside binding	300	10.45	9.20E-07	KIFC2, ADCY4, FASTKD1, XRCC2, ADCY7, AURKA, PRKG1, C230081A13RIK, 2310057M21RIK, PRKX, BTK, ACTG1, MAP3K6, MAP3K5, DHX37, OASL2, OASL1, MAP3K8, VPS4B, DHX33, DDX10, MAP2K6, DHX32, BCR, MAGI1, ROCK2, MYLK4, PIM1, MYH7, WNK2, MYH9, CARNS1, RFC5, GLUL, ABCB1A, ACAP1, MAPK4, DLD, PDGFRA, MAPK7, EIF2AK3, EIF2AK4, CHKA, CLCN3, PFKFB4, PFKFB3, FARS2, PFKFB2, PFKFB1, STK17B, MYO9B, HSPA1A, CHEK2, OAS2, HSPA1B, MUSK, SUPV3L1, POLQ, DHX58, DHX8, TGFBR1, SPHK1, TRIO, UBE2L6, TREX1, ACACB, ATP13A3, ATP13A5, EPHA2, NUBPL, HYOU1, ATP2A2, ATP2A3, KCNJ8, NTRK2, RAD54L2, TNK2, GRK5, ACAD11, ACAD10, STEAP4, ACVRL1, NUAK2, ATP10A, CAMKK1, CAMKK2, CKB, ACTR3, ADCK1, SLC22A4, EEF2K, NOS3, SLC22A5, SIK1, DHCR24, SRPK2, AIFM2, LYN, AIFM1, PKDCC, NDUFA10, BBS10, MAST4, SQLE, ADK, RIPK1, EIF4A1, TOP3A, MAP3K14, MYO5A, ALDH18A1, HK1, KIT, ACSL1, DGKE, STK40, MORC4, SNRK, FMO1, PTK2B, PARS2, CKMT2, FMO2, HK3, ABCD2, CERK, SPATA5, ETFA, ACSL5, MYO5C, CSNK1A1, ACTC1, NOS1, SWAP70, PDK4, AXL, ACLY, NADK, MAPK11, ATP1A1, RPS6KA5, RPS6KA3, RPS6KA4, ADCY9, DGKZ, GYK, AACS, KATNAL2, GRPEL2, RNASEL, D5ERTD579E, PSTK, TBK1, CTPS, F13A1, INO80, ITPKC, PI4K2B, ACSS2, MTHFD1L, DDX27, TOP1, KDM1A, MLKL, STK39, DDX21, EGFR, RET, TWF2, MYO6, RPS6KC1, CNGA3, STK3, MAP4K4, ATP9A, PI4K2A, MORC2A, BMP2K, RIPK3, CSNK1G3, OAS1A, LRRK1, SRXN1, NMNAT1, FGFR3, ABCA9, GCLC, FGR, ACTR3B, MAP4K2, TIMP4, ABCA2, RIOK1, CMPK1, IRAK4, IRAK3, DDX3X, UBE2D1, ENTPD1, RUNX1, RUNX2, CSF1R, TEC, RHOBTB3, TRP53, ABCB9, MYO1E, MET, MYO1D, MYO1G, MYO1F, IKBKE, P2RX4, DDX56, P2RX6, PLK3, PLK2, PRKAR1B, PRKAR1A, MYH11, PIP4K2A, SMC1A, ABL2, UBE2E2, KIF22, CDK17, CDK18, VARS2, SLFN8, CAD, PIP5K1A, FES, DDR2, SLFN4, SLFN1, SLFN2, GSR, PAK2, LACE1, PAK4, ATP8B1, CDK12, MKKS, TIE1, CSK, MDN1, CDK14, IRAK2, ACADM, HSP90AA1, ACOXL, ACADS, PRKCI, MCM3, UBE2C, NLRP3, ACADL, PRKCD, MCM4, TIMM44, MCM6, TOR2A, PANK4, ACADVL, ATP6V1A, KIF1B, PANK1, H2-AA, TXNRD1, BTAF1, PGS1, UBE2Z, TRIB3, ASNS, TRIB1, IGF1R, HSPA2, MAP3K2, CHD1, UCK2, HSPA5, EHD1, DCLK1, EHD4, NOX4, AMHR2, ACTB, ALPK2, MSH2, HCK, ABCA8A, HSP90B1, CYBB, GCK, ATP8A2, JAK1, APAF1, ZRANB3, IPPK, BMPR1B, H2-DMA	2006	1548	13288	1.28	1.30E-03	1.18E-04	1.52E-03

GOTERM_MF_FAT	GO:0005524~ATP binding	272	9.48	2.89E-05	KIFC2, ADCY4, FASTKD1, XRCC2, ADCY7, AURKA, PRKG1, C230081A13RIK, 2310057M21RIK, PRKX, BTK, ACTG1, MAP3K6, MAP3K5, DHX37, OASL2, OASL1, MAP3K8, VPS4B, DHX33, DDX10, MAP2K6, DHX32, BCR, MAGI1, ROCK2, MYLK4, PIM1, MYH7, WNK2, MYH9, CARNS1, RFC5, GLUL, ABCB1A, MAPK4, ACAP1, PDGFRA, MAPK7, EIF2AK3, EIF2AK4, CHKA, CLCN3, PFKFB4, PFKFB3, FARS2, PFKFB2, PFKFB1, STK17B, MYO9B, HSPA1A, CHEK2, OAS2, HSPA1B, MUSK, SUPV3L1, POLQ, DHX58, DHX8, TGFBR1, SPHK1, TRIO, UBE2L6, ACACB, ATP13A3, ATP13A5, EPHA2, NUBPL, HYOU1, ATP2A2, ATP2A3, KCNJ8, NTRK2, RAD54L2, TNK2, GRK5, ACVRL1, NUAK2, ATP10A, CAMKK1, CAMKK2, CKB, ACTR3, ADCK1, SLC22A4, EEF2K, SLC22A5, SIK1, SRPK2, LYN, PKDCC, NDUFA10, BBS10, MAST4, ADK, RIPK1, EIF4A1, TOP3A, MAP3K14, MYO5A, ALDH18A1, HK1, KIT, ACSL1, DGKE, STK40, MORC4, SNRK, PTK2B, PARS2, CKMT2, HK3, ABCD2, CERK, SPATA5, ACSL5, MYO5C, CSNK1A1, ACTC1, SWAP70, PDK4, AXL, ACLY, NADK, MAPK11, ATP1A1, RPS6KA5, RPS6KA3, RPS6KA4, ADCY9, DGKZ, GYK, AACS, KATNAL2, RNASEL, D5ERTD579E, PSTK, TBK1, CTPS, F13A1, INO80, PI4K2B, ITPKC, ACSS2, MTHFD1L, DDX27, TOP1, MLKL, STK39, DDX21, EGFR, RET, TWF2, MYO6, RPS6KC1, STK3, MAP4K4, ATP9A, PI4K2A, MORC2A, BMP2K, RIPK3, CSNK1G3, OAS1A, LRRK1, SRXN1, NMNAT1, FGFR3, ABCA9, GCLC, FGR, ACTR3B, MAP4K2, TIMP4, ABCA2, RIOK1, CMPK1, IRAK4, IRAK3, DDX3X, ENTPD1, RUNX1, UBE2D1, RUNX2, CSF1R, TEC, RHOBTB3, TRP53, ABCB9, MYO1E, MYO1D, MET, MYO1G, MYO1F, IKBKE, P2RX4, DDX56, PLK3, P2RX6, PLK2, MYH11, PIP4K2A, SMC1A, ABL2, UBE2E2, KIF22, CDK17, CDK18, VARS2, SLFN8, CAD, PIP5K1A, FES, DDR2, SLFN4, SLFN1, SLFN2, PAK2, LACE1, PAK4, ATP8B1, CDK12, MKKS, TIE1, CSK, MDN1, CDK14, IRAK2, HSP90AA1, PRKCI, MCM3, UBE2C, NLRP3, TIMM44, MCM4, PRKCD, MCM6, PANK4, TOR2A, ATP6V1A, KIF1B, PANK1, H2-AA, BTAF1, PGS1, UBE2Z, TRIB3, ASNS, TRIB1, IGF1R, HSPA2, MAP3K2, CHD1, UCK2, HSPA5, EHD1, DCLK1, EHD4, AMHR2, ACTB, ALPK2, MSH2, HCK, ABCA8A, HSP90B1, GCK, ATP8A2, JAK1, APAF1, ZRANB3, IPPK, BMPR1B, H2-DMA	2006	1443	13288	1.25	4.02E-02	1.86E-03	4.77E-02

Annotation Cluster 5 Enrichment Score: 5.914091733768272

Category	Term	Count	%	PValue	Genes	List Total	Pop Hits	Pop Total	Fold Enrichment	Bonferroni	Benjamini	FDR
GOTERM_BP_FAT	GO:0002460~adaptive immune response based on somatic recombination of immune receptors built fr	34	1.18	7.82E-08	C3, NFKB2, C1QC, CD74, C1RA, SLC11A1, MYD88, FCER1G, BCL3, BCL6, FAS, INPP5D, BCL10, ICAM1, ICOSL, C4B, CFB, SWAP70, MSH2, RELB, SERPING1, FCGR1, PRKCD, FCGR3, C1QA, C1QB, IL18BP, FCGR2B, LILRB3, LYST, VEGFA, GADD45G, H2-AA, H2-DMA	2080	84	13588	2.64	3.23E-04	2.15E-05	1.46E-04
GOTERM_BP_FAT	GO:0002250~adaptive immune response	34	1.18	7.82E-08	C3, NFKB2, C1QC, CD74, C1RA, SLC11A1, MYD88, FCER1G, BCL3, BCL6, FAS, INPP5D, BCL10, ICAM1, ICOSL, C4B, CFB, SWAP70, MSH2, RELB, SERPING1, FCGR1, PRKCD, FCGR3, C1QA, C1QB, IL18BP, FCGR2B, LILRB3, LYST, VEGFA, GADD45G, H2-AA, H2-DMA	2080	84	13588	2.64	3.23E-04	2.15E-05	1.46E-04
GOTERM_BP_FAT	GO:0002252~immune effector process	43	1.50	3.37E-07	C3, C1QC, CD74, CFP, C1RA, SLC11A1, MYD88, CFH, FCER1G, BCL3, FAS, INPP5D, LBP, PTX3, CFD, PTPRC, ICAM1, PTPN6, BCL10, ICOSL, CARD9, C4B, CFB, MSH2, NCF1, SWAP70, ELANE, MYO1F, SERPING1, NLRP3, FCGR1, PRKCD, FCGR3, C1QA, C1QB, LAT2, FCGR2B, KCNJ8, LILRB3, LYST, H2-AA, H2-DMA, CTSG	2080	126	13588	2.23	1.39E-03	6.97E-05	6.29E-04
GOTERM_BP_FAT	GO:0002443~leukocyte mediated immunity	34	1.18	3.87E-07	C3, C1QC, CD74, C1RA, SLC11A1, MYD88, FCER1G, BCL3, FAS, INPP5D, PTPN6, BCL10, ICAM1, ICOSL, C4B, CFB, MSH2, SWAP70, NCF1, ELANE, MYO1F, SERPING1, FCGR1, PRKCD, FCGR3, C1QA, C1QB, LAT2, FCGR2B, LILRB3, LYST, H2-AA, H2-DMA, CTSG	2080	89	13588	2.50	1.60E-03	6.96E-05	7.22E-04
GOTERM_BP_FAT	GO:0002449~lymphocyte mediated immunity	29	1.01	3.35E-06	C3, C1QC, CD74, C1RA, SLC11A1, MYD88, FCER1G, BCL3, INPP5D, FAS, PTPN6, BCL10, ICAM1, ICOSL, C4B, SWAP70, MSH2, CFB, SERPING1, PRKCD, FCGR1, FCGR3, C1QA, C1QB, FCGR2B, LILRB3, LYST, H2-AA, H2-DMA	2080	76	13588	2.49	1.38E-02	3.15E-04	6.25E-03
GOTERM_BP_FAT	GO:0019724~B cell mediated immunity	25	0.87	1.58E-05	C3, C1QC, CD74, C1RA, MYD88, FCER1G, BCL3, INPP5D, FAS, BCL10, ICOSL, SWAP70, MSH2, CFB, C4B, SERPING1, PRKCD, FCGR1, FCGR3, C1QA, C1QB, FCGR2B, LILRB3, H2-AA, H2-DMA	2080	65	13588	2.51	6.33E-02	1.01E-03	2.95E-02

Annotation Cluster 6 Enrichment Score: 5.684403597851492

Category	Term	Count	%	PValue	Genes	List Total	Pop Hits	Pop Total	Fold Enrichment	Bonferroni	Benjamini	FDR
GOTERM_MF_FAT	GO:0048037~cofactor binding	67	2.33	3.75E-08	STEAP4, HMGCR, EHHADH, PGD, NDUFAB1, GOT2, NDUFS7, GSR, KDM1A, ACBD3, ALAS1, ALAS2, GOT1, HDC, NOS3, DHTKD1, HADH, DHCR24, SGPL1, ACADM, ACOXL, SPTLC2, AIFM2, AIFM1, ACADS, CBR4, ACADL, POR, ACADVL, CTH, SQLE, DLD, TXNRD1, AOC3, MDH1, GCLC, UGDH, CSAD, FMO1, ALDH1A3, FMO2, FASN, IDH1, GALE, PDHX, ETFA, NOX4, DLST, SHMT1, NOS1, GMDS, NDUFA9, SIRT4, SIRT5, DLAT, SIRT1, IDH3A, SIRT3, DBT, CYBB, PYGL, NDUFV2, PHGDH, GPT, ACAD11, PSAT1, ACAD10	2006	226	13288	1.96	5.31E-05	1.33E-05	6.19E-05
GOTERM_MF_FAT	GO:0050662~coenzyme binding	50	1.74	4.43E-07	STEAP4, HMGCR, EHHADH, PGD, KDM1A, GSR, ACBD3, NOS3, DHTKD1, HADH, DHCR24, ACADM, AIFM2, ACOXL, ACADS, AIFM1, CBR4, ACADL, POR, ACADVL, SQLE, DLD, TXNRD1, MDH1, GCLC, UGDH, FMO1, FMO2, ALDH1A3, IDH1, GALE, PDHX, ETFA, NOX4, DLST, NOS1, GMDS, NDUFA9, SIRT4, SIRT5, DLAT, SIRT1, IDH3A, SIRT3, DBT, CYBB, NDUFV2, PHGDH, ACAD11, ACAD10	2006	160	13288	2.07	6.27E-04	8.96E-05	7.31E-04

Annotation Cluster 7 Enrichment Score: 5.4564121294507935

Category	Term	Count	%	PValue	Genes	List Total	Pop Hits	Pop Total	Fold Enrichment	Bonferroni	Benjamini	FDR
GOTERM_BP_FAT	GO:0001501~skeletal system development	85	2.96	6.52E-10	NOG, MMP9, EDN1, TGFB3, PRRX2, ZIC1, GJA5, TGFB1, TGFB2, HOXC6, PGP, OSR2, CTGF, PAX7, CHST11, INSIG1, TRP63, COL11A2, MYC, COL11A1, IDUA, SGPL1, ARID5B, MGP, SIX2, SIX4, MMP14, MMP13, HOXD9, PTHLH, SMO, HOXD8, HIF1A, CHRDL1, NAB2, SIX1, UBC, ZFP697, PDGFRA, FOXC1, WNT9A, COL1A1, UBB, EIF2AK3, SERP1, WNT5A, IBSP, FGFR3, PTH1R, TIPARP, CYTL1, FHL2, ACP5, COL2A1, OSTN, SOX9, COL9A1, JUND, MYOG, RUNX1, GPNMB, MN1, RUNX2, PCSK5, SPP1, BMP4, BMP2, LGALS3, TGFBR1, KLF10, SMAD5, SMAD3, TBX1, SMAD1, GAS1, COL5A2, EPHA2, RDH10, GLA, CSRNP1, PBX1, BMPR1B, IGFBP3, TCF15, IGFBP5, BMP6	2080	285	13588	1.95	2.69E-06	3.85E-07	1.22E-06
GOTERM_BP_FAT	GO:0060348~bone development	37	1.29	2.16E-05	IBSP, NOG, PTH1R, ACP5, FHL2, COL2A1, OSTN, SOX9, CTGF, JUND, INSIG1, MYOG, GPNMB, RUNX2, MN1, SPP1, BMP4, BMP2, KLF10, SMAD5, SMAD3, MGP, SMAD1, MMP14, MMP13, PTHLH, SMO, CHRDL1, GLA, NAB2, UBC, FOXC1, UBB, COL1A1, IGFBP3, EIF2AK3, BMP6, IGFBP5	2080	118	13588	2.05	8.54E-02	1.27E-03	4.03E-02

Annotation Cluster 8 Enrichment Score: 5.378494610899322

Category	Term	Count	%	PValue	Genes	List Total	Pop Hits	Pop Total	Fold Enrichment	Bonferroni	Benjamini	FDR
GOTERM_BP_FAT	GO:0001944~vasculature development	69	2.40	7.79E-07	GNA13, ACVRL1, PDGFA, PGF, EDN1, GJA1, PRRX2, GJA5, CITED1, CITED2, TGFB2, SHB, ARHGAP22, CTGF, HMOX1, CCBE1, CASP8, NOS3, LOX, FGF2, ANGPT2, CYR61, PTPRJ, SGPL1, SOCS3, PDPN, MMP19, MYH9, MMP14, JUNB, THY1, VEGFB, SMO, HIF1A, VEGFA, ZFP697, FOXC1, COL1A1, TNFAIP2, TNFRSF12A, COL3A1, TIPARP, MEIS1, ALDH1A2, AGT, GATAD2A, TGM2, ZC3H12A, PLCD1, KLF5, BMP4, COL18A1, EPAS1, TGFBR1, MYO1E, SPHK1, SMAD5, TBX1, FZD5, EPHA2, ANXA2, LAMA4, ID1, BAX, ECSCR, NTRK2, HBEGF, RBPJ, RBM15	2080	250	13588	1.80	3.21E-03	1.15E-04	1.45E-03
GOTERM_BP_FAT	GO:0001568~blood vessel development	67	2.33	1.39E-06	GNA13, ACVRL1, PDGFA, PGF, EDN1, GJA1, PRRX2, GJA5, CITED1, CITED2, TGFB2, SHB, ARHGAP22, CTGF, HMOX1, CCBE1, CASP8, NOS3, LOX, FGF2, ANGPT2, CYR61, PTPRJ, SGPL1, SOCS3, MMP19, MYH9, MMP14, JUNB, THY1, VEGFB, SMO, HIF1A, VEGFA, ZFP697, FOXC1, COL1A1, TNFAIP2, TNFRSF12A, COL3A1, TIPARP, MEIS1, ALDH1A2, AGT, TGM2, GATAD2A, ZC3H12A, PLCD1, KLF5, BMP4, COL18A1, EPAS1, TGFBR1, MYO1E, SPHK1, SMAD5, TBX1, FZD5, EPHA2, ANXA2, LAMA4, BAX, ECSCR, NTRK2, HBEGF, RBPJ, RBM15	2080	244	13588	1.79	5.74E-03	1.80E-04	2.60E-03
GOTERM_BP_FAT	GO:0048514~blood vessel morphogenesis	56	1.95	4.35E-06	GNA13, ACVRL1, PDGFA, PGF, EDN1, GJA1, PRRX2, CITED1, TGFB2, CITED2, SHB, ARHGAP22, CTGF, HMOX1, CCBE1, CASP8, NOS3, ANGPT2, FGF2, CYR61, PTPRJ, SGPL1, MMP19, MYH9, MMP14, JUNB, THY1, VEGFB, SMO, HIF1A, VEGFA, FOXC1, TNFAIP2, TNFRSF12A, TIPARP, MEIS1, TGM2, ZC3H12A, PLCD1, BMP4, KLF5, COL18A1, EPAS1, MYO1E, TGFBR1, SMAD5, TBX1, FZD5, EPHA2, ANXA2, BAX, ECSCR, NTRK2, HBEGF, RBPJ, RBM15	2080	198	13588	1.85	1.78E-02	3.74E-04	8.11E-03

Annotation Cluster 9 Enrichment Score: 5.284521889147466

Category	Term	Count	%	PValue	Genes	List Total	Pop Hits	Pop Total	Fold Enrichment	Bonferroni	Benjamini	FDR
GOTERM_BP_FAT	GO:0016477~cell migration	70	2.44	6.48E-08	PVR, PPARD, PDGFB, PLXNA2, BCAR1, S100A9, GJA1, ZEB2, PRKG1, GDNF, TGFB1, TGFB2, EDNRB, GAB2, CTGF, SEMA3F, CSF3R, POU4F1, CAP1, LBP, FMNL1, EGFR, SGPL1, RET, CCDC88A, C5AR1, ARID5B, EMX2, ELANE, SIX4, MYH9, MMP14, SMO, TNS3, HIF1A, LYST, SIX1, VEGFA, CFL1, FOXC1, LAMC1, CDK5R1, TNFRSF12A, CCR1, CXCL3, CXCL2, PF4, ITGB2, KIT, ITGB1, CD24A, ITGAM, DOCK1, FCER1G, CLASP2, SELPLG, DCLK1, TES, PLAT, SELP, TGFBR1, MET, NR4A2, TBX1, NTN1, FCGR3, CORO1A, BAX, HBEGF, PLAU	2080	240	13588	1.91	2.67E-04	1.91E-05	1.21E-04
GOTERM_BP_FAT	GO:0006928~cell motion	87	3.03	2.21E-05	PVR, PPARD, ENAH, PDGFB, PLXNA2, BCAR1, S100A9, GJA1, ZEB2, PRKG1, GDNF, TGFB1, TGFB2, EDNRB, GAB2, CTGF, SEMA3F, CSF3R, POU4F1, CAP1, LBP, BOC, FMNL1, EGFR, SGPL1, RET, CCDC88A, C5AR1, EGR2, PDPN, ARID5B, ELANE, EMX2, SIX4, MYH9, MMP14, SIRPA, VASP, SLIT3, SMO, TNS3, CATSPER4, HIF1A, LYST, SIX1, VEGFA, CFL1, FOXC1, LAMC1, NGFR, CDK5R1, TNFRSF12A, CCR1, CXCL3, CXCL2, ITGB2, PF4, KIT, CDH4, ITGB1, CD24A, ITGAM, PXN, ALCAM, CD9, DOCK1, FCER1G, B3GNT2, CLASP2, SELPLG, DCLK1, TES, PLAT, SELP, TGFBR1, MET, NR4A2, TBX1, GAS1, NTN1, FCGR3, CORO1A, SEMA6C, BAX, HBEGF, BMPR1B, PLAU	2080	367	13588	1.55	8.71E-02	1.28E-03	4.11E-02
GOTERM_BP_FAT	GO:0051674~localization of cell	71	2.47	2.26E-05	PVR, PPARD, PDGFB, PLXNA2, BCAR1, S100A9, GJA1, ZEB2, PRKG1, GDNF, TGFB1, TGFB2, EDNRB, GAB2, CTGF, SEMA3F, CSF3R, POU4F1, CAP1, LBP, FMNL1, EGFR, SGPL1, RET, CCDC88A, C5AR1, ARID5B, EMX2, ELANE, SIX4, MYH9, MMP14, SMO, TNS3, CATSPER4, HIF1A, LYST, SIX1, VEGFA, CFL1, FOXC1, LAMC1, CDK5R1, TNFRSF12A, CCR1, CXCL3, CXCL2, ITGB2, PF4, KIT, ITGB1, CD24A, ITGAM, DOCK1, FCER1G, CLASP2, SELPLG, DCLK1, TES, PLAT, SELP, TGFBR1, MET, NR4A2, TBX1, NTN1, FCGR3, CORO1A, BAX, HBEGF, PLAU	2080	284	13588	1.63	8.90E-02	1.29E-03	4.21E-02
GOTERM_BP_FAT	GO:0048870~cell motility	71	2.47	2.26E-05	PVR, PPARD, PDGFB, PLXNA2, BCAR1, S100A9, GJA1, ZEB2, PRKG1, GDNF, TGFB1, TGFB2, EDNRB, GAB2, CTGF, SEMA3F, CSF3R, POU4F1, CAP1, LBP, FMNL1, EGFR, SGPL1, RET, CCDC88A, C5AR1, ARID5B, EMX2, ELANE, SIX4, MYH9, MMP14, SMO, TNS3, CATSPER4, HIF1A, LYST, SIX1, VEGFA, CFL1, FOXC1, LAMC1, CDK5R1, TNFRSF12A, CCR1, CXCL3, CXCL2, ITGB2, PF4, KIT, ITGB1, CD24A, ITGAM, DOCK1, FCER1G, CLASP2, SELPLG, DCLK1, TES, PLAT, SELP, TGFBR1, MET, NR4A2, TBX1, NTN1, FCGR3, CORO1A, BAX, HBEGF, PLAU	2080	284	13588	1.63	8.90E-02	1.29E-03	4.21E-02

Annotation Cluster 10 Enrichment Score: 5.125914727257216

Category	Term	Count	%	PValue	Genes	List Total	Pop Hits	Pop Total	Fold Enrichment	Bonferroni	Benjamini	FDR
GOTERM_BP_FAT	GO:0012501~programmed cell death	110	3.83	4.17E-06	FASTKD1, CADM1, PGLYRP1, TGFB2, SHB, MAP3K5, ANK2, TRP63, FAS, DAP, SGPL1, BCL10, NCF1, BCL2L11, STK3, RELT, SIAH1A, TNFAIP8, RIPK3, MAPK7, TNFAIP3, EIF2AK3, LST1, MCL1, LITAF, TNFRSF12A, ADAMTSL4, STK17B, ARF6, BCL2L1, IRAK3, PEG10, DOCK1, TCTN3, SRGN, TRP53, HTT, KLF11, RYBP, GAS1, NTN1, TNFSF10, BCL2A1A, BBC3, CSRNP1, RHOT2, PDCD7, NUAK2, SGPP1, CXCR2, ASAH2, TMEM173, CASP3, TNFRSF11B, CASP4, PAK2, BAG3, CASP8, FGF2, MYC, DEDD2, IRAK2, LTBR, AIFM1, CYCS, TOPORS, NLRP3, TNFRSF10B, RIPK1, GADD45G, SH3KBP1, MFSD10, NGFR, SIAH2, GADD45B, PPP1R15A, BID, TNFRSF21, UBE2Z, PML, TRIB3, KIT, FEM1B, CD24A, TNFRSF1A, SQSTM1, COMP, SHISA5, GATAD2A, PYCARD, ZC3H12A, INPP5D, PHLDA3, PHLDA1, IL6, ACTC1, MSH2, EDA2R, PEA15A, BIRC3, NIACR1, PPP1R13L, SIRT1, RASSF5, ID1, BAX, CASP12, SULF1, APAF1, DRAM1	2080	473	13588	1.52	1.71E-02	3.66E-04	7.77E-03
GOTERM_BP_FAT	GO:0008219~cell death	116	4.04	5.04E-06	FASTKD1, CADM1, PGLYRP1, TGFB1, TGFB2, SHB, MAP3K5, ANK2, TRP63, FAS, DAP, SGPL1, BCL10, NCF1, BCL2L11, STK3, RELT, SIAH1A, TNFAIP8, RIPK3, MAPK7, TNFAIP3, EIF2AK3, LST1, MCL1, LITAF, TNFRSF12A, ADAMTSL4, CLU, STK17B, ARF6, BCL2L1, IRAK3, PEG10, DOCK1, TCTN3, FGL2, SRGN, TRP53, HTT, KLF11, RYBP, GAS1, NTN1, TNFSF10, BCL2A1A, BBC3, CSRNP1, RHOT2, PDCD7, NUAK2, SGPP1, CXCR2, ASAH2, TMEM173, CASP3, TNFRSF11B, CASP4, PAK2, BAG3, CASP8, MYC, FGF2, DEDD2, IRAK2, LTBR, AIFM1, CYCS, GAN, TOPORS, NLRP3, TNFRSF10B, RIPK1, GADD45G, SH3KBP1, MFSD10, NGFR, SIAH2, GADD45B, PPP1R15A, BID, TNFRSF21, UBE2Z, PML, TRIB3, KIT, FEM1B, CD24A, TNFRSF1A, TNFRSF1B, SQSTM1, COMP, SHISA5, GATAD2A, PYCARD, ZC3H12A, INPP5D, PHLDA3, PHLDA1, IL6, LYZ2, ACTC1, MSH2, EDA2R, PEA15A, BIRC3, NIACR1, PPP1R13L, SIRT1, RASSF5, ID1, BAX, CASP12, SULF1, APAF1, DRAM1	2080	507	13588	1.49	2.06E-02	4.01E-04	9.40E-03

GOTERM_BP_FAT	GO:0016265~death	118	4.11	5.64E-06	FASTKD1, CADM1, PGLYRP1, TGFB1, TGFB2, SHB, MAP3K5, ANK2, TRP63, FAS, DAP, SGPL1, BCL10, NCF1, BCL2L11, STK3, RELT, SIAH1A, TNFAIP8, RIPK3, MAPK7, TNFAIP3, EIF2AK3, LST1, MCL1, LITAF, TNFRSF12A, ADAMTSL4, CLU, STK17B, ARF6, BCL2L1, IRAK3, PEG10, DOCK1, TCTN3, FGL2, SRGN, TRP53, HTT, KLF11, NR4A2, RYBP, GAS1, NTN1, TNFSF10, BCL2A1A, BBC3, CSRNP1, RHOT2, TCF15, PDCD7, NUAK2, SGPP1, CXCR2, ASAH2, TMEM173, CASP3, TNFRSF11B, CASP4, PAK2, BAG3, CASP8, MYC, FGF2, DEDD2, IRAK2, LTBR, AIFM1, CYCS, GAN, TOPORS, NLRP3, TNFRSF10B, RIPK1, GADD45G, SH3KBP1, MFSD10, NGFR, SIAH2, GADD45B, PPP1R15A, BID, TNFRSF21, UBE2Z, PML, TRIB3, KIT, FEM1B, CD24A, TNFRSF1A, TNFRSF1B, SQSTM1, COMP, SHISA5, PYCARD, GATAD2A, ZC3H12A, INPP5D, PHLDA3, PHLDA1, IL6, LYZ2, ACTC1, MSH2, EDA2R, PEA15A, BIRC3, NIACR1, PPP1R13L, SIRT1, RASSF5, ID1, BAX, CASP12, SULF1, APAF1, DRAM1	2080	519	13588	1.49	2.30E-02	4.31E-04	1.05E-02
GOTERM_BP_FAT	GO:0006915~apoptosis	105	3.66	2.65E-05	FASTKD1, CADM1, PGLYRP1, TGFB2, SHB, MAP3K5, ANK2, TRP63, FAS, DAP, SGPL1, BCL10, NCF1, BCL2L11, STK3, SIAH1A, TNFAIP8, RIPK3, MAPK7, TNFAIP3, EIF2AK3, LST1, MCL1, LITAF, TNFRSF12A, ADAMTSL4, STK17B, ARF6, BCL2L1, IRAK3, PEG10, DOCK1, TCTN3, SRGN, TRP53, HTT, KLF11, RYBP, NTN1, TNFSF10, BCL2A1A, BBC3, CSRNP1, RHOT2, PDCD7, NUAK2, SGPP1, CXCR2, ASAH2, TMEM173, CASP3, TNFRSF11B, CASP4, PAK2, BAG3, CASP8, FGF2, MYC, DEDD2, IRAK2, LTBR, AIFM1, CYCS, TOPORS, NLRP3, TNFRSF10B, RIPK1, GADD45G, SH3KBP1, MFSD10, NGFR, SIAH2, GADD45B, PPP1R15A, BID, TNFRSF21, UBE2Z, PML, TRIB3, FEM1B, CD24A, TNFRSF1A, SQSTM1, COMP, SHISA5, PYCARD, ZC3H12A, INPP5D, PHLDA3, PHLDA1, ACTC1, IL6, MSH2, PEA15A, BIRC3, NIACR1, PPP1R13L, SIRT1, RASSF5, ID1, BAX, CASP12, SULF1, APAF1, DRAM1	2080	465	13588	1.48	1.04E-01	1.44E-03	4.93E-02
Annotation Cluster 11	Enrichment Score: 4.01318325859022											
Category	Term	Count	%	PValue	Genes	List Total	Pop Hits	Pop Total	Fold Enrichment	Bonferroni	Benjamini	FDR
GOTERM_MF_FAT	GO:0019899~enzyme binding	61	2.13	7.83E-06	AURKA, DMXL2, DOCK11, DHCR24, ALS2CL, FMNL1, BCL10, IRS2, TWF2, CCDC88A, NCF2, RPH3AL, LDB3, RASIP1, PTPRR, TPX2, PRKCDBP, IRS1, FLNA, NRIP1, CCND1, PLCE1, HIF1A, CLEC12A, CCND2, BTG1, CSTB, TGIF1, RANGRF, PPME1, ADAMTSL4, TRIB3, DAAM1, GCLM, CD24A, IGF1R, RASGRP3, SQSTM1, PSTPIP1, PRIMA1, TES, PARD6A, PTPRC, NOS1, EPAS1, TAF7, DOCK7, DOCK8, PEA15A, COTL1, DOCK6, SIRT1, STAT3, DUSP2, RPS6KA4, CD300A, RAD54L2, MARCKS, SYTL2, TRIP6, DIAP1	2006	229	13288	1.76	1.10E-02	6.16E-04	1.29E-02
Annotation Cluster 12	Enrichment Score: 3.904888428911996											
Category	Term	Count	%	PValue	Genes	List Total	Pop Hits	Pop Total	Fold Enrichment	Bonferroni	Benjamini	FDR
GOTERM_BP_FAT	GO:0042330~taxis	37	1.29	2.91E-06	C3AR1, CCL2, CXCL5, S100A8, CCR1, CXCL3, S100A9, CXCL2, CCL9, CCL8, CXCR2, PF4, ITGB2, KIT, ITGAM, CCL7, CCL6, TGFB2, CCL24, RAC2, SEMA3F, CSF3R, FCER1G, LBP, CYR61, C5AR1, FCGR3, CCL11, CCL12, CORO1A, CXCL13, LYST, ECSCR, CMTM4, CMTM3, PTAFR, CMTM6	2080	109	13588	2.22	1.20E-02	3.08E-04	5.43E-03
GOTERM_BP_FAT	GO:0006935~chemotaxis	37	1.29	2.91E-06	C3AR1, CCL2, CXCL5, S100A8, CCR1, CXCL3, S100A9, CXCL2, CCL9, CCL8, CXCR2, PF4, ITGB2, KIT, ITGAM, CCL7, CCL6, TGFB2, CCL24, RAC2, SEMA3F, CSF3R, FCER1G, LBP, CYR61, C5AR1, FCGR3, CCL11, CCL12, CORO1A, CXCL13, LYST, ECSCR, CMTM4, CMTM3, PTAFR, CMTM6	2080	109	13588	2.22	1.20E-02	3.08E-04	5.43E-03
GOTERM_BP_FAT	GO:0030595~leukocyte chemotaxis	15	0.52	1.03E-05	C5AR1, CXCL3, CCR1, CXCL2, S100A9, ITGB2, PF4, ITGAM, TGFB2, FCGR3, CORO1A, LYST, FCER1G, CSF3R, LBP	2080	27	13588	3.63	4.15E-02	7.19E-04	1.91E-02
GOTERM_BP_FAT	GO:0060326~cell chemotaxis	15	0.52	1.03E-05	C5AR1, CXCL3, CCR1, CXCL2, S100A9, ITGB2, PF4, ITGAM, TGFB2, FCGR3, CORO1A, LYST, FCER1G, CSF3R, LBP	2080	27	13588	3.63	4.15E-02	7.19E-04	1.91E-02
GOTERM_BP_FAT	GO:0050900~leukocyte migration	19	0.66	2.54E-05	SELP, C5AR1, CXCL3, CCR1, ELANE, CXCL2, S100A9, ITGB2, PF4, CD24A, ITGAM, FCGR3, TGFB2, CORO1A, LYST, FCER1G, CSF3R, LBP, SELPLG	2080	43	13588	2.89	9.96E-02	1.40E-03	4.74E-02
Annotation Cluster 13	Enrichment Score: 3.8758691195212593											
Category	Term	Count	%	PValue	Genes	List Total	Pop Hits	Pop Total	Fold Enrichment	Bonferroni	Benjamini	FDR
GOTERM_BP_FAT	GO:0006796~phosphate metabolic process	186	6.48	5.68E-07	RNASEL, PDP2, EFNA1, TBK1, SNCA, PTPN23, TGFB3, AURKA, LPAR1, PRKG1, C230081A13RIK, PRKX, TGFB1, TGFB2, BTK, MAP3K6, MAP3K5, MAP3K8, MLKL, STK39, MAP2K6, PTPRJ, EGFR, RET, BCR, PTPRF, ROCK2, MYLK4, PTPRR, PIM1, PTPRS, RPS6KC1, WNK2, STK3, MAP4K4, PLCE1, MAPK4, ACAP1, SLC37A2, DLD, BMP2K, PDGFRA, ATP5C1, RIPK3, MAPK7, CSNK1G3, EIF2AK3, LRRK1, EIF2AK4, WNT5A, FGFR3, FGR, SSH3, MAP4K2, DUSP10, STK17B, ACP5, CHEK2, ATP6V1B2, PXN, CD74, IRAK4, IRAK3, MUSK, DUSP13, PTPLB, CSF1R, TEC, FGD4, PTPN7, PTPN6, PTPN9, PTPN3, PTPN2, TGFBR1, MET, TRIO, DUSP22, PTPN12, EPHA2, IKBKE, PLK3, PLK2, DUSP27, PRKAR1B, NTRK2, PRKAR1A, TNK2, PTPN1, GRK5, ABL2, ATP5D, CDK17, UQCRC1, CDK18, ACVRL1, NUAK2, CCNT1, CAD, FES, DDR2, CAMKK1, CAMKK2, CCNE1, ADCK1, PAK2, PAK4, EEF2K, CDK12, SYNJ2, ATP5L, ATP5O, TIE1, BRD4, CSK, SIK1, ATP5H, FGF2, CDK14, ATP5K, ATP5J, IRAK2, SRPK2, LYN, PIK3C2B, NDUFC2, PRKCI, ATP6V1H, PKDCC, PRKCD, MTMR12, MAST4, ATP6V1A, CCND1, PPM1K, UQCRH, RIPK1, MTMR10, GADD45G, CFL1, GADD45B, MAP3K14, NDUFB8, PML, TRIB3, ABI1, ATP5G1, KIT, ATP5G3, TRIB1, IGF1R, STK40, SNRK, PTK2B, MAP3K2, PPP1R12A, DCLK1, TES, AMHR2, CSNK1A1, PTPRB, PTPRC, PTPRE, NCEH1, MSH2, ALPK2, HCK, PDK4, AXL, ATP5F1, MAPK11, DUSP5, RPS6KA5, DUSP4, RPS6KA3, DUSP2, RPS6KA4, GCK, DUSP1, PTP4A3, JAK1, GYK, IPPK, BMPR1B, IGFBP3, DUSP7	2080	866	13588	1.40	2.34E-03	9.38E-05	1.06E-03
GOTERM_BP_FAT	GO:0006793~phosphorus metabolic process	186	6.48	5.68E-07	RNASEL, PDP2, EFNA1, TBK1, SNCA, PTPN23, TGFB3, AURKA, LPAR1, PRKG1, C230081A13RIK, PRKX, TGFB1, TGFB2, BTK, MAP3K6, MAP3K5, MAP3K8, MLKL, STK39, MAP2K6, PTPRJ, EGFR, RET, BCR, PTPRF, ROCK2, MYLK4, PTPRR, PIM1, PTPRS, RPS6KC1, WNK2, STK3, MAP4K4, PLCE1, MAPK4, ACAP1, SLC37A2, DLD, BMP2K, PDGFRA, ATP5C1, RIPK3, MAPK7, CSNK1G3, EIF2AK3, LRRK1, EIF2AK4, WNT5A, FGFR3, FGR, SSH3, MAP4K2, DUSP10, STK17B, ACP5, CHEK2, ATP6V1B2, PXN, CD74, IRAK4, IRAK3, MUSK, DUSP13, PTPLB, CSF1R, TEC, FGD4, PTPN7, PTPN6, PTPN9, PTPN3, PTPN2, TGFBR1, MET, TRIO, DUSP22, PTPN12, EPHA2, IKBKE, PLK3, PLK2, DUSP27, PRKAR1B, NTRK2, PRKAR1A, TNK2, PTPN1, GRK5, ABL2, ATP5D, CDK17, UQCRC1, CDK18, ACVRL1, NUAK2, CCNT1, CAD, FES, DDR2, CAMKK1, CAMKK2, CCNE1, ADCK1, PAK2, PAK4, EEF2K, CDK12, SYNJ2, ATP5L, ATP5O, TIE1, BRD4, CSK, SIK1, ATP5H, FGF2, CDK14, ATP5K, ATP5J, IRAK2, SRPK2, LYN, PIK3C2B, NDUFC2, PRKCI, ATP6V1H, PKDCC, PRKCD, MTMR12, MAST4, ATP6V1A, CCND1, PPM1K, UQCRH, RIPK1, MTMR10, GADD45G, CFL1, GADD45B, MAP3K14, NDUFB8, PML, TRIB3, ABI1, ATP5G1, KIT, ATP5G3, TRIB1, IGF1R, STK40, SNRK, PTK2B, MAP3K2, PPP1R12A, DCLK1, TES, AMHR2, CSNK1A1, PTPRB, PTPRC, PTPRE, NCEH1, MSH2, ALPK2, HCK, PDK4, AXL, ATP5F1, MAPK11, DUSP5, RPS6KA5, DUSP4, RPS6KA3, DUSP2, RPS6KA4, GCK, DUSP1, PTP4A3, JAK1, GYK, IPPK, BMPR1B, IGFBP3, DUSP7	2080	866	13588	1.40	2.34E-03	9.38E-05	1.06E-03
GOTERM_BP_FAT	GO:0016310~phosphorylation	151	5.26	2.41E-05	RNASEL, EFNA1, TBK1, SNCA, TGFB3, AURKA, LPAR1, PRKG1, C230081A13RIK, TGFB1, PRKX, TGFB2, BTK, MAP3K6, MAP3K5, MAP3K8, MLKL, STK39, MAP2K6, EGFR, RET, BCR, ROCK2, MYLK4, PIM1, RPS6KC1, WNK2, STK3, MAP4K4, PLCE1, MAPK4, ACAP1, DLD, BMP2K, PDGFRA, ATP5C1, RIPK3, MAPK7, CSNK1G3, EIF2AK3, LRRK1, EIF2AK4, WNT5A, FGFR3, FGR, MAP4K2, STK17B, CHEK2, ATP6V1B2, CD74, PXN, IRAK4, IRAK3, MUSK, CSF1R, TEC, FGD4, TGFBR1, MET, TRIO, EPHA2, IKBKE, PLK3, PLK2, PRKAR1B, NTRK2, PRKAR1A, TNK2, GRK5, ABL2, ATP5D, CDK17, CDK18, ACVRL1, UQCRC1, NUAK2, CCNT1, CAD, FES, DDR2, CAMKK1, CAMKK2, CCNE1, ADCK1, PAK2, PAK4, EEF2K, CDK12, ATP5L, ATP5O, TIE1, BRD4, SIK1, CSK, ATP5H, FGF2, ATP5K, CDK14, ATP5J, IRAK2, SRPK2, LYN, PIK3C2B, NDUFC2, PRKCI, ATP6V1H, PKDCC, PRKCD, ATP6V1A, MAST4, CCND1, UQCRH, RIPK1, GADD45G, CFL1, GADD45B, MAP3K14, NDUFB8, PML, TRIB3, ABI1, ATP5G1, KIT, ATP5G3, TRIB1, IGF1R, STK40, SNRK, PTK2B, MAP3K2, DCLK1, TES, AMHR2, CSNK1A1, PTPRC, PTPRE, MSH2, ALPK2, HCK, PDK4, AXL, ATP5F1, MAPK11, RPS6KA5, RPS6KA3, RPS6KA4, GCK, JAK1, BMPR1B, IPPK, IGFBP3	2080	718	13588	1.37	9.48E-02	1.36E-03	4.49E-02
Annotation Cluster 14	Enrichment Score: 3.853375180161527											
Category	Term	Count	%	PValue	Genes	List Total	Pop Hits	Pop Total	Fold Enrichment	Bonferroni	Benjamini	FDR
GOTERM_BP_FAT	GO:0006732~coenzyme metabolic process	45	1.57	2.15E-06	GCLC, HMGCR, EHHADH, G6PDX, PGD, ACOT2, ALDH1L2, ACSS2, GCLM, MTHFD1L, PDSS2, GCH1, GSR, MTHFD2, NUDT7, IDH1, FH1, MOCS1, DLST, NCF2, NCF1, ALDH5A1, SUCLG1, PDK4, GSTT1, GSTT2, ACLY, COQ9, DLAT, COQ7, COQ5, IDH3A, PANK4, DBT, SDHB, GCK, MLYCD, PANK1, SDHC, SDHD, SLC27A3, MDH2, MGST1, NMNAT1, MDH1	2080	143	13588	2.06	8.84E-03	2.54E-04	4.01E-03
GOTERM_BP_FAT	GO:0051186~cofactor metabolic process	51	1.78	1.62E-05	HMGCR, COX10, EHHADH, G6PDX, PGD, ACOT2, ACSS2, ALDH1L2, MTHFD1L, GSR, MTHFD2, ALAS1, ANK1, ALAS2, HMOX1, NCF2, ALDH5A1, NCF1, SUCLG1, GSTT1, COQ9, GSTT2, COQ7, COQ5, PANK4, PANK1, SLC27A3, MDH2, NMNAT1, MDH1, GCLC, GCLM, PDSS2, GCH1, NUDT7, IDH1, FH1, MOCS1, DLST, PDK4, ACLY, DLAT, IDH3A, SDHB, DBT, GCK, MLYCD, SDHC, SDHD, NARFL, MGST1	2080	182	13588	1.83	6.46E-02	1.01E-03	3.02E-02
Annotation Cluster 15	Enrichment Score: 3.748663051430174											
Category	Term	Count	%	PValue	Genes	List Total	Pop Hits	Pop Total	Fold Enrichment	Bonferroni	Benjamini	FDR
GOTERM_BP_FAT	GO:0031328~positive regulation of cellular biosynthetic process	128	4.46	7.37E-07	MYOD1, THRB, TBK1, TLR1, TGFB3, TLR2, TLR4, TGFB1, CITED2, KDM1A, MYD88, TRP63, RARA, RARB, BCL10, ICOSL, RELA, SIX2, ARNTL, SIX4, MED13, PPARGC1B, PTHLH, HOXD9, HIF1A, ZFP462, JUN, VEGFA, SIX1, VGLL2, NFE2L2, FUS, HMGB2, ADORA2B, CYTL1, SOX4, SFPI1, ITGB2, SOX9, PLAGL1, SLC11A1, TEAD4, NPM1, AGRN, RUNX1, RUNX2, TRP53, BMP4, KLF5, KLF6, BMP2, ESRRA, EPAS1, IKZF1, MAFB, MAML1, SMAD5, CREBBP, NR4A2, ESRRG, NR4A1, SMAD3, TEAD3, KLF15, SMAD1, NR4A3, FCGR3, SREBF2, ATF4, SALL4, EAF1, CSRNP1, KLF2, KLF4, PPARA, CHURC1, FOXK1, ELF4, FOS, SDPR, GUCY1A3, YAP1, MYC, FGF2, RHOG, SERTAD1, EGR1, AR, EGR2, HSP90AA1, TOPORS, HMGA1, SMO, MURC, CAND1, FOXC1, EIF5A2, SERP1, ING5, ABLIM2, GLIS2, ABLIM3, NUFIP1, PRDM16, TNFRSF1A, REL, AGT, NFAT5, BCL3, MYOG, PTX3, CEBPA, BCKDHA, MYF6, IL6, CEBPB, CEBPD, TBX1, AFF1, STAT3, GCK, IRF8, IRF1, PBX1, RBM14, RBM15, NFIA, ARAP1	2080	552	13588	1.51	3.04E-03	1.13E-04	1.37E-03
GOTERM_BP_FAT	GO:0009891~positive regulation of biosynthetic process	128	4.46	1.21E-06	MYOD1, THRB, TBK1, TLR1, TGFB3, TLR2, TLR4, TGFB1, CITED2, KDM1A, MYD88, TRP63, RARA, RARB, BCL10, ICOSL, RELA, SIX2, ARNTL, SIX4, MED13, PPARGC1B, PTHLH, HOXD9, HIF1A, ZFP462, JUN, VEGFA, SIX1, VGLL2, NFE2L2, FUS, HMGB2, ADORA2B, CYTL1, SOX4, SFPI1, ITGB2, SOX9, PLAGL1, SLC11A1, TEAD4, NPM1, AGRN, RUNX1, RUNX2, TRP53, BMP4, KLF5, KLF6, BMP2, ESRRA, EPAS1, IKZF1, MAFB, MAML1, SMAD5, CREBBP, NR4A2, ESRRG, NR4A1, SMAD3, TEAD3, KLF15, SMAD1, NR4A3, FCGR3, SREBF2, ATF4, SALL4, EAF1, CSRNP1, KLF2, KLF4, PPARA, CHURC1, FOXK1, ELF4, FOS, SDPR, GUCY1A3, YAP1, MYC, FGF2, RHOG, SERTAD1, EGR1, AR, EGR2, HSP90AA1, TOPORS, HMGA1, SMO, MURC, CAND1, FOXC1, EIF5A2, SERP1, ING5, ABLIM2, GLIS2, ABLIM3, NUFIP1, PRDM16, TNFRSF1A, REL, AGT, NFAT5, BCL3, MYOG, PTX3, CEBPA, BCKDHA, MYF6, IL6, CEBPB, CEBPD, TBX1, AFF1, STAT3, GCK, IRF8, IRF1, PBX1, RBM14, RBM15, NFIA, ARAP1	2080	557	13588	1.50	4.99E-03	1.67E-04	2.26E-03
GOTERM_BP_FAT	GO:0045893~positive regulation of transcription, DNA-dependent	101	3.52	1.46E-06	MYOD1, THRB, TGFB3, TGFB1, CITED2, KDM1A, MYD88, TRP63, RARA, RARB, BCL10, RELA, SIX2, SIX4, MED13, ARNTL, PPARGC1B, HOXD9, HIF1A, ZFP462, JUN, VEGFA, SIX1, VGLL2, NFE2L2, FUS, HMGB2, CYTL1, SOX4, SFPI1, SOX9, PLAGL1, SLC11A1, TEAD4, AGRN, RUNX1, RUNX2, TRP53, BMP4, KLF6, ESRRA, BMP2, IKZF1, EPAS1, MAFB, MAML1, SMAD5, CREBBP, NR4A2, ESRRG, NR4A1, SMAD3, NR4A3, SMAD1, KLF15, TEAD3, SREBF2, ATF4, SALL4, EAF1, CSRNP1, KLF4, PPARA, CHURC1, FOXK1, ELF4, FOS, SDPR, YAP1, FGF2, MYC, EGR1, AR, EGR2, MURC, SMO, FOXC1, CAND1, ABLIM2, ABLIM3, NUFIP1, PRDM16, TNFRSF1A, REL, NFAT5, BCL3, MYOG, CEBPA, BCKDHA, IL6, CEBPB, CEBPD, TBX1, AFF1, STAT3, IRF8, IRF1, PBX1, RBM14, RBM15, NFIA	2080	416	13588	1.59	6.02E-03	1.83E-04	2.73E-03
GOTERM_BP_FAT	GO:0051254~positive regulation of RNA metabolic process	101	3.52	2.06E-06	MYOD1, THRB, TGFB3, TGFB1, CITED2, KDM1A, MYD88, TRP63, RARA, RARB, BCL10, RELA, SIX2, SIX4, MED13, ARNTL, PPARGC1B, HOXD9, HIF1A, ZFP462, JUN, VEGFA, SIX1, VGLL2, NFE2L2, FUS, HMGB2, CYTL1, SOX4, SFPI1, SOX9, PLAGL1, SLC11A1, TEAD4, AGRN, RUNX1, RUNX2, TRP53, BMP4, KLF6, ESRRA, BMP2, IKZF1, EPAS1, MAFB, MAML1, SMAD5, CREBBP, NR4A2, ESRRG, NR4A1, SMAD3, NR4A3, SMAD1, KLF15, TEAD3, SREBF2, ATF4, SALL4, EAF1, CSRNP1, KLF4, PPARA, CHURC1, FOXK1, ELF4, FOS, SDPR, YAP1, FGF2, MYC, EGR1, AR, EGR2, MURC, SMO, FOXC1, CAND1, ABLIM2, ABLIM3, NUFIP1, PRDM16, TNFRSF1A, REL, NFAT5, BCL3, MYOG, CEBPA, BCKDHA, IL6, CEBPB, CEBPD, TBX1, AFF1, STAT3, IRF8, IRF1, PBX1, RBM14, RBM15, NFIA	2080	419	13588	1.57	8.48E-03	2.50E-04	3.84E-03

GOTERM_BP_FAT	GO:0051173~positive regulation of nitrogen compound metabolic process	121	4.22	2.33E-06	MYOD1, THRB, TGFB3, TLR2, TLR4, PNP, TGFB1, CITED2, KDM1A, MYD88, TRP63, RARA, RARB, BCL10, RELA, SIX2, ARNTL, SIX4, MED13, PPARGC1B, PTHLH, HOXD9, HIF1A, ZFP462, JUN, VEGFA, SIX1, VGLL2, NFE2L2, FUS, HMGB2, ADORA2B, CYTL1, SOX4, ITGB2, SFPI1, SOX9, PLAGL1, SLC11A1, TEAD4, AGRN, RUNX1, RUNX2, TRP53, BMP4, KLF5, KLF6, BMP2, ESRRA, EPAS1, IKZF1, MAFB, MAML1, SMAD5, CREBBP, NR4A2, ESRRG, NR4A1, SMAD3, SMAD1, NR4A3, KLF15, TEAD3, SREBF2, ATF4, SALL4, EAF1, CSRNP1, KLF2, KLF4, PPARA, CHURC1, FOXK1, ELF4, FOS, SDPR, GUCY1A3, YAP1, MYC, FGF2, RHOG, SERTAD1, EGR1, AR, EGR2, HSP90AA1, TOPORS, HMGA1, SMO, MURC, CAND1, FOXC1, ING5, ABLIM2, GLIS2, ABLIM3, NUFIP1, PRDM16, TNFRSF1A, REL, NFAT5, BCL3, MYOG, PTX3, CEBPA, BCKDHA, MYF6, PTPRC, IL6, CEBPB, CEBPD, TBX1, AFF1, STAT3, IRF8, IRF1, PBX1, RBM14, RBM15, NFIA, ARAP1	2080	526	13588	1.50	9.57E-03	2.67E-04	4.34E-03
GOTERM_BP_FAT	GO:0045944~positive regulation of transcription from RNA polymerase II promoter	89	3.10	2.36E-06	MYOD1, PPARA, CHURC1, THRB, ELF4, TGFB3, TGFB1, CITED2, KDM1A, FOS, MYD88, SDPR, TRP63, RARA, YAP1, RARB, FGF2, MYC, EGR1, AR, EGR2, SIX2, ARNTL, SIX4, MED13, PPARGC1B, HOXD9, SMO, MURC, HIF1A, ZFP462, JUN, VEGFA, SIX1, VGLL2, CAND1, FOXC1, FUS, ABLIM2, HMGB2, ABLIM3, CYTL1, SOX4, NUFIP1, SOX9, PLAGL1, SLC11A1, TNFRSF1A, TEAD4, NFAT5, BCL3, MYOG, AGRN, RUNX1, RUNX2, BMP4, CEBPA, TRP53, KLF6, BMP2, ESRRA, IL6, CEBPB, IKZF1, EPAS1, MAFB, CEBPD, MAML1, CREBBP, SMAD5, NR4A2, SMAD3, NR4A1, TBX1, NR4A3, SMAD1, TEAD3, STAT3, SREBF2, ATF4, SALL4, EAF1, CSRNP1, IRF1, PBX1, RBM14, RBM15, NFIA, KLF4	2080	358	13588	1.62	9.72E-03	2.64E-04	4.41E-03
GOTERM_BP_FAT	GO:0045941~positive regulation of transcription	111	3.87	2.96E-06	MYOD1, THRB, TGFB3, TGFB1, CITED2, KDM1A, MYD88, TRP63, RARA, RARB, BCL10, RELA, SIX2, ARNTL, SIX4, MED13, PPARGC1B, HOXD9, HIF1A, ZFP462, JUN, VEGFA, SIX1, VGLL2, NFE2L2, FUS, HMGB2, CYTL1, SOX4, SFPI1, SOX9, PLAGL1, SLC11A1, TEAD4, AGRN, RUNX1, RUNX2, TRP53, BMP4, KLF5, KLF6, ESRRA, BMP2, EPAS1, IKZF1, MAFB, MAML1, SMAD5, CREBBP, NR4A2, ESRRG, NR4A1, SMAD3, SMAD1, NR4A3, KLF15, TEAD3, SREBF2, ATF4, SALL4, EAF1, CSRNP1, KLF2, KLF4, PPARA, CHURC1, FOXK1, ELF4, FOS, SDPR, YAP1, FGF2, MYC, RHOG, SERTAD1, EGR1, AR, EGR2, TOPORS, HMGA1, SMO, MURC, CAND1, FOXC1, ING5, ABLIM2, ABLIM3, GLIS2, NUFIP1, PRDM16, TNFRSF1A, REL, NFAT5, BCL3, MYOG, CEBPA, BCKDHA, MYF6, IL6, CEBPB, CEBPD, TBX1, AFF1, STAT3, IRF8, IRF1, PBX1, RBM14, RBM15, NFIA, ARAP1	2080	475	13588	1.53	1.21E-02	2.98E-04	5.52E-03
GOTERM_BP_FAT	GO:0010628~positive regulation of gene expression	113	3.94	3.77E-06	MYOD1, THRB, TGFB3, TGFB1, CITED2, KDM1A, MYD88, TRP63, RARA, RARB, BCL10, RELA, SIX2, ARNTL, SIX4, MED13, PPARGC1B, HOXD9, HIF1A, ZFP462, JUN, VEGFA, SIX1, VGLL2, NFE2L2, FUS, HMGB2, CYTL1, SOX4, SFPI1, SOX9, PLAGL1, SLC11A1, TEAD4, AGRN, RUNX1, RUNX2, TRP53, BMP4, KLF5, KLF6, ESRRA, BMP2, EPAS1, IKZF1, MAFB, MAML1, SMAD5, CREBBP, NR4A2, ESRRG, NR4A1, SMAD3, SMAD1, NR4A3, KLF15, TEAD3, SREBF2, ATF4, SALL4, EAF1, CSRNP1, KLF2, KLF4, PPARA, CHURC1, FOXK1, ELF4, FOS, SDPR, YAP1, MYC, FGF2, RHOG, SERTAD1, EGR1, AR, EGR2, TOPORS, HMGA1, SMO, MURC, CAND1, FOXC1, ING5, ABLIM2, LRRC32, ABLIM3, GLIS2, NUFIP1, KIT, PRDM16, TNFRSF1A, REL, NFAT5, BCL3, MYOG, CEBPA, BCKDHA, MYF6, IL6, CEBPB, CEBPD, TBX1, AFF1, STAT3, IRF8, IRF1, PBX1, RBM14, RBM15, NFIA, ARAP1	2080	488	13588	1.51	1.54E-02	3.38E-04	7.02E-03
GOTERM_BP_FAT	GO:0006357~regulation of transcription from RNA polymerase II promoter	136	4.74	5.27E-06	MYOD1, THRB, TGFB3, ANKRD1, TGFB1, CITED2, KDM1A, MYD88, TRP63, RARA, RARB, SIX2, MED11, ARNTL, SIX4, MED13, PPARGC1B, HOXD9, HOXD8, HIF1A, MED15, ZFP462, JUN, VEGFA, SIX1, MNDA, VGLL2, TGIF1, FUS, HMGB2, CYTL1, SOX4, ABCA2, TXN1, SOX9, MEIS1, PLAGL1, SLC11A1, HEXIM1, TEAD4, AGRN, RUNX1, RUNX2, TRP53, BMP4, KLF6, BMP2, ESRRA, EPAS1, IKZF1, MAFB, MAML1, SMAD5, CREBBP, LMCD1, NR4A2, RYBP, KLF16, NR4A1, SMAD3, TEAD3, SMAD1, NR4A3, MED13L, FOXP4, SREBF2, ZBTB42, ATF4, SALL4, EAF1, RNF2, CSRNP1, JAZF1, RBPJ, HDAC9, KLF4, PPARA, CHURC1, PPARD, E2F4, FOXK1, ELF4, NR6A1, FOS, SAP30, LBH, SDPR, YAP1, MYC, FGF2, EGR1, AR, EGR2, TLE4, NRIP1, SMO, IFNAR2, MURC, BPTF, CAND1, FOXC1, KDM6B, ABLIM2, ZFP395, GLIS2, ABLIM3, FHL2, NUFIP1, PRDM16, TNFRSF1A, SORBS3, NFAT5, GM608, PER1, BCL3, BCL6, MYOG, PER3, SIM2, ETV3, CEBPA, IL6, CEBPB, CEBPD, TBX1, SIRT1, STAT3, ID2, ID1, HEYL, IRF1, PBX1, RBM14, RBM15, NFIA, IFI204	2080	616	13588	1.44	2.15E-02	4.10E-04	9.82E-03
GOTERM_BP_FAT	GO:0045935~positive regulation of nucleobase, nucleoside, nucleotide and nucleic acid metabolic proc	116	4.04	6.74E-06	MYOD1, THRB, TGFB3, PNP, TGFB1, CITED2, KDM1A, MYD88, TRP63, RARA, RARB, BCL10, RELA, SIX2, ARNTL, SIX4, MED13, PPARGC1B, PTHLH, HOXD9, HIF1A, ZFP462, JUN, VEGFA, SIX1, VGLL2, NFE2L2, FUS, HMGB2, ADORA2B, CYTL1, SOX4, SFPI1, SOX9, PLAGL1, SLC11A1, TEAD4, AGRN, RUNX1, RUNX2, TRP53, BMP4, KLF5, KLF6, ESRRA, BMP2, EPAS1, IKZF1, MAFB, MAML1, SMAD5, CREBBP, NR4A2, ESRRG, NR4A1, SMAD3, SMAD1, NR4A3, KLF15, TEAD3, SREBF2, ATF4, SALL4, EAF1, CSRNP1, KLF2, KLF4, PPARA, CHURC1, FOXK1, ELF4, FOS, SDPR, GUCY1A3, YAP1, MYC, FGF2, RHOG, SERTAD1, EGR1, AR, EGR2, TOPORS, HMGA1, SMO, MURC, CAND1, FOXC1, ING5, ABLIM2, GLIS2, ABLIM3, NUFIP1, PRDM16, TNFRSF1A, REL, NFAT5, BCL3, MYOG, CEBPA, BCKDHA, MYF6, PTPRC, IL6, CEBPB, CEBPD, TBX1, AFF1, STAT3, IRF8, IRF1, PBX1, RBM14, RBM15, NFIA, ARAP1	2080	510	13588	1.49	2.74E-02	5.06E-04	1.26E-02
GOTERM_BP_FAT	GO:0010557~positive regulation of macromolecule biosynthetic process	119	4.15	9.65E-06	MYOD1, THRB, TBK1, TLR1, TGFB3, TLR4, TGFB1, CITED2, KDM1A, MYD88, TRP63, RARA, RARB, BCL10, ICOSL, RELA, SIX2, ARNTL, SIX4, MED13, PPARGC1B, HOXD9, HIF1A, ZFP462, JUN, VEGFA, SIX1, VGLL2, NFE2L2, FUS, HMGB2, CYTL1, SOX4, SFPI1, SOX9, PLAGL1, SLC11A1, TEAD4, AGRN, RUNX1, RUNX2, TRP53, BMP4, KLF5, KLF6, ESRRA, BMP2, EPAS1, IKZF1, MAFB, MAML1, SMAD5, CREBBP, NR4A2, ESRRG, NR4A1, SMAD3, SMAD1, NR4A3, KLF15, TEAD3, FCGR3, SREBF2, ATF4, SALL4, EAF1, CSRNP1, KLF2, KLF4, PPARA, CHURC1, FOXK1, ELF4, FOS, SDPR, YAP1, MYC, FGF2, RHOG, SERTAD1, EGR1, AR, EGR2, TOPORS, HMGA1, SMO, MURC, CAND1, FOXC1, EIF5A2, SERP1, ING5, ABLIM2, GLIS2, ABLIM3, NUFIP1, PRDM16, TNFRSF1A, REL, NFAT5, BCL3, MYOG, CEBPA, BCKDHA, MYF6, IL6, CEBPB, CEBPD, TBX1, AFF1, STAT3, GCK, IRF8, IRF1, PBX1, RBM14, RBM15, NFIA, ARAP1	2080	530	13588	1.47	3.91E-02	6.87E-04	1.80E-02

Table S1.C GO Terms upregulated 24 hours post injury

Annotation Cluster 1 Enrichment Score: 12.9139933966702

Category	Term	Count	%	PValue	Genes	List Total	Pop Hits	Pop Total	Fold Enrichment	Bonferroni	Benjamini	FDR
GOTERM_BP_FAT	GO:0009611~response to wounding	112	3.92	2.91E-16	GNA13, F2RL3, F13A1, TLR1, TLR2, TLR4, TLR6, TLR7, C1QC, TGFB1, TLR8, TLR9, CFP, MYD88, DYSF, CD44, IL1B, CFD, TFPI2, RAB27A, F10, NCF1, C4B, PLAUR, C1QA, C1QB, HIF1A, THBD, UNC13D, CCR5, LYST, CCR2, CCL3, CCL2, NFKBID, NINJ1, CCL8, ITGB2, ELK3, CCL7, ARG1, SLC11A1, MTPN, CNR2, ENTPD1, FN1, PTPN6, KLF6, BMP2, EFEMP2, TLR13, SPHK1, CHI3L3, FCGR1, FCGR3, NOTCH2, P2RX7, SLC7A2, CLEC7A, LY86, PPARG, HPS1, GJA1, NOD2, CASP3, SAA1, TICAM1, TICAM2, NFKBIZ, LY96, SAA3, GAL, NLRP3, CHST1, SERPINA3N, OTOG, PLA2G7, NPPB, NGFR, CTSB, PROS1, LCP1, PTAFR, CXCL1, CXCL3, CCR1, PF4, ABHD2, TNFRSF4, CCL24, CCL25, TNFRSF1A, TNFRSF1B, MEFV, BCL2, PYCARD, C2, THBS1, PAPSS2, SELP, IL6, LIPA, PLEK, ANXA5, STAT3, CD180, CCL12, STAB1, BAX, HBEGF, ALOX5, CD14	2019	347	13588	2.17	1.33E-12	6.67E-13	6.22E-13
GOTERM_BP_FAT	GO:0006954~inflammatory response	77	2.70	6.90E-13	LY86, TLR1, PPARG, TLR2, TLR4, TLR6, C1QC, TLR7, TLR8, TGFB1, TLR9, CFP, NOD2, MYD88, CD44, SAA1, TICAM1, TICAM2, IL1B, CFD, NFKBIZ, C4B, NCF1, LY96, SAA3, GAL, NLRP3, CHST1, C1QA, C1QB, SERPINA3N, UNC13D, HIF1A, CCR5, CCR2, PLA2G7, NPPB, PTAFR, CXCL1, CCL3, CCL2, NFKBID, CCR1, CXCL3, CCL8, ITGB2, TNFRSF4, CCL7, CCL24, TNFRSF1A, SLC11A1, CCL25, TNFRSF1B, MEFV, CNR2, PYCARD, C2, THBS1, FN1, SELP, IL6, BMP2, LIPA, SPHK1, TLR13, CHI3L3, FCGR1, STAT3, CD180, FCGR3, CCL12, P2RX7, STAB1, SLC7A2, ALOX5, CLEC7A, CD14	2019	225	13588	2.30	2.76E-09	5.53E-10	1.28E-09
GOTERM_BP_FAT	GO:0006952~defense response	122	4.27	9.03E-12	TLR1, TLR2, TLR4, TLR6, TLR7, C1QC, TGFB1, TLR8, TLR9, B2M, CFP, MYD88, CD44, IL1B, MX1, CFD, MX2, RAB27A, BCL10, C5AR1, NCF1, C4B, RELA, IFI47, C1QA, C1QB, HIF1A, UNC13D, CCR5, LYST, CCR2, IFIH1, IL1R1, CCL3, CCL2, NFKBID, FGR, CCL8, ITGB2, SP110, CD74, CCL7, SLC11A1, CNR2, FCER1G, DHX58, FN1, PTPN6, BMP2, CARD9, IL1RL1, TLR13, SPHK1, MYO1F, CHI3L3, VDAC3, FCGR1, FCGR3, VDAC1, DDX58, P2RX7, SLC7A2, CLEC7A, RBPJ, LY86, PPARG, H2-D1, BNIP3, TMEM173, NOD2, SAA1, TICAM1, IL1RAP, TICAM2, NFKBIZ, IL18RAP, LY96, CNPY3, SAA3, CLIC1, GAL, NLRP3, CHST1, SERPINA3N, PLA2G7, NPPB, H2-AA, PTAFR, CXCL1, CXCL3, CCR1, RSAD2, TNFRSF4, CCL24, CCL25, TNFRSF1A, TNFRSF1B, MEFV, BCL2, TAP1, PYCARD, BCL3, C2, THBS1, PTPRC, SELP, IL6, LYZ2, LIPA, H2-M3, HCK, SAMHD1, COTL1, STAT3, CD180, CCL12, FCGR2B, STAB1, IRF8, APAF1, ALOX5, CD14	2019	448	13588	1.83	3.62E-08	4.02E-09	1.68E-08

Annotation Cluster 2 Enrichment Score: 10.70304093875696

Category	Term	Count	%	PValue	Genes	List Total	Pop Hits	Pop Total	Fold Enrichment	Bonferroni	Benjamini	FDR
GOTERM_BP_FAT	GO:0019318~hexose metabolic process	63	2.21	1.59E-12	PRKAG3, LDHA, GNPDA1, PHKB, G6PDX, PGD, PGAM1, PGAM2, ACN9, MAN2B1, MYC, B3GALTL, PHKG1, PFKM, FBP2, PPP1R3A, PGM2L1, PGM2, PGM3, MAN2A1, RENBP, PGM1, GM12070, SERP1, MDH1, PFKFB3, PFKFB1, SLC37A4, PHKA1, HK1, GPI1, GALK2, GALK1, TPI1, PPP1R3C, PPP1R3B, HK3, ENO2, ENO3, GALE, PRKAA1, AGL, ENO1, GPD2, ALDOART1, PDK2, GPD1, WDTC1, GMDS, PDK3, PDK4, BPGM, MAN1C1, ADIPOQ, CPT1A, PCX, MPI, ATF3, PYGM, PYGL, PGK1, FABP5, PYGB	2019	169	13588	2.51	6.35E-09	1.06E-09	2.95E-09
GOTERM_BP_FAT	GO:0005996~monosaccharide metabolic process	68	2.38	2.25E-12	PRKAG3, LDHA, GNPDA1, PHKB, G6PDX, PGD, PGAM1, PGAM2, ACN9, UGT1A7C, MAN2B1, MYC, B3GALTL, PHKG1, PFKM, PPP1R3A, FBP2, PGM2L1, PGM2, PGM3, MAN2A1, RENBP, PGM1, OTOG, GM12070, SERP1, MDH1, PFKFB3, PFKFB1, SLC37A4, PHKA1, FN3K, HK1, GPI1, GALK2, GALK1, TPI1, PPP1R3C, PPP1R3B, HK3, ENO2, ENO3, GALE, PRKAA1, AGL, ENO1, GPD2, ALDOART1, PDK2, GPD1, WDTC1, GMDS, PDK3, PDK4, BPGM, MAN1C1, ADIPOQ, CPT1A, CSGALNACT1, AMDHD2, PCX, MPI, ATF3, PYGM, PYGL, PGK1, FABP5, PYGB	2019	191	13588	2.40	8.99E-09	1.28E-09	4.17E-09
GOTERM_BP_FAT	GO:0006006~glucose metabolic process	50	1.75	2.18E-09	PRKAG3, LDHA, PHKB, G6PDX, PGD, PGAM1, PGAM2, ACN9, MYC, PHKG1, PFKM, FBP2, PPP1R3A, PGM2L1, PGM2, PGM3, PGM1, GM12070, SERP1, MDH1, PHKA1, SLC37A4, HK1, GPI1, TPI1, PPP1R3C, PPP1R3B, HK3, ENO2, ENO3, PRKAA1, AGL, ENO1, GPD2, ALDOART1, PDK2, GPD1, WDTC1, PDK3, PDK4, BPGM, ADIPOQ, CPT1A, PCX, ATF3, PYGM, PYGL, PGK1, FABP5, PYGB	2019	140	13588	2.40	8.75E-06	5.83E-07	4.06E-06

Annotation Cluster 3 Enrichment Score: 8.960616277409079

Category	Term	Count	%	PValue	Genes	List Total	Pop Hits	Pop Total	Fold Enrichment	Bonferroni	Benjamini	FDR
GOTERM_BP_FAT	GO:0030029~actin filament-based process	63	2.21	1.21E-11	ENAH, TLN1, LIMA1, NUAK2, AIF1, CAPZA2, S100A9, WASF2, CAPZA1, PDLIM3, SHC1, CAP1, RHOG, PLS3, FMNL1, DBNL, ARHGEF2, FMNL3, ACTN4, PRKCI, ACTN1, TMSB10, MYH9, FLNC, WAS, SIRPA, FLNA, ELMO1, XIRP1, CFL1, USH1C, TMSB4X, DBN1, AKAP2, LCP1, MYO5A, TCAP, ARPC4, MYO9B, CALR, ITGB1, PFN2, EZR, RAC2, BCL2, AVIL, CNN2, SH2B2, WIPF1, INF2, ACTC1, RACGAP1, GAS7, CAPN3, CORO1C, PPP1R9B, CORO1A, EPB4.1L1, EPS8, NRAP, DIAP2, TMOD1, DIAP1	2019	176	13588	2.41	4.83E-08	4.83E-09	2.24E-08
GOTERM_BP_FAT	GO:0030036~actin cytoskeleton organization	59	2.07	6.11E-11	ENAH, TLN1, LIMA1, NUAK2, AIF1, CAPZA2, S100A9, WASF2, CAPZA1, PDLIM3, SHC1, CAP1, PLS3, RHOG, FMNL1, DBNL, ARHGEF2, FMNL3, ACTN4, PRKCI, ACTN1, TMSB10, MYH9, WAS, SIRPA, FLNA, ELMO1, XIRP1, CFL1, USH1C, TMSB4X, DBN1, AKAP2, LCP1, TCAP, ARPC4, CALR, ITGB1, PFN2, EZR, RAC2, BCL2, AVIL, SH2B2, CNN2, INF2, ACTC1, RACGAP1, GAS7, CAPN3, CORO1C, PPP1R9B, CORO1A, EPB4.1L1, EPS8, NRAP, DIAP2, TMOD1, DIAP1	2019	165	13588	2.41	2.45E-07	2.04E-08	1.14E-07
GOTERM_BP_FAT	GO:0007015~actin filament organization	28	0.98	3.34E-09	LIMA1, ENAH, AIF1, PDLIM3, ARPC4, EZR, BCL2, AVIL, PLS3, DBNL, ARHGEF2, ACTC1, ACTN4, PRKCI, ACTN1, GAS7, SIRPA, WAS, FLNA, PPP1R9B, CORO1A, CFL1, USH1C, DIAP2, AKAP2, DBN1, LCP1, DIAP1	2019	56	13588	3.37	1.34E-05	8.35E-07	6.20E-06
GOTERM_BP_FAT	GO:0007010~cytoskeleton organization	83	2.91	5.83E-07	LIMA1, TLN1, ENAH, NUAK2, AIF1, CAPZA2, CAPZA1, WASF2, S100A9, PDLIM3, VILL, SEH1L, TUBB5, SHC1, RANBP1, NEURL1A, CAP1, TUBA1B, RHOG, PLS3, FMNL1, DBNL, ARHGEF2, FMNL3, KIF11, ACTN4, PRKCI, ACTN1, TMSB10, MYH9, WAS, SIRPA, UBE2B, FLNA, TACC1, THY1, ELMO1, XIRP1, TPPP, CFL1, USH1C, TMSB4X, STMN1, HAUS8, DBN1, GADD45A, AKAP2, LCP1, TCAP, ARPC4, CALR, ITGB1, PFN2, DOCK2, EZR, RAC2, BCL2, NPM1, AVIL, FBXO5, CNN2, SH2B2, TES, INF2, ACTC1, CEP192, KIF18A, NUF2, NDC80, RACGAP1, CAPN3, GAS7, CORO1C, PPP1R9B, CORO1A, YWHAH, EPB4.1L1, NRAP, EPS8, LASP1, DIAP2, TMOD1, DIAP1	2019	326	13588	1.71	2.33E-03	5.43E-05	1.08E-03

Annotation Cluster 4 Enrichment Score: 8.289551872967115

Category	Term	Count	%	PValue	Genes	List Total	Pop Hits	Pop Total	Fold Enrichment	Bonferroni	Benjamini	FDR
GOTERM_MF_FAT	GO:0017076~purine nucleotide binding	370	12.95	2.47E-11	OASL2, OASL1, MAP3K8, GM4841, VPS4B, DHX34, DHX33, DDX10, RAB20, MAP2K6, RAB27A, PIK3CG, GBP6, BCR, MYLK4, MYLK2, IFI47, MYH7, WNK2, GEM, MYH9, MYH8, CARNS1, RFC5, RND3, RFC3, MAPK6, ACAP1, PDGFRA, RAB15, ARL8A, RAB12, MAPK7, EIF2AK3, 5430435G22RIK, CHKA, ACADSB, GNAI3, GNAI2, PFKFB3, PFKFB1, OAS3, STK17B, CHEK1, ARF6, HSPA1A, MYO9B, HSPA1B, OAS2, RAB40B, TK2, MOV10, MUSK, RAC2, DHX58, KIF3B, SPHK1, TAOK3, UBE2L6, RAB7L1, ABCG3, KIF3C, ATP13A5, EPHA2, ABCG1, HYOU1, ATP2A2, ARF3, ARF4, HSPA13, GRK5, ACAD11, CLCN5, NUAK2, MYO7A, ATP10A, CAMKK1, CKB, CAMKK2, ACTR3, MCCC2, ACTR2, FANCM, NOD2, SLC22A4, EEF2K, TUBB5, TUBB6, DOCK10, ARL5C, SIK1, TOP2A, TARSL2, DOCK11, TUBB3, DHCR24, CDC7, RAP2B, RAP2A, LYN, PHKG1, RAB4A, RAB4B, PKDCC, PDE10A, 1700017B05RIK, MAST3, GM12250, SQLE, ADK, RIPK1, EIF4A1, MAP3K14, ARL4D, STEAP1, ARL4C, RASD2, MYO5A, RAB3A, ALDH18A1, MKNK2, HK1, KIT, GCH1, GALK2, GALK1, RAB44, RAB43, MORC4, FMO1, PTK2B, HK3, ABCD2, PRKAA1, CERK, PAPSS2, TRIP13, ACSL5, MYO5C, PDK2, ACTC1, NOS1, SWAP70, PDK3, PDK4, ATP1A3, AXL, ACLY, NADK, ATP1A1, APRT, KHK, RPS6KA5, RAB32, GM6548, RAB31, RPS6KA4, RPS6KA1, FYN, RAB34, ARAF, ABCC3, GYK, PGK1, GNA13, RNASEL, GNA15, CTPS, F13A1, RRAD, ITPKC, PI4K2B, MTHFD1L, DDX27, PRKAR2B, ARL11, RALB, MLKL, DDX21, STK39, SAR1B, MX1, MX2, MATK, EGFR, NUDT2, PDXK, TWF2, PNCK, CNGA3, STK4, STK3, MAP4K4, DDX39, ATP9A, PI4K2A, RIPK3, OAS1A, LRRK1, OAS1G, PCCA, EP400, SRXN1, PRPS2, GPN2, IFIH1, ASS1, FGR, ACTR3B, STK10, MAP4K1, CTPS2, TIMP4, ABCA1, RRAGD, IRAK4, IRAK3, RHOBTB2, ERO1L, UBE2D1, RUNX1, ENTPD1, HELLS, CSF1R, TEC, RHOBTB3, TRP53, EEF1A1, RAB8A, ABCB9, RAB8B, MYO1C, EEF1A2, AK1, MYO1G, ATAD2, MYO1F, DOCK7, DOCK8, ABCB7, PSMB8, MMAB, PSMB9, RAB33B, DDX58, IKBKE, P2RX4, DNA2, PLK4, DDX56, P2RX7, P2RX6, PLK3, PLK2, ULK1, DYRK1B, ULK2, TUBD1, RAP1B, MERTK, PIP4K2A, UBE2E1, CDK19, KIF22, CDK17, D2HGDH, ATL2, FIGNL1, SLFN9, ATL3, PPIP5K2, SLFN8, CAD, FES, RHOV, SLFN4, SLFN1, SLFN2, ACVR1B, GSR, PAK2, PAK4, ATP8B1, ATP8B2, MKKS, CSK, TUBA1A, MDN1, RHOF, TUBA1B, CDK14, TUBA1C, RHOG, ATP8B4, PRKCA, IRAK2, CDK1, SGK1, ACADM, KIF11, HSP90AA1, LIMK1, PRKCI, CDK6, PFKM, PBK, UBE2H, MCM3, NLRP3, PRKCE, PRKCD, MCM4, UBE2B, CDK2, MCM5, ARL3, MCM6, PRKCB, DAPK1, PANK4, ATP6V1A, PRKCQ, RRAS2, KHSRP, ERN1, H2-AA, TXNRD1, CAMK1D, UBE2Z, DCK, TRIB3, ASNS, TRIB1, IGF1R, NAIP2, TAP2, TAP1, TOR1A, PIKFYVE, CAMK2B, UCK2, HSPA5, EHD1, CAMK2A, DCLK1, EHD4, AMHR2, ACTB, ALPK1, HCK, KIF18A, SMC2, PCX, HSP90B1, CYBB, ATP8A2, APAF1, H2-	1935	1871	13288	1.36	3.50E-08	1.17E-08	4.08E-08
GOTERM_MF_FAT	GO:0032553~ribonucleotide binding	356	12.46	4.82E-11	KIFC2, XRCC5, KIFC1, ADCY7, TUBB2B, TUBB2A, AURKB, C230081A13RIK, PRKX, BTK, DHX37, OASL2, OASL1, MAP3K8, GM4841, VPS4B, DHX34, DHX33, DDX10, RAB20, MAP2K6, RAB27A, PIK3CG, GBP6, BCR, MYLK4, MYLK2, IFI47, MYH7, WNK2, GEM, MYH9, MYH8, CARNS1, RFC5, RND3, RFC3, MAPK6, ACAP1, PDGFRA, RAB15, ARL8A, RAB12, MAPK7, EIF2AK3, 5430435G22RIK, CHKA, GNAI3, GNAI2, PFKFB3, PFKFB1, OAS3, STK17B, CHEK1, ARF6, HSPA1A, MYO9B, HSPA1B, OAS2, RAB40B, TK2, MOV10, MUSK, RAC2, DHX58, KIF3B, SPHK1, TAOK3, UBE2L6, RAB7L1, ABCG3, KIF3C, ATP13A5, EPHA2, ABCG1, HYOU1, ATP2A2, ARF3, ARF4, HSPA13, GRK5, CLCN5, NUAK2, MYO7A, ATP10A, CAMKK1, CKB, CAMKK2, ACTR3, MCCC2, ACTR2, FANCM, NOD2, SLC22A4, EEF2K, TUBB5, TUBB6, DOCK10, ARL5C, SIK1, TOP2A, TARSL2, DOCK11, TUBB3, CDC7, RAP2B, RAP2A, LYN, PHKG1, RAB4A, RAB4B, PKDCC, PDE10A, 1700017B05RIK, MAST3, GM12250, ADK, RIPK1, EIF4A1, MAP3K14, ARL4D, ARL4C, RASD2, MYO5A, RAB3A, ALDH18A1, MKNK2, HK1, KIT, GCH1, GALK2, GALK1, RAB44, RAB43, MORC4, PTK2B, HK3, ABCD2, PRKAA1, CERK, PAPSS2, TRIP13, ACSL5, MYO5C, PDK2, ACTC1, SWAP70, PDK3, PDK4, ATP1A3, AXL, ACLY, NADK, ATP1A1, APRT, KHK, RPS6KA5, RAB32, GM6548, RAB31, RPS6KA4, RPS6KA1, FYN, RAB34, ARAF, ABCC3, GYK, PGK1, GNA13, RNASEL, GNA15, CTPS, F13A1, RRAD, ITPKC, PI4K2B, MTHFD1L, DDX27, PRKAR2B, ARL11, RALB, MLKL, DDX21, STK39, SAR1B, MX1, MX2, MATK, EGFR, NUDT2, PDXK, TWF2, PNCK, CNGA3, STK4, STK3, MAP4K4, DDX39, ATP9A, PI4K2A, RIPK3, OAS1A, LRRK1, OAS1G, PCCA, EP400, SRXN1, PRPS2, GPN2, IFIH1, ASS1, FGR, ACTR3B, STK10, MAP4K1, CTPS2, TIMP4, ABCA1, RRAGD, IRAK4, IRAK3, RHOBTB2, UBE2D1, RUNX1, ENTPD1, HELLS, CSF1R, TEC, RHOBTB3, TRP53, EEF1A1, RAB8A, ABCB9, RAB8B, MYO1C, EEF1A2, AK1, MYO1G, ATAD2, MYO1F, DOCK7, DOCK8, ABCB7, PSMB8, MMAB, PSMB9, RAB33B, DDX58, IKBKE, P2RX4, DNA2, PLK4, DDX56, P2RX7, P2RX6, PLK3, PLK2, ULK1, DYRK1B, ULK2, TUBD1, RAP1B, MERTK, PIP4K2A, UBE2E1, CDK19, KIF22, CDK17, ATL2, FIGNL1, SLFN9, ATL3, PPIP5K2, SLFN8, CAD, FES, RHOV, SLFN4, SLFN1, SLFN2, ACVR1B, PAK2, PAK4, ATP8B1, ATP8B2, MKKS, CSK, TUBA1A, MDN1, RHOF, TUBA1B, TUBA1C, RHOG, CDK14, ATP8B4, PRKCA, IRAK2, CDK1, SGK1, KIF11, HSP90AA1, LIMK1, PRKCI, CDK6, PFKM, PBK, UBE2H, MCM3, NLRP3, PRKCE, PRKCD, MCM4, UBE2B, CDK2, MCM5, ARL3, MCM6, PRKCB, DAPK1, PANK4, ATP6V1A, PRKCQ, RRAS2, KHSRP, ERN1, H2-AA, CAMK1D, UBE2Z, DCK, TRIB3, ASNS, TRIB1, IGF1R, NAIP2, TAP2, TAP1, TOR1A, PIKFYVE, CAMK2B, UCK2, HSPA5, EHD1, CAMK2A, DCLK1, EHD4, AMHR2, ACTB, ALPK1, HCK, KIF18A, SMC2, PCX, HSP90B1, ATP8A2, APAF1, IPPK, H2-DMA, CDK20	1935	1796	13288	1.36	6.82E-08	1.70E-08	7.95E-08

GOTERM_MF_FAT	GO:0032555~purine ribonucleotide binding	356	12.46	4.82E-11	KIFC2, XRCC5, KIFC1, ADCY7, TUBB2B, TUBB2A, AURKB, C230081A13RIK, PRKX, BTK, DHX37, OASL2, OASL1, MAP3K8, GM4841, VPS4B, DHX34, DHX33, DDX10, RAB20, MAP2K6, RAB27A, PIK3CG, GBP6, BCR, MYLK4, MYLK2, IFI47, MYH7, WNK2, GEM, MYH9, MYH8, CARNS1, RFC5, RND3, RFC3, MAPK6, ACAP1, PDGFRA, RAB15, ARL8A, RAB12, MAPK7, EIF2AK3, 5430435G22RIK, CHKA, GNAI3, GNAI2, PFKFB3, PFKFB1, OAS3, STK17B, CHEK1, ARF6, HSPA1A, MYO9B, HSPA1B, OAS2, RAB40B, TK2, MOV10, MUSK, RAC2, DHX58, KIF3B, SPHK1, TAOK3, UBE2L6, RAB7L1, ABCG3, KIF3C, ATP13A5, EPHA2, ABCG1, HYOU1, ATP2A2, ARF3, ARF4, HSPA13, GRK5, CLCN5, NUAK2, MYO7A, ATP10A, CAMKK1, CKB, CAMKK2, ACTR3, MCCC2, ACTR2, FANCM, NOD2, SLC22A4, EEF2K, TUBB5, TUBB6, DOCK10, ARL5C, SIK1, TOP2A, TARSL2, DOCK11, TUBB3, CDC7, RAP2B, RAP2A, LYN, PHKG1, RAB4A, RAB4B, PKDCC, PDE10A, 1700017B05RIK, MAST3, GM12250, ADK, RIPK1, EIF4A1, MAP3K14, ARL4D, ARL4C, RASD2, MYO5A, RAB3A, ALDH18A1, MKNK2, HK1, KIT, GCH1, GALK2, GALK1, RAB44, RAB43, MORC4, PTK2B, HK3, ABCD2, PRKAA1, CERK, PAPSS2, TRIP13, ACSL5, MYO5C, PDK2, ACTC1, SWAP70, PDK3, PDK4, ATP1A3, AXL, ACLY, NADK, ATP1A1, APRT, KHK, RPS6KA5, RAB32, GM6548, RAB31, RPS6KA4, RPS6KA1, FYN, RAB34, ARAF, ABCC3, GYK, PGK1, GNA13, RNASEL, GNA15, CTPS, F13A1, RRAD, ITPKC, PI4K2B, MTHFD1L, DDX27, PRKAR2B, ARL11, RALB, MLKL, DDX21, STK39, SAR1B, MX1, MX2, MATK, EGFR, NUDT2, PDXK, TWF2, PNCK, CNGA3, STK4, STK3, MAP4K4, DDX39, ATP9A, PI4K2A, RIPK3, OAS1A, LRRK1, OAS1G, PCCA, EP400, SRXN1, PRPS2, GPN2, IFIH1, ASS1, FGR, ACTR3B, STK10, MAP4K1, CTPS2, TIMP4, ABCA1, RRAGD, IRAK4, IRAK3, RHOBTB2, UBE2D1, RUNX1, ENTPD1, HELLS, CSF1R, TEC, RHOBTB3, TRP53, EEF1A1, RAB8A, ABCB9, RAB8B, MYO1C, EEF1A2, AK1, MYO1G, ATAD2, MYO1F, DOCK7, DOCK8, ABCB7, PSMB8, MMAB, PSMB9, RAB33B, DDX58, IKBKE, P2RX4, DNA2, PLK4, DDX56, P2RX7, P2RX6, PLK3, PLK2, ULK1, DYRK1B, ULK2, TUBD1, RAP1B, MERTK, PIP4K2A, UBE2E1, CDK19, KIF22, CDK17, ATL2, FIGNL1, SLFN9, ATL3, PPIP5K2, SLFN8, CAD, FES, RHOV, SLFN4, SLFN1, SLFN2, ACVR1B, PAK2, PAK4, ATP8B1, ATP8B2, MKKS, CSK, TUBA1A, MDN1, RHOF, TUBA1B, TUBA1C, RHOG, CDK14, ATP8B4, PRKCA, IRAK2, CDK1, SGK1, KIF11, HSP90AA1, LIMK1, PRKCI, CDK6, PFKM, PBK, UBE2H, MCM3, NLRP3, PRKCE, PRKCD, MCM4, UBE2B, CDK2, MCM5, ARL3, MCM6, PRKCB, DAPK1, PANK4, ATP6V1A, PRKCQ, RRAS2, KHSRP, ERN1, H2-AA, CAMK1D, UBE2Z, DCK, TRIB3, ASNS, TRIB1, IGF1R, NAIP2, TAP2, TAP1, TOR1A, PIKFYVE, CAMK2B, UCK2, HSPA5, EHD1, CAMK2A, DCLK1, EHD4, AMHR2, ACTB, ALPK1, HCK, KIF18A, SMC2, PCX, HSP90B1, ATP8A2, APAF1, IPPK, H2-DMA, CDK20	1935	1796	13288	1.36	6.82E-08	1.70E-08	7.95E-08
GOTERM_MF_FAT	GO:0000166~nucleotide binding	413	14.46	7.12E-10	DHX34, DHX33, MAP2K6, RAB27A, PIK3CG, GBP6, MYLK4, PTBP1, MYLK2, MYH7, GEM, MYH9, MYH8, MAPK6, ARL8A, MAPK7, GM12070, EIF2AK3, ACADSB, GNAI3, GNAI2, HSPA1A, ARF6, HSPA1B, MUSK, RAC2, PABPC1, DHX58, GPD1, KIF3B, SPHK1, TAOK3, UBE2L6, RAB7L1, VDAC2, KIF3C, VDAC3, VDAC1, ARF3, ARF4, PPRC1, HSPA13, ACAD11, NUAK2, ATP10A, NOD2, SLC22A4, EEF2K, SIK1, ARL5C, TARSL2, CDC7, RAP2B, RAP2A, LYN, PHKG1, PDE10A, PKDCC, 1700017B05RIK, MAST3, SQLE, RIPK1, EIF4A1, ARL4D, ARL4C, RASD2, ALDH18A1, UGDH, POLA1, MKNK2, HK1, SF3B4, FMO1, PTK2B, HK3, CERK, PAPSS2, TRIP13, ACSL5, PDK2, ACTC1, SWAP70, PDK3, PDK4, AXL, NADK, ACLY, RPS6KA5, RPS6KA4, RPS6KA1, POLDIP3, ARAF, HPGD, GNA13, GNA15, RNASEL, HMGCR, RBM3, RBM15B, RRAD, MTHFD1L, DDX27, PRKAR2B, RALB, DDX21, STK39, SAR1B, RDM1, MATK, EGFR, NUDT2, PDXK, TWF2, DDX39, ATP9A, OAS1A, LRRK1, OAS1G, SRXN1, EP400, ME1, ME2, FGR, ASS1, STK10, NT5C1A, TIMP4, CTPS2, ABCA1, P2RY6, HNRNPF, HELLS, TEC, TRP53, ABCB9, RAB8A, RAB8B, MYO1C, MYO1G, MYO1F, DOCK7, DOCK8, ABCB7, MMAB, IDH3A, DDX58, IKBKE, DNA2, P2RX4, PLK4, P2RX7, DDX56, P2RX6, PLK3, PLK2, DYRK1B, POLD1, TUBD1, MERTK, KIF22, ATL2, FIGNL1, SLFN9, ATL3, SLFN8, CAD, FES, RHOV, SLFN4, SLFN1, SLFN2, RHOF, RHOG, CDK1, SGK1, ACADM, KIF11, LIMK1, CDK6, PBK, NCL, CDK2, ARL3, ATP6V1A, RRAS2, ERN1, TXNRD1, DCK, ASNS, IGF1R, NAIP2, TOR1A, HSPA5, EHD1, DCLK1, EHD4, AMHR2, ALPK1, HCK, SMC2, PCX, CYBB, HSP90B1, APAF1, IPPK, H2-DMA, ADCY7, AURKB, PRKX, SULT1A1, OASL2, OASL1, NT5C2, GM4841, DDX10, RAB20, BCR, IFI47, NAV3, WNK2, CARNS1, RFC5, RND3, RFC3, ACAP1, PDGFRA, RAB15, RAB12, MDH1, 5430435G22RIK, CHKA, PFKFB3, PFKFB1, OAS3, STK17B, CHEK1, MYO9B, IGF2BP2, OAS2, RAB40B, TK2, MOV10, ABCG3, ATP13A5, ABCG1, EPHA2, HYOU1, ATP2A2, GRK5, CLCN5, MYO7A, CAMKK1, CKB, CAMKK2, ACTR3, ACTR2, MCCC2, FANCM, TUBB5, TUBB6, RBM47, DOCK10, TOP2A, DOCK11, NT5E, TUBB3, DHCR24, NT5C, RAB4A, RAB4B, CBR4, GM12250, ADK, MAP3K14, STEAP1, MYO5A, RAB3A, KIT, GCH1, GALK2, GALK1, RAB44, RAB43, MORC4, ABCD2, PRKAA1, MYO5C, NOS1, ATP1A3, ATP1A1, APRT, KHK, RAB32, GM6548, RAB31, PYGM, PYGL, FYN, RAB34, PHGDH, ABCC3, GYK, RBM14, PGK1, CTPS, F13A1, PI4K2B, ITPKC, ARL11, MLKL, MX1, MX2, RBMS1, PNCK, CNGA3, STK4, POR, STK3, MAP4K4, PI4K2A, RIPK3, PRPS2, PCCA, GPN2, IFIH1, ACTR3B, MAP4K1, RRAGD, IRAK4, IRAK3, RHOBTB2, ERO1L, UBE2D1, RUNX1, ENTPD1, CSF1R, RHOBTB3, EEF1A1, AK1, EEF1A2, ATAD2, PSMB8, PSMB9, RAB33B, ULK1, ULK2, RAP1B, PIP4K2A, UBE2E1, CDK19, CDK17, D2HGDH, PPIP5K2, MKI67IP, GSR, ACVR1B, PAK2, PAK4, ATP8B1, ATP8B2,	1935	2183	13288	1.30	1.01E-06	1.68E-07	1.17E-06
GOTERM_MF_FAT	GO:0030554~adenyl nucleotide binding	295	10.33	9.02E-08	XRCC5, KIFC2, KIFC1, ADCY7, AURKB, C230081A13RIK, PRKX, BTK, ERO1LB, DHX37, OASL2, OASL1, MAP3K8, DHX34, VPS4B, DHX33, DDX10, MAP2K6, PIK3CG, BCR, MYLK4, MYLK2, MYH7, WNK2, MYH9, MYH8, CARNS1, RFC5, RFC3, MAPK6, ACAP1, PDGFRA, MAPK7, EIF2AK3, CHKA, ACADSB, PFKFB3, PFKFB1, OAS3, STK17B, CHEK1, HSPA1A, MYO9B, HSPA1B, OAS2, TK2, MOV10, MUSK, DHX58, KIF3B, SPHK1, TAOK3, UBE2L6, ABCG3, KIF3C, ATP13A5, EPHA2, ABCG1, HYOU1, ATP2A2, HSPA13, GRK5, ACAD11, CLCN5, NUAK2, MYO7A, ATP10A, CAMKK1, CAMKK2, CKB, ACTR3, ACTR2, MCCC2, FANCM, NOD2, SLC22A4, EEF2K, SIK1, TOP2A, TARSL2, DHCR24, CDC7, LYN, PHKG1, PKDCC, PDE10A, MAST3, SQLE, ADK, RIPK1, EIF4A1, MAP3K14, STEAP1, MYO5A, ALDH18A1, MKNK2, HK1, KIT, GALK2, GALK1, MORC4, FMO1, PTK2B, HK3, ABCD2, PRKAA1, CERK, PAPSS2, TRIP13, ACSL5, MYO5C, PDK2, ACTC1, NOS1, SWAP70, PDK3, PDK4, ATP1A3, AXL, ACLY, NADK, ATP1A1, APRT, KHK, RPS6KA5, RPS6KA4, RPS6KA1, FYN, ARAF, ABCC3, GYK, PGK1, RNASEL, CTPS, F13A1, ITPKC, PI4K2B, MTHFD1L, DDX27, PRKAR2B, MLKL, STK39, DDX21, MATK, EGFR, TWF2, PDXK, PNCK, STK4, STK3, MAP4K4, DDX39, ATP9A, PI4K2A, RIPK3, OAS1A, LRRK1, OAS1G, PCCA, EP400, SRXN1, PRPS2, IFIH1, FGR, ASS1, ACTR3B, STK10, MAP4K1, CTPS2, TIMP4, ABCA1, IRAK4, IRAK3, ERO1L, UBE2D1, ENTPD1, RUNX1, HELLS, CSF1R, TEC, RHOBTB3, TRP53, ABCB9, MYO1C, AK1, MYO1G, ATAD2, MYO1F, ABCB7, PSMB8, MMAB, PSMB9, DDX58, IKBKE, P2RX4, DNA2, P2RX7, DDX56, PLK4, P2RX6, PLK3, PLK2, ULK1, ULK2, DYRK1B, MERTK, PIP4K2A, UBE2E1, CDK19, KIF22, CDK17, D2HGDH, FIGNL1, SLFN9, PPIP5K2, SLFN8, CAD, FES, SLFN4, SLFN1, SLFN2, ACVR1B, GSR, PAK2, PAK4, ATP8B1, ATP8B2, MKKS, CSK, MDN1, CDK14, ATP8B4, PRKCA, IRAK2, CDK1, SGK1, ACADM, KIF11, HSP90AA1, LIMK1, PRKCI, CDK6, PFKM, PBK, UBE2H, MCM3, NLRP3, PRKCE, PRKCD, MCM4, UBE2B, CDK2, MCM5, DAPK1, PRKCB, MCM6, PANK4, ATP6V1A, PRKCQ, KHSRP, ERN1, H2-AA, TXNRD1, CAMK1D, UBE2Z, DCK, TRIB3, ASNS, TRIB1, IGF1R, NAIP2, TAP2, TAP1, TOR1A, PIKFYVE, CAMK2B, UCK2, HSPA5, EHD1, CAMK2A, DCLK1, EHD4, AMHR2, ACTB, ALPK1, HCK, KIF18A, SMC2, PCX, HSP90B1, CYBB, ATP8A2, APAF1, IPPK, H2-DMA, CDK20	1935	1535	13288	1.32	1.28E-04	1.59E-05	1.49E-04
GOTERM_MF_FAT	GO:0001883~purine nucleoside binding	296	10.36	1.32E-07	XRCC5, KIFC2, KIFC1, ADCY7, AURKB, C230081A13RIK, PRKX, BTK, ERO1LB, DHX37, OASL2, OASL1, MAP3K8, DHX34, VPS4B, DHX33, DDX10, MAP2K6, PIK3CG, BCR, MYLK4, MYLK2, MYH7, WNK2, MYH9, MYH8, CARNS1, RFC5, RFC3, MAPK6, ACAP1, PDGFRA, MAPK7, EIF2AK3, CHKA, ACADSB, PFKFB3, PFKFB1, OAS3, STK17B, CHEK1, HSPA1A, MYO9B, HSPA1B, OAS2, TK2, MOV10, MUSK, DHX58, KIF3B, SPHK1, TAOK3, UBE2L6, ABCG3, KIF3C, ATP13A5, EPHA2, ABCG1, HYOU1, ATP2A2, HSPA13, GRK5, ACAD11, CLCN5, NUAK2, MYO7A, ATP10A, CAMKK1, CAMKK2, CKB, ACTR3, MCCC2, ACTR2, FANCM, NOD2, SLC22A4, EEF2K, SIK1, TOP2A, TARSL2, DHCR24, CDC7, LYN, PHKG1, PKDCC, PDE10A, MAST3, SQLE, ADK, RIPK1, EIF4A1, MAP3K14, STEAP1, MYO5A, ALDH18A1, MKNK2, HK1, KIT, GALK2, GALK1, MORC4, FMO1, PTK2B, HK3, ABCD2, PRKAA1, CERK, PAPSS2, TRIP13, ACSL5, MYO5C, PDK2, ACTC1, NOS1, SWAP70, PDK3, PDK4, ATP1A3, AXL, ACLY, NADK, ATP1A1, APRT, KHK, RPS6KA5, RPS6KA4, RPS6KA1, FYN, ARAF, ABCC3, GYK, PGK1, RNASEL, CTPS, F13A1, ITPKC, PI4K2B, MTHFD1L, DDX27, PRKAR2B, MLKL, STK39, DDX21, MATK, EGFR, TWF2, PDXK, PNCK, CNGA3, STK4, STK3, MAP4K4, DDX39, ATP9A, PI4K2A, RIPK3, OAS1A, LRRK1, OAS1G, PCCA, EP400, SRXN1, PRPS2, IFIH1, FGR, ASS1, ACTR3B, STK10, MAP4K1, CTPS2, TIMP4, ABCA1, IRAK4, IRAK3, ERO1L, UBE2D1, ENTPD1, RUNX1, HELLS, CSF1R, TEC, RHOBTB3, TRP53, ABCB9, MYO1C, AK1, MYO1G, ATAD2, MYO1F, ABCB7, PSMB8, MMAB, PSMB9, DDX58, IKBKE, P2RX4, DNA2, P2RX7, DDX56, PLK4, P2RX6, PLK3, PLK2, ULK1, ULK2, DYRK1B, MERTK, PIP4K2A, UBE2E1, CDK19, KIF22, CDK17, D2HGDH, FIGNL1, SLFN9, PPIP5K2, SLFN8, CAD, FES, SLFN4, SLFN1, SLFN2, ACVR1B, GSR, PAK2, PAK4, ATP8B1, ATP8B2, MKKS, CSK, MDN1, CDK14, ATP8B4, PRKCA, IRAK2, CDK1, SGK1, ACADM, KIF11, HSP90AA1, LIMK1, PRKCI, CDK6, PFKM, PBK, UBE2H, MCM3, NLRP3, PRKCE, PRKCD, MCM4, UBE2B, CDK2, MCM5, DAPK1, PRKCB, MCM6, PANK4, ATP6V1A, PRKCQ, KHSRP, ERN1, H2-AA, TXNRD1, CAMK1D, UBE2Z, DCK, TRIB3, ASNS, TRIB1, IGF1R, NAIP2, TAP2, TAP1, TOR1A, PIKFYVE, CAMK2B, UCK2, HSPA5, EHD1, CAMK2A, DCLK1, EHD4, AMHR2, ACTB, ALPK1, HCK, KIF18A, SMC2, PCX, HSP90B1, CYBB, ATP8A2, APAF1, IPPK, H2-DMA, CDK20	1935	1548	13288	1.31	1.87E-04	1.70E-05	2.17E-04
GOTERM_MF_FAT	GO:0032559~adenyl ribonucleotide binding	281	9.84	1.66E-07	XRCC5, KIFC2, KIFC1, ADCY7, AURKB, C230081A13RIK, PRKX, BTK, DHX37, OASL2, OASL1, MAP3K8, DHX34, VPS4B, DHX33, DDX10, MAP2K6, PIK3CG, BCR, MYLK4, MYLK2, MYH7, WNK2, MYH9, MYH8, CARNS1, RFC5, RFC3, MAPK6, ACAP1, PDGFRA, MAPK7, EIF2AK3, CHKA, PFKFB3, PFKFB1, OAS3, STK17B, CHEK1, MYO9B, HSPA1A, OAS2, HSPA1B, TK2, MOV10, MUSK, DHX58, KIF3B, SPHK1, TAOK3, UBE2L6, ABCG3, KIF3C, ATP13A5, EPHA2, ABCG1, HYOU1, ATP2A2, HSPA13, GRK5, CLCN5, NUAK2, MYO7A, ATP10A, CAMKK1, CAMKK2, CKB, ACTR3, ACTR2, MCCC2, FANCM, NOD2, SLC22A4, EEF2K, SIK1, TOP2A, TARSL2, CDC7, LYN, PHKG1, PKDCC, PDE10A, MAST3, ADK, RIPK1, EIF4A1, MAP3K14, MYO5A, ALDH18A1, MKNK2, HK1, KIT, GALK2, GALK1, MORC4, PTK2B, HK3, ABCD2, PRKAA1, CERK, PAPSS2, TRIP13, ACSL5, MYO5C, PDK2, ACTC1, SWAP70, PDK3, PDK4, ATP1A3, AXL, ACLY, NADK, ATP1A1, APRT, KHK, RPS6KA5, RPS6KA4, RPS6KA1, FYN, ARAF, ABCC3, GYK, PGK1, RNASEL, CTPS, F13A1, ITPKC, PI4K2B, MTHFD1L, DDX27, PRKAR2B, MLKL, STK39, DDX21, MATK, EGFR, TWF2, PDXK, PNCK, STK4, STK3, MAP4K4, DDX39, ATP9A, PI4K2A, RIPK3, OAS1A, LRRK1, OAS1G, PCCA, EP400, PRPS2, SRXN1, IFIH1, FGR, ASS1, ACTR3B, STK10, MAP4K1, CTPS2, TIMP4, ABCA1, IRAK4, IRAK3, UBE2D1, ENTPD1, RUNX1, HELLS, CSF1R, TEC, RHOBTB3, TRP53, ABCB9, MYO1C, AK1, MYO1G, ATAD2, MYO1F, ABCB7, PSMB8, MMAB, PSMB9, DDX58, IKBKE, P2RX4, DNA2, P2RX7, DDX56, PLK4, P2RX6, PLK3, PLK2, ULK1, ULK2, DYRK1B, MERTK, PIP4K2A, UBE2E1, CDK19, KIF22, CDK17, FIGNL1, SLFN9, PPIP5K2, SLFN8, CAD, FES, SLFN4, SLFN1, SLFN2, ACVR1B, PAK2, PAK4, ATP8B1, ATP8B2, MKKS, CSK, MDN1, CDK14, ATP8B4, PRKCA, IRAK2, CDK1, SGK1, KIF11, HSP90AA1, LIMK1, PRKCI, CDK6, PFKM, PBK, UBE2H, MCM3, NLRP3, PRKCE, PRKCD, MCM4, UBE2B, CDK2, MCM5, DAPK1, PRKCB, MCM6, PANK4, ATP6V1A, PRKCQ, KHSRP, ERN1, H2-AA, CAMK1D, UBE2Z, DCK, TRIB3, ASNS, TRIB1, IGF1R, NAIP2, TAP2, TAP1, TOR1A, PIKFYVE, CAMK2B, UCK2, HSPA5, EHD1, CAMK2A, DCLK1, EHD4, AMHR2, ACTB, ALPK1, HCK, KIF18A, SMC2, PCX, HSP90B1, ATP8A2, APAF1, IPPK, H2-DMA, CDK20	1935	1460	13288	1.32	2.35E-04	1.96E-05	2.74E-04

GOTERM_MF_FAT	GO:0001882~nucleoside binding	297	10.40	1.71E-07	XRCC5, KIFC2, KIFC1, ADCY7, AURKB, C230081A13RIK, PRKX, BTK, ERO1LB, DHX37, OASL2, OASL1, MAP3K8, DHX34, VPS4B, DHX33, DDX10, MAP2K6, PIK3CG, BCR, MYLK4, MYLK2, MYH7, WNK2, MYH9, MYH8, CARNS1, RFC5, RFC3, MAPK6, ACAP1, PDGFRA, MAPK7, EIF2AK3, CHKA, ACADSB, PFKFB3, PFKFB1, OAS3, STK17B, CHEK1, HSPA1A, MYO9B, HSPA1B, OAS2, TK2, MOV10, MUSK, DHX58, KIF3B, SPHK1, TAOK3, UBE2L6, ABCG3, KIF3C, ATP13A5, EPHA2, ABCG1, HYOU1, ATP2A2, HSPA13, GRK5, ACAD11, CLCN5, NUAK2, MYO7A, ATP10A, CAMKK1, CAMKK2, CKB, ACTR3, MCCC2, ACTR2, FANCM, NOD2, SLC22A4, EEF2K, SIK1, TOP2A, TARSL2, DHCR24, CDC7, LYN, PHKG1, PKDCC, PDE10A, MAST3, SQLE, ADK, RIPK1, EIF4A1, MAP3K14, STEAP1, MYO5A, ALDH18A1, POLA1, MKNK2, HK1, KIT, GALK2, GALK1, MORC4, FMO1, PTK2B, HK3, ABCD2, PRKAA1, CERK, PAPSS2, TRIP13, ACSL5, MYO5C, PDK2, ACTC1, NOS1, SWAP70, PDK3, PDK4, ATP1A3, AXL, ACLY, NADK, ATP1A1, APRT, KHK, RPS6KA5, RPS6KA4, RPS6KA1, FYN, ARAF, ABCC3, GYK, PGK1, RNASEL, CTPS, F13A1, ITPKC, PI4K2B, MTHFD1L, DDX27, PRKAR2B, MLKL, STK39, DDX21, MATK, EGFR, TWF2, PDXK, PNCK, CNGA3, STK4, STK3, MAP4K4, DDX39, ATP9A, PI4K2A, RIPK3, OAS1A, LRRK1, OAS1G, PCCA, EP400, SRXN1, PRPS2, IFIH1, FGR, ASS1, ACTR3B, STK10, MAP4K1, CTPS2, TIMP4, ABCA1, IRAK4, IRAK3, ERO1L, UBE2D1, ENTPD1, RUNX1, HELLS, CSF1R, TEC, RHOBTB3, TRP53, ABCB9, MYO1C, AK1, MYO1G, ATAD2, MYO1F, ABCB7, PSMB8, MMAB, PSMB9, DDX58, IKBKE, P2RX4, DNA2, P2RX7, DDX56, PLK4, P2RX6, PLK3, PLK2, ULK1, ULK2, DYRK1B, MERTK, PIP4K2A, UBE2E1, CDK19, KIF22, CDK17, D2HGDH, FIGNL1, SLFN9, PPIP5K2, SLFN8, CAD, FES, SLFN4, SLFN1, SLFN2, ACVR1B, GSR, PAK2, PAK4, ATP8B1, ATP8B2, MKKS, CSK, MDN1, CDK14, ATP8B4, PRKCA, IRAK2, CDK1, SGK1, ACADM, KIF11, HSP90AA1, LIMK1, PRKCI, CDK6, PFKM, PBK, UBE2H, MCM3, NLRP3, PRKCE, PRKCD, MCM4, UBE2B, CDK2, MCM5, DAPK1, PRKCB, MCM6, PANK4, ATP6V1A, PRKCQ, KHSRP, ERN1, H2-AA, TXNRD1, CAMK1D, UBE2Z, DCK, TRIB3, ASNS, TRIB1, IGF1R, NAIP2, TAP2, TAP1, TOR1A, PIKFYVE, CAMK2B, UCK2, HSPA5, EHD1, CAMK2A, DCLK1, EHD4, AMHR2, ACTB, ALPK1, HCK, KIF18A, SMC2, PCX, HSP90B1, CYBB, ATP8A2, APAF1, IPPK, H2-DMA, CDK20	1935	1558	13288	1.31	2.43E-04	1.87E-05	2.83E-04
GOTERM_MF_FAT	GO:0005524~ATP binding	278	9.73	1.79E-07	XRCC5, KIFC2, KIFC1, ADCY7, AURKB, C230081A13RIK, PRKX, BTK, DHX37, OASL2, OASL1, MAP3K8, DHX34, VPS4B, DHX33, DDX10, MAP2K6, PIK3CG, BCR, MYLK4, MYLK2, MYH7, WNK2, MYH9, MYH8, CARNS1, RFC5, RFC3, MAPK6, ACAP1, PDGFRA, MAPK7, EIF2AK3, CHKA, PFKFB3, PFKFB1, OAS3, STK17B, CHEK1, MYO9B, HSPA1A, OAS2, HSPA1B, TK2, MOV10, MUSK, DHX58, KIF3B, SPHK1, TAOK3, UBE2L6, ABCG3, KIF3C, ATP13A5, EPHA2, ABCG1, HYOU1, ATP2A2, HSPA13, GRK5, CLCN5, NUAK2, MYO7A, ATP10A, CAMKK1, CAMKK2, CKB, ACTR3, ACTR2, MCCC2, FANCM, NOD2, SLC22A4, EEF2K, SIK1, TOP2A, TARSL2, CDC7, LYN, PHKG1, PKDCC, MAST3, ADK, RIPK1, EIF4A1, MAP3K14, MYO5A, ALDH18A1, MKNK2, HK1, KIT, GALK2, GALK1, MORC4, PTK2B, HK3, ABCD2, PRKAA1, CERK, PAPSS2, TRIP13, ACSL5, MYO5C, PDK2, ACTC1, SWAP70, PDK3, PDK4, ATP1A3, AXL, ACLY, NADK, ATP1A1, KHK, RPS6KA5, RPS6KA4, RPS6KA1, FYN, ARAF, ABCC3, GYK, PGK1, RNASEL, CTPS, F13A1, ITPKC, PI4K2B, MTHFD1L, DDX27, MLKL, STK39, DDX21, MATK, EGFR, TWF2, PDXK, PNCK, STK4, STK3, MAP4K4, DDX39, ATP9A, PI4K2A, RIPK3, OAS1A, LRRK1, OAS1G, PCCA, EP400, PRPS2, SRXN1, IFIH1, FGR, ASS1, ACTR3B, STK10, MAP4K1, CTPS2, TIMP4, ABCA1, IRAK4, IRAK3, UBE2D1, ENTPD1, RUNX1, HELLS, CSF1R, TEC, RHOBTB3, TRP53, ABCB9, MYO1C, AK1, MYO1G, ATAD2, MYO1F, ABCB7, PSMB8, MMAB, PSMB9, DDX58, IKBKE, P2RX4, DNA2, P2RX7, DDX56, PLK4, P2RX6, PLK3, PLK2, ULK1, ULK2, DYRK1B, MERTK, PIP4K2A, UBE2E1, CDK19, KIF22, CDK17, FIGNL1, SLFN9, PPIP5K2, SLFN8, CAD, FES, SLFN4, SLFN1, SLFN2, ACVR1B, PAK2, PAK4, ATP8B1, ATP8B2, MKKS, CSK, MDN1, CDK14, ATP8B4, PRKCA, IRAK2, CDK1, SGK1, KIF11, HSP90AA1, LIMK1, PRKCI, CDK6, PFKM, PBK, UBE2H, MCM3, NLRP3, PRKCE, PRKCD, UBE2B, MCM4, CDK2, MCM5, DAPK1, PRKCB, MCM6, PANK4, ATP6V1A, PRKCQ, KHSRP, ERN1, H2-AA, CAMK1D, UBE2Z, DCK, TRIB3, ASNS, TRIB1, IGF1R, NAIP2, TAP2, TAP1, TOR1A, PIKFYVE, CAMK2B, UCK2, HSPA5, EHD1, CAMK2A, DCLK1, EHD4, AMHR2, ACTB, ALPK1, HCK, KIF18A, SMC2, PCX, HSP90B1, ATP8A2, APAF1, IPPK, H2-DMA, CDK20	1935	1443	13288	1.32	2.54E-04	1.81E-05	2.96E-04

Annotation Cluster 5 Enrichment Score: 5.893799472872889

Category	Term	Count	%	PValue	Genes	List Total	Pop Hits	Pop Total	Fold Enrichment	Bonferroni	Benjamini	FDR
GOTERM_BP_FAT	GO:0001775~cell activation	83	2.91	1.92E-13	GNA13, ELF4, TLR1, STAT5B, RORC, PTPN22, TLR4, IL15, NFKB2, SKAP2, TPD52, TGFB1, CBFB, CD48, SHB, CLCF1, CXCR4, WWP1, TICAM1, LTB, IMPDH1, RAB27A, EGR1, GAPT, BCL10, ICOSL, RELB, CD40, NFAM1, MYH9, WAS, PRKCD, IFNAR1, DCLRE1C, CD86, LAT2, UNC13D, LILRB3, GADD45G, CX3CR1, LCP1, LCP2, ITGAL, SBNO2, LST1, SOX4, ITGB2, SFPI1, PF4, IL7R, CD74, ADA, ITGAM, SLC11A1, DOCK2, ITGAX, BCL2, PPP3CB, FCER1G, BCL3, NDRG1, ENTPD1, HELLS, BLNK, FYB, TRP53, PTPRC, IKZF1, PLEK, H2-M3, SWAP70, MYO1F, VAV1, FCGR3, P2RX7, FCGR2B, SLC7A2, FYN, BAX, PLCG2, IRF1, H2-DMA, RBPJ	2019	246	13588	2.27	7.70E-10	2.57E-10	3.58E-10
GOTERM_BP_FAT	GO:0045321~leukocyte activation	76	2.66	4.43E-13	ELF4, TLR1, STAT5B, RORC, PTPN22, TLR4, IL15, SKAP2, TPD52, CBFB, TGFB1, CD48, SHB, CLCF1, CXCR4, WWP1, TICAM1, IMPDH1, RAB27A, EGR1, GAPT, BCL10, ICOSL, RELB, NFAM1, CD40, MYH9, WAS, PRKCD, IFNAR1, DCLRE1C, CD86, LAT2, UNC13D, LILRB3, GADD45G, CX3CR1, LCP1, LCP2, ITGAL, SBNO2, SOX4, ITGB2, SFPI1, IL7R, CD74, ADA, ITGAM, SLC11A1, DOCK2, ITGAX, BCL2, PPP3CB, FCER1G, BCL3, NDRG1, HELLS, BLNK, FYB, TRP53, PTPRC, IKZF1, H2-M3, SWAP70, MYO1F, VAV1, FCGR3, P2RX7, FCGR2B, SLC7A2, FYN, BAX, PLCG2, IRF1, H2-DMA, RBPJ	2019	219	13588	2.34	1.77E-09	4.44E-10	8.24E-10
GOTERM_BP_FAT	GO:0046649~lymphocyte activation	61	2.14	4.47E-09	ELF4, STAT5B, PTPN22, RORC, IL15, TPD52, SKAP2, CBFB, TGFB1, CD48, SHB, CLCF1, CXCR4, WWP1, IMPDH1, RAB27A, EGR1, GAPT, ICOSL, RELB, NFAM1, CD40, MYH9, WAS, PRKCD, IFNAR1, DCLRE1C, CD86, LAT2, UNC13D, GADD45G, LCP1, ITGAL, SOX4, SFPI1, ITGB2, IL7R, CD74, ITGAM, ADA, SLC11A1, DOCK2, ITGAX, BCL2, PPP3CB, BCL3, HELLS, BLNK, TRP53, PTPRC, IKZF1, H2-M3, SWAP70, VAV1, P2RX7, FYN, BAX, PLCG2, IRF1, H2-DMA, RBPJ	2019	191	13588	2.15	1.79E-05	1.05E-06	8.32E-06
GOTERM_BP_FAT	GO:0042110~T cell activation	41	1.44	1.02E-07	ITGAL, ELF4, STAT5B, SOX4, PTPN22, RORC, ITGB2, IL15, IL7R, CD74, ITGAM, TGFB1, CD48, SLC11A1, DOCK2, ITGAX, CXCR4, BCL2, WWP1, PPP3CB, BCL3, RAB27A, EGR1, TRP53, PTPRC, ICOSL, H2-M3, IKZF1, RELB, MYH9, WAS, VAV1, IFNAR1, CD86, P2RX7, FYN, BAX, GADD45G, IRF1, H2-DMA, LCP1	2019	116	13588	2.38	4.09E-04	1.51E-05	1.90E-04
GOTERM_BP_FAT	GO:0070661~leukocyte proliferation	22	0.77	1.41E-07	TRP53, PTPRC, ITGAL, ELF4, ITGB2, IL15, IL7R, PRKCD, TGFB1, ITGAM, SLC11A1, SHB, DOCK2, P2RX7, CD86, ITGAX, FYN, CXCR4, BAX, BCL2, IMPDH1, HELLS	2019	43	13588	3.44	5.64E-04	1.94E-05	2.62E-04
GOTERM_BP_FAT	GO:0032943~mononuclear cell proliferation	22	0.77	1.41E-07	TRP53, PTPRC, ITGAL, ELF4, ITGB2, IL15, IL7R, PRKCD, TGFB1, ITGAM, SLC11A1, SHB, DOCK2, P2RX7, CD86, ITGAX, FYN, CXCR4, BAX, BCL2, IMPDH1, HELLS	2019	43	13588	3.44	5.64E-04	1.94E-05	2.62E-04
GOTERM_BP_FAT	GO:0046651~lymphocyte proliferation	21	0.74	4.71E-07	TRP53, PTPRC, ITGAL, ELF4, ITGB2, IL15, IL7R, PRKCD, ITGAM, SLC11A1, SHB, DOCK2, P2RX7, CD86, ITGAX, FYN, CXCR4, BAX, BCL2, IMPDH1, HELLS	2019	42	13588	3.37	1.88E-03	4.71E-05	8.75E-04
GOTERM_BP_FAT	GO:0030097~hemopoiesis	68	2.38	6.57E-07	XRCC5, GMPR2, G6PDX, STAT5B, PTPN22, RORC, IL15, TPD52, CBFB, TGFB1, ANK1, ALAS2, CLCF1, WWP1, CASP8, HBB-B1, EGR1, SGPL1, STAP1, LYN, RELB, CDK6, NFAM1, MYH9, DNASE2A, DCLRE1C, PGM3, HIF1A, LILRB3, CCR2, VEGFA, GADD45G, CCR1, SLC37A4, PML, SOX4, SFXN1, SFPI1, TTC7, KIT, IL7R, CD74, ADA, MLF1, RPA1, DOCK2, BCL2, PPP3CB, BCL3, RUNX1, HELLS, CEBPA, TRP53, PTPRC, IKZF1, EPAS1, HCLS1, BPGM, VAV1, HBA-A1, PLSCR1, ID2, BAX, PLCG2, IRF8, IRF1, H2-DMA, RBPJ	2019	251	13588	1.82	2.63E-03	5.98E-05	1.22E-03
GOTERM_BP_FAT	GO:0048534~hemopoietic or lymphoid organ development	72	2.52	2.76E-06	XRCC5, GMPR2, G6PDX, STAT5B, PTPN22, RORC, IL15, NFKB2, TPD52, CBFB, TGFB1, ANK1, ALAS2, TNFRSF11A, CLCF1, WWP1, CASP8, HBB-B1, LTB, EGR1, SGPL1, STAP1, LYN, RELB, CDK6, NFAM1, MYH9, DNASE2A, BCL2L11, DCLRE1C, PGM3, HIF1A, LILRB3, CCR2, VEGFA, GADD45G, CCR1, SLC37A4, PML, SOX4, SFXN1, SFPI1, TTC7, KIT, IL7R, CD74, ADA, MLF1, RPA1, DOCK2, BCL2, PPP3CB, BCL3, RUNX1, HELLS, CEBPA, TRP53, PTPRC, IKZF1, EPAS1, HCLS1, BPGM, VAV1, HBA-A1, PLSCR1, ID2, BAX, PLCG2, IRF8, IRF1, H2-DMA, RBPJ	2019	281	13588	1.72	1.10E-02	2.17E-04	5.14E-03
GOTERM_BP_FAT	GO:0002520~immune system development	74	2.59	4.53E-06	XRCC5, GMPR2, G6PDX, STAT5B, RORC, PTPN22, IL15, NFKB2, TPD52, TGFB1, CBFB, ANK1, ALAS2, TNFRSF11A, CLCF1, WWP1, CASP8, HBB-B1, LTB, EGR1, SGPL1, ICOSL, STAP1, LYN, RELB, CDK6, NFAM1, MYH9, DNASE2A, BCL2L11, DCLRE1C, PGM3, HIF1A, LILRB3, CCR2, VEGFA, GADD45G, CCR1, SLC37A4, PML, SOX4, SFXN1, SFPI1, TTC7, KIT, IL7R, CD74, ADA, MLF1, RPA1, DOCK2, BCL2, PPP3CB, BCL3, RUNX1, HELLS, CEBPA, TRP53, PTPRC, IKZF1, EPAS1, SWAP70, HCLS1, BPGM, VAV1, HBA-A1, PLSCR1, ID2, BAX, PLCG2, IRF8, IRF1, H2-DMA, RBPJ	2019	295	13588	1.69	1.80E-02	3.29E-04	8.41E-03
GOTERM_BP_FAT	GO:0042098~T cell proliferation	15	0.53	1.24E-05	TRP53, ITGAL, PTPRC, ELF4, ITGB2, IL15, ITGAM, SLC11A1, DOCK2, CD86, P2RX7, ITGAX, FYN, CXCR4, BAX	2019	28	13588	3.61	4.85E-02	7.54E-04	2.31E-02

Annotation Cluster 6 Enrichment Score: 5.8345687099535395

Category	Term	Count	%	PValue	Genes	List Total	Pop Hits	Pop Total	Fold Enrichment	Bonferroni	Benjamini	FDR
GOTERM_BP_FAT	GO:0042330~taxis	43	1.51	1.09E-09	NRP2, C3AR1, CCL3, CCL2, S100A8, IL16, CYSLTR1, CCR1, CXCL3, SLC37A4, S100A9, CCL9, CCL8, CXCR2, ITGB2, PF4, FPR2, KIT, ITGAM, CCL7, CCL6, CCL24, CCL25, DOCK2, RAC2, CKLF, CSF3R, IL1B, FCER1G, PRKCA, C5AR1, FCGR3, CCL12, CORO1A, LYST, CXCL16, CX3CR1, ECSCR, CMTM7, AMOT, CMTM3, CMTM6, PTAFR	2019	109	13588	2.65	4.37E-06	3.36E-07	2.03E-06
GOTERM_BP_FAT	GO:0006935~chemotaxis	43	1.51	1.09E-09	NRP2, C3AR1, CCL3, CCL2, S100A8, IL16, CYSLTR1, CCR1, CXCL3, SLC37A4, S100A9, CCL9, CCL8, CXCR2, ITGB2, PF4, FPR2, KIT, ITGAM, CCL7, CCL6, CCL24, CCL25, DOCK2, RAC2, CKLF, CSF3R, IL1B, FCER1G, PRKCA, C5AR1, FCGR3, CCL12, CORO1A, LYST, CXCL16, CX3CR1, ECSCR, CMTM7, AMOT, CMTM3, CMTM6, PTAFR	2019	109	13588	2.65	4.37E-06	3.36E-07	2.03E-06
GOTERM_BP_FAT	GO:0030595~leukocyte chemotaxis	17	0.60	1.35E-07	PRKCA, C5AR1, IL16, CXCL3, CCR1, S100A9, SLC37A4, ITGB2, PF4, ITGAM, FCGR3, CORO1A, LYST, CX3CR1, FCER1G, IL1B, CSF3R	2019	27	13588	4.24	5.39E-04	1.93E-05	2.50E-04
GOTERM_BP_FAT	GO:0060326~cell chemotaxis	17	0.60	1.35E-07	PRKCA, C5AR1, IL16, CXCL3, CCR1, S100A9, SLC37A4, ITGB2, PF4, ITGAM, FCGR3, CORO1A, LYST, CX3CR1, FCER1G, IL1B, CSF3R	2019	27	13588	4.24	5.39E-04	1.93E-05	2.50E-04
GOTERM_BP_FAT	GO:0050900~leukocyte migration	20	0.70	3.73E-06	PRKCA, SELP, C5AR1, IL16, CXCL3, CCR1, SLC37A4, S100A9, ITGB2, PF4, ITGAM, FCGR3, CCL25, CORO1A, LYST, CX3CR1, FCER1G, IL1B, CSF3R, SELPLG	2019	43	13588	3.13	1.48E-02	2.82E-04	6.93E-03

Annotation Cluster 7 Enrichment Score: 5.573628774239573

Category	Term	Count	%	PValue	Genes	List Total	Pop Hits	Pop Total	Fold Enrichment	Bonferroni	Benjamini	FDR
GOTERM_BP_FAT	GO:0001817~regulation of cytokine production	50	1.75	1.66E-09	PPARG, STAT5B, TLR1, TLR2, TLR4, TLR6, TLR7, TLR8, TLR9, NOD2, MYD88, HMOX1, TICAM1, TICAM2, IL1B, CASP1, LTB, SCAMP5, BCL10, ICOSL, RELA, CD40, NLRP3, IFNAR1, PRKCQ, CD83, ADORA2B, PANX1, LRRC32, SLC37A4, TNFRSF4, SLC11A1, IRAK3, PYCARD, BCL3, ADAM33, FCER1G, INPP5D, SRGN, IL6, CARD9, H2-M3, SPHK1, FCGR3, DDX58, P2RX7, IRF1, CLEC7A, SASH3, CD14	2019	139	13588	2.42	6.63E-06	4.74E-07	3.08E-06

Annotation Cluster 8 Enrichment Score: 5.550600088199916

Category	Term	Count	%	PValue	Genes	List Total	Pop Hits	Pop Total	Fold Enrichment	Bonferroni	Benjamini	FDR
GOTERM_BP_FAT	GO:0002250~adaptive immune response	34	1.19	3.76E-08	NFKB2, TLR6, C1QC, CD74, SLC11A1, NOD2, KLHL6, MYD88, FCER1G, BCL3, C2, INPP5D, RAB27A, BCL10, ICAM1, ICOSL, C4B, SWAP70, RELB, FCGR1, PRKCD, FCGR3, C1QA, C1QB, IL18BP, UNC13D, FCGR2B, LILRB3, IRF7, LYST, VEGFA, GADD45G, H2-AA, H2-DMA	2019	84	13588	2.72	1.51E-04	7.17E-06	6.99E-05
GOTERM_BP_FAT	GO:0002460~adaptive immune response based on somatic recombination of immune receptors built fr	34	1.19	3.76E-08	NFKB2, TLR6, C1QC, CD74, SLC11A1, NOD2, KLHL6, MYD88, FCER1G, BCL3, C2, INPP5D, RAB27A, BCL10, ICAM1, ICOSL, C4B, SWAP70, RELB, FCGR1, PRKCD, FCGR3, C1QA, C1QB, IL18BP, UNC13D, FCGR2B, LILRB3, IRF7, LYST, VEGFA, GADD45G, H2-AA, H2-DMA	2019	84	13588	2.72	1.51E-04	7.17E-06	6.99E-05
GOTERM_BP_FAT	GO:0002252~immune effector process	43	1.51	1.46E-07	RSAD2, BNIP3, IL7R, TLR7, C1QC, TLR8, CD74, CFP, SLC11A1, NOD2, MYD88, TICAM1, BCL3, FCER1G, INPP5D, C2, CFD, RAB27A, PTPRC, ICAM1, PTPN6, BCL10, ICOSL, CARD9, C4B, NCF1, SWAP70, SAMHD1, MYO1F, NLRP3, FCGR1, PRKCD, FCGR3, C1QA, C1QB, LAT2, UNC13D, FCGR2B, LILRB3, LYST, IRF7, H2-AA, H2-DMA	2019	126	13588	2.30	5.85E-04	1.95E-05	2.72E-04
GOTERM_BP_FAT	GO:0002449~lymphocyte mediated immunity	28	0.98	6.23E-06	C1QC, CD74, SLC11A1, NOD2, MYD88, FCER1G, BCL3, C2, INPP5D, RAB27A, PTPN6, BCL10, ICAM1, ICOSL, C4B, SWAP70, PRKCD, FCGR1, FCGR3, C1QA, C1QB, UNC13D, FCGR2B, LILRB3, LYST, IRF7, H2-AA, H2-DMA	2019	76	13588	2.48	2.46E-02	4.30E-04	1.16E-02
GOTERM_BP_FAT	GO:0002443~leukocyte mediated immunity	31	1.09	6.62E-06	C1QC, CD74, SLC11A1, NOD2, MYD88, FCER1G, BCL3, C2, INPP5D, RAB27A, PTPN6, BCL10, ICAM1, ICOSL, C4B, SWAP70, NCF1, MYO1F, PRKCD, FCGR1, FCGR3, C1QA, C1QB, LAT2, UNC13D, FCGR2B, LILRB3, LYST, IRF7, H2-AA, H2-DMA	2019	89	13588	2.34	2.62E-02	4.49E-04	1.23E-02

Annotation Cluster 9 Enrichment Score: 5.433316786887672

Category	Term	Count	%	PValue	Genes	List Total	Pop Hits	Pop Total	Fold Enrichment	Bonferroni	Benjamini	FDR
GOTERM_BP_FAT	GO:0001817~regulation of cytokine production	50	1.75	1.66E-09	PPARG, STAT5B, TLR1, TLR2, TLR4, TLR6, TLR7, TLR8, TLR9, NOD2, MYD88, HMOX1, TICAM1, TICAM2, IL1B, CASP1, LTB, SCAMP5, BCL10, ICOSL, RELA, CD40, NLRP3, IFNAR1, PRKCQ, CD83, ADORA2B, PANX1, LRRC32, SLC37A4, TNFRSF4, SLC11A1, IRAK3, PYCARD, BCL3, ADAM33, FCER1G, INPP5D, SRGN, IL6, CARD9, H2-M3, SPHK1, FCGR3, DDX58, P2RX7, IRF1, CLEC7A, SASH3, CD14	2019	139	13588	2.42	6.63E-06	4.74E-07	3.08E-06

GOTERM_BP_FAT	GO:0001819~positive regulation of cytokine production	25	0.88	3.63E-06	PANX1, ADORA2B, TLR2, TLR4, TLR7, SLC11A1, NOD2, MYD88, TICAM1, PYCARD, FCER1G, IL1B, BCL3, CASP1, CARD9, H2-M3, CD40, NLRP3, IFNAR1, DDX58, CD83, P2RX7, CLEC7A, SASH3, CD14	2019	62	13588	2.71	1.44E-02	2.79E-04	6.74E-03
Annotation Cluster 10	Enrichment Score: 5.3785447785268605											
Category	Term	Count	%	PValue	Genes	List Total	Pop Hits	Pop Total	Fold Enrichment	Bonferroni	Benjamini	FDR
GOTERM_BP_FAT	GO:0048002~antigen processing and presentation of peptide antigen	21	0.74	8.51E-09	H2-Q10, H2-M3, H2-D1, UNC93B1, H2-DMB1, 2610524H06RIK, IFI30, H2-AB1, CALR, FCGR1, CD74, FCGR3, B2M, SLC11A1, FCGR2B, TAP2, H2-EB1, FCER1G, H2-AA, H2-T23, H2-DMA	2019	35	13588	4.04	3.41E-05	1.89E-06	1.58E-05
GOTERM_BP_FAT	GO:0002478~antigen processing and presentation of exogenous peptide antigen	16	0.56	5.39E-08	H2-M3, UNC93B1, 2610524H06RIK, IFI30, H2-DMB1, H2-AB1, FCGR1, CD74, B2M, FCGR3, FCGR2B, TAP2, H2-EB1, FCER1G, H2-AA, H2-DMA	2019	23	13588	4.68	2.16E-04	9.38E-06	1.00E-04
GOTERM_BP_FAT	GO:0019884~antigen processing and presentation of exogenous antigen	16	0.56	1.96E-06	H2-M3, UNC93B1, 2610524H06RIK, IFI30, H2-DMB1, H2-AB1, FCGR1, CD74, B2M, FCGR3, FCGR2B, TAP2, H2-EB1, FCER1G, H2-AA, H2-DMA	2019	28	13588	3.85	7.83E-03	1.60E-04	3.65E-03
GOTERM_BP_FAT	GO:0019886~antigen processing and presentation of exogenous peptide antigen via MHC class II	11	0.39	1.73E-05	FCGR2B, H2-EB1, UNC93B1, 2610524H06RIK, H2-AA, FCER1G, H2-DMB1, IFI30, H2-AB1, H2-DMA, CD74	2019	16	13588	4.63	6.69E-02	1.02E-03	3.21E-02
GOTERM_BP_FAT	GO:0002495~antigen processing and presentation of peptide antigen via MHC class II	11	0.39	1.73E-05	FCGR2B, H2-EB1, UNC93B1, 2610524H06RIK, H2-AA, FCER1G, H2-DMB1, IFI30, H2-AB1, H2-DMA, CD74	2019	16	13588	4.63	6.69E-02	1.02E-03	3.21E-02
Annotation Cluster 11	Enrichment Score: 5.149700998944686											
Category	Term	Count	%	PValue	Genes	List Total	Pop Hits	Pop Total	Fold Enrichment	Bonferroni	Benjamini	FDR
GOTERM_BP_FAT	GO:0012501~programmed cell death	107	3.75	5.42E-06	CADM1, PMAIP1, CRADD, SHB, CLPTM1L, APP, CUL7, NGFRAP1, DAP, CIB1, SGPL1, BCL10, NCF1, MADD, FADD, TNFRSF14, STK4, BCL2L11, STK3, TRIM35, KRT18, TNFAIP8, RIPK3, PDCD6IP, MAPK7, TNFAIP3, EIF2AK3, TRAF1, LST1, LITAF, TNFRSF12A, STK17B, ARF6, PPT1, BCL2L1, SP110, IRAK3, PRUNE2, FAIM, SRGN, TRP53, CKAP2, RYBP, VDAC1, CIDEC, TRADD, BBC3, CSRNP1, E2F1, NUAK2, BNIP3, CXCR2, CASP6, TMEM173, CASP3, CASP4, PAK2, CASP9, CASP8, TICAM1, CASP1, LTB, MYC, IRAK2, SGK1, CYCS, NLRP3, DNASE2A, DAPK1, ELMO1, TNFRSF10B, RIPK1, GADD45G, SH3KBP1, ERN1, MFSD10, NGFR, PPP1R15A, BID, UBE2Z, PML, TRIB3, KIT, FEM1B, TNFRSF1A, NAIP2, BCL2, NAIP5, SHISA5, KRT8, PYCARD, ZC3H12A, INPP5D, XAF1, PHLDA1, ACTC1, IL6, AIMP2, EDA2R, PEA15A, PPP1R13L, RASSF5, BAX, ENDOG, APAF1, APIP, DRAM1	2019	473	13588	1.52	2.15E-02	3.87E-04	1.01E-02
GOTERM_BP_FAT	GO:0008219~cell death	113	3.96	5.90E-06	CADM1, PMAIP1, TGFB1, CRADD, CLPTM1L, SHB, APP, CUL7, NGFRAP1, DAP, CIB1, SGPL1, BCL10, NCF1, MADD, FADD, TNFRSF14, STK4, BCL2L11, STK3, TRIM35, KRT18, TNFAIP8, RIPK3, PDCD6IP, MAPK7, TNFAIP3, EIF2AK3, TRAF1, LST1, LITAF, TNFRSF12A, CLU, STK17B, ARF6, PPT1, BCL2L1, SP110, IRAK3, PRUNE2, FGL2, FAIM, SRGN, TRP53, CKAP2, RYBP, VDAC1, CIDEC, TRADD, P2RX7, BBC3, CSRNP1, E2F1, NUAK2, BNIP3, CXCR2, CASP6, TMEM173, CASP3, CASP4, PAK2, CASP9, CASP8, TICAM1, CASP1, MYC, LTB, IRAK2, SGK1, CYCS, NLRP3, DNASE2A, DAPK1, ELMO1, TNFRSF10B, RIPK1, GADD45G, SH3KBP1, ERN1, MFSD10, NGFR, PPP1R15A, BID, UBE2Z, PML, TRIB3, KIT, FEM1B, TNFRSF1A, TNFRSF1B, NAIP2, BCL2, NAIP5, SHISA5, KRT8, PYCARD, ZC3H12A, INPP5D, XAF1, PHLDA1, IL6, LYZ2, ACTC1, AIMP2, EDA2R, PEA15A, PPP1R13L, RASSF5, BAX, ENDOG, APAF1, APIP, DRAM1	2019	507	13588	1.50	2.33E-02	4.14E-04	1.10E-02
GOTERM_BP_FAT	GO:0006915~apoptosis	105	3.68	7.21E-06	CADM1, PMAIP1, CRADD, SHB, CLPTM1L, APP, CUL7, NGFRAP1, DAP, CIB1, SGPL1, BCL10, MADD, NCF1, FADD, TNFRSF14, STK4, BCL2L11, STK3, TRIM35, KRT18, TNFAIP8, RIPK3, PDCD6IP, MAPK7, TNFAIP3, EIF2AK3, TRAF1, LST1, LITAF, TNFRSF12A, STK17B, ARF6, PPT1, SP110, BCL2L1, IRAK3, PRUNE2, FAIM, SRGN, TRP53, CKAP2, RYBP, VDAC1, CIDEC, TRADD, BBC3, CSRNP1, E2F1, NUAK2, BNIP3, CXCR2, CASP6, TMEM173, CASP3, CASP4, PAK2, CASP9, CASP8, TICAM1, CASP1, LTB, MYC, IRAK2, SGK1, CYCS, NLRP3, DNASE2A, DAPK1, ELMO1, TNFRSF10B, RIPK1, GADD45G, SH3KBP1, ERN1, MFSD10, NGFR, PPP1R15A, BID, UBE2Z, PML, TRIB3, FEM1B, TNFRSF1A, NAIP2, BCL2, NAIP5, SHISA5, KRT8, PYCARD, ZC3H12A, INPP5D, XAF1, PHLDA1, ACTC1, IL6, AIMP2, PEA15A, PPP1R13L, RASSF5, BAX, ENDOG, APAF1, APIP, DRAM1	2019	465	13588	1.52	2.85E-02	4.81E-04	1.34E-02
GOTERM_BP_FAT	GO:0016265~death	114	3.99	1.09E-05	CADM1, PMAIP1, TGFB1, CRADD, CLPTM1L, SHB, APP, CUL7, NGFRAP1, DAP, CIB1, SGPL1, BCL10, NCF1, MADD, FADD, TNFRSF14, STK4, BCL2L11, STK3, TRIM35, KRT18, TNFAIP8, RIPK3, PDCD6IP, MAPK7, TNFAIP3, EIF2AK3, TRAF1, LST1, LITAF, TNFRSF12A, CLU, STK17B, ARF6, PPT1, BCL2L1, SP110, IRAK3, PRUNE2, FGL2, FAIM, SRGN, TRP53, CKAP2, RYBP, VDAC1, CIDEC, TRADD, P2RX7, BBC3, CSRNP1, TCF15, E2F1, NUAK2, BNIP3, CXCR2, CASP6, TMEM173, CASP3, CASP4, PAK2, CASP9, CASP8, TICAM1, CASP1, MYC, LTB, IRAK2, SGK1, CYCS, NLRP3, DNASE2A, DAPK1, ELMO1, TNFRSF10B, RIPK1, GADD45G, SH3KBP1, ERN1, MFSD10, NGFR, PPP1R15A, BID, UBE2Z, PML, TRIB3, KIT, FEM1B, TNFRSF1A, TNFRSF1B, NAIP2, BCL2, NAIP5, SHISA5, KRT8, PYCARD, ZC3H12A, INPP5D, XAF1, PHLDA1, IL6, LYZ2, ACTC1, AIMP2, EDA2R, PEA15A, PPP1R13L, RASSF5, BAX, ENDOG, APAF1, APIP, DRAM1	2019	519	13588	1.48	4.28E-02	6.84E-04	2.03E-02
Annotation Cluster 12	Enrichment Score: 4.768117672113028											
Category	Term	Count	%	PValue	Genes	List Total	Pop Hits	Pop Total	Fold Enrichment	Bonferroni	Benjamini	FDR
GOTERM_MF_FAT	GO:0032559~adenyl ribonucleotide binding	281	9.84	1.66E-07	XRCC5, KIFC2, KIFC1, ADCY7, AURKB, C230081A13RIK, PRKX, BTK, DHX37, OASL2, OASL1, MAP3K8, DHX34, VPS4B, DHX33, DDX10, MAP2K6, PIK3CG, BCR, MYLK4, MYLK2, MYH7, WNK2, MYH9, MYH8, CARNS1, RFC5, RFC3, MAPK6, ACAP1, PDGFRA, MAPK7, EIF2AK3, CHKA, PFKFB3, PFKFB1, OAS3, STK17B, CHEK1, MYO9B, HSPA1A, OAS2, HSPA1B, TK2, MOV10, MUSK, DHX58, KIF3B, SPHK1, TAOK3, UBE2L6, ABCG3, KIF3C, ATP13A5, EPHA2, ABCG1, HYOU1, ATP2A2, HSPA13, GRK5, CLCN5, NUAK2, MYO7A, ATP10A, CAMKK1, CAMKK2, CKB, ACTR3, ACTR2, MCCC2, FANCM, NOD2, SLC22A4, EEF2K, SIK1, TOP2A, TARSL2, CDC7, LYN, PHKG1, PKDCC, PDE10A, MAST3, ADK, RIPK1, EIF4A1, MAP3K14, MYO5A, ALDH18A1, MKNK2, HK1, KIT, GALK2, GALK1, MORC4, PTK2B, HK3, ABCD2, PRKAA1, CERK, PAPSS2, TRIP13, ACSL5, MYO5C, PDK2, ACTC1, SWAP70, PDK3, PDK4, ATP1A3, AXL, ACLY, NADK, ATP1A1, APRT, KHK, RPS6KA5, RPS6KA4, RPS6KA1, FYN, ARAF, ABCC3, GYK, PGK1, RNASEL, CTPS, F13A1, ITPKC, PI4K2B, MTHFD1L, DDX27, PRKAR2B, MLKL, STK39, DDX21, MATK, EGFR, TWF2, PDXK, PNCK, STK4, STK3, MAP4K4, DDX39, ATP9A, PI4K2A, RIPK3, OAS1A, LRRK1, OAS1G, PCCA, EP400, PRPS2, SRXN1, IFIH1, FGR, ASS1, ACTR3B, STK10, MAP4K1, CTPS2, TIMP4, ABCA1, IRAK4, IRAK3, UBE2D1, ENTPD1, RUNX1, HELLS, CSF1R, TEC, RHOBTB3, TRP53, ABCB9, MYO1C, AK1, MYO1G, ATAD2, MYO1F, ABCB7, PSMB8, MMAB, PSMB9, DDX58, IKBKE, P2RX4, DNA2, P2RX7, DDX56, PLK4, P2RX6, PLK3, PLK2, ULK1, ULK2, DYRK1B, MERTK, PIP4K2A, UBE2E1, CDK19, KIF22, CDK17, FIGNL1, SLFN9, PPIP5K2, SLFN8, CAD, FES, SLFN4, SLFN1, SLFN2, ACVR1B, PAK2, PAK4, ATP8B1, ATP8B2, MKKS, CSK, MDN1, CDK14, ATP8B4, PRKCA, IRAK2, CDK1, SGK1, KIF11, HSP90AA1, LIMK1, PRKCI, CDK6, PFKM, PBK, UBE2H, MCM3, NLRP3, PRKCE, PRKCD, MCM4, UBE2B, CDK2, MCM5, DAPK1, PRKCB, MCM6, PANK4, ATP6V1A, PRKCQ, KHSRP, ERN1, H2-AA, CAMK1D, UBE2Z, DCK, TRIB3, ASNS, TRIB1, IGF1R, NAIP2, TAP2, TAP1, TOR1A, PIKFYVE, CAMK2B, UCK2, HSPA5, EHD1, CAMK2A, DCLK1, EHD4, AMHR2, ACTB, ALPK1, HCK, KIF18A, SMC2, PCX, HSP90B1, ATP8A2, APAF1, IPPK, H2-DMA, CDK20	1935	1460	13288	1.32	2.35E-04	1.96E-05	2.74E-04
GOTERM_MF_FAT	GO:0005524~ATP binding	278	9.73	1.79E-07	XRCC5, KIFC2, KIFC1, ADCY7, AURKB, C230081A13RIK, PRKX, BTK, DHX37, OASL2, OASL1, MAP3K8, DHX34, VPS4B, DHX33, DDX10, MAP2K6, PIK3CG, BCR, MYLK4, MYLK2, MYH7, WNK2, MYH9, MYH8, CARNS1, RFC5, RFC3, MAPK6, ACAP1, PDGFRA, MAPK7, EIF2AK3, CHKA, PFKFB3, PFKFB1, OAS3, STK17B, CHEK1, MYO9B, HSPA1A, OAS2, HSPA1B, TK2, MOV10, MUSK, DHX58, KIF3B, SPHK1, TAOK3, UBE2L6, ABCG3, KIF3C, ATP13A5, EPHA2, ABCG1, HYOU1, ATP2A2, HSPA13, GRK5, CLCN5, NUAK2, MYO7A, ATP10A, CAMKK1, CAMKK2, CKB, ACTR3, ACTR2, MCCC2, FANCM, NOD2, SLC22A4, EEF2K, SIK1, TOP2A, TARSL2, CDC7, LYN, PHKG1, PKDCC, MAST3, ADK, RIPK1, EIF4A1, MAP3K14, MYO5A, ALDH18A1, MKNK2, HK1, KIT, GALK2, GALK1, MORC4, PTK2B, HK3, ABCD2, PRKAA1, CERK, PAPSS2, TRIP13, ACSL5, MYO5C, PDK2, ACTC1, SWAP70, PDK3, PDK4, ATP1A3, AXL, ACLY, NADK, ATP1A1, KHK, RPS6KA5, RPS6KA4, RPS6KA1, FYN, ARAF, ABCC3, GYK, PGK1, RNASEL, CTPS, F13A1, ITPKC, PI4K2B, MTHFD1L, DDX27, MLKL, STK39, DDX21, MATK, EGFR, TWF2, PDXK, PNCK, STK4, STK3, MAP4K4, DDX39, ATP9A, PI4K2A, RIPK3, OAS1A, LRRK1, OAS1G, PCCA, EP400, PRPS2, SRXN1, IFIH1, FGR, ASS1, ACTR3B, STK10, MAP4K1, CTPS2, TIMP4, ABCA1, IRAK4, IRAK3, UBE2D1, ENTPD1, RUNX1, HELLS, CSF1R, TEC, RHOBTB3, TRP53, ABCB9, MYO1C, AK1, MYO1G, ATAD2, MYO1F, ABCB7, PSMB8, MMAB, PSMB9, DDX58, IKBKE, P2RX4, DNA2, P2RX7, DDX56, PLK4, P2RX6, PLK3, PLK2, ULK1, ULK2, DYRK1B, MERTK, PIP4K2A, UBE2E1, CDK19, KIF22, CDK17, FIGNL1, SLFN9, PPIP5K2, SLFN8, CAD, FES, SLFN4, SLFN1, SLFN2, ACVR1B, PAK2, PAK4, ATP8B1, ATP8B2, MKKS, CSK, MDN1, CDK14, ATP8B4, PRKCA, IRAK2, CDK1, SGK1, KIF11, HSP90AA1, LIMK1, PRKCI, CDK6, PFKM, PBK, UBE2H, MCM3, NLRP3, PRKCE, PRKCD, UBE2B, MCM4, CDK2, MCM5, DAPK1, PRKCB, MCM6, PANK4, ATP6V1A, PRKCQ, KHSRP, ERN1, H2-AA, CAMK1D, UBE2Z, DCK, TRIB3, ASNS, TRIB1, IGF1R, NAIP2, TAP2, TAP1, TOR1A, PIKFYVE, CAMK2B, UCK2, HSPA5, EHD1, CAMK2A, DCLK1, EHD4, AMHR2, ACTB, ALPK1, HCK, KIF18A, SMC2, PCX, HSP90B1, ATP8A2, APAF1, IPPK, H2-DMA, CDK20	1935	1443	13288	1.32	2.54E-04	1.81E-05	2.96E-04
GOTERM_BP_FAT	GO:0006796~phosphate metabolic process	177	6.20	4.18E-06	RNASEL, CAPZA2, PTPN23, PTPN22, RORC, AURKB, LPAR1, C230081A13RIK, TGFB1, PRKX, DUPD1, BTK, PRKAR2B, APP, MDFIC, MAP3K8, MLKL, STK39, MAP2K6, MATK, PTPRJ, PIK3CG, EGFR, BCR, MADD, PNCK, MYLK4, PTPRS, MYLK2, WNK2, STK4, PTPRO, STK3, MAP4K4, MAPK6, ACAP1, SLC37A2, PDGFRA, RIPK3, MAPK7, EIF2AK3, LRRK1, GNAI2, FGR, SSH3, STK10, STK17B, MAP4K1, CHEK1, CD74, IRAK4, IRAK3, MUSK, ATP6V0E, DUSP19, PTPLB, PTPLA, CSF1R, TEC, PTPN7, GPD2, MDFI, PTPN6, GPD1, PTPN9, PTPN18, PTPN2, TAOK3, DUSP23, PTPN12, EPHA2, IKBKE, DUSP28, PLK4, P2RX7, PLK3, PLK2, ULK1, DYRK1B, ULK2, PTPN1, GRK5, MERTK, CDK19, CDK17, NUAK2, SYNJ1, CAD, FES, CAMKK1, CAMKK2, ACVR1B, CCNE1, PAK2, PAK4, EEF2K, SYNJ2, SHC1, CSK, SIK1, CDK14, ATP5J, PRKCA, IRAK2, CDC7, CDK1, SGK1, LYN, PHKG1, LIMK1, NDUFC2, PRKCI, ATP6V1H, CDK6, PKDCC, PBK, PRKCE, PRKCD, CDK2, DAPK1, PRKCB, MAST3, ATP6V1A, PRKCQ, CCND1, PPM1J, RIPK1, PPM1H, GADD45G, CFL1, ERN1, PPM1L, PPM1M, MAP3K14, UQCRB, CAMK1D, PML, MKNK2, TRIB3, ABI2, KIT, TRIB1, GALK2, GALK1, IGF1R, PTK2B, BCL2, PPP3CB, PRKAA1, CAMK2B, CAMK2A, DCLK1, TES, AMHR2, TCIRG1, PTPRC, PDK2, ALPK1, PTPRE, PDK3, HCK, PDK4, AXL, TRIM24, DUSP5, RPS6KA5, DUSP4, RPS6KA4, DUSP2, RPS6KA1, FYN, ARAF, GYK, IPPK, PGK1, CDK20, DUSP6	2019	866	13588	1.38	1.66E-02	3.10E-04	7.77E-03
GOTERM_BP_FAT	GO:0006793~phosphorus metabolic process	177	6.20	4.18E-06	RNASEL, CAPZA2, PTPN23, PTPN22, RORC, AURKB, LPAR1, C230081A13RIK, TGFB1, PRKX, DUPD1, BTK, PRKAR2B, APP, MDFIC, MAP3K8, MLKL, STK39, MAP2K6, MATK, PTPRJ, PIK3CG, EGFR, BCR, MADD, PNCK, MYLK4, PTPRS, MYLK2, WNK2, STK4, PTPRO, STK3, MAP4K4, MAPK6, ACAP1, SLC37A2, PDGFRA, RIPK3, MAPK7, EIF2AK3, LRRK1, GNAI2, FGR, SSH3, STK10, STK17B, MAP4K1, CHEK1, CD74, IRAK4, IRAK3, MUSK, ATP6V0E, DUSP19, PTPLB, PTPLA, CSF1R, TEC, PTPN7, GPD2, MDFI, PTPN6, GPD1, PTPN9, PTPN18, PTPN2, TAOK3, DUSP23, PTPN12, EPHA2, IKBKE, DUSP28, PLK4, P2RX7, PLK3, PLK2, ULK1, DYRK1B, ULK2, PTPN1, GRK5, MERTK, CDK19, CDK17, NUAK2, SYNJ1, CAD, FES, CAMKK1, CAMKK2, ACVR1B, CCNE1, PAK2, PAK4, EEF2K, SYNJ2, SHC1, CSK, SIK1, CDK14, ATP5J, PRKCA, IRAK2, CDC7, CDK1, SGK1, LYN, PHKG1, LIMK1, NDUFC2, PRKCI, ATP6V1H, CDK6, PKDCC, PBK, PRKCE, PRKCD, CDK2, DAPK1, PRKCB, MAST3, ATP6V1A, PRKCQ, CCND1, PPM1J, RIPK1, PPM1H, GADD45G, CFL1, ERN1, PPM1L, PPM1M, MAP3K14, UQCRB, CAMK1D, PML, MKNK2, TRIB3, ABI2, KIT, TRIB1, GALK2, GALK1, IGF1R, PTK2B, BCL2, PPP3CB, PRKAA1, CAMK2B, CAMK2A, DCLK1, TES, AMHR2, TCIRG1, PTPRC, PDK2, ALPK1, PTPRE, PDK3, HCK, PDK4, AXL, TRIM24, DUSP5, RPS6KA5, DUSP4, RPS6KA4, DUSP2, RPS6KA1, FYN, ARAF, GYK, IPPK, PGK1, CDK20, DUSP6	2019	866	13588	1.38	1.66E-02	3.10E-04	7.77E-03
Annotation Cluster 13	Enrichment Score: 4.524001805935109											
Category	Term	Count	%	PValue	Genes	List Total	Pop Hits	Pop Total	Fold Enrichment	Bonferroni	Benjamini	FDR
GOTERM_BP_FAT	GO:0007264~small GTPase mediated signal transduction	72	2.52	8.44E-08	GNA13, WASF2, RASGEF1B, IQGAP2, RRAD, RHOV, IQGAP1, RGL1, ARL11, TIAM1, HMOX1, ARHGAP1, RALB, ARL5C, RHOF, RAB20, RHOG, RAB27A, RAP2B, RAP2A, DBNL, RAB4A, BAIAP2, RAB4B, GEM, ARHGEF9, ELMO1, ARL3, RND3, DOK1, DOK2, DOK3, HRASLS, RRAS2, SDCBP, RAB15, RAB12, ARL8A, ARL4D, LRRK1, ARL4C, RASD2, 5430435G22RIK, RAB3A, CDC42SE1, ARF6, RAB40B, RAB44, RAB43, RASGRP3, RAC2, RASGRP4, RHOBTB2, CDC42EP4, ARHGDIA, CNKSR1, RAB8A, VAV3, RAB8B, RAB7L1, TAX1BP3, RAB33B, RAB32, RAB31, ULK1, RASSF1, ARF3, ARF4, RAB34, YWHAQ, RAP1B, BCAR3	2019	258	13588	1.88	3.38E-04	1.30E-05	1.57E-04
Annotation Cluster 14	Enrichment Score: 4.382199869781188											
Category	Term	Count	%	PValue	Genes	List Total	Pop Hits	Pop Total	Fold Enrichment	Bonferroni	Benjamini	FDR

GOTERM_BP_FAT	GO:0016044~membrane organization	72	2.52	7.66E-07	CD209A, MSR1, LDLR, MYO7A, SYNJ1, WASF2, BNIP3, PMAIP1, DAB2, APP, CTTN, DYSF, CAP1, DHCR24, GHR, CLN3, DBNL, ARC, CCDC88A, PRKCI, LPCAT2, LDLRAP1, SIRPA, TIMM8B, ELMO1, UNC13D, LRP12, SH3KBP1, SORT1, RIN1, ANKFY1, STON2, BID, RAB3A, CDC42SE1, PPT1, ABCA1, CD9, SLC11A1, DOCK2, BCL2, FCER1G, EHD1, MRC1, MICALL2, TRP53, VAV3, A4GALT, HCK, MRC2, LMNA, SUN2, ITGA3, STAB2, VAV1, FCGR1, FCGR3, CORO1C, P2RX7, LRP1, TFRC, FCGR2B, ULK1, BAX, RAB34, IRF8, CLEC7A, CSTAD, MERTK, SCARA5, CLCN5, VLDLR	2019	272	13588	1.78	3.06E-03	6.82E-05	1.42E-03
GOTERM_BP_FAT	GO:0010324~membrane invagination	52	1.82	8.43E-06	CD209A, MSR1, LDLR, MYO7A, WASF2, SYNJ1, DAB2, CTTN, APP, CAP1, GHR, DBNL, CLN3, ARC, SIRPA, LDLRAP1, ELMO1, UNC13D, LRP12, SH3KBP1, RIN1, SORT1, ANKFY1, STON2, RAB3A, CDC42SE1, PPT1, ABCA1, SLC11A1, FCER1G, EHD1, MRC1, MICALL2, HCK, MRC2, STAB2, FCGR1, VAV1, FCGR3, CORO1C, P2RX7, LRP1, FCGR2B, TFRC, ULK1, RAB34, IRF8, CLEC7A, MERTK, SCARA5, CLCN5, VLDLR	2019	188	13588	1.86	3.32E-02	5.45E-04	1.57E-02
GOTERM_BP_FAT	GO:0006897~endocytosis	52	1.82	8.43E-06	CD209A, MSR1, LDLR, MYO7A, WASF2, SYNJ1, DAB2, CTTN, APP, CAP1, GHR, DBNL, CLN3, ARC, SIRPA, LDLRAP1, ELMO1, UNC13D, LRP12, SH3KBP1, RIN1, SORT1, ANKFY1, STON2, RAB3A, CDC42SE1, PPT1, ABCA1, SLC11A1, FCER1G, EHD1, MRC1, MICALL2, HCK, MRC2, STAB2, FCGR1, VAV1, FCGR3, CORO1C, P2RX7, LRP1, FCGR2B, TFRC, ULK1, RAB34, IRF8, CLEC7A, MERTK, SCARA5, CLCN5, VLDLR	2019	188	13588	1.86	3.32E-02	5.45E-04	1.57E-02
Annotation Cluster 15	Enrichment Score: 4.304883282557456											
Category	Term	Count	%	PValue	Genes	List Total	Pop Hits	Pop Total	Fold Enrichment	Bonferroni	Benjamini	FDR
GOTERM_BP_FAT	GO:0043068~positive regulation of programmed cell death	69	2.42	2.55E-07	PDIA3, MMP9, BNIP3, PMAIP1, TGFB1, CASP6, CASP3, CASP4, CASP9, APOE, HMOX1, TICAM1, POU4F1, NGFRAP1, RARB, CASP1, NDUFS3, MYC, LTB, RAB27A, PRKCA, BCL10, NUDT2, CYCS, SKP2, INHA, GAL, PRKCD, STK3, BCL2L11, DAPK1, TRIM35, UNC13D, RIPK3, ERN1, NGFR, BID, LST1, NFKBID, STK17B, PML, BCL2L1, SP110, ZC3H8, GCH1, ALDH1A1, SHISA5, PYCARD, BCL3, INPP5D, PLAGL2, COL18A1, TRP53, PTPRC, PTPN6, NR4A1, FCGR1, FCGR3, CIDEC, TRADD, NOTCH2, P2RX7, CDKN1A, NOTCH1, BBC3, BAX, ENDOG, APAF1, IFI204	2019	250	13588	1.86	1.02E-03	3.19E-05	4.73E-04
GOTERM_BP_FAT	GO:0043067~regulation of programmed cell death	128	4.48	3.24E-07	XRCC5, MMP9, STAT5B, PMAIP1, PNP, CRADD, TGFB1, CUL7, APOE, NGFRAP1, RARB, RAB27A, PIK3CG, BCL10, NUDT2, ACTN4, MADD, SKP2, FADD, BCL2L11, STK3, TRIM35, HIF1A, UNC13D, VEGFA, CX3CR1, RIPK3, EIF2AK3, TRAF1, IFIH1, LST1, SYVN1, NFKBID, CLU, STK17B, HSPA1A, PPT1, BCL2L1, HSPA1B, SP110, SOX9, ADA, CD74, ZC3H8, IRAK3, FCER1G, GLO1, FAIM, HELLS, PLAGL2, ANGPTL4, SPP1, TRP53, COL18A1, PTPN6, CARD9, EEF1A2, SPHK1, NR4A1, FCGR1, FCGR3, TRADD, CIDEC, NOTCH2, CDKN1A, NOTCH1, P2RX7, BCL2A1B, BBC3, PLCG2, CFDP1, PDIA3, NUAK2, BNIP3, CASP6, NOD2, CASP3, CASP4, CASP9, HMOX1, CASP8, TICAM1, CHST11, POU4F1, DLG5, CASP1, NDUFS3, MYC, LTB, DHCR24, PRKCA, IRAK2, CLN3, CYCS, INHA, NLRP3, GAL, PRKCD, UBE2B, DAPK1, SMO, CARD14, TNFRSF10B, BTG2, BTG1, ERN1, NGFR, CAMK1D, BID, TNFRSF22, PML, KIT, TNFRSF4, GCH1, ALDH1A1, BCL2, SHISA5, PYCARD, BCL3, INPP5D, PTPRC, IL6, PEA15A, YWHAH, BAX, ENDOG, APAF1, APIP, IFI204	2019	560	13588	1.54	1.29E-03	3.81E-05	6.01E-04
GOTERM_BP_FAT	GO:0010942~positive regulation of cell death	69	2.42	3.52E-07	PDIA3, MMP9, BNIP3, PMAIP1, TGFB1, CASP6, CASP3, CASP4, CASP9, APOE, HMOX1, TICAM1, POU4F1, NGFRAP1, RARB, CASP1, NDUFS3, MYC, LTB, RAB27A, PRKCA, BCL10, NUDT2, CYCS, SKP2, INHA, GAL, PRKCD, STK3, BCL2L11, DAPK1, TRIM35, UNC13D, RIPK3, ERN1, NGFR, BID, LST1, NFKBID, STK17B, PML, BCL2L1, SP110, ZC3H8, GCH1, ALDH1A1, SHISA5, PYCARD, BCL3, INPP5D, PLAGL2, COL18A1, TRP53, PTPRC, PTPN6, NR4A1, FCGR1, FCGR3, CIDEC, TRADD, NOTCH2, P2RX7, CDKN1A, NOTCH1, BBC3, BAX, ENDOG, APAF1, IFI204	2019	252	13588	1.84	1.41E-03	4.02E-05	6.54E-04
GOTERM_BP_FAT	GO:0043065~positive regulation of apoptosis	68	2.38	4.08E-07	PDIA3, MMP9, BNIP3, PMAIP1, TGFB1, CASP6, CASP3, CASP4, CASP9, APOE, HMOX1, TICAM1, POU4F1, NGFRAP1, RARB, NDUFS3, CASP1, MYC, LTB, RAB27A, PRKCA, BCL10, NUDT2, CYCS, SKP2, INHA, GAL, PRKCD, STK3, BCL2L11, DAPK1, TRIM35, UNC13D, RIPK3, ERN1, NGFR, BID, LST1, NFKBID, STK17B, PML, BCL2L1, SP110, ZC3H8, GCH1, ALDH1A1, SHISA5, PYCARD, BCL3, INPP5D, PLAGL2, COL18A1, TRP53, PTPRC, PTPN6, NR4A1, FCGR1, FCGR3, CIDEC, TRADD, NOTCH2, P2RX7, NOTCH1, BBC3, BAX, ENDOG, APAF1, IFI204	2019	248	13588	1.85	1.63E-03	4.42E-05	7.59E-04
GOTERM_BP_FAT	GO:0010941~regulation of cell death	128	4.48	4.38E-07	XRCC5, MMP9, STAT5B, PMAIP1, PNP, CRADD, TGFB1, CUL7, APOE, NGFRAP1, RARB, RAB27A, PIK3CG, BCL10, NUDT2, ACTN4, MADD, SKP2, FADD, BCL2L11, STK3, TRIM35, HIF1A, UNC13D, VEGFA, CX3CR1, RIPK3, EIF2AK3, TRAF1, IFIH1, LST1, SYVN1, NFKBID, CLU, STK17B, HSPA1A, PPT1, BCL2L1, HSPA1B, SP110, SOX9, ADA, CD74, ZC3H8, IRAK3, FCER1G, GLO1, FAIM, HELLS, PLAGL2, ANGPTL4, SPP1, TRP53, COL18A1, PTPN6, CARD9, EEF1A2, SPHK1, NR4A1, FCGR1, FCGR3, TRADD, CIDEC, NOTCH2, CDKN1A, NOTCH1, P2RX7, BCL2A1B, BBC3, PLCG2, CFDP1, PDIA3, NUAK2, BNIP3, CASP6, NOD2, CASP3, CASP4, CASP9, HMOX1, CASP8, TICAM1, CHST11, POU4F1, DLG5, CASP1, NDUFS3, MYC, LTB, DHCR24, PRKCA, IRAK2, CLN3, CYCS, INHA, NLRP3, GAL, PRKCD, UBE2B, DAPK1, SMO, CARD14, TNFRSF10B, BTG2, BTG1, ERN1, NGFR, CAMK1D, BID, TNFRSF22, PML, KIT, TNFRSF4, GCH1, ALDH1A1, BCL2, SHISA5, PYCARD, BCL3, INPP5D, PTPRC, IL6, PEA15A, YWHAH, BAX, ENDOG, APAF1, APIP, IFI204	2019	563	13588	1.53	1.75E-03	4.61E-05	8.14E-04
GOTERM_BP_FAT	GO:0042981~regulation of apoptosis	126	4.41	4.81E-07	XRCC5, MMP9, STAT5B, PMAIP1, PNP, CRADD, TGFB1, CUL7, APOE, NGFRAP1, RARB, RAB27A, PIK3CG, BCL10, NUDT2, ACTN4, MADD, SKP2, FADD, BCL2L11, STK3, TRIM35, HIF1A, UNC13D, VEGFA, CX3CR1, RIPK3, EIF2AK3, TRAF1, IFIH1, LST1, SYVN1, NFKBID, CLU, STK17B, HSPA1A, PPT1, BCL2L1, HSPA1B, SP110, SOX9, ADA, CD74, ZC3H8, IRAK3, FCER1G, GLO1, FAIM, HELLS, ANGPTL4, SPP1, PLAGL2, TRP53, COL18A1, PTPN6, CARD9, EEF1A2, SPHK1, NR4A1, FCGR1, FCGR3, TRADD, CIDEC, NOTCH2, CDKN1A, NOTCH1, P2RX7, BCL2A1B, BBC3, CFDP1, PDIA3, NUAK2, BNIP3, CASP6, NOD2, CASP3, CASP4, CASP9, HMOX1, CASP8, TICAM1, CHST11, POU4F1, DLG5, CASP1, NDUFS3, MYC, LTB, DHCR24, PRKCA, IRAK2, CLN3, CYCS, INHA, NLRP3, GAL, PRKCD, UBE2B, DAPK1, SMO, CARD14, TNFRSF10B, BTG2, BTG1, ERN1, NGFR, CAMK1D, BID, TNFRSF22, PML, TNFRSF4, GCH1, ALDH1A1, BCL2, SHISA5, PYCARD, BCL3, INPP5D, PTPRC, IL6, PEA15A, YWHAH, BAX, ENDOG, APAF1, APIP, IFI204	2019	553	13588	1.53	1.93E-03	4.70E-05	8.95E-04
Annotation Cluster 16	Enrichment Score: 4.139925294215899											
Category	Term	Count	%	PValue	Genes	List Total	Pop Hits	Pop Total	Fold Enrichment	Bonferroni	Benjamini	FDR
GOTERM_BP_FAT	GO:0002366~leukocyte activation during immune response	16	0.56	1.01E-05	TRP53, SBNO2, H2-M3, RELB, MYO1F, ADA, SLC11A1, LAT2, UNC13D, TICAM1, PLCG2, GADD45G, CX3CR1, BCL3, LCP1, RAB27A	2019	31	13588	3.47	3.97E-02	6.43E-04	1.88E-02
GOTERM_BP_FAT	GO:0002263~cell activation during immune response	16	0.56	1.01E-05	TRP53, SBNO2, H2-M3, RELB, MYO1F, ADA, SLC11A1, LAT2, UNC13D, TICAM1, PLCG2, GADD45G, CX3CR1, BCL3, LCP1, RAB27A	2019	31	13588	3.47	3.97E-02	6.43E-04	1.88E-02
Annotation Cluster 17	Enrichment Score: 3.860978297723781											
Category	Term	Count	%	PValue	Genes	List Total	Pop Hits	Pop Total	Fold Enrichment	Bonferroni	Benjamini	FDR
GOTERM_BP_FAT	GO:0002237~response to molecule of bacterial origin	23	0.81	4.58E-07	BCL10, C5AR1, CARD9, H2-M3, LY96, RELA, TLR1, TLR2, NFKBIA, TLR4, B2M, IRAK3, SLC11A1, NOD2, P2RX7, MYD88, THBD, TAP2, TICAM1, PLCG2, IL1B, CD14, PTAFR	2019	49	13588	3.16	1.83E-03	4.70E-05	8.52E-04
Annotation Cluster 18	Enrichment Score: 3.8236958872743068											
Category	Term	Count	%	PValue	Genes	List Total	Pop Hits	Pop Total	Fold Enrichment	Bonferroni	Benjamini	FDR
GOTERM_BP_FAT	GO:0048514~blood vessel morphogenesis	53	1.86	1.86E-05	GNA13, RTN4, PGF, EDN1, WASF2, GJA1, ANPEP, PRRX2, CITED1, SHB, ARHGAP22, CUL7, CTGF, CXCR4, APOE, HMOX1, CCBE1, CASP8, IL1B, SEMA3C, SHC1, ANGPT2, PTPRJ, SGPL1, MMP19, MYH9, MMP14, JUNB, THY1, SMO, HIF1A, VEGFA, TNFRSF12A, FOXM1, ELK3, GPI1, ZFP36L1, PLCD3, ZC3H12A, COL18A1, KLF5, EPAS1, ITGA4, FZD5, EPHA2, ANXA2, NOTCH1, BAX, ECSCR, ITGA7, HBEGF, AMOT, RBPJ	2019	198	13588	1.80	7.16E-02	1.08E-03	3.45E-02
Annotation Cluster 19	Enrichment Score: 3.8181212850860247											
Category	Term	Count	%	PValue	Genes	List Total	Pop Hits	Pop Total	Fold Enrichment	Bonferroni	Benjamini	FDR
GOTERM_BP_FAT	GO:0002684~positive regulation of immune system process	71	2.49	4.08E-12	CADM1, STAT5B, HPS1, TLR2, PTPN22, TLR4, IL15, PNP, C1QC, TLR8, TGFB1, B2M, CFP, NOD2, MYD88, KLHL6, TICAM1, CFD, BCL10, ICOSL, HSP90AA1, LYN, C4B, RELA, NFAM1, CD40, THY1, C1QA, C1QB, CD83, PRKCQ, CD37, LAT2, CD80, LYST, IL12A, H2-AA, ITGAL, ADORA2B, IL4RA, UNC93B1, NFKBIA, IL7R, TNFRSF4, CD74, ADA, IRAK3, SLC11A1, TAP2, FCER1G, IL2RG, SH2B2, C2, INPP5D, THBS1, PTPRC, PTPN6, SELP, IL6, CARD9, IKZF1, H2-M3, FCGR1, FCGR3, P2RX7, CORO1A, CDKN1A, PLCG2, CLEC7A, H2-DMA, SASH3	2019	206	13588	2.32	1.63E-08	2.04E-09	7.57E-09
GOTERM_BP_FAT	GO:0050865~regulation of cell activation	52	1.82	1.46E-08	STAT5B, HPS1, TLR4, IL15, PNP, TGFB1, CASP3, MYD88, HMOX1, TICAM1, CSK, BCL10, ICOSL, HSP90AA1, TNFRSF14, NFAM1, CD40, GAL, THY1, PRKCQ, CD83, CD80, LYST, PRAM1, IL12A, H2-AA, ITGAL, LST1, NFKBID, ADORA2B, LRRC32, IL4RA, IL7R, TNFRSF4, ADA, CD74, ZC3H8, FCER1G, IL2RG, INPP5D, SELP, PTPN6, PTPRC, IL6, PLEK, IKZF1, CORO1A, CDKN1A, FCGR2B, SLC7A2, H2-DMA, SASH3	2019	156	13588	2.24	5.85E-05	3.08E-06	2.71E-05
GOTERM_BP_FAT	GO:0050867~positive regulation of cell activation	38	1.33	5.09E-08	ITGAL, ADORA2B, STAT5B, IL4RA, HPS1, TLR4, IL15, IL7R, TNFRSF4, PNP, CD74, TGFB1, ADA, MYD88, TICAM1, FCER1G, IL2RG, INPP5D, PTPRC, BCL10, SELP, ICOSL, IL6, HSP90AA1, IKZF1, PLEK, CD40, THY1, PRKCQ, CD83, CORO1A, CDKN1A, CD80, LYST, IL12A, H2-AA, H2-DMA, SASH3	2019	101	13588	2.53	2.04E-04	9.26E-06	9.46E-05
GOTERM_BP_FAT	GO:0002694~regulation of leukocyte activation	50	1.75	7.32E-08	STAT5B, HPS1, TLR4, IL15, PNP, TGFB1, CASP3, MYD88, HMOX1, TICAM1, CSK, BCL10, ICOSL, HSP90AA1, TNFRSF14, NFAM1, CD40, GAL, THY1, PRKCQ, CD83, CD80, LYST, PRAM1, IL12A, H2-AA, ITGAL, LST1, ADORA2B, NFKBID, LRRC32, IL4RA, IL7R, TNFRSF4, ADA, CD74, ZC3H8, FCER1G, IL2RG, INPP5D, PTPN6, PTPRC, IL6, IKZF1, CORO1A, CDKN1A, FCGR2B, SLC7A2, H2-DMA, SASH3	2019	154	13588	2.19	2.93E-04	1.17E-05	1.36E-04
GOTERM_BP_FAT	GO:0002696~positive regulation of leukocyte activation	36	1.26	3.18E-07	ITGAL, ADORA2B, STAT5B, IL4RA, HPS1, TLR4, IL15, IL7R, TNFRSF4, PNP, CD74, TGFB1, ADA, MYD88, TICAM1, FCER1G, IL2RG, INPP5D, PTPRC, BCL10, ICOSL, IL6, HSP90AA1, IKZF1, CD40, THY1, PRKCQ, CD83, CORO1A, CDKN1A, CD80, LYST, IL12A, H2-AA, H2-DMA, SASH3	2019	99	13588	2.45	1.27E-03	3.85E-05	5.90E-04
GOTERM_BP_FAT	GO:0051251~positive regulation of lymphocyte activation	34	1.19	8.23E-07	ITGAL, STAT5B, IL4RA, HPS1, TLR4, IL15, IL7R, TNFRSF4, PNP, CD74, TGFB1, ADA, MYD88, TICAM1, IL2RG, INPP5D, PTPRC, BCL10, ICOSL, IL6, HSP90AA1, IKZF1, CD40, THY1, PRKCQ, CD83, CDKN1A, CORO1A, CD80, LYST, IL12A, H2-AA, H2-DMA, SASH3	2019	94	13588	2.43	3.29E-03	7.16E-05	1.53E-03
GOTERM_BP_FAT	GO:0051249~regulation of lymphocyte activation	45	1.58	1.17E-06	ITGAL, LST1, NFKBID, LRRC32, STAT5B, IL4RA, HPS1, TLR4, IL15, IL7R, TNFRSF4, PNP, CD74, TGFB1, ADA, ZC3H8, CASP3, MYD88, TICAM1, IL2RG, INPP5D, CSK, PTPRC, BCL10, PTPN6, ICOSL, IL6, HSP90AA1, IKZF1, TNFRSF14, NFAM1, CD40, GAL, THY1, PRKCQ, CD83, CORO1A, CDKN1A, CD80, FCGR2B, LYST, IL12A, H2-AA, H2-DMA, SASH3	2019	144	13588	2.10	4.68E-03	9.76E-05	2.18E-03
Annotation Cluster 20	Enrichment Score: 3.7603734791236745											
Category	Term	Count	%	PValue	Genes	List Total	Pop Hits	Pop Total	Fold Enrichment	Bonferroni	Benjamini	FDR
GOTERM_MF_FAT	GO:0008047~enzyme activator activity	69	2.42	9.84E-08	GM2A, IQGAP2, ARHGAP15, TBC1D19, IQGAP1, TBC1D16, HMHA1, ARHGAP22, ARHGAP20, TIAM2, RINL, APOE, ARHGAP1, RANBP1, ARHGAP11A, ARHGAP9, BCL10, BCR, ARHGAP28, SIPA1L2, CYB5B, MMP14, THY1, ARHGAP25, ARHGAP30, TNFRSF10B, ACAP1, RIN1, RIN3, ARFGAP2, ARHGDIG, ARFGAP3, RAP1GAP, TBC1D9, SIPA1, GPSM3, APOC2, MYO9B, AZIN1, FAM13B, ADAP1, DOCK2, RGS12, GMIP, ALOX5AP, GPSM1, PYCARD, RASA3, ARHGDIA, ARHGDIB, FN1, TBC1D2, TBC1D2B, TBC1D10C, ABR, TBC1D10A, RGS18, RGS16, RACGAP1, RGS14, USP6NL, FBLN1, ADAP2, RGS2, CHML, GRTP1, APAF1, SH3BP1, ARAP1	1935	249	13288	1.90	1.39E-04	1.55E-05	1.62E-04
GOTERM_MF_FAT	GO:0030695~GTPase regulator activity	91	3.19	9.93E-08	PREX1, RASGEF1B, IQGAP2, ARHGAP15, TBC1D19, IQGAP1, RGL1, TBC1D16, ARHGAP22, HMHA1, DMXL2, ARHGAP20, TIAM2, RINL, TIAM1, ARHGAP1, RANBP1, ARHGAP11A, DOCK10, DOCK11, ARHGAP9, ARHGEF2, BCR, MADD, SIPA1L2, ARHGAP28, RAB3IL1, PSD4, ARHGEF9, WAS, THY1, ARHGAP25, ARHGAP30, MAP4K4, ACAP1, RIN1, RANGRF, SPATA13, RIN3, FGD2, ARFGAP2, ARHGDIG, ARFGAP3, RAP1GAP, TBC1D9, SIPA1, GPSM3, CYTH4, MAP4K1, MYO9B, CYTH3, FAM13B, ADAP1, PLEKHG3, DOCK2, PLEKHG2, RGS12, RASGRP3, GMIP, RASGRP4, GPSM1, IQSEC3, RASA3, FGD6, ARHGDIA, FGD3, ARHGDIB, TBC1D2, TBC1D2B, ABR, VAV3, TBC1D10C, TBC1D10A, EXPH5, RGS18, DOCK7, DOCK8, DOCK5, RACGAP1, RGS16, VAV1, RGS14, USP6NL, DOCK4, ADAP2, RGS2, CHML, GRTP1, SH3BP1, ARAP1, BCAR3	1935	361	13288	1.73	1.41E-04	1.41E-05	1.64E-04
GOTERM_MF_FAT	GO:0060589~nucleoside-triphosphatase regulator activity	91	3.19	2.21E-07	PREX1, RASGEF1B, IQGAP2, ARHGAP15, TBC1D19, IQGAP1, RGL1, TBC1D16, ARHGAP22, HMHA1, DMXL2, ARHGAP20, TIAM2, RINL, TIAM1, ARHGAP1, RANBP1, ARHGAP11A, DOCK10, DOCK11, ARHGAP9, ARHGEF2, BCR, MADD, SIPA1L2, ARHGAP28, RAB3IL1, PSD4, ARHGEF9, WAS, THY1, ARHGAP25, ARHGAP30, MAP4K4, ACAP1, RIN1, RANGRF, SPATA13, RIN3, FGD2, ARFGAP2, ARHGDIG, ARFGAP3, RAP1GAP, TBC1D9, SIPA1, GPSM3, CYTH4, MAP4K1, MYO9B, CYTH3, FAM13B, ADAP1, PLEKHG3, DOCK2, PLEKHG2, RGS12, RASGRP3, GMIP, RASGRP4, GPSM1, IQSEC3, RASA3, FGD6, ARHGDIA, FGD3, ARHGDIB, TBC1D2, TBC1D2B, ABR, VAV3, TBC1D10C, TBC1D10A, EXPH5, RGS18, DOCK7, DOCK8, DOCK5, RACGAP1, RGS16, VAV1, RGS14, USP6NL, DOCK4, ADAP2, RGS2, CHML, GRTP1, SH3BP1, ARAP1, BCAR3	1935	367	13288	1.70	3.12E-04	2.08E-05	3.64E-04

GOTERM_MF_FAT	GO:0005096~GTPase activator activity	56	1.96	3.05E-07	IQGAP2, ARHGAP15, TBC1D19, IQGAP1, TBC1D16, HMHA1, ARHGAP22, ARHGAP20, RINL, TIAM2, ARHGAP1, RANBP1, ARHGAP11A, ARHGAP9, BCR, ARHGAP28, SIPA1L2, THY1, ARHGAP25, ARHGAP30, ACAP1, RIN1, RIN3, ARFGAP2, ARHGDIG, ARFGAP3, RAP1GAP, TBC1D9, GPSM3, SIPA1, MYO9B, FAM13B, ADAP1, DOCK2, RGS12, GMIP, GPSM1, RASA3, ARHGDIA, ARHGDIB, TBC1D2, TBC1D2B, TBC1D10C, ABR, TBC1D10A, RGS18, RGS16, RACGAP1, RGS14, USP6NL, ADAP2, RGS2, CHML, GRTP1, SH3BP1, ARAP1	1935	192	13288	2.00	4.32E-04	2.70E-05	5.03E-04

Table S1.D GO Terms upregulated 48 hours post injury

Category	Term	Count	%	PValue	Genes	List Total	Pop Hits	Pop Total	Fold Enrichment	Bonferroni	Benjamini	FDR
Annotation Cluster 1	Enrichment Score: 9.395065310967698											
Category	Term	Count	%	PValue	Genes	List Total	Pop Hits	Pop Total	Fold Enrichment	Bonferroni	Benjamini	FDR
GOTERM_BP_FAT	GO:0030029~actin filament-based process	54	2.51	3.73E-11	TLN1, LIMA1, AIF1, S100A9, CAPZA1, RHOQ, ACTG1, RHOA, CAP1, RHOG, FMNL1, DBNL, ARHGEF2, ACTN4, ACTN1, TMSB10, MYH9, SIRPA, WAS, VASP, FLNA, ELMO1, FMN1, XIRP1, CFL1, TMSB4X, LCP1, MYO5A, MYL6, SHROOM3, TCAP, MYL2, ARPC4, MYO9B, CALR, PFN2, EZR, RAC2, SH2B2, CNN2, WIPF1, INF2, CSRP1, RACGAP1, GAS7, CAPN3, CORO1C, EPB4.1L2, NRAS, CORO1A, EPB4.1L1, EPS8, EPB4.1, TMOD1	1594	176	13588	2.62	1.36E-07	2.26E-08	6.87E-08
GOTERM_BP_FAT	GO:0030036~actin cytoskeleton organization	50	2.32	3.49E-10	TLN1, LIMA1, AIF1, S100A9, CAPZA1, RHOQ, ACTG1, RHOA, CAP1, RHOG, FMNL1, DBNL, ARHGEF2, ACTN4, ACTN1, TMSB10, MYH9, SIRPA, WAS, VASP, FLNA, ELMO1, FMN1, XIRP1, CFL1, TMSB4X, LCP1, SHROOM3, TCAP, MYL2, ARPC4, CALR, PFN2, EZR, RAC2, SH2B2, CNN2, INF2, CSRP1, RACGAP1, GAS7, CAPN3, EPB4.1L2, CORO1C, NRAS, CORO1A, EPB4.1L1, EPS8, EPB4.1, TMOD1	1594	165	13588	2.58	1.27E-06	1.06E-07	6.42E-07
GOTERM_BP_FAT	GO:0007010~cytoskeleton organization	76	3.53	5.01E-09	TLN1, LIMA1, AIF1, CAPZA1, S100A9, RHOQ, VILL, ACTG1, TUBB5, RHOA, RANBP1, CAP1, TUBA1B, RHOG, FMNL1, DBNL, ARHGEF2, KIF11, ACTN4, ACTN1, NUSAP1, TMSB10, ESPL1, PLK1S1, MYH9, TACC3, WAS, SIRPA, UBE2B, FLNA, VASP, THY1, ELMO1, FMN1, SGCG, XIRP1, TPPP, CFL1, TMSB4X, STMN1, GADD45A, LCP1, SHROOM3, MYL2, TCAP, ARPC4, CALR, SPC25, PFN2, DOCK2, EZR, RAC2, FBXO5, CNN2, SH2B2, TES, SYNPO, INF2, CEP192, BIRC5, CSRP1, RACGAP1, GAS7, CAPN3, BRCA1, CORO1C, EPB4.1L2, NRAS, CORO1A, YWHAH, EPB4.1L1, EPS8, LASP1, SVIL, EPB4.1, TMOD1	1594	326	13588	1.99	1.82E-05	1.40E-06	9.21E-06
Annotation Cluster 2	Enrichment Score: 8.96343293736693											
Category	Term	Count	%	PValue	Genes	List Total	Pop Hits	Pop Total	Fold Enrichment	Bonferroni	Benjamini	FDR
GOTERM_BP_FAT	GO:0019318~hexose metabolic process	52	2.42	8.01E-11	PRKAG3, PPARA, LDHB, GNPDA1, PHKB, PGD, PGAM1, PGAM2, OGDH, HIBADH, PPP1R1A, PDHA1, MAN2B1, MYC, ALDH5A1, PHKG1, FBP2, PPP1R3A, PGM2L1, PGM2, MAN2A2, MAN2A1, RENBP, PGM1, MDH1, PFKFB3, PFKFB1, SLC37A4, PHKA1, HK1, GALK2, PPP1R3B, ENO2, GYS1, GALE, AGL, ENO1, GPD2, PDK2, GPD1, WDTC1, GMDS, PDK3, EPM2A, BPGM, DLAT, MAN1C1, CPT1A, ATF3, PYGL, FABP5, PYGB	1594	169	13588	2.62	2.92E-07	3.24E-08	1.47E-07
GOTERM_BP_FAT	GO:0005996~monosaccharide metabolic process	55	2.56	3.39E-10	PRKAG3, PPARA, LDHB, GNPDA1, PHKB, PGD, PGAM1, PGAM2, OGDH, HIBADH, PPP1R1A, PDHA1, MAN2B1, MYC, ALDH5A1, PHKG1, FBP2, PPP1R3A, PGM2L1, PGM2, MAN2A2, MAN2A1, RENBP, PGM1, MDH1, PFKFB3, PFKFB1, SLC37A4, PHKA1, FN3K, HK1, NAGK, GALK2, PPP1R3B, ENO2, GYS1, GALE, AGL, ENO1, GPD2, PDK2, GPD1, WDTC1, GMDS, PDK3, EPM2A, BPGM, DLAT, MAN1C1, CPT1A, CSGALNACT1, ATF3, PYGL, FABP5, PYGB	1594	191	13588	2.45	1.23E-06	1.12E-07	6.24E-07
GOTERM_BP_FAT	GO:0006006~glucose metabolic process	41	1.91	4.74E-08	PRKAG3, LDHB, PPARA, PHKB, PHKA1, SLC37A4, PGD, PGAM1, PGAM2, HK1, OGDH, HIBADH, PPP1R3B, PPP1R1A, ENO2, GYS1, PDHA1, MYC, AGL, ENO1, GPD2, PDK2, GPD1, WDTC1, PHKG1, ALDH5A1, PDK3, EPM2A, BPGM, DLAT, FBP2, PPP1R3A, CPT1A, PGM2L1, PGM2, ATF3, PYGL, PGM1, FABP5, MDH1, PYGB	1594	140	13588	2.50	1.72E-04	7.84E-06	8.72E-05
Annotation Cluster 3	Enrichment Score: 8.576800896766448											
Category	Term	Count	%	PValue	Genes	List Total	Pop Hits	Pop Total	Fold Enrichment	Bonferroni	Benjamini	FDR
GOTERM_BP_FAT	GO:0009611~response to wounding	85	3.95	3.90E-11	GNA13, PPARA, LY86, F13A1, HPS1, TLR2, TLR4, TLR6, TLR7, C1QC, TGFB1, TLR8, CFP, GPX1, CASP3, MYD88, CD44, IL1B, CIITA, NFKBIZ, GATM, NCF1, LY96, MYH2, CD163, PLAUR, C1QA, C1QB, SERPINA3N, UNC13D, HIF1A, THBD, CCR5, CCR2, CTSB, PROS1, LCP1, PTAFR, CCL2, CCR1, CXCL3, CCL8, NINJ1, ITGB2, PF4, ABHD2, CCL4, TIMP3, CCL7, SLC11A1, TNFRSF1A, TNFRSF1B, MTPN, PYCARD, ENTPD1, THBS1, FN1, PTPN6, KLF6, LIPA, PLEK, CFB, EFEMP2, SPHK1, TLR13, EPHX2, CHI3L3, ANXA5, FCGR1, STAT3, CD180, FCGR3, SOD2, HDAC5, NOTCH2, STAB1, SLC7A2, CXCL13, BAX, ID3, ALOX5, CLEC7A, CD14, ORM2, IGFBP4	1594	347	13588	2.09	1.42E-07	2.03E-08	7.18E-08
GOTERM_BP_FAT	GO:0006952~defense response	94	4.37	1.86E-08	LY86, TLR2, H2-D1, BNIP3, TLR4, TLR6, TLR7, C1QC, TGFB1, IL10, TLR8, B2M, CFP, TMEM173, MYD88, CD44, IL1RAP, IL1B, MX1, CIITA, NFKBIZ, C5AR1, NGP, NCF1, LY96, CNPY3, CLIC1, ECSIT, CD163, C1QA, C1QB, SERPINA3N, UNC13D, HIF1A, CCR5, CCR2, H2-AA, PTAFR, IL1R1, CCL2, FGR, CCR1, CXCL3, CCL8, ITGB2, SP110, CCL4, CD74, CCL7, SLC11A1, TNFRSF1A, TNFRSF1B, TAP1, PYCARD, FCER1G, BCL3, THBS1, DHX58, FN1, PTPRC, PTPN6, LYZ2, LIPA, CARD9, H2-M3, CFB, IL1RL1, HCK, SPHK1, TLR13, EPHX2, MYO1F, SAMHD1, CHI3L3, COTL1, VDAC3, FCGR1, STAT3, CD180, FCGR3, VDAC1, HDAC5, ABCC9, FCGR2B, STAB1, SLC7A2, CXCL13, IRF8, APAF1, ALOX5, CLEC7A, CD14, ORM2, IGFBP4	1594	448	13588	1.79	6.76E-05	3.76E-06	3.42E-05
GOTERM_BP_FAT	GO:0006954~inflammatory response	57	2.65	2.57E-08	LY86, TLR2, TLR4, TLR6, C1QC, TLR7, TLR8, TGFB1, CFP, MYD88, CD44, IL1B, CIITA, NFKBIZ, LY96, NCF1, CD163, C1QA, C1QB, SERPINA3N, HIF1A, UNC13D, CCR5, CCR2, PTAFR, CCL2, CXCL3, CCR1, CCL8, ITGB2, CCL4, CCL7, TNFRSF1A, SLC11A1, TNFRSF1B, PYCARD, THBS1, FN1, LIPA, CFB, TLR13, SPHK1, EPHX2, CHI3L3, FCGR1, CD180, STAT3, FCGR3, HDAC5, STAB1, CXCL13, SLC7A2, CLEC7A, ALOX5, CD14, IGFBP4, ORM2	1594	225	13588	2.16	9.34E-05	4.67E-06	4.72E-05
Annotation Cluster 4	Enrichment Score: 6.230189097120934											
Category	Term	Count	%	PValue	Genes	List Total	Pop Hits	Pop Total	Fold Enrichment	Bonferroni	Benjamini	FDR
GOTERM_MF_FAT	GO:0000166~nucleotide binding	335	15.57	1.59E-09	KIFC1, ADCY7, TUBB2B, TUBB2A, PGD, LDHD, PRKX, HIBADH, BTK, ACTG1, GM5088, RAB28, OASL2, OASL1, MAP3K8, PIK3CG, SUCLG1, PTBP1, MYH2, MYLK2, WNK2, GEM, MYH9, MYH8, RFC5, RND3, MAPK6, NME1, DLD, PDGFRA, RAB12, MAPK7, MDH1, 5430435G22RIK, ACADSB, GNAI3, GNAI2, NEK2, PFKFB3, PFKFB1, STK17B, MYEF2, CHEK1, IGF2BP2, ARF6, MYO9B, OAS2, NAGK, HADHA, HSPA1L, MUSK, RAC2, IDH2, PABPC1, DHX58, GCDH, GPD1, KIF3B, SPHK1, ABCG3, ATP13A3, VDAC3, ABCG1, VDAC1, ATP13A4, HYOU1, ATP2A2, ARF3, ARF4, TNK2, HSPA13, ACAD11, CLCN5, SLC27A1, SPG7, BCKDK, RAB5C, KCNJ11, CAMKK2, CKB, ACTR3, MCCC2, ACTR2, MCCC1, TUBB5, TUBB6, MASTL, DOCK10, SIK1, TOP2A, DOCK11, TARSL2, NT5C, CDC7, RAP2B, CDC6, RAP2A, LYN, PHKG1, RAB4B, NDUFA13, PKDCC, 1700017B05RIK, GRHPR, NDUFA10, MAST3, DNAJC27, GM12250, SQLE, ADK, RIPK1, EIF4A1, MAP3K10, RBM38, STEAP2, ARL4C, MYO5A, ALDH18A1, UGDH, POLA1, HK1, GALK2, RAB44, RAB43, PTK2B, ABCD2, BUB1, CERK, PRKAA2, KIF21B, ETFA, ACSL5, MYO5C, PDK2, NOS1, PDK3, ACLY, NADK, NPR2, ATP1A1, APRT, SDHA, RPS6KA5, RAB32, NRAS, ABCC9, RAB31, MAPK12, ADCY9, PYGL, RAB34, HSPA4L, KIF20B, PHGDH, ABCC3, CHTF18, GYK, AACS, GNA13, RNASEL, D5ERTD579E, HMGCR, RBM3, F13A1, RRAD, PI4K2B, MTHFD1L, PRKAR2B, ACSS1, IDH3G, OPLAH, GBP10, RALB, MLKL, SAR1B, MX1, INSR, RDM1, MATK, RBMS1, CIITA, POR, MAP4K4, RIPK3, OAS1A, PCCB, PCCA, SRXN1, ME1, ME3, ME2, FGR, NT5C1A, STK10, BMPR2, MAP4K1, TIMP4, RRAGD, CMPK1, IRAK4, P2RY6, IVD, HNRNPF, DYRK2, UBE2D1, ENTPD1, RUNX1, SLC30A9, HELLS, CSF1R, ERCC6L, TRP53, EEF1A1, OBSCN, ADSSL1, RAB8A, RAB8B, MYO1C, EEF1A2, AK1, MYO1G, IDH3B, BRIP1, ATAD2, MYO1F, DOCK8, PSMB8, MMAB, IDH3A, ABCB4, IKBKE, P2RX4, DNA2, PLK4, P2RX6, PLK3, PLK2, ULK1, ULK2, NDUFV1, NDUFV2, RHOT2, MYH13, RAP1B, MERTK, PIP4K2A, KIF23, KIF22, ACOX1, D2HGDH, ATL2, FIGNL1, SLFN9, ATL3, PEX6, PPIP5K2, PPIP5K1, PINK1, SLFN8, RHOQ, FES, RHOV, SLFN4, SLFN2, ACVR1B, GSR, RHOA, RHOC, CSK, TUBA1A, SUCLA2, NDUFS2, TUBA1B, TUBA1C, RHOG, CDK14, ATP8B4, RHOH, PRKCA, ACADM, KIF11, HSP90AA1, LIMK1, ACADS, MCM3, UBE2C, CDK4, PRKCD, MCM4, UBE2B, CDK2, MCM5, ARL3, PRKCB, MCM6, ACADVL, MFN2, KIF1C, MFN1, ATP6V1A, ACVR2B, RRAS2, H2-AA, BUB1B, CAMK1D, CAMK2G, TRA2A, DCK, TRIB3, ASNS, NAIP2, KIF4, TAP1, UCK2, HSPA5, EHD1, CAMK2A, DCLK1, EHD4, ACTB, ALPK1, HCK, ABCA8B, ABCA8A, GRSF1, SIRT7, SMC2, SMC4, HSP90B1, CYBB, TEP1, JAK2, APAF1, H2-DMA, ATP8A1	1524	2183	13288	1.34	2.04E-06	4.07E-07	2.59E-06
GOTERM_MF_FAT	GO:0017076~purine nucleotide binding	294	13.67	1.93E-09	KIFC1, ADCY7, TUBB2B, TUBB2A, LDHD, PRKX, BTK, ACTG1, RAB28, OASL2, OASL1, MAP3K8, PIK3CG, SUCLG1, MYH2, MYLK2, WNK2, GEM, MYH9, MYH8, RFC5, RND3, MAPK6, NME1, DLD, PDGFRA, RAB12, MAPK7, 5430435G22RIK, ACADSB, GNAI3, GNAI2, NEK2, PFKFB3, PFKFB1, STK17B, CHEK1, ARF6, MYO9B, OAS2, NAGK, HSPA1L, MUSK, RAC2, DHX58, GCDH, KIF3B, SPHK1, ABCG3, ATP13A3, ABCG1, ATP13A4, HYOU1, ATP2A2, ARF3, ARF4, TNK2, HSPA13, ACAD11, CLCN5, SPG7, BCKDK, RAB5C, KCNJ11, CAMKK2, CKB, ACTR3, ACTR2, MCCC2, MCCC1, TUBB5, TUBB6, MASTL, DOCK10, SIK1, TOP2A, DOCK11, TARSL2, RAP2B, CDC7, CDC6, RAP2A, LYN, PHKG1, RAB4B, NDUFA13, PKDCC, 1700017B05RIK, NDUFA10, MAST3, DNAJC27, GM12250, SQLE, ADK, RIPK1, EIF4A1, MAP3K10, STEAP2, ARL4C, MYO5A, ALDH18A1, HK1, GALK2, RAB44, RAB43, PTK2B, ABCD2, BUB1, CERK, PRKAA2, KIF21B, ETFA, ACSL5, MYO5C, PDK2, NOS1, PDK3, ACLY, NADK, NPR2, ATP1A1, APRT, SDHA, RPS6KA5, RAB32, NRAS, ABCC9, RAB31, ADCY9, MAPK12, RAB34, HSPA4L, KIF20B, ABCC3, CHTF18, GYK, AACS, GNA13, RNASEL, D5ERTD579E, F13A1, RRAD, PI4K2B, MTHFD1L, PRKAR2B, ACSS1, IDH3G, OPLAH, GBP10, RALB, MLKL, SAR1B, MX1, INSR, MATK, CIITA, MAP4K4, RIPK3, OAS1A, PCCB, PCCA, SRXN1, FGR, STK10, BMPR2, MAP4K1, TIMP4, RRAGD, CMPK1, IRAK4, IVD, DYRK2, UBE2D1, ENTPD1, RUNX1, HELLS, CSF1R, ERCC6L, TRP53, EEF1A1, OBSCN, ADSSL1, RAB8A, RAB8B, MYO1C, AK1, EEF1A2, MYO1G, ATAD2, BRIP1, MYO1F, DOCK8, PSMB8, MMAB, ABCB4, IKBKE, P2RX4, DNA2, PLK4, P2RX6, PLK3, PLK2, ULK1, ULK2, RHOT2, MYH13, RAP1B, MERTK, PIP4K2A, KIF23, KIF22, ACOX1, D2HGDH, ATL2, FIGNL1, SLFN9, ATL3, PEX6, PPIP5K2, PPIP5K1, PINK1, RHOQ, SLFN8, FES, RHOV, SLFN4, SLFN2, ACVR1B, GSR, RHOA, RHOC, CSK, SUCLA2, TUBA1A, TUBA1B, TUBA1C, RHOG, CDK14, ATP8B4, RHOH, PRKCA, ACADM, HSP90AA1, KIF11, LIMK1, ACADS, MCM3, UBE2C, CDK4, PRKCD, MCM4, UBE2B, CDK2, MCM5, PRKCB, MCM6, ARL3, ACADVL, MFN2, KIF1C, MFN1, ATP6V1A, ACVR2B, RRAS2, BUB1B, H2-AA, CAMK1D, CAMK2G, DCK, TRIB3, ASNS, NAIP2, KIF4, TAP1, UCK2, HSPA5, EHD1, CAMK2A, DCLK1, EHD4, ACTB, ALPK1, HCK, ABCA8B, ABCA8A, SMC2, SMC4, HSP90B1, CYBB, TEP1, JAK2, APAF1, H2-DMA, ATP8A1	1524	1871	13288	1.37	2.47E-06	4.12E-07	3.14E-06
GOTERM_MF_FAT	GO:0032553~ribonucleotide binding	276	12.83	6.49E-08	KIFC1, ADCY7, TUBB2B, TUBB2A, PRKX, BTK, ACTG1, RAB28, OASL2, OASL1, MAP3K8, PIK3CG, SUCLG1, MYH2, MYLK2, WNK2, GEM, MYH9, MYH8, RFC5, RND3, MAPK6, NME1, PDGFRA, RAB12, MAPK7, 5430435G22RIK, GNAI3, GNAI2, PFKFB3, NEK2, PFKFB1, STK17B, CHEK1, MYO9B, ARF6, OAS2, NAGK, HSPA1L, MUSK, RAC2, DHX58, KIF3B, SPHK1, ABCG3, ATP13A3, ABCG1, ATP13A4, HYOU1, ATP2A2, ARF3, ARF4, TNK2, HSPA13, CLCN5, SPG7, BCKDK, RAB5C, KCNJ11, CAMKK2, CKB, ACTR3, ACTR2, MCCC2, MCCC1, TUBB5, TUBB6, MASTL, DOCK10, SIK1, TOP2A, DOCK11, TARSL2, RAP2B, CDC7, RAP2A, CDC6, LYN, PHKG1, RAB4B, NDUFA13, PKDCC, 1700017B05RIK, NDUFA10, MAST3, DNAJC27, GM12250, ADK, RIPK1, EIF4A1, MAP3K10, ARL4C, MYO5A, ALDH18A1, HK1, GALK2, RAB44, RAB43, PTK2B, ABCD2, BUB1, CERK, PRKAA2, KIF21B, ACSL5, MYO5C, PDK2, PDK3, ACLY, NADK, ATP1A1, NPR2, APRT, RPS6KA5, RAB32, NRAS, ABCC9, RAB31, ADCY9, MAPK12, RAB34, HSPA4L, KIF20B, ABCC3, CHTF18, GYK, AACS, GNA13, RNASEL, D5ERTD579E, F13A1, RRAD, PI4K2B, MTHFD1L, PRKAR2B, ACSS1, IDH3G, OPLAH, GBP10, RALB, MLKL, SAR1B, MX1, INSR, MATK, CIITA, MAP4K4, RIPK3, OAS1A, PCCB, PCCA, SRXN1, FGR, STK10, BMPR2, MAP4K1, TIMP4, RRAGD, CMPK1, IRAK4, DYRK2, UBE2D1, ENTPD1, RUNX1, HELLS, CSF1R, ERCC6L, TRP53, EEF1A1, OBSCN, RAB8A, ADSSL1, RAB8B, MYO1C, AK1, EEF1A2, MYO1G, ATAD2, BRIP1, MYO1F, DOCK8, PSMB8, MMAB, ABCB4, IKBKE, P2RX4, DNA2, PLK4, P2RX6, PLK3, PLK2, ULK1, ULK2, RHOT2, MYH13, RAP1B, MERTK, PIP4K2A, KIF23, KIF22, ATL2, FIGNL1, SLFN9, ATL3, PEX6, PPIP5K2, PPIP5K1, PINK1, RHOQ, SLFN8, FES, RHOV, SLFN4, SLFN2, ACVR1B, RHOA, RHOC, CSK, SUCLA2, TUBA1A, TUBA1B, TUBA1C, RHOG, CDK14, ATP8B4, RHOH, PRKCA, HSP90AA1, KIF11, LIMK1, MCM3, UBE2C, CDK4, PRKCD, UBE2B, MCM4, CDK2, MCM5, PRKCB, MCM6, ARL3, MFN2, KIF1C, MFN1, ATP6V1A, ACVR2B, RRAS2, BUB1B, H2-AA, CAMK1D, CAMK2G, DCK, TRIB3, ASNS, NAIP2, KIF4, TAP1, UCK2, HSPA5, EHD1, CAMK2A, DCLK1, EHD4, ACTB, ALPK1, HCK, ABCA8B, ABCA8A, SMC2, SMC4, HSP90B1, TEP1, JAK2, APAF1, H2-DMA, ATP8A1	1524	1796	13288	1.34	8.33E-05	9.26E-06	1.06E-04

Category	Term	Count	%	PValue	Genes	List Total	Pop Hits	Pop Total	Fold Enrichment	Bonferroni	Benjamini	FDR
GOTERM_MF_FAT	GO:0032555~purine ribonucleotide binding	276	12.83	6.49E-08	KIFC1, ADCY7, TUBB2B, TUBB2A, PRKX, BTK, ACTG1, RAB28, OASL2, OASL1, MAP3K8, PIK3CG, SUCLG1, MYH2, MYLK2, WNK2, GEM, MYH9, MYH8, RFC5, RND3, MAPK6, NME1, PDGFRA, RAB12, MAPK7, 5430435G22RIK, GNAI3, GNAI2, PFKFB3, NEK2, PFKFB1, STK17B, CHEK1, MYO9B, ARF6, OAS2, NAGK, HSPA1L, MUSK, RAC2, DHX58, KIF3B, SPHK1, ABCG3, ATP13A3, ABCG1, ATP13A4, HYOU1, ATP2A2, ARF3, ARF4, TNK2, HSPA13, CLCN5, SPG7, BCKDK, RAB5C, KCNJ11, CAMKK2, CKB, ACTR3, ACTR2, MCCC2, MCCC1, TUBB5, TUBB6, MASTL, DOCK10, SIK1, TOP2A, DOCK11, TARSL2, RAP2B, CDC7, RAP2A, CDC6, LYN, PHKG1, RAB4B, NDUFA13, PKDCC, 1700017B05RIK, NDUFA10, MAST3, DNAJC27, GM12250, ADK, RIPK1, EIF4A1, MAP3K10, ARL4C, MYO5A, ALDH18A1, HK1, GALK2, RAB44, RAB43, PTK2B, ABCD2, BUB1, CERK, PRKAA2, KIF21B, ACSL5, MYO5C, PDK2, PDK3, ACLY, NADK, ATP1A1, NPR2, APRT, RPS6KA5, RAB32, NRAS, ABCC9, RAB31, ADCY9, MAPK12, RAB34, HSPA4L, KIF20B, ABCC3, CHTF18, GYK, AACS, GNA13, RNASEL, D5ERTD579E, F13A1, RRAD, PI4K2B, MTHFD1L, PRKAR2B, ACSS1, IDH3G, OPLAH, GBP10, RALB, MLKL, SAR1B, MX1, INSR, MATK, CIITA, MAP4K4, RIPK3, OAS1A, PCCB, PCCA, SRXN1, FGR, STK10, BMPR2, MAP4K1, TIMP4, RRAGD, CMPK1, IRAK4, DYRK2, UBE2D1, ENTPD1, RUNX1, HELLS, CSF1R, ERCC6L, TRP53, EEF1A1, OBSCN, RAB8A, ADSSL1, RAB8B, MYO1C, AK1, EEF1A2, MYO1G, ATAD2, BRIP1, MYO1F, DOCK8, PSMB8, MMAB, ABCB4, IKBKE, P2RX4, DNA2, PLK4, P2RX6, PLK3, PLK2, ULK1, ULK2, RHOT2, MYH13, RAP1B, MERTK, PIP4K2A, KIF23, KIF22, ATL2, FIGNL1, SLFN9, ATL3, PEX6, PPIP5K2, PPIP5K1, PINK1, RHOQ, SLFN8, FES, RHOV, SLFN4, SLFN2, ACVR1B, RHOA, RHOC, CSK, SUCLA2, TUBA1A, TUBA1B, TUBA1C, RHOG, CDK14, ATP8B4, RHOH, PRKCA, HSP90AA1, KIF11, LIMK1, MCM3, UBE2C, CDK4, PRKCD, UBE2B, MCM4, CDK2, MCM5, PRKCB, MCM6, ARL3, MFN2, KIF1C, MFN1, ATP6V1A, ACVR2B, RRAS2, BUB1B, H2-AA, CAMK1D, CAMK2G, DCK, TRIB3, ASNS, NAIP2, KIF4, TAP1, UCK2, HSPA5, EHD1, CAMK2A, DCLK1, EHD4, ACTB, ALPK1, HCK, ABCA8B, ABCA8A, SMC2, SMC4, HSP90B1, TEP1, JAK2, APAF1, H2-DMA, ATP8A1	1524	1796	13288	1.34	8.33E-05	9.26E-06	1.06E-04
GOTERM_MF_FAT	GO:0030554~adenyl nucleotide binding	232	10.79	3.48E-06	KIFC1, ADCY7, LDHD, PRKX, BTK, ACTG1, OASL2, OASL1, MAP3K8, PIK3CG, MYH2, MYLK2, WNK2, MYH9, MYH8, RFC5, MAPK6, NME1, DLD, PDGFRA, MAPK7, ACADSB, PFKFB3, NEK2, PFKFB1, STK17B, CHEK1, MYO9B, OAS2, NAGK, HSPA1L, MUSK, DHX58, GCDH, KIF3B, SPHK1, ABCG3, ATP13A3, ABCG1, ATP13A4, HYOU1, ATP2A2, HSPA13, TNK2, ACAD11, CLCN5, SPG7, BCKDK, KCNJ11, CAMKK2, CKB, ACTR3, ACTR2, MCCC2, MCCC1, MASTL, SIK1, TOP2A, TARSL2, CDC7, CDC6, LYN, PHKG1, NDUFA13, PKDCC, NDUFA10, MAST3, SQLE, RIPK1, ADK, EIF4A1, MAP3K10, STEAP2, MYO5A, ALDH18A1, HK1, GALK2, PTK2B, ABCD2, BUB1, CERK, PRKAA2, KIF21B, MYO5C, ACSL5, ETFA, PDK2, NOS1, PDK3, ACLY, ATP1A1, NPR2, NADK, APRT, SDHA, RPS6KA5, ABCC9, ADCY9, MAPK12, HSPA4L, ABCC3, KIF20B, CHTF18, GYK, AACS, RNASEL, D5ERTD579E, F13A1, PI4K2B, MTHFD1L, PRKAR2B, ACSS1, IDH3G, OPLAH, MLKL, INSR, MATK, CIITA, MAP4K4, RIPK3, OAS1A, PCCB, PCCA, SRXN1, FGR, STK10, BMPR2, MAP4K1, TIMP4, CMPK1, IRAK4, IVD, DYRK2, ENTPD1, RUNX1, UBE2D1, HELLS, CSF1R, ERCC6L, TRP53, OBSCN, MYO1C, AK1, MYO1G, MYO1F, ATAD2, BRIP1, MMAB, PSMB8, ABCB4, P2RX4, DNA2, IKBKE, PLK4, PLK3, P2RX6, PLK2, ULK1, ULK2, MYH13, MERTK, PIP4K2A, KIF23, KIF22, ACOX1, D2HGDH, FIGNL1, SLFN9, PEX6, PPIP5K2, PPIP5K1, PINK1, SLFN8, FES, SLFN4, SLFN2, ACVR1B, GSR, SUCLA2, CSK, CDK14, ATP8B4, PRKCA, HSP90AA1, KIF11, ACADM, ACADS, LIMK1, MCM3, CDK4, UBE2C, UBE2B, MCM4, PRKCD, CDK2, MCM5, PRKCB, MCM6, ACADVL, KIF1C, ATP6V1A, ACVR2B, BUB1B, H2-AA, CAMK1D, CAMK2G, DCK, TRIB3, ASNS, NAIP2, KIF4, TAP1, UCK2, HSPA5, EHD1, CAMK2A, DCLK1, EHD4, ACTB, ALPK1, ABCA8B, HCK, ABCA8A, SMC2, SMC4, CYBB, HSP90B1, TEP1, JAK2, APAF1, H2-DMA, ATP8A1	1524	1535	13288	1.32	4.46E-03	3.19E-04	5.67E-03
GOTERM_MF_FAT	GO:0001883~purine nucleoside binding	232	10.79	6.47E-06	KIFC1, ADCY7, LDHD, PRKX, BTK, ACTG1, OASL2, OASL1, MAP3K8, PIK3CG, MYH2, MYLK2, WNK2, MYH9, MYH8, RFC5, MAPK6, NME1, DLD, PDGFRA, MAPK7, ACADSB, PFKFB3, NEK2, PFKFB1, STK17B, CHEK1, MYO9B, OAS2, NAGK, HSPA1L, MUSK, DHX58, GCDH, KIF3B, SPHK1, ABCG3, ATP13A3, ABCG1, ATP13A4, HYOU1, ATP2A2, HSPA13, TNK2, ACAD11, CLCN5, SPG7, BCKDK, KCNJ11, CAMKK2, CKB, ACTR3, ACTR2, MCCC2, MCCC1, MASTL, SIK1, TOP2A, TARSL2, CDC7, CDC6, LYN, PHKG1, NDUFA13, PKDCC, NDUFA10, MAST3, SQLE, RIPK1, ADK, EIF4A1, MAP3K10, STEAP2, MYO5A, ALDH18A1, HK1, GALK2, PTK2B, ABCD2, BUB1, CERK, PRKAA2, KIF21B, MYO5C, ACSL5, ETFA, PDK2, NOS1, PDK3, ACLY, ATP1A1, NPR2, NADK, APRT, SDHA, RPS6KA5, ABCC9, ADCY9, MAPK12, HSPA4L, ABCC3, KIF20B, CHTF18, GYK, AACS, RNASEL, D5ERTD579E, F13A1, PI4K2B, MTHFD1L, PRKAR2B, ACSS1, IDH3G, OPLAH, MLKL, INSR, MATK, CIITA, MAP4K4, RIPK3, OAS1A, PCCB, PCCA, SRXN1, FGR, STK10, BMPR2, MAP4K1, TIMP4, CMPK1, IRAK4, IVD, DYRK2, ENTPD1, RUNX1, UBE2D1, HELLS, CSF1R, ERCC6L, TRP53, OBSCN, MYO1C, AK1, MYO1G, MYO1F, ATAD2, BRIP1, MMAB, PSMB8, ABCB4, P2RX4, DNA2, IKBKE, PLK4, PLK3, P2RX6, PLK2, ULK1, ULK2, MYH13, MERTK, PIP4K2A, KIF23, KIF22, ACOX1, D2HGDH, FIGNL1, SLFN9, PEX6, PPIP5K2, PPIP5K1, PINK1, SLFN8, FES, SLFN4, SLFN2, ACVR1B, GSR, SUCLA2, CSK, CDK14, ATP8B4, PRKCA, HSP90AA1, KIF11, ACADM, ACADS, LIMK1, MCM3, CDK4, UBE2C, UBE2B, MCM4, PRKCD, CDK2, MCM5, PRKCB, MCM6, ACADVL, KIF1C, ATP6V1A, ACVR2B, BUB1B, H2-AA, CAMK1D, CAMK2G, DCK, TRIB3, ASNS, NAIP2, KIF4, TAP1, UCK2, HSPA5, EHD1, CAMK2A, DCLK1, EHD4, ACTB, ALPK1, ABCA8B, HCK, ABCA8A, SMC2, SMC4, CYBB, HSP90B1, TEP1, JAK2, APAF1, H2-DMA, ATP8A1	1524	1548	13288	1.31	8.27E-03	5.19E-04	1.05E-02
GOTERM_MF_FAT	GO:0001882~nucleoside binding	233	10.83	7.09E-06	KIFC1, ADCY7, LDHD, PRKX, BTK, ACTG1, OASL2, OASL1, MAP3K8, PIK3CG, MYH2, MYLK2, WNK2, MYH9, MYH8, RFC5, MAPK6, NME1, DLD, PDGFRA, MAPK7, ACADSB, PFKFB3, NEK2, PFKFB1, STK17B, CHEK1, MYO9B, OAS2, NAGK, HSPA1L, MUSK, DHX58, GCDH, KIF3B, SPHK1, ABCG3, ATP13A3, ABCG1, ATP13A4, HYOU1, ATP2A2, HSPA13, TNK2, ACAD11, CLCN5, SPG7, BCKDK, KCNJ11, CAMKK2, CKB, ACTR3, ACTR2, MCCC2, MCCC1, MASTL, SIK1, TOP2A, TARSL2, CDC7, CDC6, LYN, PHKG1, NDUFA13, PKDCC, NDUFA10, MAST3, SQLE, RIPK1, ADK, EIF4A1, MAP3K10, STEAP2, MYO5A, ALDH18A1, POLA1, HK1, GALK2, PTK2B, ABCD2, BUB1, CERK, PRKAA2, KIF21B, MYO5C, ACSL5, ETFA, PDK2, NOS1, PDK3, ACLY, ATP1A1, NPR2, NADK, APRT, SDHA, RPS6KA5, ABCC9, ADCY9, MAPK12, HSPA4L, ABCC3, KIF20B, CHTF18, GYK, AACS, RNASEL, D5ERTD579E, F13A1, PI4K2B, MTHFD1L, PRKAR2B, ACSS1, IDH3G, OPLAH, MLKL, INSR, MATK, CIITA, MAP4K4, RIPK3, OAS1A, PCCB, PCCA, SRXN1, FGR, STK10, BMPR2, MAP4K1, TIMP4, CMPK1, IRAK4, IVD, DYRK2, ENTPD1, RUNX1, UBE2D1, HELLS, CSF1R, ERCC6L, TRP53, OBSCN, MYO1C, AK1, MYO1G, MYO1F, ATAD2, BRIP1, MMAB, PSMB8, ABCB4, P2RX4, DNA2, IKBKE, PLK4, PLK3, P2RX6, PLK2, ULK1, ULK2, MYH13, MERTK, PIP4K2A, KIF23, KIF22, ACOX1, D2HGDH, FIGNL1, SLFN9, PEX6, PPIP5K2, PPIP5K1, PINK1, SLFN8, FES, SLFN4, SLFN2, ACVR1B, GSR, SUCLA2, CSK, CDK14, ATP8B4, PRKCA, HSP90AA1, KIF11, ACADM, ACADS, LIMK1, MCM3, CDK4, UBE2C, UBE2B, MCM4, PRKCD, CDK2, MCM5, PRKCB, MCM6, ACADVL, KIF1C, ATP6V1A, ACVR2B, BUB1B, H2-AA, CAMK1D, CAMK2G, DCK, TRIB3, ASNS, NAIP2, KIF4, TAP1, UCK2, HSPA5, EHD1, CAMK2A, DCLK1, EHD4, ACTB, ALPK1, ABCA8B, HCK, ABCA8A, SMC2, SMC4, CYBB, HSP90B1, TEP1, JAK2, APAF1, H2-DMA, ATP8A1	1524	1558	13288	1.30	9.06E-03	5.35E-04	1.16E-02
Annotation Cluster 5	Enrichment Score: 5.855933634213578											
Category	Term	Count	%	PValue	Genes	List Total	Pop Hits	Pop Total	Fold Enrichment	Bonferroni	Benjamini	FDR
GOTERM_BP_FAT	GO:0048002~antigen processing and presentation of peptide antigen	19	0.88	1.01E-08	H2-M3, H2-D1, UNC93B1, 2610524H06RIK, IFI30, H2-DMB1, H2-AB1, CALR, FCGR1, CD74, FCGR3, B2M, SLC11A1, FCGR2B, H2-EB1, FCER1G, H2-AA, MR1, H2-DMA	1594	35	13588	4.63	3.68E-05	2.45E-06	1.86E-05
GOTERM_BP_FAT	GO:0002478~antigen processing and presentation of exogenous peptide antigen	15	0.70	2.56E-08	H2-M3, UNC93B1, 2610524H06RIK, IFI30, H2-DMB1, H2-AB1, FCGR1, CD74, FCGR3, B2M, FCGR2B, H2-EB1, FCER1G, H2-AA, H2-DMA	1594	23	13588	5.56	9.33E-05	4.91E-06	4.72E-05
GOTERM_BP_FAT	GO:0019884~antigen processing and presentation of exogenous antigen	15	0.70	7.11E-07	H2-M3, UNC93B1, 2610524H06RIK, IFI30, H2-DMB1, H2-AB1, FCGR1, CD74, FCGR3, B2M, FCGR2B, H2-EB1, FCER1G, H2-AA, H2-DMA	1594	28	13588	4.57	2.58E-03	8.35E-05	1.31E-03
GOTERM_BP_FAT	GO:0002495~antigen processing and presentation of peptide antigen via MHC class II	11	0.51	1.96E-06	FCGR2B, H2-EB1, UNC93B1, 2610524H06RIK, H2-AA, FCER1G, H2-DMB1, IFI30, H2-AB1, H2-DMA, CD74	1594	16	13588	5.86	7.10E-03	1.93E-04	3.60E-03
GOTERM_BP_FAT	GO:0019886~antigen processing and presentation of exogenous peptide antigen via MHC class II	11	0.51	1.96E-06	FCGR2B, H2-EB1, UNC93B1, 2610524H06RIK, H2-AA, FCER1G, H2-DMB1, IFI30, H2-AB1, H2-DMA, CD74	1594	16	13588	5.86	7.10E-03	1.93E-04	3.60E-03
GOTERM_BP_FAT	GO:0002504~antigen processing and presentation of peptide or polysaccharide antigen via MHC class I	11	0.51	1.62E-05	FCGR2B, H2-EB1, UNC93B1, 2610524H06RIK, H2-AA, FCER1G, H2-DMB1, IFI30, H2-AB1, H2-DMA, CD74	1594	19	13588	4.94	5.72E-02	1.02E-03	2.98E-02
Annotation Cluster 6	Enrichment Score: 5.827203670584949											
Category	Term	Count	%	PValue	Genes	List Total	Pop Hits	Pop Total	Fold Enrichment	Bonferroni	Benjamini	FDR
GOTERM_MF_FAT	GO:0016651~oxidoreductase activity, acting on NADH or NADPH	23	1.07	9.98E-09	CYB5R3, NDUFB3, NDUFA5, GMPR2, NCF2, NDUFB8, NCF1, NDUFA13, GMPR, ECSIT, NDUFAF1, POR, NDUFA12, NDUFS7, GSR, NDUFS4, NDUFV1, NDUFS8, NDUFV2, DLD, NDUFS3, NDUFS2, NDUFS1	1524	51	13288	3.93	1.28E-05	1.83E-06	1.63E-05
GOTERM_MF_FAT	GO:0003954~NADH dehydrogenase activity	13	0.60	3.55E-06	NDUFB3, NDUFB8, NDUFA13, NDUFAF1, NDUFA12, NDUFS7, NDUFS4, NDUFV1, NDUFV2, NDUFS8, NDUFS3, NDUFS2, NDUFS1	1524	24	13288	4.72	4.55E-03	3.04E-04	5.78E-03
GOTERM_MF_FAT	GO:0050136~NADH dehydrogenase (quinone) activity	13	0.60	3.55E-06	NDUFB3, NDUFB8, NDUFA13, NDUFAF1, NDUFA12, NDUFS7, NDUFS4, NDUFV1, NDUFV2, NDUFS8, NDUFS3, NDUFS2, NDUFS1	1524	24	13288	4.72	4.55E-03	3.04E-04	5.78E-03
GOTERM_MF_FAT	GO:0008137~NADH dehydrogenase (ubiquinone) activity	13	0.60	3.55E-06	NDUFB3, NDUFB8, NDUFA13, NDUFAF1, NDUFA12, NDUFS7, NDUFS4, NDUFV1, NDUFV2, NDUFS8, NDUFS3, NDUFS2, NDUFS1	1524	24	13288	4.72	4.55E-03	3.04E-04	5.78E-03
GOTERM_MF_FAT	GO:0016655~oxidoreductase activity, acting on NADH or NADPH, quinone or similar compound as acce	13	0.60	1.64E-05	NDUFB3, NDUFB8, NDUFA13, NDUFAF1, NDUFA12, NDUFS7, NDUFS4, NDUFV1, NDUFV2, NDUFS8, NDUFS3, NDUFS2, NDUFS1	1524	27	13288	4.20	2.08E-02	9.15E-04	2.67E-02
Annotation Cluster 7	Enrichment Score: 5.643439383324884											
Category	Term	Count	%	PValue	Genes	List Total	Pop Hits	Pop Total	Fold Enrichment	Bonferroni	Benjamini	FDR
GOTERM_BP_FAT	GO:0045333~cellular respiration	25	1.16	1.38E-08	UQCRC1, NDUFB8, IDH3G, UQCR11, IDH2, FH1, SUCLA2, NDUFS1, NDUFA5, ALDH5A1, SUCLG1, CS, IDH3B, IDH3A, SOD2, SLC25A12, SDHA, SDHB, SLC25A13, SDHC, DLD, SLC37A2, 2610507B11RIK, UQCRB, MDH1	1594	59	13588	3.61	5.04E-05	2.96E-06	2.55E-05
GOTERM_BP_FAT	GO:0051187~cofactor catabolic process	17	0.79	1.13E-07	ALDH1L1, NCF2, NCF1, SUCLG1, CS, IDH3B, IDH3A, SDHA, SDHB, IDH3G, SDHC, HMOX1, IDH2, 2610507B11RIK, FH1, SUCLA2, MDH1	1594	32	13588	4.53	4.11E-04	1.71E-05	2.08E-04
GOTERM_BP_FAT	GO:0009109~coenzyme catabolic process	16	0.74	1.59E-07	ALDH1L1, NCF2, NCF1, SUCLG1, CS, IDH3B, IDH3A, SDHA, SDHB, IDH3G, SDHC, IDH2, 2610507B11RIK, FH1, SUCLA2, MDH1	1594	29	13588	4.70	5.77E-04	2.22E-05	2.92E-04
GOTERM_BP_FAT	GO:0006084~acetyl-CoA metabolic process	16	0.74	4.89E-07	SUCLG1, CS, IDH3B, ACLY, DLAT, IDH3A, SDHA, SDHB, ACSS1, IDH3G, SDHC, IDH2, 2610507B11RIK, FH1, SUCLA2, MDH1	1594	31	13588	4.40	1.78E-03	6.36E-05	9.00E-04
GOTERM_BP_FAT	GO:0006099~tricarboxylic acid cycle	13	0.60	2.53E-06	SUCLG1, CS, IDH3B, IDH3A, SDHA, SDHB, IDH3G, SDHC, IDH2, 2610507B11RIK, FH1, SUCLA2, MDH1	1594	23	13588	4.82	9.15E-03	2.14E-04	4.65E-03
GOTERM_BP_FAT	GO:0046356~acetyl-CoA catabolic process	13	0.60	4.51E-06	SUCLG1, CS, IDH3B, IDH3A, SDHA, SDHB, IDH3G, SDHC, IDH2, 2610507B11RIK, FH1, SUCLA2, MDH1	1594	24	13588	4.62	1.63E-02	3.42E-04	8.30E-03
GOTERM_BP_FAT	GO:0009060~aerobic respiration	13	0.60	2.07E-05	SUCLG1, CS, IDH3B, IDH3A, SDHA, SDHB, IDH3G, SDHC, IDH2, 2610507B11RIK, FH1, SUCLA2, MDH1	1594	27	13588	4.10	7.26E-02	1.28E-03	3.81E-02
Annotation Cluster 8	Enrichment Score: 5.266184011114663											
Category	Term	Count	%	PValue	Genes	List Total	Pop Hits	Pop Total	Fold Enrichment	Bonferroni	Benjamini	FDR
GOTERM_BP_FAT	GO:0006935~chemotaxis	36	1.67	1.25E-08	NRP2, C3AR1, CCL2, CYSLTR1, S100A8, CCR1, CXCL3, SLC37A4, S100A9, CCL9, CCL8, CXCR2, PF4, ITGB2, CCL4, CCL7, ITGAM, CCL6, DOCK2, EAR2, RAC2, CKLF, CSF3R, IL1B, FCER1G, PRKCA, C5AR1, FCGR3, CORO1A, CXCL13, CXCL16, CX3CR1, CMTM7, CMTM3, PTAFR, CMTM6	1594	109	13588	2.82	4.54E-05	2.84E-06	2.30E-05
GOTERM_BP_FAT	GO:0042330~taxis	36	1.67	1.25E-08	NRP2, C3AR1, CCL2, CYSLTR1, S100A8, CCR1, CXCL3, SLC37A4, S100A9, CCL9, CCL8, CXCR2, PF4, ITGB2, CCL4, CCL7, ITGAM, CCL6, DOCK2, EAR2, RAC2, CKLF, CSF3R, IL1B, FCER1G, PRKCA, C5AR1, FCGR3, CORO1A, CXCL13, CXCL16, CX3CR1, CMTM7, CMTM3, PTAFR, CMTM6	1594	109	13588	2.82	4.54E-05	2.84E-06	2.30E-05
GOTERM_BP_FAT	GO:0030595~leukocyte chemotaxis	15	0.70	3.98E-07	PRKCA, C5AR1, CXCL3, CCR1, S100A9, SLC37A4, ITGB2, PF4, ITGAM, FCGR3, CORO1A, CX3CR1, FCER1G, IL1B, CSF3R	1594	27	13588	4.74	1.45E-03	5.37E-05	7.33E-04
GOTERM_BP_FAT	GO:0060326~cell chemotaxis	15	0.70	3.98E-07	PRKCA, C5AR1, CXCL3, CCR1, S100A9, SLC37A4, ITGB2, PF4, ITGAM, FCGR3, CORO1A, CX3CR1, FCER1G, IL1B, CSF3R	1594	27	13588	4.74	1.45E-03	5.37E-05	7.33E-04
Annotation Cluster 9	Enrichment Score: 5.130596702981142											
Category	Term	Count	%	PValue	Genes	List Total	Pop Hits	Pop Total	Fold Enrichment	Bonferroni	Benjamini	FDR
GOTERM_BP_FAT	GO:0001775~cell activation	67	3.11	4.70E-11	GNA13, HPRT, ELF4, STAT5B, PTPN22, TLR4, NFKB2, TPD52, SKAP2, CBFB, TGFB1, CD48, CXCR4, WWP1, IMPDH1, RHOH, EGR1, SATB1, RELB, NFAM1, CD40, MYH9, WAS, PRKCD, IFNAR1, CD86, LAT2, UNC13D, LILRB3, CX3CR1, LCP1, LCP2, ITGAL, SBNO2, LST1, ITGB2, SFPI1, PF4, IL7R, CD74, ITGAM, ADA, SLC11A1, DOCK2, ITGAX, PPP3CB, BCL3, FCER1G, ENTPD1, HELLS, BLNK, EXO1, FYB, TRP53, PTPRC, IKZF1, PLEK, H2-M3, MYO1F, VAV1, FCGR3, HDAC5, FCGR2B, SLC7A2, BAX, PLCG2, H2-DMA	1594	246	13588	2.32	1.71E-07	2.14E-08	8.64E-08

Category	Term	Count	%	PValue	Genes	List Total	Pop Hits	Pop Total	Fold Enrichment	Bonferroni	Benjamini	FDR
GOTERM_BP_FAT	GO:0045321~leukocyte activation	61	2.84	1.44E-10	HPRT, ELF4, STAT5B, PTPN22, TLR4, TPD52, SKAP2, CBFB, TGFB1, CD48, CXCR4, WWP1, IMPDH1, RHOH, EGR1, SATB1, RELB, NFAM1, CD40, MYH9, WAS, PRKCD, IFNAR1, LAT2, CD86, UNC13D, LILRB3, CX3CR1, LCP1, LCP2, ITGAL, SBNO2, SFPI1, ITGB2, IL7R, CD74, ITGAM, ADA, SLC11A1, DOCK2, ITGAX, PPP3CB, BCL3, FCER1G, HELLS, BLNK, EXO1, FYB, TRP53, PTPRC, IKZF1, H2-M3, MYO1F, VAV1, FCGR3, HDAC5, FCGR2B, SLC7A2, BAX, PLCG2, H2-DMA	1594	219	13588	2.37	5.23E-07	5.23E-08	2.64E-07
GOTERM_BP_FAT	GO:0046649~lymphocyte activation	50	2.32	6.88E-08	HPRT, ELF4, STAT5B, PTPN22, TPD52, SKAP2, CBFB, TGFB1, CD48, CXCR4, WWP1, IMPDH1, RHOH, EGR1, SATB1, RELB, NFAM1, CD40, MYH9, PRKCD, WAS, IFNAR1, LAT2, CD86, UNC13D, LCP1, ITGAL, SFPI1, ITGB2, IL7R, ITGAM, ADA, CD74, SLC11A1, DOCK2, ITGAX, PPP3CB, BCL3, HELLS, BLNK, TRP53, EXO1, PTPRC, H2-M3, IKZF1, VAV1, HDAC5, BAX, PLCG2, H2-DMA	1594	191	13588	2.23	2.50E-04	1.09E-05	1.27E-04
GOTERM_BP_FAT	GO:0032943~mononuclear cell proliferation	19	0.88	5.38E-07	TRP53, PTPRC, SATB1, ITGAL, HPRT, ELF4, ITGB2, IL7R, PRKCD, TGFB1, ITGAM, SLC11A1, DOCK2, CD86, ITGAX, CXCR4, BAX, IMPDH1, HELLS	1594	43	13588	3.77	1.95E-03	6.75E-05	9.89E-04
GOTERM_BP_FAT	GO:0070661~leukocyte proliferation	19	0.88	5.38E-07	TRP53, PTPRC, SATB1, ITGAL, HPRT, ELF4, ITGB2, IL7R, PRKCD, TGFB1, ITGAM, SLC11A1, DOCK2, CD86, ITGAX, CXCR4, BAX, IMPDH1, HELLS	1594	43	13588	3.77	1.95E-03	6.75E-05	9.89E-04
GOTERM_BP_FAT	GO:0046651~lymphocyte proliferation	18	0.84	1.96E-06	TRP53, PTPRC, SATB1, ITGAL, HPRT, ELF4, ITGB2, IL7R, PRKCD, ITGAM, SLC11A1, DOCK2, CD86, ITGAX, CXCR4, BAX, IMPDH1, HELLS	1594	42	13588	3.65	7.09E-03	1.98E-04	3.60E-03
GOTERM_BP_FAT	GO:0042110~T cell activation	33	1.53	2.37E-06	ITGAL, ELF4, STAT5B, PTPN22, ITGB2, IL7R, ITGAM, TGFB1, CD74, CD48, SLC11A1, DOCK2, ITGAX, CXCR4, WWP1, PPP3CB, BCL3, RHOH, EGR1, TRP53, PTPRC, SATB1, H2-M3, IKZF1, RELB, MYH9, WAS, VAV1, IFNAR1, CD86, BAX, H2-DMA, LCP1	1594	116	13588	2.43	8.58E-03	2.15E-04	4.35E-03
GOTERM_BP_FAT	GO:0048534~hemopoietic or lymphoid organ development	60	2.79	5.16E-06	GMPR2, STAT5B, PTPN22, NFKB2, PRDX3, TPD52, CBFB, TGFB1, ANK1, TNFRSF11A, WWP1, CASP8, RHOH, EGR1, SGPL1, SATB1, FECH, LYN, RELB, NFAM1, MYH9, TACC3, DNASE2A, HIF1A, LILRB3, CCR2, SIX1, VEGFA, CCR1, SLC37A4, SFXN1, SFPI1, SOX6, IL7R, CD74, ADA, MLF1, DOCK2, PPP3CB, BCL3, RUNX1, HELLS, CEBPA, TRP53, PTPRC, IKZF1, HCLS1, BPGM, VAV1, SOD2, HDAC5, PLSCR1, CCNB2, ID2, CXCL13, BAX, PLCG2, IRF8, JAK2, H2-DMA	1594	281	13588	1.82	1.86E-02	3.83E-04	9.49E-03
GOTERM_BP_FAT	GO:0030097~hemopoiesis	55	2.56	6.08E-06	GMPR2, STAT5B, PTPN22, PRDX3, TPD52, CBFB, TGFB1, ANK1, WWP1, CASP8, RHOH, EGR1, SGPL1, SATB1, FECH, LYN, RELB, NFAM1, MYH9, TACC3, DNASE2A, HIF1A, LILRB3, VEGFA, CCR2, CCR1, SLC37A4, SFXN1, SFPI1, SOX6, IL7R, ADA, CD74, MLF1, DOCK2, PPP3CB, BCL3, RUNX1, HELLS, CEBPA, TRP53, PTPRC, IKZF1, HCLS1, BPGM, VAV1, SOD2, HDAC5, PLSCR1, ID2, BAX, IRF8, PLCG2, JAK2, H2-DMA	1594	251	13588	1.87	2.19E-02	4.34E-04	1.12E-02
GOTERM_BP_FAT	GO:0002520~immune system development	61	2.84	1.22E-05	GMPR2, STAT5B, PTPN22, NFKB2, PRDX3, TPD52, CBFB, TGFB1, ANK1, TNFRSF11A, WWP1, CASP8, RHOH, EGR1, SGPL1, SATB1, FECH, LYN, RELB, NFAM1, MYH9, TACC3, DNASE2A, HIF1A, LILRB3, CCR2, SIX1, VEGFA, CCR1, SLC37A4, SFXN1, SFPI1, SOX6, IL7R, CD74, ADA, MLF1, DOCK2, PPP3CB, BCL3, RUNX1, HELLS, EXO1, CEBPA, TRP53, PTPRC, IKZF1, HCLS1, BPGM, VAV1, SOD2, HDAC5, PLSCR1, CCNB2, ID2, CXCL13, BAX, PLCG2, IRF8, JAK2, H2-DMA	1594	295	13588	1.76	4.33E-02	7.90E-04	2.24E-02
Annotation Cluster 10	Enrichment Score: 4.737722967929924											
Category	Term	Count	%	PValue	Genes	List Total	Pop Hits	Pop Total	Fold Enrichment	Bonferroni	Benjamini	FDR
GOTERM_BP_FAT	GO:0015980~energy derivation by oxidation of organic compounds	39	1.81	5.03E-12	PRKAG3, UQCRC1, PHKB, NDUFB8, PHKA1, SLC37A4, UQCR11, GOT1, PPP1R3B, IDH3G, PPP1R1A, GYS1, IDH2, FH1, SUCLA2, NDUFS1, AGL, NDUFA5, PHKG1, ALDH5A1, SUCLG1, EPM2A, CS, IDH3B, PPP1R3A, IDH3A, SOD2, SDHA, SLC25A12, SDHB, SLC25A13, PYGL, SDHC, SLC37A2, DLD, 2610507B11RIK, UQCRB, MDH1, PYGB	1594	98	13588	3.39	1.83E-08	3.66E-09	9.25E-09
Annotation Cluster 11	Enrichment Score: 4.5444652420965435											
Category	Term	Count	%	PValue	Genes	List Total	Pop Hits	Pop Total	Fold Enrichment	Bonferroni	Benjamini	FDR
GOTERM_MF_FAT	GO:0051015~actin filament binding	21	0.98	1.43E-07	MYO5A, DBNL, LIMA1, ACTN4, AIF1, MYH2, FSCN1, ACTN1, MYO9B, MYH9, GAS7, MYH8, FLNA, PKNOX2, CORO1A, EZR, LASP1, AIF1L, LRPPRC, LCP1, MYO5C	1524	49	13288	3.74	1.84E-04	1.84E-05	2.33E-04
Annotation Cluster 12	Enrichment Score: 4.414295044097882											
Category	Term	Count	%	PValue	Genes	List Total	Pop Hits	Pop Total	Fold Enrichment	Bonferroni	Benjamini	FDR
GOTERM_BP_FAT	GO:0002250~adaptive immune response	27	1.26	2.00E-06	IL18, NFKB2, TLR6, C1QC, CD74, SLC11A1, MYD88, KLHL6, FCER1G, BCL3, INPP5D, EXO1, ICAM1, CFB, RELB, PRKCD, FCGR1, FCGR3, C1QA, C1QB, UNC13D, FCGR2B, LILRB3, IRF7, VEGFA, H2-AA, H2-DMA	1594	84	13588	2.74	7.26E-03	1.92E-04	3.68E-03
GOTERM_BP_FAT	GO:0002460~adaptive immune response based on somatic recombination of immune receptors built fr	27	1.26	2.00E-06	IL18, NFKB2, TLR6, C1QC, CD74, SLC11A1, MYD88, KLHL6, FCER1G, BCL3, INPP5D, EXO1, ICAM1, CFB, RELB, PRKCD, FCGR1, FCGR3, C1QA, C1QB, UNC13D, FCGR2B, LILRB3, IRF7, VEGFA, H2-AA, H2-DMA	1594	84	13588	2.74	7.26E-03	1.92E-04	3.68E-03
GOTERM_BP_FAT	GO:0002252~immune effector process	35	1.63	2.00E-06	BNIP3, IL7R, TLR7, C1QC, TLR8, CD74, CFP, SLC11A1, MYD88, FCER1G, BCL3, INPP5D, PTX3, EXO1, PTPRC, PTPN6, ICAM1, CARD9, CFB, NCF1, SAMHD1, MYO1F, PRKCD, FCGR1, FCGR3, C1QA, C1QB, ABCC9, LAT2, UNC13D, FCGR2B, LILRB3, IRF7, H2-AA, H2-DMA	1594	126	13588	2.37	7.26E-03	1.87E-04	3.68E-03
Annotation Cluster 13	Enrichment Score: 4.305942935764653											
Category	Term	Count	%	PValue	Genes	List Total	Pop Hits	Pop Total	Fold Enrichment	Bonferroni	Benjamini	FDR
GOTERM_BP_FAT	GO:0016044~membrane organization	61	2.84	7.88E-07	CD209A, LDLR, HIP1R, SORL1, BNIP3, NRCAM, DAB2, AP1S1, CTTN, NECAP2, CAP1, GHR, DBNL, CCDC88A, MYH2, LPCAT2, SIRPA, LDLRAP1, ELMO1, MFN2, MFN1, UNC13D, SGCG, NME1, LRP12, RIN2, RIN1, STEAP2, ANKFY1, SGCA, STON2, BID, PPT1, CD9, SLC11A1, DOCK2, FCER1G, PTX3, EHD1, MRC1, TRP53, VAV3, HCK, LMNA, SUN2, STAB2, FCGR1, VAV1, FCGR3, CORO1C, NRAS, KCNN4, LRP1, FCGR2B, ULK1, BAX, RAB34, IRF8, CLEC7A, MERTK, CLCN5	1594	272	13588	1.91	2.86E-03	8.96E-05	1.45E-03
GOTERM_BP_FAT	GO:0006897~endocytosis	43	2.00	2.50E-05	STON2, CD209A, LDLR, HIP1R, SORL1, PPT1, SLC11A1, DAB2, CTTN, AP1S1, NECAP2, FCER1G, CAP1, EHD1, PTX3, GHR, MRC1, DBNL, HCK, STAB2, FCGR1, SIRPA, VAV1, LDLRAP1, FCGR3, ELMO1, CORO1C, NRAS, LRP1, UNC13D, FCGR2B, ULK1, NME1, LRP12, RAB34, IRF8, RIN2, RIN1, CLEC7A, STEAP2, MERTK, ANKFY1, CLCN5	1594	188	13588	1.95	8.69E-02	1.46E-03	4.59E-02
GOTERM_BP_FAT	GO:0010324~membrane invagination	43	2.00	2.50E-05	STON2, CD209A, LDLR, HIP1R, SORL1, PPT1, SLC11A1, DAB2, CTTN, AP1S1, NECAP2, FCER1G, CAP1, EHD1, PTX3, GHR, MRC1, DBNL, HCK, STAB2, FCGR1, SIRPA, VAV1, LDLRAP1, FCGR3, ELMO1, CORO1C, NRAS, LRP1, UNC13D, FCGR2B, ULK1, NME1, LRP12, RAB34, IRF8, RIN2, RIN1, CLEC7A, STEAP2, MERTK, ANKFY1, CLCN5	1594	188	13588	1.95	8.69E-02	1.46E-03	4.59E-02
Annotation Cluster 14	Enrichment Score: 4.221758640092546											
Category	Term	Count	%	PValue	Genes	List Total	Pop Hits	Pop Total	Fold Enrichment	Bonferroni	Benjamini	FDR
GOTERM_BP_FAT	GO:0007049~cell cycle	120	5.58	9.58E-09	KIFC1, S100A6, PRC1, DBF4, PTTG1, FOXO4, CDCA8, CUL7, INCENP, MAP3K8, ATF6B, CDCA2, MTUS1, CDCA3, PDPN, SGOL2, SKP2, ESPL1, GPR132, PLK1S1, TACC3, MYH9, FOXN3, UHRF1, MAD2L1, RCC2, MAPK6, ZWINT, STMN1, MAPK7, PPP2R3A, NEK2, CHEK1, ANLN, CCNG1, SPC25, NCAPG2, TFDP2, FBXO5, SKA1, LFNG, HELLS, ERCC6L, TRP53, CKAP2, CCPG1, IKZF1, MKI67, AK1, BRCA1, NOTCH2, CDKN1A, PLK3, PLK2, RGS2, FBXO31, CHAF1A, CHAF1B, E2F1, GAS2L3, E2F6, E2F8, CUZD1, CDT1, CCNE1, CGREF1, TUBB5, RANBP1, SIK1, CDC7, CDC6, ARHGEF2, CCNK, KIF11, NUSAP1, 1700017B05RIK, HMGA2, MCM3, CDK4, UBE2C, UBE2B, CDK2, JMY, MCM6, ARL3, RBBP8, MFN2, CCND1, BUB1B, RBM38, GADD45A, CKS1B, CAMK2G, POLA1, MLF1, NCAPH, BUB1, PPP3CA, ZWILCH, CAMK2A, EXO1, BOD1, KAT2B, NASP, ANXA1, BIRC5, SIRT7, RACGAP1, SMC2, RGS14, SMC4, RASSF4, CCNB2, MAPK12, RASSF2, CKS2, PHGDH, KIF20B, GAS2L1, CHTF18	1594	611	13588	1.67	3.49E-05	2.49E-06	1.76E-05
GOTERM_BP_FAT	GO:0000278~mitotic cell cycle	55	2.56	2.50E-06	KIFC1, E2F6, DBF4, PTTG1, FOXO4, CDCA8, INCENP, ATF6B, CDCA2, CDCA3, CDC6, ARHGEF2, CCNK, KIF11, SKP2, NUSAP1, GPR132, UBE2C, HMGA2, CDK2, RBBP8, CCND1, MAD2L1, RCC2, ZWINT, BUB1B, STMN1, GADD45A, PPP2R3A, NEK2, CAMK2G, POLA1, ANLN, CHEK1, CCNG1, SPC25, NCAPH, NCAPG2, BUB1, FBXO5, SKA1, PPP3CA, ZWILCH, CAMK2A, HELLS, ERCC6L, BOD1, BIRC5, SIRT7, SMC2, RGS14, SMC4, CCNB2, PLK2, KIF20B	1594	244	13588	1.92	9.05E-03	2.16E-04	4.59E-03
GOTERM_BP_FAT	GO:0022402~cell cycle process	78	3.63	3.25E-06	GAS2L3, KIFC1, E2F6, DBF4, PTTG1, FOXO4, CGREF1, CDCA8, INCENP, ATF6B, TUBB5, CDCA2, RANBP1, CDCA3, CDC6, ARHGEF2, CCNK, KIF11, SGOL2, SKP2, NUSAP1, ESPL1, GPR132, PLK1S1, MYH9, HMGA2, TACC3, UBE2C, UBE2B, CDK2, JMY, RBBP8, MFN2, CCND1, MAD2L1, RCC2, ZWINT, BUB1B, STMN1, GADD45A, PPP2R3A, NEK2, CAMK2G, POLA1, CHEK1, ANLN, CCNG1, MLF1, SPC25, NCAPH, NCAPG2, BUB1, FBXO5, SKA1, PPP3CA, ZWILCH, LFNG, CAMK2A, HELLS, ERCC6L, EXO1, BOD1, MKI67, AK1, BIRC5, SIRT7, RACGAP1, SMC2, BRCA1, RGS14, SMC4, NOTCH2, CDKN1A, CCNB2, PHGDH, KIF20B, CKS2, GAS2L1	1594	393	13588	1.69	1.18E-02	2.57E-04	5.99E-03
GOTERM_BP_FAT	GO:0022403~cell cycle phase	65	3.02	2.50E-05	KIFC1, E2F6, DBF4, PTTG1, FOXO4, CDCA8, INCENP, ATF6B, TUBB5, CDCA2, RANBP1, CDCA3, CDC6, ARHGEF2, CCNK, KIF11, SGOL2, SKP2, NUSAP1, GPR132, ESPL1, MYH9, UBE2C, TACC3, HMGA2, UBE2B, CDK2, RBBP8, CCND1, MAD2L1, RCC2, ZWINT, BUB1B, STMN1, GADD45A, PPP2R3A, NEK2, CAMK2G, POLA1, CHEK1, ANLN, CCNG1, SPC25, NCAPH, NCAPG2, BUB1, FBXO5, SKA1, PPP3CA, ZWILCH, LFNG, CAMK2A, HELLS, ERCC6L, EXO1, BOD1, MKI67, BIRC5, SIRT7, SMC2, RGS14, SMC4, CCNB2, KIF20B, CKS2	1594	328	13588	1.69	8.68E-02	1.49E-03	4.59E-02
Annotation Cluster 15	Enrichment Score: 4.1734066916287205											
Category	Term	Count	%	PValue	Genes	List Total	Pop Hits	Pop Total	Fold Enrichment	Bonferroni	Benjamini	FDR
GOTERM_MF_FAT	GO:0030246~carbohydrate binding	64	2.98	7.74E-06	CD209A, POSTN, FSTL1, GPCPD1, CANX, SERPINE2, CD93, CD44, APOE, CLEC4A1, CLEC4A2, CLEC4D, CLEC4A3, MAN2B1, BSG, PICK1, M6PR, CLEC4N, VEGFB, MAN2A2, SIGLEC1, MAN2A1, EGFLAM, BGN, CLEC12A, NAV2, CD33, VEGFA, VCAN, LAMC2, CLEC5A, SIGLECE, GALNT7, HSD17B12, CCL8, PF4, CALR, CLEC10A, CCL7, ITGAM, HMMR, SMOC2, PTX3, THBS1, GPNMB, SELPLG, AGL, FN1, MRC1, LPL, PTPRC, LMAN1L, LGALS3, SELL, LGALS1, EPM2A, FBN1, CHI3L3, STAB2, LGALS9, LYVE1, STAB1, LAYN, CLEC7A	1524	317	13288	1.76	9.89E-03	5.52E-04	1.26E-02
GOTERM_MF_FAT	GO:0001871~pattern binding	33	1.53	1.39E-05	HSD17B12, CCL8, POSTN, PF4, FSTL1, CCL7, ITGAM, HMMR, SMOC2, CD44, SERPINE2, APOE, GPNMB, THBS1, PTX3, AGL, FN1, LPL, PTPRC, FBN1, CHI3L3, STAB2, VEGFB, LYVE1, BGN, EGFLAM, STAB1, LAYN, NAV2, VEGFA, LAMC2, VCAN, CLEC7A	1524	128	13288	2.25	1.76E-02	8.47E-04	2.26E-02
GOTERM_MF_FAT	GO:0030247~polysaccharide binding	33	1.53	1.39E-05	HSD17B12, CCL8, POSTN, PF4, FSTL1, CCL7, ITGAM, HMMR, SMOC2, CD44, SERPINE2, APOE, GPNMB, THBS1, PTX3, AGL, FN1, LPL, PTPRC, FBN1, CHI3L3, STAB2, VEGFB, LYVE1, BGN, EGFLAM, STAB1, LAYN, NAV2, VEGFA, LAMC2, VCAN, CLEC7A	1524	128	13288	2.25	1.76E-02	8.47E-04	2.26E-02
Annotation Cluster 16	Enrichment Score: 3.9596571907277394											
Category	Term	Count	%	PValue	Genes	List Total	Pop Hits	Pop Total	Fold Enrichment	Bonferroni	Benjamini	FDR
GOTERM_BP_FAT	GO:0007264~small GTPase mediated signal transduction	58	2.70	1.40E-06	GNA13, RAB5C, RASGEF1B, RRAD, RHOQ, IQGAP2, RHOV, RGL1, IQGAP1, RAB28, HMOX1, ARHGAP1, RHOA, RALB, RHOC, RHOG, RHOH, RAP2B, DBNL, RAP2A, BAIAP2, RAB4B, GEM, ARHGEF9, ARL3, ELMO1, DNAJC27, DOK1, RND3, DOK2, DOK3, HRASLS, RRAS2, SDCBP, RAB12, ARL4C, 5430435G22RIK, ARF6, RAB44, RAB43, RAC2, RASGRP3, SOS2, ARHGDIA, RAB8A, RAB8B, VAV3, NRAS, RAB32, RAB31, ULK1, ARF3, ARF4, RAB34, YWHAQ, RHOT2, RAP1B, BCAR3	1594	258	13588	1.92	5.09E-03	1.50E-04	2.58E-03
Annotation Cluster 17	Enrichment Score: 3.651934493413683											
Category	Term	Count	%	PValue	Genes	List Total	Pop Hits	Pop Total	Fold Enrichment	Bonferroni	Benjamini	FDR
GOTERM_BP_FAT	GO:0043068~positive regulation of programmed cell death	56	2.60	2.45E-06	PDIA3, IL18, MMP9, BNIP3, IL10, TGFB1, GPX1, CASP6, CASP3, CASP4, CASP9, BOK, APOE, HMOX1, NGFRAP1, NDUFS3, CASP1, MYC, PRKCA, CYCS, SKP2, NDUFA13, PRKCD, JMY, UNC13D, RIPK3, BID, CDK5R1, LST1, ADAMTSL4, STK17B, SP110, BCL2L1, ALDH1A1, SHISA5, PYCARD, BCL3, INPP5D, DYRK2, TRP53, COL18A1, PTPRC, PTPN6, KLF10, NR4A1, FCGR1, BRCA1, FCGR3, TRADD, NOTCH2, CDKN1A, UACA, BAX, ID3, APAF1, IFI204	1594	250	13588	1.91	8.88E-03	2.18E-04	4.51E-03
GOTERM_BP_FAT	GO:0010942~positive regulation of cell death	56	2.60	3.17E-06	PDIA3, IL18, MMP9, BNIP3, IL10, TGFB1, GPX1, CASP6, CASP3, CASP4, CASP9, BOK, APOE, HMOX1, NGFRAP1, NDUFS3, CASP1, MYC, PRKCA, CYCS, SKP2, NDUFA13, PRKCD, JMY, UNC13D, RIPK3, BID, CDK5R1, LST1, ADAMTSL4, STK17B, SP110, BCL2L1, ALDH1A1, SHISA5, PYCARD, BCL3, INPP5D, DYRK2, TRP53, COL18A1, PTPRC, PTPN6, KLF10, NR4A1, FCGR1, BRCA1, FCGR3, TRADD, NOTCH2, CDKN1A, UACA, BAX, ID3, APAF1, IFI204	1594	252	13588	1.89	1.15E-02	2.56E-04	5.83E-03
GOTERM_BP_FAT	GO:0043065~positive regulation of apoptosis	55	2.56	4.18E-06	PDIA3, IL18, MMP9, BNIP3, IL10, TGFB1, GPX1, CASP6, CASP3, CASP4, CASP9, BOK, APOE, HMOX1, NGFRAP1, NDUFS3, CASP1, MYC, PRKCA, CYCS, SKP2, NDUFA13, PRKCD, JMY, UNC13D, RIPK3, BID, CDK5R1, LST1, ADAMTSL4, STK17B, SP110, BCL2L1, ALDH1A1, SHISA5, PYCARD, BCL3, INPP5D, DYRK2, TRP53, COL18A1, PTPN6, PTPRC, KLF10, NR4A1, FCGR1, BRCA1, FCGR3, TRADD, NOTCH2, UACA, BAX, ID3, APAF1, IFI204	1594	248	13588	1.89	1.51E-02	3.24E-04	7.69E-03
Annotation Cluster 18	Enrichment Score: 3.383098795598096											
Category	Term	Count	%	PValue	Genes	List Total	Pop Hits	Pop Total	Fold Enrichment	Bonferroni	Benjamini	FDR

GOTERM_MF_FAT	GO:0016791~phosphatase activity	50	2.32	2.99E-05	PPIP5K2, PGAM1, PTPN22, PGAM2, PPIP5K1, NT5C, PTPRJ, PPAP2C, FBP2, PPP1R3A, LPIN2, PTPRO, LPIN3, PPM1H, PPM1L, PPM1M, PFKFB3, NT5C1A, 9630033F20RIK, PFKFB1, PPM1A, ACP5, PTPLB, PPP3CB, PPP3CA, INPP5D, PPTC7, PTPN7, PTPN6, PTPRC, PTPRE, PTPN18, PTPN3, PTPN4, PTPRA, EPM2A, EPHX2, DUSP23, ATP1A1, BPGM, PTPN12, DUSP5, DUSP4, DUSP28, PTP4A3, DUSP26, PTPN1, PHPT1, DUSP8, DUSP6	1524	238	13288	1.83	3.77E-02	1.54E-03	4.88E-02
Annotation Cluster 19	Enrichment Score: 3.3671298805420657											
Category	Term	Count	%	PValue	Genes	List Total	Pop Hits	Pop Total	Fold Enrichment	Bonferroni	Benjamini	FDR
GOTERM_BP_FAT	GO:0006631~fatty acid metabolic process	48	2.23	1.46E-07	MYO5A, PRKAG3, SLC27A1, PPARA, ACOX1, ACADSB, ECH1, CPT2, STAT5B, NDUFAB1, ECHS1, CD74, HADHA, HADHB, SC4MOL, FAR1, ELOVL1, PRKAR2B, TNFRSF1A, ALOX5AP, PRKAA2, ELOVL6, HADH, HPGDS, GHR, ACSL5, SCD1, CPT1B, ACAA2, SCD2, ACADM, TBXAS1, NCF1, ALDH5A1, ACADS, PDPN, FADS1, PRKAB2, EPHX2, FADS3, CRAT, PHYH, CPT1A, BRCA1, ACADVL, ALOX5, AACS, DEGS1	1594	184	13588	2.22	5.31E-04	2.12E-05	2.68E-04
Annotation Cluster 25	Enrichment Score: 2.4724967356094183											
Category	Term	Count	%	PValue	Genes	List Total	Pop Hits	Pop Total	Fold Enrichment	Bonferroni	Benjamini	FDR
GOTERM_BP_FAT	GO:0051130~positive regulation of cellular component organization	32	1.49	2.08E-05	LST1, TNFRSF12A, PPT1, CALR, CDH4, TGFB1, DSTN, SLC11A1, ARPC2, RHOA, FCER1G, RANBP1, PTX3, SYNPO, TES, TRP53, PLEK, ACTN4, LIMK1, PLXNB2, NUSAP1, CD63, LDLRAP1, SIRPA, FCGR1, FCGR3, FMN1, PLK4, FCGR2B, TPPP, CFL1, CLEC7A	1594	122	13588	2.24	7.30E-02	1.26E-03	3.83E-02
Annotation Cluster 27	Enrichment Score: 2.4478334888926443											
Category	Term	Count	%	PValue	Genes	List Total	Pop Hits	Pop Total	Fold Enrichment	Bonferroni	Benjamini	FDR
GOTERM_BP_FAT	GO:0051493~regulation of cytoskeleton organization	29	1.35	5.90E-06	LIMA1, CAPZA1, ARPC5, VILL, DSTN, ACTR3, ARPC3, ARPC2, RHOA, RANBP1, SKA1, SYNPO, TES, DBNL, ARHGEF2, CCDC88A, PLEK, CEP192, MYO1F, TMSB10, MID1IP1, FMN1, ARPC1B, PLK4, CORO1A, CFL1, CAPG, TMSB4X, STMN1	1594	99	13588	2.50	2.12E-02	4.29E-04	1.08E-02
GOTERM_BP_FAT	GO:0032956~regulation of actin cytoskeleton organization	21	0.98	8.63E-06	DBNL, LIMA1, CCDC88A, PLEK, CAPZA1, MYO1F, TMSB10, ARPC5, VILL, DSTN, ACTR3, FMN1, ARPC1B, CORO1A, ARPC3, ARPC2, CFL1, CAPG, RHOA, TMSB4X, SYNPO	1594	60	13588	2.98	3.09E-02	6.04E-04	1.59E-02
GOTERM_BP_FAT	GO:0032970~regulation of actin filament-based process	21	0.98	1.14E-05	DBNL, LIMA1, CCDC88A, PLEK, CAPZA1, MYO1F, TMSB10, ARPC5, VILL, DSTN, ACTR3, FMN1, ARPC1B, CORO1A, ARPC3, ARPC2, CFL1, CAPG, RHOA, TMSB4X, SYNPO	1594	61	13588	2.93	4.08E-02	7.56E-04	2.10E-02
GOTERM_BP_FAT	GO:0033043~regulation of organelle organization	38	1.77	1.37E-05	LIMA1, LST1, CAPZA1, ARPC5, TGFB1, VILL, DSTN, ACTR3, CUL7, ARPC3, ARPC2, RHOA, RANBP1, SKA1, TES, SYNPO, TRP53, DBNL, ARHGEF2, CCDC88A, PLEK, CEP192, NUSAP1, MYO1F, TMSB10, HERC2, MID1IP1, UBE2B, FMN1, ARPC1B, CORO1A, PLK4, YWHAH, MAD2L1, CFL1, CAPG, TMSB4X, STMN1	1594	154	13588	2.10	4.88E-02	8.77E-04	2.53E-02
Annotation Cluster 30	Enrichment Score: 2.3517354573541587											
Category	Term	Count	%	PValue	Genes	List Total	Pop Hits	Pop Total	Fold Enrichment	Bonferroni	Benjamini	FDR
GOTERM_MF_FAT	GO:0030695~GTPase regulator activity	70	3.25	1.14E-05	RASGEF1B, ARHGAP18, IQGAP2, ARHGAP15, RGL1, IQGAP1, TBC1D16, HMHA1, ARHGAP22, DMXL2, ARHGAP20, RINL, ARHGAP1, RANBP1, ARHGAP11A, DOCK10, DOCK11, DAB2IP, ARHGEF2, MADD, ARHGEF6, RAB3IL1, ARHGAP28, PSD4, ARHGEF9, ECT2, WAS, THY1, ARHGAP25, ARHGAP30, MAP4K4, RIN2, SRGAP3, RIN1, FGD2, ARFGAP3, TBC1D9, GPSM3, CYTH4, MAP4K1, MYO9B, ADAP1, DOCK2, RASGRP3, GMIP, SOS2, RASAL3, IQSEC3, RASA3, RAP1GAP2, FGD3, ARHGDIA, ARHGDIB, OBSCN, TBC1D2B, VAV3, ABR, EXPH5, RGS18, DOCK8, RGS16, RACGAP1, DOCK5, VAV1, RGS14, ADAP2, RGS2, SH3BP1, ARAP1, BCAR3	1524	361	13288	1.69	1.45E-02	7.32E-04	1.86E-02
GOTERM_MF_FAT	GO:0060589~nucleoside-triphosphatase regulator activity	70	3.25	2.01E-05	RASGEF1B, ARHGAP18, IQGAP2, ARHGAP15, RGL1, IQGAP1, TBC1D16, HMHA1, ARHGAP22, DMXL2, ARHGAP20, RINL, ARHGAP1, RANBP1, ARHGAP11A, DOCK10, DOCK11, DAB2IP, ARHGEF2, MADD, ARHGEF6, RAB3IL1, ARHGAP28, PSD4, ARHGEF9, ECT2, WAS, THY1, ARHGAP25, ARHGAP30, MAP4K4, RIN2, SRGAP3, RIN1, FGD2, ARFGAP3, TBC1D9, GPSM3, CYTH4, MAP4K1, MYO9B, ADAP1, DOCK2, RASGRP3, GMIP, SOS2, RASAL3, IQSEC3, RASA3, RAP1GAP2, FGD3, ARHGDIA, ARHGDIB, OBSCN, TBC1D2B, VAV3, ABR, EXPH5, RGS18, DOCK8, RGS16, RACGAP1, DOCK5, VAV1, RGS14, ADAP2, RGS2, SH3BP1, ARAP1, BCAR3	1524	367	13288	1.66	2.55E-02	1.07E-03	3.27E-02
Annotation Cluster 32	Enrichment Score: 2.1864103669637593											
Category	Term	Count	%	PValue	Genes	List Total	Pop Hits	Pop Total	Fold Enrichment	Bonferroni	Benjamini	FDR
GOTERM_BP_FAT	GO:0002684~positive regulation of immune system process	50	2.32	8.11E-07	IL18, STAT5B, HPS1, TLR2, PTPN22, TLR4, C1QC, PNP, TLR8, TGFB1, B2M, CFP, KLHL6, MYD88, HSP90AA1, LYN, NFAM1, CD40, THY1, C1QA, C1QB, LAT2, CD37, H2-AA, ITGAL, IL4RA, UNC93B1, IL7R, ADA, CD74, SLC11A1, FCER1G, IL2RG, SH2B2, INPP5D, THBS1, PTPN6, PTPRC, CARD9, H2-M3, IKZF1, CFB, FCGR1, FCGR3, CORO1A, CDKN1A, PLCG2, CLEC7A, H2-DMA, SASH3	1594	206	13588	2.07	2.95E-03	8.94E-05	1.49E-03
Annotation Cluster 58	Enrichment Score: 1.5613806466898854											
Category	Term	Count	%	PValue	Genes	List Total	Pop Hits	Pop Total	Fold Enrichment	Bonferroni	Benjamini	FDR
GOTERM_BP_FAT	GO:0044271~nitrogen compound biosynthetic process	61	2.84	2.55E-05	ADCY7, ATP1B3, HPRT, ATP1B2, LDHD, PNP, MTHFD1L, ANK1, GOT1, HDC, IMPDH1, ATP5K, ATP8B4, HSP90AA1, FECH, ATP6V1H, PADI2, PADI4, ATP6V1A, NME1, ADK, RRM2, ATP5C1, ADSL, BCAT1, ALDH18A1, BCAT2, SRM, NAGS, ASNSD1, ASNS, AZIN1, CMPK1, ADA, TYMS, ATP6V0E, ALDH4A1, DCTD, TCIRG1, CEBPA, ODC1, ADSSL1, NOS1, NPR2, ATP1A1, ATP13A3, AMPD2, AMPD3, MMAB, ATP13A4, APRT, AMPD1, ADI1, P2RX4, ATP6V0E2, ADCY9, ATP2A2, SLC7A2, PHGDH, PSAT1, ATP8A1	1594	302	13588	1.72	8.85E-02	1.47E-03	4.68E-02
Annotation Cluster 69	Enrichment Score: 1.2861838620748725											
Category	Term	Count	%	PValue	Genes	List Total	Pop Hits	Pop Total	Fold Enrichment	Bonferroni	Benjamini	FDR
GOTERM_BP_FAT	GO:0009081~branched chain family amino acid metabolic process	10	0.46	1.06E-05	BCAT1, BCKDHA, ALDH6A1, BCKDK, BCAT2, STAT5B, BCKDHB, HIBADH, GHR, AUH	1594	15	13588	5.68	3.77E-02	7.12E-04	1.94E-02

Table S1.E GO Terms upregulated 72 hours post injury

Annotation Cluster 1 — Enrichment Score: 19.262320577011828

Category	Term	Count	%	PValue	Genes	List Total	Pop Hits	Pop Total	Fold Enrichment	Bonferroni	Benjamini	FDR
GOTERM_BP_FAT	GO:0009611~response to wounding	122	4.29	1.49E-21	F13A1, TLR1, F2RL1, TLR2, TLR3, TLR4, TLR5, TLR6, TLR7, C1QC, TGFB1, TLR8, TLR9, CFP, C1RB, NLRC4, MYD88, CD44, APOH, IL1B, TFPI2, RAB27A, CIITA, F10, GATM, NCF1, C4B, F7, PLAUR, C1QA, C1QB, HIF1A, UNC13D, CCR5, CCR2, CCL3, CCL2, NFKBID, NINJ1, CCL8, ITGB2, CCL5, CCL4, CCL7, TRF, ARG1, SLC11A1, MTPN, CNR2, ENTPD1, FN1, TLR12, PTPN6, KLF6, CFB, EFEMP2, TLR13, SPHK1, EPHX2, CHI3L3, FCGR1, FCGR3, LAT, NOTCH2, GGT5, P2RX7, CXCL13, CLEC7A, LY86, HPS1, GJA1, GPX1, NOD2, CASP3, SLC1A3, TICAM1, TICAM2, LBP, NFKBIZ, LY96, CHST2, SAA3, GAL, CD163, CHST1, SERPINA3N, NPPB, CTSB, PROS1, LCP1, PTAFR, GAP43, CXCL1, TNF, CXCL5, C3, CCR1, CXCL9, ABHD2, PF4, TNFRSF1A, ANXA8, CCL22, TNFRSF1B, MEFV, PYCARD, GP1BA, THBS1, PAPSS2, SELP, IL6, LIPA, PLEK, ANXA5, CD180, P2RY12, CCL12, STAB1, C1RL, ALOX5, CD14, IGFBP4	2002	347	13588	2.39	6.03E-18	2.01E-18	2.77E-18
GOTERM_BP_FAT	GO:0006952~defense response	140	4.93	2.50E-19	TLR1, PGLYRP1, TLR2, TLR3, TLR4, TLR5, TLR6, TLR7, C1QC, TLR8, TGFB1, TAPBP, TLR9, B2M, CFP, C1RB, NLRC4, MYD88, CD44, IL1B, MX1, MX2, SPN, RAB27A, CIITA, C5AR1, NCF1, C4B, IFI47, C1QA, C1QB, HIF1A, UNC13D, CCR5, CCR2, TNFAIP8L2, IFIH1, IL1R1, CCL3, CCL2, NFKBID, FGR, CCL8, ITGB2, SP110, CCL5, CCL4, TRF, CCL7, CD74, SLC11A1, CNR2, FCER1G, DHX58, FN1, TLR12, PTPN6, CARD9, CFB, IL1RL1, IL1RL2, TLR13, SPHK1, EPHX2, MYO1F, CHI3L3, VDAC3, FCGR1, H2-Q7, FCGR3, VDAC1, DDX58, LAT, GGT5, P2RX7, KCNJ8, CXCL13, CLEC7A, IL27RA, LY86, H2-D1, BNIP3, CD1D1, TMEM173, NOD2, TICAM1, TICAM2, LBP, H2-K1, NFKBIZ, IL18RAP, LY96, CHST2, SAA3, CLIC1, GAL, ECSIT, CD163, CHST1, SERPINA3N, NPPB, H2-AA, PTAFR, CXCL1, TNF, CXCL5, C3, CCR1, CXCL9, RSAD2, NLRP1A, NLRP1B, TNFRSF1A, TNFRSF1B, CCL22, MEFV, TAP1, PYCARD, BCL3, THBS1, PTPRC, SELP, IRGM1, IL6, LYZ2, LIPA, H2-M3, HCK, SAMHD1, COTL1, CD180, CCL12, FCGR2B, STAB1, IRF8, C1RL, APAF1, ALOX5, IGFBP4, CD14	2002	448	13588	2.12	1.01E-15	2.03E-16	4.65E-16
GOTERM_BP_FAT	GO:0006954~inflammatory response	88	3.10	4.40E-19	LY86, TLR1, TLR2, TLR3, TLR4, TLR5, TLR6, TLR7, C1QC, TGFB1, TLR8, TLR9, CFP, NOD2, C1RB, NLRC4, MYD88, CD44, TICAM1, TICAM2, IL1B, LBP, CIITA, NFKBIZ, C4B, NCF1, LY96, SAA3, CHST2, GAL, CD163, CHST1, C1QA, C1QB, SERPINA3N, UNC13D, HIF1A, CCR5, CCR2, NPPB, PTAFR, CXCL1, CCL3, TNF, CCL2, CXCL5, NFKBID, C3, CCR1, CXCL9, CCL8, ITGB2, CCL5, CCL4, CCL7, TRF, SLC11A1, TNFRSF1A, CCL22, TNFRSF1B, MEFV, CNR2, PYCARD, THBS1, FN1, TLR12, SELP, IL6, LIPA, CFB, SPHK1, TLR13, EPHX2, CHI3L3, FCGR1, CD180, FCGR3, LAT, GGT5, CCL12, P2RX7, STAB1, CXCL13, C1RL, ALOX5, CLEC7A, CD14, IGFBP4	2002	225	13588	2.65	1.79E-15	2.98E-16	8.18E-16

Annotation Cluster 2 — Enrichment Score: 10.46671132690926

Category	Term	Count	%	PValue	Genes	List Total	Pop Hits	Pop Total	Fold Enrichment	Bonferroni	Benjamini	FDR
GOTERM_BP_FAT	GO:0048002~antigen processing and presentation of peptide antigen	27	0.95	3.72E-15	UNC93B1, H2-D1, IFI30, CALR, CD74, B2M, TAPBP, SLC11A1, TAP2, FCER1G, 1500011B03RIK, H2-K1, H2-Q10, H2-M3, H2-DMB1, 2610524H06RIK, H2-AB1, H2-Q7, FCGR1, H2-DMB2, FCGR3, FCGR2B, H2-OA, H2-EB1, H2-AA, H2-T23, H2-DMA	2002	35	13588	5.24	1.49E-11	1.35E-12	6.82E-12
GOTERM_BP_FAT	GO:0002478~antigen processing and presentation of exogenous peptide antigen	21	0.74	2.39E-14	H2-K1, H2-M3, UNC93B1, H2-DMB1, 2610524H06RIK, IFI30, H2-AB1, FCGR1, H2-DMB2, CD74, FCGR3, TAPBP, B2M, H2-OA, FCGR2B, TAP2, H2-EB1, FCER1G, H2-AA, H2-DMA, 1500011B03RIK	2002	23	13588	6.20	9.74E-11	6.49E-12	4.46E-11
GOTERM_BP_FAT	GO:0019884~antigen processing and presentation of exogenous antigen	22	0.77	1.28E-12	H2-K1, H2-M3, UNC93B1, H2-DMB1, 2610524H06RIK, IFI30, H2-AB1, CD1D1, FCGR1, H2-DMB2, CD74, FCGR3, TAPBP, B2M, FCGR2B, H2-OA, TAP2, H2-EB1, FCER1G, H2-AA, H2-DMA, 1500011B03RIK	2002	28	13588	5.33	5.21E-09	2.37E-10	2.39E-09
GOTERM_BP_FAT	GO:0019882~antigen processing and presentation	37	1.30	1.36E-09	H2-D1, UNC93B1, IFI30, CD1D1, CALR, CD74, B2M, TAPBP, SLC11A1, TAP2, FCER1G, H2-T10, 1500011B03RIK, H2-K1, ICAM1, H2-Q10, H2-Q5, H2-M3, H2-DMB1, 2610524H06RIK, H2-AB1, H2-Q7, FCGR1, H2-DMB2, PSMB8, FCGR3, PSMB9, FCGR2B, H2-OA, C920025E04RIK, ULBP1, H2-EB1, H2-OB, H2-AA, H2-T23, H2-T24, H2-DMA	2002	87	13588	2.89	5.53E-06	1.54E-07	2.54E-06
GOTERM_BP_FAT	GO:0019886~antigen processing and presentation of exogenous peptide antigen via MHC class II	14	0.49	5.35E-09	UNC93B1, 2610524H06RIK, IFI30, H2-DMB1, H2-AB1, CD74, H2-DMB2, H2-OA, FCGR2B, H2-EB1, H2-AA, FCER1G, H2-DMA, 1500011B03RIK	2002	16	13588	5.94	2.17E-05	4.94E-07	9.96E-06
GOTERM_BP_FAT	GO:0002495~antigen processing and presentation of peptide antigen via MHC class II	14	0.49	5.35E-09	UNC93B1, 2610524H06RIK, IFI30, H2-DMB1, H2-AB1, CD74, H2-DMB2, H2-OA, FCGR2B, H2-EB1, H2-AA, FCER1G, H2-DMA, 1500011B03RIK	2002	16	13588	5.94	2.17E-05	4.94E-07	9.96E-06
GOTERM_BP_FAT	GO:0002504~antigen processing and presentation of peptide or polysaccharide antigen via MHC class I	15	0.53	1.21E-08	UNC93B1, 2610524H06RIK, IFI30, H2-DMB1, H2-AB1, CD74, H2-DMB2, H2-OA, FCGR2B, H2-EB1, H2-OB, FCER1G, H2-AA, H2-DMA, 1500011B03RIK	2002	19	13588	5.36	4.92E-05	1.07E-06	2.26E-05

Annotation Cluster 3 — Enrichment Score: 9.623234366337368

Category	Term	Count	%	PValue	Genes	List Total	Pop Hits	Pop Total	Fold Enrichment	Bonferroni	Benjamini	FDR
GOTERM_BP_FAT	GO:0001817~regulation of cytokine production	60	2.11	1.97E-15	ELF1, IL27RA, IL18, TLR1, TLR2, TLR3, TLR4, CD1D1, TLR6, TLR7, TLR8, TLR9, NOD2, MYD88, SH2D1B1, HMOX1, TICAM1, TICAM2, IL1B, LBP, CASP1, LTB, LAG3, SPN, SCAMP5, ICOSL, CD40, IFNAR1, CARD11, CD83, ULBP1, TNF, ADORA2B, SLC37A4, KLRK1, IRAK3, SLC11A1, PYCARD, BCL3, FCER1G, INPP5D, CD27, CD28, CLEC2I, FCER1A, IL6, CARD9, H2-M3, SPHK1, CD276, CENPF, H2-Q7, FCGR3, DDX58, P2RX7, IRF1, IRF4, CLEC7A, CD14, SASH3	2002	139	13588	2.93	8.11E-12	9.02E-13	3.72E-12
GOTERM_BP_FAT	GO:0042108~positive regulation of cytokine biosynthetic process	22	0.77	7.21E-08	FCER1A, ICOSL, TLR1, TLR3, TLR4, CD1D1, TLR6, TLR7, TLR8, TLR9, FCGR3, CARD11, MYD88, TICAM1, IRF1, IL1B, BCL3, IRF4, LTB, SPN, CD27, CD28	2002	42	13588	3.56	2.93E-04	5.14E-06	1.34E-04
GOTERM_BP_FAT	GO:0042035~regulation of cytokine biosynthetic process	28	0.99	9.51E-08	TLR1, TLR3, TLR4, TLR6, CD1D1, TLR7, TLR8, TLR9, MYD88, TICAM1, IL1B, BCL3, INPP5D, LTB, LAG3, SPN, CD27, CD28, CLEC2I, FCER1A, ICOSL, IL6, CARD9, CD276, FCGR3, CARD11, IRF1, IRF4	2002	64	13588	2.97	3.86E-04	6.55E-06	1.77E-04

Annotation Cluster 4 — Enrichment Score: 9.23522290730371

Category	Term	Count	%	PValue	Genes	List Total	Pop Hits	Pop Total	Fold Enrichment	Bonferroni	Benjamini	FDR
GOTERM_BP_FAT	GO:0001817~regulation of cytokine production	60	2.11	1.97E-15	ELF1, IL27RA, IL18, TLR1, TLR2, TLR3, TLR4, CD1D1, TLR6, TLR7, TLR8, TLR9, NOD2, MYD88, SH2D1B1, HMOX1, TICAM1, TICAM2, IL1B, LBP, CASP1, LTB, LAG3, SPN, SCAMP5, ICOSL, CD40, IFNAR1, CARD11, CD83, ULBP1, TNF, ADORA2B, SLC37A4, KLRK1, IRAK3, SLC11A1, PYCARD, BCL3, FCER1G, INPP5D, CD27, CD28, CLEC2I, FCER1A, IL6, CARD9, H2-M3, SPHK1, CD276, CENPF, H2-Q7, FCGR3, DDX58, P2RX7, IRF1, IRF4, CLEC7A, CD14, SASH3	2002	139	13588	2.93	8.11E-12	9.02E-13	3.72E-12
GOTERM_BP_FAT	GO:0001819~positive regulation of cytokine production	31	1.09	3.26E-10	TNF, ADORA2B, IL27RA, IL18, TLR2, KLRK1, TLR3, TLR4, TLR7, SLC11A1, NOD2, MYD88, TICAM1, PYCARD, IL1B, FCER1G, BCL3, LBP, CASP1, CARD9, H2-M3, CD40, H2-Q7, IFNAR1, DDX58, CD83, P2RX7, ULBP1, CLEC7A, CD14, SASH3	2002	62	13588	3.39	1.32E-06	4.13E-08	6.06E-07

Annotation Cluster 5 — Enrichment Score: 8.14169939983026

Category	Term	Count	%	PValue	Genes	List Total	Pop Hits	Pop Total	Fold Enrichment	Bonferroni	Benjamini	FDR
GOTERM_BP_FAT	GO:0002684~positive regulation of immune system process	87	3.06	1.98E-21	IL27RA, IL18, HPS1, TLR2, TNFSF14, PTPN22, TLR4, TNFSF13, CD1D1, C1QC, PNP, TGFB1, TLR8, B2M, CFP, NOD2, C1RB, MYD88, KLHL6, HLX, TICAM1, LBP, LAG3, SPN, H2-K1, ICOSL, LYN, C4B, CD40, NFAM1, THY1, C1QA, C1QB, CARD11, CD83, CD37, LAT2, CD80, TNFSF13B, ULBP1, LCK, H2-AA, ITGAL, ADORA2B, C3, IL4RA, UNC93B1, KLRK1, CACNB3, IL7R, CD74, ADA, IRAK3, SLC11A1, TAP2, FCER1G, CD4, IL2RG, SH2B2, INPP5D, THBS1, CD27, CLEC2I, CD28, FCER1A, PTPRC, PTPN6, SELP, IL6, CARD9, IKZF1, H2-M3, CFB, H2-Q7, FCGR1, FOXP1, FCGR3, BCL2A1D, CDKN1A, P2RX7, CORO1A, PLCG2, C1RL, CD79B, CLEC7A, H2-DMA, SASH3	2002	206	13588	2.87	8.05E-18	2.01E-18	3.69E-18
GOTERM_BP_FAT	GO:0048584~positive regulation of response to stimulus	72	2.53	2.35E-15	IL16, IL27RA, TLR2, PTPN22, TLR3, TLR4, TNFSF13, CD1D1, CXCL12, PNP, C1QC, TLR8, B2M, CFP, CD47, NOD2, C1RB, MYD88, KLHL6, TICAM1, IL1B, LBP, LAG3, H2-K1, LYN, C4B, NFAM1, THY1, C1QA, C1QB, LAT2, TNFSF13B, ULBP1, LCK, H2-AA, TNF, ADORA2B, C3, KLRK1, UNC93B1, CACNB3, ADA, SRC, SLC11A1, TNFRSF1A, TAP2, FCER1G, SH2B2, THBS1, CD27, CLEC2I, CD28, FCER1A, PTPRC, PTPN6, CARD9, H2-M3, CFB, EDA2R, AGER, H2-Q7, FCGR1, FCGR3, BCL2A1D, P2RX7, NPY, PLCG2, C1RL, CD79B, CLEC7A, H2-DMA, SASH3	2002	186	13588	2.63	9.47E-12	9.47E-13	4.34E-12
GOTERM_BP_FAT	GO:0050778~positive regulation of immune response	58	2.04	1.15E-14	IL27RA, TLR2, PTPN22, TNFSF13, TLR4, CD1D1, PNP, C1QC, TLR8, B2M, CFP, C1RB, NOD2, KLHL6, MYD88, TICAM1, LBP, LAG3, H2-K1, LYN, C4B, NFAM1, THY1, C1QA, C1QB, LAT2, TNFSF13B, ULBP1, LCK, H2-AA, C3, KLRK1, UNC93B1, CACNB3, ADA, SLC11A1, TAP2, FCER1G, SH2B2, CD28, CLEC2I, FCER1A, PTPN6, PTPRC, CARD9, H2-M3, CFB, H2-Q7, FCGR1, FCGR3, BCL2A1D, P2RX7, PLCG2, C1RL, CD79B, CLEC7A, H2-DMA, SASH3	2002	136	13588	2.89	4.69E-11	3.35E-12	2.15E-11
GOTERM_BP_FAT	GO:0002764~immune response-regulating signal transduction	26	0.91	1.15E-08	TLR2, KLRK1, UNC93B1, PTPN22, CACNB3, NOD2, KLHL6, MYD88, SH2D1B1, TICAM1, FCER1G, SH2B2, CLEC2I, PTPN6, PTPRC, LYN, CD40, NFAM1, THY1, BTLA, BCL2A1D, LAT2, LCK, PLCG2, CD79B, CLEC7A	2002	52	13588	3.39	4.67E-05	1.04E-06	2.14E-05
GOTERM_BP_FAT	GO:0002768~immune response-regulating cell surface receptor signaling pathway	22	0.77	2.00E-07	PTPN6, PTPRC, LYN, TLR2, KLRK1, PTPN22, CACNB3, NFAM1, CD40, THY1, BTLA, BCL2A1D, LAT2, KLHL6, SH2D1B1, LCK, PLCG2, FCER1G, CD79B, SH2B2, CLEC7A, CLEC2I	2002	44	13588	3.39	8.12E-04	1.23E-05	3.73E-04
GOTERM_BP_FAT	GO:0002253~activation of immune response	33	1.16	2.18E-07	C3, TLR2, KLRK1, UNC93B1, PTPN22, CACNB3, TLR4, C1QC, CFP, C1RB, NOD2, KLHL6, MYD88, TICAM1, FCER1G, SH2B2, CLEC2I, PTPN6, PTPRC, LYN, C4B, CFB, NFAM1, THY1, C1QA, C1QB, BCL2A1D, LAT2, LCK, PLCG2, C1RL, CD79B, CLEC7A	2002	86	13588	2.60	8.84E-04	1.32E-05	4.06E-04
GOTERM_BP_FAT	GO:0002757~immune response-activating signal transduction	23	0.81	2.52E-07	PTPN6, PTPRC, LYN, TLR2, UNC93B1, KLRK1, PTPN22, CACNB3, NFAM1, THY1, BCL2A1D, LAT2, NOD2, MYD88, KLHL6, TICAM1, LCK, PLCG2, FCER1G, CD79B, SH2B2, CLEC7A, CLEC2I	2002	48	13588	3.25	1.02E-03	1.48E-05	4.70E-04
GOTERM_BP_FAT	GO:0002429~immune response-activating cell surface receptor signaling pathway	19	0.67	6.64E-06	PTPN6, PTPRC, LYN, KLRK1, TLR2, PTPN22, CACNB3, NFAM1, THY1, BCL2A1D, LAT2, KLHL6, LCK, PLCG2, FCER1G, CD79B, SH2B2, CLEC7A, CLEC2I	2002	41	13588	3.15	2.66E-02	2.59E-04	1.24E-02

Annotation Cluster 6 — Enrichment Score: 7.711135357517727

Category	Term	Count	%	PValue	Genes	List Total	Pop Hits	Pop Total	Fold Enrichment	Bonferroni	Benjamini	FDR
GOTERM_BP_FAT	GO:0007049~cell cycle	154	5.42	5.17E-12	ADCY3, KIFC1, CEP72, S100A6, PRC1, DBF4, KNTC1, PKMYT1, AURKB, APP, CDCA8, CUL7, DDX11, OIP5, INCENP, MAP3K8, CDCA2, CCNA2, CDCA5, ASPM, CDCA3, CGRRF1, PDPN, SGOL2, LIG1, RBL1, SGOL1, 4632434I11RIK, SKP2, ESPL1, GPR132, PLK1S1, TACC3, MYH9, TACC1, ESCO2, UHRF1, MAD2L1, RCC2, TIMELESS, SPAG5, BIN3, MAPK7, STMN1, DSCC1, NEK2, CHEK1, ANLN, CCNG1, SPC24, SPC25, NCAPG2, OVOL1, FBXO5, LFNG, HELLS, ERCC6L, CKAP2, IKZF1, MKI67, AK1, NUF2, TPD52L1, NDC80, CDC20, GAS1, BRCA1, NOTCH2, CDKN1A, PLK3, PLK2, RGS2, PLK1, PARD6G, CHAF1A, CHAF1B, KCTD11, E2F1, GAS2L3, E2F2, CLSPN, E2F6, E2F7, E2F8, CUZD1, SLFN1, CDT1, CCNE1, CGREF1, MCM7, TUBB5, MTBP, SIK1, TUBB3, CDC7, CDK1, CDC6, ARHGEF2, KIF11, CCNF, NUSAP1, CDK6, 1700017B05RIK, HMGA2, MCM3, UBE2C, UBE2B, CDK2, RBBP8, ARL3, MCM6, RAD51, CCND1, FANCD2, NSL1, BUB1B, G0S2, HAUS8, CKS1B, PML, POLA1, MLF1, NCAPH, MDC1, HJURP, BUB1, ZWILCH, TRIP13, BOD1, DLGAP5, ANXA1, CENPF, CENPE, BIRC5, SIRT7, RACGAP1, CDKN3, SMC2, RGS14, GSG2, CDC25B, SMC4, CCNB1, RASSF4, CCNB2, GSPT2, RASSF2, CKS2, PHGDH, KIF20B, CHTF18, GAS2L1, CIT, CDK20	2002	611	13588	1.71	2.10E-08	8.75E-10	9.63E-09
GOTERM_BP_FAT	GO:0000278~mitotic cell cycle	73	2.57	1.95E-09	KIFC1, E2F6, DBF4, KNTC1, AURKB, SLFN1, APP, CDCA8, OIP5, INCENP, MTBP, CDCA2, CDCA5, CCNA2, TUBB3, ASPM, CDCA3, CDC6, CDK1, ARHGEF2, KIF11, SGOL1, CCNF, SKP2, NUSAP1, GPR132, HMGA2, UBE2C, CDK2, RBBP8, CCND1, MAD2L1, RCC2, TIMELESS, SPAG5, NSL1, BUB1B, STMN1, HAUS8, NEK2, POLA1, CHEK1, ANLN, CCNG1, SPC24, SPC25, NCAPH, NCAPG2, BUB1, FBXO5, ZWILCH, HELLS, ERCC6L, BOD1, DLGAP5, NUF2, CENPF, TPD52L1, CDC20, CENPE, NDC80, BIRC5, SIRT7, SMC2, RGS14, SMC4, CDC25B, CCNB1, CCNB2, PLK2, PLK1, KIF20B, CIT	2002	244	13588	2.03	7.91E-06	2.14E-07	3.63E-06
GOTERM_BP_FAT	GO:0022402~cell cycle process	103	3.62	2.67E-09	ADCY3, KIFC1, CEP72, DBF4, KNTC1, AURKB, CDCA8, APP, OIP5, INCENP, CDCA2, CDCA5, CCNA2, ASPM, CDCA3, CGRRF1, SGOL2, SGOL1, SKP2, 4632434I11RIK, GPR132, ESPL1, PLK1S1, MYH9, TACC3, MAD2L1, TIMELESS, RCC2, SPAG5, STMN1, NEK2, CHEK1, ANLN, CCNG1, SPC24, SPC25, NCAPG2, OVOL1, FBXO5, LFNG, HELLS, ERCC6L, MKI67, AK1, NUF2, NDC80, CDC20, TPD52L1, GAS1, BRCA1, NOTCH2, CDKN1A, PLK1, GAS2L3, E2F6, SLFN1, CGREF1, MTBP, TUBB5, TUBB3, CDC6, CDK1, ARHGEF2, KIF11, CCNF, NUSAP1, UBE2C, HMGA2, UBE2B, CDK2, RAD51, RBBP8, CCND1, FANCD2, NSL1, BUB1B, HAUS8, PML, POLA1, MLF1, NCAPH, BUB1, ZWILCH, TRIP13, BOD1, DLGAP5, CENPF, BIRC5, CENPE, SIRT7, RACGAP1, CDKN3, SMC2, RGS14, CDC25B, SMC4, CCNB1, CCNB2, CKS2, PHGDH, KIF20B, GAS2L1, CIT	2002	393	13588	1.78	1.08E-05	2.78E-07	4.96E-06
GOTERM_BP_FAT	GO:0051301~cell division	80	2.81	3.81E-09	KIFC1, PRC1, KNTC1, CUZD1, AURKB, CCNE1, CDCA8, CDCA7, OIP5, INCENP, CDCA2, CDCA5, CCNA2, TOP2A, ASPM, CDCA3, CDC7, CDC6, CDK1, ARHGEF2, KIF11, SGOL2, LIG1, SGOL1, CCNF, NUSAP1, CDK6, 1700017B05RIK, MYH9, HMGA2, UBE2C, TACC1, MCM5, CDK2, ARL3, CCND1, MAD2L1, TIMELESS, RCC2, SPAG5, NSL1, CFL1, BUB1B, BIN3, HAUS8, CKS1B, RBP4, NEK2, ANLN, CCNG1, SPC24, SPC25, NCAPH, NCAPG2, BUB1, PSTPIP1, FBXO5, ZWILCH, HELLS, ERCC6L, BOD1, NUF2, BIRC5, NDC80, CDC20, CENPE, DPPA3, RACGAP1, SMC2, SMC4, CDC25B, CCNB1, HOXB4, CCNB2, PLK1, KIF20B, CKS2, PARD6G, CIT, CDK20	2002	281	13588	1.93	1.55E-05	3.68E-07	7.09E-06
GOTERM_BP_FAT	GO:0000087~M phase of mitotic cell cycle	59	2.08	4.31E-08	KIFC1, KNTC1, AURKB, CDCA8, OIP5, INCENP, CDCA2, CDCA5, CCNA2, ASPM, TUBB3, CDCA3, CDC6, CDK1, ARHGEF2, KIF11, SGOL1, CCNF, NUSAP1, UBE2C, HMGA2, CDK2, MAD2L1, RCC2, TIMELESS, SPAG5, NSL1, BUB1B, HAUS8, NEK2, ANLN, CCNG1, SPC24, SPC25, NCAPH, NCAPG2, BUB1, FBXO5, ZWILCH, HELLS, ERCC6L, BOD1, DLGAP5, NUF2, CENPF, CDC20, CENPE, NDC80, BIRC5, SIRT7, SMC2, RGS14, CDC25B, SMC4, CCNB1, CCNB2, PLK1, KIF20B, CIT	2002	194	13588	2.06	1.75E-04	3.50E-06	8.02E-05

GOTERM_BP_FAT	GO:0022403~cell cycle phase	86	3.03	6.15E-08	ADCY3, KIFC1, E2F6, DBF4, KNTC1, AURKB, SLFN1, APP, CDCA8, OIP5, INCENP, MTBP, TUBB5, CDCA2, CDCA5, CCNA2, TUBB3, ASPM, CDCA3, CDC6, CDK1, ARHGEF2, KIF11, SGOL2, SGOL1, CCNF, SKP2, NUSAP1, ESPL1, GPR132, MYH9, HMGA2, TACC3, UBE2C, UBE2B, CDK2, RAD51, RBBP8, CCND1, MAD2L1, TIMELESS, RCC2, SPAG5, FANCD2, NSL1, BUB1B, STMN1, HAUS8, NEK2, POLA1, CHEK1, ANLN, CCNG1, SPC24, SPC25, NCAPH, NCAPG2, OVOL1, BUB1, FBXO5, ZWILCH, LFNG, HELLS, TRIP13, ERCC6L, BOD1, MKI67, DLGAP5, NUF2, CENPF, TPD52L1, BIRC5, NDC80, CDC20, CENPE, SIRT7, SMC2, RGS14, SMC4, CDC25B, CCNB1, CCNB2, PLK1, KIF20B, CKS2, CIT	2002	328	13588	1.78	2.50E-04	4.46E-06	1.14E-04
GOTERM_BP_FAT	GO:0000280~nuclear division	56	1.97	3.11E-07	KIFC1, KNTC1, AURKB, CDCA8, OIP5, INCENP, CDCA2, CDCA5, CCNA2, ASPM, TUBB3, CDCA3, CDC6, CDK1, ARHGEF2, KIF11, SGOL1, CCNF, NUSAP1, UBE2C, HMGA2, CDK2, MAD2L1, RCC2, TIMELESS, SPAG5, NSL1, BUB1B, HAUS8, NEK2, ANLN, CCNG1, SPC24, SPC25, NCAPH, NCAPG2, BUB1, FBXO5, ZWILCH, HELLS, ERCC6L, BOD1, NUF2, CDC20, CENPE, NDC80, BIRC5, SMC2, RGS14, CDC25B, SMC4, CCNB1, CCNB2, PLK1, KIF20B, CIT	2002	190	13588	2.00	1.26E-03	1.75E-05	5.78E-04
GOTERM_BP_FAT	GO:0007067~mitosis	56	1.97	3.11E-07	KIFC1, KNTC1, AURKB, CDCA8, OIP5, INCENP, CDCA2, CDCA5, CCNA2, ASPM, TUBB3, CDCA3, CDC6, CDK1, ARHGEF2, KIF11, SGOL1, CCNF, NUSAP1, UBE2C, HMGA2, CDK2, MAD2L1, RCC2, TIMELESS, SPAG5, NSL1, BUB1B, HAUS8, NEK2, ANLN, CCNG1, SPC24, SPC25, NCAPH, NCAPG2, BUB1, FBXO5, ZWILCH, HELLS, ERCC6L, BOD1, NUF2, CDC20, CENPE, NDC80, BIRC5, SMC2, RGS14, CDC25B, SMC4, CCNB1, CCNB2, PLK1, KIF20B, CIT	2002	190	13588	2.00	1.26E-03	1.75E-05	5.78E-04
GOTERM_BP_FAT	GO:0048285~organelle fission	57	2.01	4.69E-07	KIFC1, KNTC1, AURKB, CDCA8, OIP5, INCENP, CDCA2, CDCA5, CCNA2, ASPM, TUBB3, CDCA3, CDC6, CDK1, ARHGEF2, KIF11, SGOL1, CCNF, NUSAP1, UBE2C, HMGA2, CDK2, MAD2L1, RCC2, TIMELESS, SPAG5, NSL1, BUB1B, HAUS8, NEK2, ANLN, CCNG1, SPC24, SPC25, NCAPH, NCAPG2, BUB1, FBXO5, ZWILCH, HELLS, ERCC6L, BOD1, NUF2, CDC20, CENPE, NDC80, BIRC5, SMC2, RGS14, CDC25B, SMC4, CCNB1, PEX11A, CCNB2, PLK1, KIF20B, CIT	2002	197	13588	1.96	1.90E-03	2.47E-05	8.73E-04
GOTERM_BP_FAT	GO:0000279~M phase	74	2.60	6.31E-07	ADCY3, KIFC1, KNTC1, AURKB, CDCA8, OIP5, INCENP, TUBB5, CDCA2, CDCA5, CCNA2, TUBB3, ASPM, CDCA3, CDC6, CDK1, ARHGEF2, KIF11, SGOL2, SGOL1, CCNF, NUSAP1, ESPL1, MYH9, HMGA2, TACC3, UBE2C, UBE2B, CDK2, RAD51, MAD2L1, RCC2, TIMELESS, SPAG5, FANCD2, NSL1, BUB1B, STMN1, HAUS8, NEK2, ANLN, CCNG1, SPC24, SPC25, NCAPH, NCAPG2, OVOL1, BUB1, FBXO5, ZWILCH, LFNG, HELLS, TRIP13, ERCC6L, BOD1, MKI67, DLGAP5, NUF2, CENPF, CDC20, CENPE, NDC80, BIRC5, SIRT7, SMC2, RGS14, CDC25B, SMC4, CCNB1, CCNB2, PLK1, KIF20B, CKS2, CIT	2002	283	13588	1.77	2.56E-03	3.05E-05	1.17E-03

Annotation Cluster 7 Enrichment Score: 7.056293825949427

Category	Term	Count	%	PValue	Genes	List Total	Pop Hits	Pop Total	Fold Enrichment	Bonferroni	Benjamini	FDR
GOTERM_BP_FAT	GO:0001775~cell activation	86	3.03	4.01E-15	ELF4, TLR1, RORC, PTPN22, TLR4, NFKB2, CD1D1, SKAP2, CXCL12, TGFB1, CD48, NLRC3, CLCF1, CXCR4, TICAM1, LBP, LTB, SPN, RHOH, RAB27A, EGR1, GAPT, ICOSL, NFAM1, CD40, MYH9, WAS, PRKCD, IFNAR1, DCLRE1C, CARD11, CD86, LAT2, UNC13D, LILRB3, LCK, CX3CR1, LCP1, LCP2, ITGAL, SBNO2, LST1, TNF, KLRK1, ITGB2, PF4, SFPI1, IL7R, FKBP1B, CD74, ADA, ITGAM, SLC11A1, DOCK2, ITGAX, PPP3CB, FCER1G, BCL3, CD4, ENTPD1, HELLS, CD27, BLNK, CD28, FYB, PTPRC, IKZF1, PLEK, H2-M3, SWAP70, FLT3, MYO1F, VAV1, FOXP1, FCGR3, P2RY12, LAT, BCL2A1D, P2RX7, FCGR2B, FYN, PLCG2, IRF1, IRF4, BANK1, H2-DMA	2002	246	13588	2.37	1.62E-11	1.35E-12	7.44E-12
GOTERM_BP_FAT	GO:0045321~leukocyte activation	78	2.74	2.79E-14	ELF4, TLR1, PTPN22, RORC, TLR4, CD1D1, SKAP2, CXCL12, TGFB1, CD48, NLRC3, CLCF1, CXCR4, TICAM1, LBP, SPN, RHOH, RAB27A, EGR1, GAPT, ICOSL, NFAM1, CD40, MYH9, WAS, PRKCD, IFNAR1, DCLRE1C, CARD11, CD86, LAT2, UNC13D, LILRB3, LCK, CX3CR1, LCP1, LCP2, ITGAL, SBNO2, KLRK1, ITGB2, SFPI1, IL7R, FKBP1B, CD74, ADA, ITGAM, SLC11A1, DOCK2, ITGAX, PPP3CB, BCL3, FCER1G, CD4, CD27, HELLS, BLNK, CD28, FYB, PTPRC, IKZF1, H2-M3, SWAP70, FLT3, MYO1F, VAV1, FOXP1, FCGR3, LAT, BCL2A1D, P2RX7, FCGR2B, FYN, PLCG2, IRF1, IRF4, BANK1, H2-DMA	2002	219	13588	2.42	1.13E-10	7.07E-12	5.19E-11
GOTERM_BP_FAT	GO:0046649~lymphocyte activation	63	2.22	4.06E-10	ELF4, PTPN22, RORC, CD1D1, SKAP2, CXCL12, TGFB1, CD48, NLRC3, CXCR4, CLCF1, SPN, RHOH, RAB27A, EGR1, GAPT, ICOSL, NFAM1, CD40, MYH9, WAS, PRKCD, IFNAR1, DCLRE1C, CARD11, LAT2, CD86, UNC13D, LCK, LCP1, ITGAL, KLRK1, SFPI1, ITGB2, IL7R, FKBP1B, CD74, ITGAM, ADA, SLC11A1, DOCK2, ITGAX, PPP3CB, BCL3, CD4, CD27, HELLS, BLNK, CD28, PTPRC, IKZF1, H2-M3, SWAP70, FLT3, VAV1, FOXP1, BCL2A1D, P2RX7, FYN, PLCG2, IRF1, BANK1, H2-DMA	2002	191	13588	2.24	1.65E-06	4.99E-08	7.56E-07
GOTERM_BP_FAT	GO:0042110~T cell activation	44	1.55	2.17E-09	ITGAL, ELF4, PTPN22, RORC, ITGB2, CD1D1, IL7R, CXCL12, FKBP1B, CD74, ITGAM, TGFB1, CD48, SLC11A1, DOCK2, NLRC3, ITGAX, CXCR4, PPP3CB, BCL3, CD4, SPN, CD28, RHOH, RAB27A, EGR1, PTPRC, ICOSL, H2-M3, IKZF1, FLT3, MYH9, WAS, VAV1, IFNAR1, CARD11, BCL2A1D, CD86, P2RX7, FYN, LCK, IRF1, H2-DMA, LCP1	2002	116	13588	2.57	8.80E-06	2.32E-07	4.03E-06
GOTERM_BP_FAT	GO:0048534~hemopoietic or lymphoid organ development	74	2.60	4.71E-07	GMPR2, G6PDX, PTPN22, RORC, NFKB2, CD1D1, TGFB1, ANK1, ALAS2, TNFRSF11A, CLCF1, GAB3, CASP8, HBB-B1, LTB, SPN, MB, RHOH, EGR1, SGPL1, STAP1, LYN, CDK6, NFAM1, MYH9, TACC3, DNASE2A, BCL2L11, DCLRE1C, CARD11, HIF1A, LILRB3, CCR2, LCK, CD300LF, TNF, CCR1, SLC37A4, PML, TTC7, SFXN1, SFPI1, IL7R, CD74, ADA, MLF1, DOCK2, PPP3CB, BCL3, CD4, RUNX1, HELLS, CD28, CEBPA, PTPRC, IKZF1, FLT3, HCLS1, BPGM, VAV1, FOXP1, HBA-A1, HOXB4, PLSCR1, BCL2A1D, CCNB2, ID2, CXCL13, PLCG2, IRF8, IRF1, IRF4, JAK3, H2-DMA	2002	281	13588	1.79	1.91E-03	2.45E-05	8.76E-04
GOTERM_BP_FAT	GO:0002520~immune system development	76	2.67	8.16E-07	GMPR2, G6PDX, RORC, PTPN22, NFKB2, CD1D1, TGFB1, ANK1, ALAS2, TNFRSF11A, CLCF1, GAB3, CASP8, HBB-B1, LTB, SPN, MB, RHOH, EGR1, SGPL1, ICOSL, STAP1, LYN, CDK6, NFAM1, MYH9, TACC3, DNASE2A, BCL2L11, DCLRE1C, CARD11, HIF1A, LILRB3, CCR2, LCK, CD300LF, TNF, CCR1, SLC37A4, PML, TTC7, SFXN1, SFPI1, IL7R, CD74, ADA, MLF1, DOCK2, PPP3CB, BCL3, CD4, RUNX1, HELLS, CD28, CEBPA, PTPRC, IKZF1, SWAP70, FLT3, HCLS1, BPGM, VAV1, FOXP1, HBA-A1, HOXB4, PLSCR1, BCL2A1D, CCNB2, ID2, CXCL13, PLCG2, IRF8, IRF1, IRF4, JAK3, H2-DMA	2002	295	13588	1.75	3.31E-03	3.81E-05	1.52E-03
GOTERM_BP_FAT	GO:0030097~hemopoiesis	67	2.36	1.04E-06	GMPR2, G6PDX, PTPN22, RORC, CD1D1, TGFB1, ANK1, ALAS2, CLCF1, GAB3, CASP8, HBB-B1, SPN, MB, RHOH, EGR1, SGPL1, STAP1, LYN, CDK6, NFAM1, MYH9, TACC3, DNASE2A, DCLRE1C, CARD11, HIF1A, LILRB3, LCK, CCR2, CD300LF, TNF, CCR1, SLC37A4, PML, SFXN1, SFPI1, TTC7, IL7R, CD74, ADA, MLF1, DOCK2, PPP3CB, BCL3, CD4, RUNX1, HELLS, CD28, CEBPA, PTPRC, IKZF1, FLT3, HCLS1, BPGM, VAV1, FOXP1, HBA-A1, HOXB4, PLSCR1, BCL2A1D, ID2, PLCG2, IRF8, IRF1, IRF4, H2-DMA	2002	251	13588	1.81	4.23E-03	4.81E-05	1.94E-03

Annotation Cluster 8 Enrichment Score: 7.047990221245499

Category	Term	Count	%	PValue	Genes	List Total	Pop Hits	Pop Total	Fold Enrichment	Bonferroni	Benjamini	FDR
GOTERM_BP_FAT	GO:0002252~immune effector process	51	1.79	5.83E-12	BNIP3, TNFSF13, C1QC, TLR7, TLR8, CFP, CD47, C1RB, NOD2, MYD88, TICAM1, LBP, RAB27A, ICAM1, ICOSL, NCF1, C4B, PRKCD, C1QA, C1QB, LAT2, UNC13D, TNFSF13B, LILRB3, H2-AA, C3, RSAD2, IL7R, CD74, SLC11A1, BCL3, FCER1G, INPP5D, CD27, PTPN6, PTPRC, CARD9, SWAP70, CFB, SAMHD1, MYO1F, FCGR1, H2-Q7, FOXP1, FCGR3, LAT, FCGR2B, KCNJ8, IRF7, C1RL, H2-DMA	2002	126	13588	2.75	2.37E-08	9.46E-10	1.08E-08
GOTERM_BP_FAT	GO:0002460~adaptive immune response based on somatic recombination of immune receptors built fr	34	1.20	3.05E-08	C3, IL18, TNFSF13, NFKB2, TLR6, C1QC, CD74, SLC11A1, NOD2, C1RB, KLHL6, MYD88, FCER1G, BCL3, INPP5D, RAB27A, ICAM1, ICOSL, C4B, CFB, SWAP70, PRKCD, FCGR1, FCGR3, C1QA, C1QB, IL18BP, UNC13D, FCGR2B, LILRB3, IRF7, C1RL, H2-AA, H2-DMA	2002	84	13588	2.75	1.24E-04	2.53E-06	5.68E-05
GOTERM_BP_FAT	GO:0002250~adaptive immune response	34	1.20	3.05E-08	C3, IL18, TNFSF13, NFKB2, TLR6, C1QC, CD74, SLC11A1, NOD2, C1RB, KLHL6, MYD88, FCER1G, BCL3, INPP5D, RAB27A, ICAM1, ICOSL, C4B, CFB, SWAP70, PRKCD, FCGR1, FCGR3, C1QA, C1QB, IL18BP, UNC13D, FCGR2B, LILRB3, IRF7, C1RL, H2-AA, H2-DMA	2002	84	13588	2.75	1.24E-04	2.53E-06	5.68E-05
GOTERM_BP_FAT	GO:0002443~leukocyte mediated immunity	34	1.20	1.56E-07	C3, TNFSF13, C1QC, CD74, SLC11A1, NOD2, C1RB, MYD88, FCER1G, BCL3, INPP5D, RAB27A, PTPN6, ICAM1, ICOSL, C4B, CFB, SWAP70, NCF1, MYO1F, PRKCD, FCGR1, FCGR3, C1QA, C1QB, LAT, LAT2, UNC13D, FCGR2B, LILRB3, IRF7, C1RL, H2-AA, H2-DMA	2002	89	13588	2.59	6.32E-04	9.89E-06	2.90E-04
GOTERM_BP_FAT	GO:0002449~lymphocyte mediated immunity	30	1.06	4.30E-07	C3, TNFSF13, C1QC, CD74, SLC11A1, C1RB, NOD2, MYD88, FCER1G, BCL3, INPP5D, RAB27A, PTPN6, ICAM1, ICOSL, C4B, SWAP70, CFB, PRKCD, FCGR1, FCGR3, C1QA, C1QB, UNC13D, FCGR2B, LILRB3, IRF7, C1RL, H2-AA, H2-DMA	2002	76	13588	2.68	1.75E-03	2.33E-05	8.01E-04
GOTERM_BP_FAT	GO:0019724~B cell mediated immunity	25	0.88	8.16E-06	C3, TNFSF13, C1QC, CD74, C1RB, NOD2, MYD88, FCER1G, BCL3, INPP5D, ICOSL, SWAP70, CFB, C4B, PRKCD, FCGR1, FCGR3, C1QA, C1QB, FCGR2B, IRF7, LILRB3, C1RL, H2-AA, H2-DMA	2002	65	13588	2.61	3.26E-02	3.07E-04	1.52E-02
GOTERM_BP_FAT	GO:0016064~immunoglobulin mediated immune response	24	0.84	1.56E-05	ICOSL, C3, C4B, CFB, SWAP70, TNFSF13, C1QC, PRKCD, FCGR1, CD74, FCGR3, C1QA, C1QB, NOD2, C1RB, MYD88, FCGR2B, IRF7, C1RL, FCER1G, BCL3, H2-AA, INPP5D, H2-DMA	2002	63	13588	2.59	6.12E-02	5.59E-04	2.89E-02

Annotation Cluster 9 Enrichment Score: 6.858086090880854

Category	Term	Count	%	PValue	Genes	List Total	Pop Hits	Pop Total	Fold Enrichment	Bonferroni	Benjamini	FDR
GOTERM_MF_FAT	GO:0032555~purine ribonucleotide binding	349	12.28	2.89E-10	ADCY3, GM6682, KIFC1, ADCY7, TUBB2B, TUBB2A, AURKB, PRKG2, BTK, ACTG1, DDX11, RAB28, OASL2, OASL1, MAP3K8, MAP2K6, RAB27A, GBP8, PIK3CG, TYRO3, GBP5, SUCLG1, LIG1, MYH3, MYLK4, KIF5C, GBP9, MYLK2, IFI47, MYH7, WNK2, GEM, MYH9, RFC5, NME4, NME5, NAV1, RAB19, ACAP1, NEK8, PDGFRA, MAPK7, EIF2AK2, 5430435G22RIK, CHKA, GNAI3, GNAI2, NEK2, PFKFB1, OAS3, UBA7, CHEK1, ARF6, MYO9B, OAS2, NAGK, EPHB3, EPHB2, VRK1, MOV10, RAC2, RAC3, POLQ, DHX58, SPHK1, TAOK3, UBE2L6, ABCG3, DDX4, RAD54L, ATP13A5, EPHA2, ABCG1, EPHA3, EPHA7, KCNJ8, ARF3, ARF4, GRK4, RAD54B, GM4951, CLCN5, NUAK2, MYO7A, ATP10A, CAMKK1, CKB, CAMKK2, ACTR3, ACTR2, NOD2, TUBB5, TUBB6, MASTL, DOCK10, ARL5C, SIK1, TOP2A, TARSL2, DOCK11, TUBB3, CDC7, RAP2B, CDC6, RAP2A, LYN, PHKG1, RAB4B, NDUFA13, PKDCC, 1700017B05RIK, BBS12, RAD51, MAST3, GM12250, PAPD4, ADK, RIPK1, EIF4A1, ARL4C, RASD2, ARL4A, MYO5A, RAB3A, ALDH18A1, RAB3D, CETN4, HK1, NLRP1A, ITM2C, GCH1, IGSF10, GALK2, GALK1, RAB44, RAB43, IGTP, PTK2B, CKMT2, HK3, BUB1, CERK, PAPSS1, PAPSS2, KIF21B, TRIP13, ACSL5, MYO5C, IRGM1, ACTC1, SWAP70, PDK3, ATP1A3, AXL, ATP1A4, ACLY, PCK2, RPS6KA6, RAB32, GM6548, RAB31, RPS6KA4, RPS6KA1, FYN, RAB34, KIF20B, ABCC3, CHTF18, RAB38, GYK, CIT, DNM1, KIF20A, RNASEL, GNA15, PSTK, F13A1, PASK, RRAD, TTK, PKMYT1, PI4K2B, MTHFD1L, PRKAR2B, ARL11, NLRC4, NLRC3, RALB, MLKL, SAR1B, MX1, MX2, MATK, CIITA, EGFR, PDXK, MAP4K4, H2-OB, RIPK3, OAS1B, OAS1C, OAS1A, LRRK1, GBP3, OAS1G, PRPS2, GBP2, 9930111J21RIK1, IFIH1, FGFR4, ABCA9, FGR, ASS1, ACTR3B, STK10, PTK7, MAP4K1, CTPS2, TIMP4, ABCA1, RRAGD, SRC, CMPK2, IRAK4, IRAK3, IIGP1, RUNX1, ENTPD1, RUNX2, HELLS, RUNX3, CSF1R, ERCC6L, TEC, EEF1A1, RAB8B, AK1, MYO1G, BRIP1, ATAD2, MYO1F, DOCK7, ATAD5, DOCK8, PSMB8, PSMB9, DDX58, IKBKE, P2RX4, DNA2, PLK4, P2RX7, P2RX6, PLK3, PLK2, PLK1, RAP1B, MERTK, PIP4K2A, KIF23, KIF22, KIF24, ATL2, FIGNL1, SLFN9, ATL3, PPIP5K2, SLFN8, SLFN5, FES, SLFN3, SLFN4, ACSF2, SLFN1, SLFN2, KIF2C, TOR3A, MCM7, ATP8B1, RHOB, ATP8B2, MKKS, RHOD, CSK, TUBA1A, SUCLA2, CDK15, TUBA1B, TUBA1C, RHOG, CDK14, RHOH, ATP8B4, KIF14, CDK1, KIF11, PFKL, LIMK1, KIF15, PFKP, CDK6, PFKM, PBK, MCM3, UBE2C, PRKCD, MCM4, UBE2B, CDK2, MCM5, ARL3, MCM6, PRKCB, DAPK1, TOR2A, ATP6V1A, LCK, H2-AA, BUB1B, NKIRAS1, MELK, CAMK1D, FRK, DCK, ASNS, GM5431, KIF9, NAIP6, NAIP2, TAP2, KIF4, TAP1, DCLK1, CHD3, ACTB, GIMAP3, ALPK1, FLT3, HCK, KIF18B, CENPE, SMC2, GSG2, SMC4, GCK, GSPT2, TEP1, APAF1, JAK3, H2-DMA, CDK20	1916	1796	13288	1.35	3.83E-07	1.28E-07	4.73E-07

GOTERM_MF_FAT	GO:0032553~ribonucleotide binding	349	12.28	2.89E-10	ADCY3, GM6682, KIFC1, ADCY7, TUBB2B, TUBB2A, AURKB, PRKG2, BTK, ACTG1, DDX11, RAB28, OASL2, OASL1, MAP3K8, MAP2K6, RAB27A, GBP8, PIK3CG, TYRO3, GBP5, SUCLG1, LIG1, MYH3, MYLK4, KIF5C, GBP9, MYLK2, IFI47, MYH7, WNK2, GEM, MYH9, RFC5, NME4, NME5, NAV1, RAB19, ACAP1, NEK8, PDGFRA, MAPK7, EIF2AK2, 5430435G22RIK, CHKA, GNAI3, GNAI2, NEK2, PFKFB1, OAS3, UBA7, CHEK1, ARF6, MYO9B, OAS2, NAGK, EPHB3, EPHB2, VRK1, MOV10, RAC2, RAC3, POLQ, DHX58, SPHK1, TAOK3, UBE2L6, ABCG3, DDX4, RAD54L, ATP13A5, EPHA2, ABCG1, EPHA3, EPHA7, KCNJ8, ARF3, ARF4, GRK4, RAD54B, GM4951, CLCN5, NUAK2, MYO7A, ATP10A, CAMKK1, CKB, CAMKK2, ACTR3, ACTR2, NOD2, TUBB5, TUBB6, MASTL, DOCK10, ARL5C, SIK1, TOP2A, TARSL2, DOCK11, TUBB3, CDC7, RAP2B, CDC6, RAP2A, LYN, PHKG1, RAB4B, NDUFA13, PKDCC, 1700017B05RIK, BBS12, RAD51, MAST3, GM12250, PAPD4, ADK, RIPK1, EIF4A1, ARL4C, RASD2, ARL4A, MYO5A, RAB3A, ALDH18A1, RAB3D, CETN4, HK1, NLRP1A, ITM2C, GCH1, IGSF10, GALK2, GALK1, RAB44, RAB43, IGTP, PTK2B, CKMT2, HK3, BUB1, CERK, PAPSS1, PAPSS2, KIF21B, TRIP13, ACSL5, MYO5C, IRGM1, ACTC1, SWAP70, PDK3, ATP1A3, AXL, ATP1A4, ACLY, PCK2, RPS6KA6, RAB32, GM6548, RAB31, RPS6KA4, RPS6KA1, FYN, RAB34, KIF20B, ABCC3, CHTF18, RAB38, GYK, CIT, DNM1, KIF20A, RNASEL, GNA15, PSTK, F13A1, PASK, RRAD, TTK, PKMYT1, PI4K2B, MTHFD1L, PRKAR2B, ARL11, NLRC4, NLRC3, RALB, MLKL, SAR1B, MX1, MX2, MATK, CIITA, EGFR, PDXK, MAP4K4, H2-OB, RIPK3, OAS1B, OAS1C, OAS1A, LRRK1, GBP3, OAS1G, PRPS2, GBP2, 9930111J21RIK1, IFIH1, FGFR4, ABCA9, FGR, ASS1, ACTR3B, STK10, PTK7, MAP4K1, CTPS2, TIMP4, ABCA1, RRAGD, SRC, CMPK2, IRAK4, IRAK3, IIGP1, RUNX1, ENTPD1, RUNX2, HELLS, RUNX3, CSF1R, ERCC6L, TEC, EEF1A1, RAB8B, AK1, MYO1G, BRIP1, ATAD2, MYO1F, DOCK7, ATAD5, DOCK8, PSMB8, PSMB9, DDX58, IKBKE, P2RX4, DNA2, PLK4, P2RX7, P2RX6, PLK3, PLK2, PLK1, RAP1B, MERTK, PIP4K2A, KIF23, KIF22, KIF24, ATL2, FIGNL1, SLFN9, ATL3, PPIP5K2, SLFN8, SLFN5, FES, SLFN3, SLFN4, ACSF2, SLFN1, SLFN2, KIF2C, TOR3A, MCM7, ATP8B1, RHOB, ATP8B2, MKKS, RHOD, CSK, TUBA1A, SUCLA2, CDK15, TUBA1B, TUBA1C, RHOG, CDK14, RHOH, ATP8B4, KIF14, CDK1, KIF11, PFKL, LIMK1, KIF15, PFKP, CDK6, PFKM, PBK, MCM3, UBE2C, PRKCD, MCM4, UBE2B, CDK2, MCM5, ARL3, MCM6, PRKCB, DAPK1, TOR2A, ATP6V1A, LCK, H2-AA, BUB1B, NKIRAS1, MELK, CAMK1D, FRK, DCK, ASNS, GM5431, KIF9, NAIP6, NAIP2, TAP2, KIF4, TAP1, DCLK1, CHD3, ACTB, GIMAP3, ALPK1, FLT3, HCK, KIF18B, CENPE, SMC2, GSG2, SMC4, GCK, GSPT2, TEP1, APAF1, JAK3, H2-DMA, CDK20	1916	1796	13288	1.35	3.83E-07	1.28E-07	4.73E-07
GOTERM_MF_FAT	GO:0017076~purine nucleotide binding	358	12.60	1.12E-09	ADCY3, GM6682, KIFC1, ADCY7, TUBB2B, TUBB2A, AURKB, PRKG2, BTK, ACTG1, DDX11, RAB28, OASL2, OASL1, MAP3K8, MAP2K6, RAB27A, GBP8, PIK3CG, TYRO3, GBP5, SUCLG1, LIG1, MYH3, MYLK4, KIF5C, GBP9, MYLK2, IFI47, MYH7, WNK2, GEM, MYH9, RFC5, NME4, NME5, NAV1, RAB19, ACAP1, DLD, NEK8, PDGFRA, MAPK7, EIF2AK2, 5430435G22RIK, CHKA, GNAI3, GNAI2, NEK2, PFKFB1, OAS3, UBA7, CHEK1, ARF6, MYO9B, OAS2, NAGK, EPHB3, EPHB2, VRK1, MOV10, RAC2, RAC3, POLQ, DHX58, SPHK1, FOXRED2, TAOK3, UBE2L6, ABCG3, DDX4, RAD54L, ATP13A5, EPHA2, ABCG1, EPHA3, EPHA7, KCNJ8, ARF3, ARF4, GRK4, RAD54B, GM4951, CLCN5, NUAK2, MYO7A, ATP10A, CAMKK1, CKB, CAMKK2, ACTR3, ACTR2, NOD2, TUBB5, TUBB6, MASTL, DOCK10, ARL5C, SIK1, TOP2A, TARSL2, DOCK11, TUBB3, CDC7, RAP2B, CDC6, RAP2A, LYN, PHKG1, RAB4B, NDUFA13, PKDCC, 1700017B05RIK, BBS12, RAD51, MAST3, GM12250, PAPD4, SQLE, ADK, RIPK1, EIF4A1, STEAP2, STEAP1, ARL4C, RASD2, ARL4A, MYO5A, RAB3A, ALDH18A1, RAB3D, CETN4, HK1, NLRP1A, ITM2C, GCH1, IGSF10, GALK2, GALK1, RAB44, RAB43, IGTP, PTK2B, CKMT2, HK3, BUB1, CERK, PAPSS1, PAPSS2, KIF21B, TRIP13, ACSL5, MYO5C, IRGM1, ACTC1, NOS1, SWAP70, PDK3, ATP1A3, AXL, ATP1A4, ACLY, PCK2, RPS6KA6, RAB32, GM6548, RAB31, RPS6KA4, RPS6KA1, FYN, RAB34, KIF20B, ABCC3, CHTF18, RAB38, GYK, CIT, DNM1, KIF20A, RNASEL, GNA15, PSTK, F13A1, PASK, RRAD, TTK, PKMYT1, PI4K2B, MTHFD1L, PRKAR2B, ARL11, NLRC4, NLRC3, RALB, MLKL, SAR1B, MX1, MX2, MATK, CIITA, EGFR, PDXK, MAP4K4, H2-OB, RIPK3, OAS1B, OAS1C, OAS1A, LRRK1, GBP3, OAS1G, PRPS2, GBP2, 9930111J21RIK1, XDH, IFIH1, FGFR4, ABCA9, ASS1, FGR, ACTR3B, STK10, PTK7, MAP4K1, CTPS2, TIMP4, ABCA1, RRAGD, SRC, CMPK2, IRAK4, IRAK3, IIGP1, RUNX1, ENTPD1, RUNX2, HELLS, RUNX3, CSF1R, ERCC6L, TEC, EEF1A1, RAB8B, AK1, MYO1G, BRIP1, ATAD2, MYO1F, DOCK7, ATAD5, DOCK8, PSMB8, PSMB9, DDX58, IKBKE, P2RX4, DNA2, PLK4, P2RX7, P2RX6, PLK3, PLK2, PLK1, RAP1B, MERTK, PIP4K2A, KIF23, KIF22, KIF24, ATL2, FIGNL1, SLFN9, ATL3, PPIP5K2, SLFN8, SLFN5, FES, SLFN3, SLFN4, ACSF2, SLFN1, SLFN2, KIF2C, TOR3A, MCM7, ATP8B1, RHOB, ATP8B2, MKKS, RHOD, CSK, TUBA1A, SUCLA2, CDK15, TUBA1B, TUBA1C, RHOG, CDK14, RHOH, ATP8B4, KIF14, CDK1, ACADM, KIF11, PFKL, LIMK1, KIF15, PFKP, CDK6, PFKM, PBK, MCM3, UBE2C, PRKCD, MCM4, UBE2B, CDK2, MCM5, ARL3, MCM6, PRKCB, DAPK1, TOR2A, ATP6V1A, LCK, H2-AA, BUB1B, NKIRAS1, MELK, CAMK1D, FRK, DCK, ASNS, GM5431, KIF9, NAIP6, NAIP2, TAP2, KIF4, TAP1, DCLK1, CHD3, ACTB, GIMAP3, ALPK1, FLT3, HCK, KIF18B, CENPE, SMC2, GSG2, SMC4, CYBB, GCK, GSPT2, TEP1, APAF1, JAK3, H2-DMA, CDK20	1916	1871	13288	1.33	1.49E-06	2.98E-07	1.84E-06
GOTERM_MF_FAT	GO:0000166~nucleotide binding	397	13.97	7.17E-08	TYRO3, GBP5, MYH3, LIG1, MYLK4, KIF5C, GBP9, PTBP1, MYLK2, MYH7, GEM, MYH9, NME4, NME5, MAPK7, EIF2AK2, GNAI3, GNAI2, UBA7, MYEF2, ZCRB1, ARF6, NAGK, RAC2, RAC3, DHX58, GPD1, SPHK1, TAOK3, FOXRED2, UBE2L6, VDAC3, VDAC1, KCNJ8, ARF3, ARF4, NUAK2, ATP10A, NOD2, GM9833, MASTL, SIK1, ARL5C, TARSL2, CDC7, RAP2B, CDC6, RAP2A, LYN, PHKG1, NDUFA13, PKDCC, 1700017B05RIK, BBS12, MAST3, PAPD4, SQLE, RIPK1, EIF4A1, ARL4C, RASD2, ENOX1, ARL4A, ALDH18A1, UGDH, POLA1, HK1, NLRP1A, ITM2C, IGSF10, PTK2B, CKMT2, HK3, BUB1, DAZL, CERK, PAPSS1, PAPSS2, TRIP13, ACSL5, ACTC1, SWAP70, PDK3, AXL, ACLY, PCK2, RPS6KA6, RPS6KA4, RPS6KA1, CHTF18, HPGD, DNM1, GNA15, RNASEL, HMGCR, RBM3, PASK, RRAD, MTHFD1L, PRKAR2B, NLRC4, NLRC3, RALB, SAR1B, RDM1, MATK, EGFR, PDXK, H2-OB, OAS1B, OAS1C, OAS1A, LRRK1, OAS1G, XDH, ME1, 9930111J21RIK1, ME3, ME2, ABCA9, FGR, ASS1, STK10, NT5C1A, PTK7, TIMP4, CTPS2, ABCA1, P2RY6, HELLS, TEC, ERCC6L, RAB8B, MYO1G, IDH3B, BRIP1, MYO1F, DOCK7, DOCK8, IDH3A, DDX58, IKBKE, DNA2, P2RX4, PLK4, P2RX7, P2RX6, PLK3, PLK2, PLK1, NDUFV2, MERTK, KIF23, KIF22, KIF24, ATL2, FIGNL1, SLFN9, ATL3, SLFN8, SLFN5, FES, SLFN3, SLFN4, SLFN1, ACSF2, SLFN2, KIF2C, TOR3A, RHOB, RHOD, SUCLA2, RHOG, RHOH, KIF14, CDK1, ACADM, KIF11, LIMK1, KIF15, CDK6, PBK, CDK2, ARL3, TOR2A, ATP6V1A, LCK, DCK, ASNS, KIF9, GM5431, NAIP6, NAIP2, KIF4, DCLK1, CHD3, ALPK1, FLT3, HCK, SMC2, SMC4, CYBB, GCK, DACH2, JAK3, APAF1, H2-DMA, ADCY3, ADCY7, AURKB, PRKG2, DDX11, RAB28, OASL2, OASL1, NT5C2, SUCLG1, POLE, IFI47, WNK2, RFC5, NAV1, RAB19, ACAP1, DLD, NEK8, PDGFRA, MDH1, 5430435G22RIK, CHKA, NEK2, PFKFB1, OAS3, CHEK1, MYO9B, IGF2BP2, OAS2, EPHB3, EPHB2, VRK1, MOV10, POLQ, ABCG3, RAD54L, DDX4, ATP13A5, ABCG1, EPHA2, EPHA3, EPHA7, GRK4, RAD54B, GM4951, CLCN5, MYO7A, CAMKK1, CKB, CAMKK2, ACTR3, ACTR2, TUBB5, TUBB6, DOCK10, TOP2A, DOCK11, TUBB3, NT5C, RAB4B, CBR4, RAD51, GM12250, ADK, STEAP2, STEAP1, MYO5A, RAB3A, RAB3D, CETN4, GCH1, GALK2, GALK1, RAB44, IGTP, RAB43, KIF21B, MYO5C, IRGM1, NOS1, ATP1A3, ATP1A4, RAB32, GM6548, RAB31, PYGM, PYGL, FYN, RAB34, PHGDH, ABCC3, KIF20B, GYK, RAB38, RBM14, CIT, KIF20A, PSTK, F13A1, TTK, PKMYT1, PI4K2B, ARL11, MLKL, MX1, MX2, CIITA, POR, MAP4K4, RIPK3, GBP3, PRPS2, GBP2, IFIH1, FGFR4, ACTR3B, MAP4K1, RRAGD, SRC, CMPK2, IRAK4, IRAK3, IIGP1, RUNX1, ENTPD1, RUNX2, RUNX3, CSF1R, EEF1A1, AK1, ATAD2, ATAD5, PSMB8, PSMB9, RAP1B, PIP4K2A, PPIP5K2, CRYL1, MCM7, ATP8B1, ATP8B2, MKKS, CSK, TUBA1A, TUBA1B, CDK15, TUBA1C, CDK14, ATP8B4, PFKL, PFKP, PFKM, MCM3, UBE2C, UBE2B, MCM4,	1916	2183	13288	1.26	9.50E-05	1.06E-05	1.17E-04
GOTERM_MF_FAT	GO:0005524~ATP binding	270	9.50	1.51E-06	ADCY3, KIFC1, ADCY7, AURKB, PRKG2, BTK, ACTG1, DDX11, OASL2, OASL1, MAP3K8, MAP2K6, PIK3CG, TYRO3, LIG1, MYH3, MYLK4, KIF5C, MYLK2, MYH7, WNK2, MYH9, RFC5, NME4, NME5, NAV1, ACAP1, NEK8, PDGFRA, MAPK7, EIF2AK2, CHKA, NEK2, PFKFB1, OAS3, UBA7, CHEK1, MYO9B, OAS2, NAGK, EPHB3, EPHB2, VRK1, MOV10, POLQ, DHX58, SPHK1, TAOK3, UBE2L6, ABCG3, DDX4, RAD54L, ATP13A5, EPHA2, ABCG1, EPHA3, EPHA7, KCNJ8, RAD54B, GRK4, CLCN5, NUAK2, MYO7A, ATP10A, CAMKK1, CAMKK2, CKB, ACTR3, ACTR2, NOD2, MASTL, SIK1, TOP2A, TARSL2, CDC7, CDC6, LYN, PHKG1, NDUFA13, PKDCC, BBS12, RAD51, MAST3, PAPD4, ADK, RIPK1, EIF4A1, MYO5A, ALDH18A1, CETN4, HK1, NLRP1A, ITM2C, IGSF10, GALK2, GALK1, PTK2B, CKMT2, HK3, BUB1, CERK, PAPSS1, PAPSS2, KIF21B, TRIP13, ACSL5, MYO5C, ACTC1, SWAP70, PDK3, ATP1A3, AXL, ATP1A4, ACLY, RPS6KA6, RPS6KA4, RPS6KA1, FYN, KIF20B, ABCC3, CHTF18, GYK, CIT, KIF20A, RNASEL, PSTK, F13A1, PASK, TTK, PKMYT1, PI4K2B, MTHFD1L, NLRC4, NLRC3, MLKL, MATK, EGFR, CIITA, PDXK, MAP4K4, H2-OB, RIPK3, OAS1B, OAS1C, OAS1A, LRRK1, OAS1G, PRPS2, IFIH1, FGFR4, ABCA9, FGR, ASS1, ACTR3B, STK10, PTK7, MAP4K1, CTPS2, TIMP4, ABCA1, SRC, CMPK2, IRAK4, IRAK3, ENTPD1, RUNX1, RUNX2, RUNX3, HELLS, CSF1R, ERCC6L, TEC, AK1, MYO1G, ATAD2, BRIP1, MYO1F, ATAD5, PSMB8, PSMB9, DDX58, IKBKE, P2RX4, DNA2, P2RX7, PLK4, P2RX6, PLK3, PLK2, PLK1, MERTK, PIP4K2A, KIF23, KIF22, KIF24, FIGNL1, SLFN9, PPIP5K2, SLFN8, SLFN5, FES, SLFN3, SLFN4, ACSF2, SLFN1, SLFN2, TOR3A, KIF2C, MCM7, ATP8B1, ATP8B2, MKKS, CSK, SUCLA2, CDK15, CDK14, ATP8B4, KIF14, CDK1, KIF11, PFKL, LIMK1, KIF15, PFKP, CDK6, PFKM, PBK, MCM3, UBE2C, UBE2B, MCM4, PRKCD, CDK2, MCM5, DAPK1, PRKCB, MCM6, TOR2A, ATP6V1A, LCK, BUB1B, H2-AA, MELK, CAMK1D, FRK, DCK, ASNS, KIF9, NAIP6, NAIP2, TAP2, KIF4, TAP1, DCLK1, CHD3, ACTB, ALPK1, FLT3, HCK, KIF18B, CENPE, SMC2, GSG2, SMC4, GCK, TEP1, APAF1, JAK3, H2-DMA, CDK20	1916	1443	13288	1.30	2.00E-03	1.11E-04	2.47E-03
GOTERM_MF_FAT	GO:0032559~adenyl ribonucleotide binding	271	9.54	2.85E-06	ADCY3, KIFC1, ADCY7, AURKB, PRKG2, BTK, ACTG1, DDX11, OASL2, OASL1, MAP3K8, MAP2K6, PIK3CG, TYRO3, LIG1, MYH3, MYLK4, KIF5C, MYLK2, MYH7, WNK2, MYH9, RFC5, NME4, NME5, NAV1, ACAP1, NEK8, PDGFRA, MAPK7, EIF2AK2, CHKA, NEK2, PFKFB1, OAS3, UBA7, CHEK1, MYO9B, OAS2, NAGK, EPHB3, EPHB2, VRK1, MOV10, POLQ, DHX58, SPHK1, TAOK3, UBE2L6, ABCG3, DDX4, RAD54L, ATP13A5, EPHA2, ABCG1, EPHA3, EPHA7, KCNJ8, RAD54B, GRK4, CLCN5, NUAK2, MYO7A, ATP10A, CAMKK1, CAMKK2, CKB, ACTR3, ACTR2, NOD2, MASTL, SIK1, TOP2A, TARSL2, CDC7, CDC6, LYN, PHKG1, NDUFA13, PKDCC, BBS12, RAD51, MAST3, PAPD4, ADK, RIPK1, EIF4A1, MYO5A, ALDH18A1, CETN4, HK1, NLRP1A, ITM2C, IGSF10, GALK2, GALK1, PTK2B, CKMT2, HK3, BUB1, CERK, PAPSS1, PAPSS2, KIF21B, TRIP13, ACSL5, MYO5C, ACTC1, SWAP70, PDK3, ATP1A3, AXL, ATP1A4, ACLY, RPS6KA6, RPS6KA4, RPS6KA1, FYN, KIF20B, ABCC3, CHTF18, GYK, CIT, KIF20A, RNASEL, PSTK, F13A1, PASK, TTK, PKMYT1, PI4K2B, MTHFD1L, PRKAR2B, NLRC4, NLRC3, MLKL, MATK, EGFR, CIITA, PDXK, MAP4K4, H2-OB, RIPK3, OAS1B, OAS1C, OAS1A, LRRK1, OAS1G, PRPS2, IFIH1, FGFR4, ABCA9, FGR, ASS1, ACTR3B, STK10, PTK7, MAP4K1, CTPS2, TIMP4, ABCA1, SRC, CMPK2, IRAK4, IRAK3, ENTPD1, RUNX1, RUNX2, RUNX3, HELLS, CSF1R, ERCC6L, TEC, AK1, MYO1G, ATAD2, BRIP1, MYO1F, ATAD5, PSMB8, PSMB9, DDX58, IKBKE, P2RX4, DNA2, P2RX7, PLK4, P2RX6, PLK3, PLK2, PLK1, MERTK, PIP4K2A, KIF23, KIF22, KIF24, FIGNL1, SLFN9, PPIP5K2, SLFN8, SLFN5, FES, SLFN3, SLFN4, ACSF2, SLFN1, SLFN2, TOR3A, KIF2C, MCM7, ATP8B1, ATP8B2, MKKS, CSK, SUCLA2, CDK15, CDK14, ATP8B4, KIF14, CDK1, KIF11, PFKL, LIMK1, KIF15, PFKP, CDK6, PFKM, PBK, MCM3, UBE2C, UBE2B, MCM4, PRKCD, CDK2, MCM5, DAPK1, PRKCB, MCM6, TOR2A, ATP6V1A, LCK, BUB1B, H2-AA, MELK, CAMK1D, FRK, DCK, ASNS, KIF9, NAIP6, NAIP2, TAP2, KIF4, TAP1, DCLK1, CHD3, ACTB, ALPK1, FLT3, HCK, KIF18B, CENPE, SMC2, GSG2, SMC4, GCK, TEP1, APAF1, JAK3, H2-DMA, CDK20	1916	1460	13288	1.29	3.77E-03	1.99E-04	4.67E-03

Category	Term	Count	%	PValue	Genes	List Total	Pop Hits	Pop Total	Fold Enrichment	Bonferroni	Benjamini	FDR
GOTERM_MF_FAT	GO:0030554~adenyl nucleotide binding	280	9.85	7.97E-06	ADCY3, KIFC1, ADCY7, AURKB, PRKG2, BTK, ACTG1, DDX11, OASL2, OASL1, MAP3K8, MAP2K6, PIK3CG, TYRO3, LIG1, MYH3, MYLK4, KIF5C, MYLK2, MYH7, WNK2, MYH9, RFC5, NME4, NME5, NAV1, ACAP1, DLD, NEK8, PDGFRA, MAPK7, EIF2AK2, CHKA, NEK2, PFKFB1, OAS3, UBA7, CHEK1, MYO9B, OAS2, NAGK, EPHB3, EPHB2, VRK1, MOV10, POLQ, DHX58, SPHK1, FOXRED2, TAOK3, UBE2L6, ABCG3, DDX4, RAD54L, ATP13A5, EPHA2, ABCG1, EPHA3, EPHA7, KCNJ8, GRK4, RAD54B, CLCN5, NUAK2, MYO7A, ATP10A, CAMKK1, CAMKK2, CKB, ACTR3, ACTR2, NOD2, MASTL, SIK1, TOP2A, TARSL2, CDC7, CDC6, LYN, PHKG1, NDUFA13, PKDCC, BBS12, RAD51, MAST3, PAPD4, SQLE, ADK, RIPK1, EIF4A1, STEAP2, STEAP1, MYO5A, ALDH18A1, CETN4, HK1, NLRP1A, ITM2C, IGSF10, GALK2, GALK1, PTK2B, CKMT2, HK3, BUB1, CERK, PAPSS1, PAPSS2, KIF21B, TRIP13, ACSL5, MYO5C, ACTC1, NOS1, SWAP70, PDK3, ATP1A3, AXL, ATP1A4, ACLY, RPS6KA6, RPS6KA4, RPS6KA1, FYN, KIF20B, ABCC3, CHTF18, GYK, CIT, KIF20A, RNASEL, PSTK, F13A1, PASK, TTK, PKMYT1, PI4K2B, MTHFD1L, PRKAR2B, NLRC4, NLRC3, MLKL, MATK, EGFR, CIITA, PDXK, MAP4K4, H2-OB, RIPK3, OAS1B, OAS1C, OAS1A, LRRK1, OAS1G, PRPS2, XDH, IFIH1, FGFR4, ABCA9, FGR, ASS1, ACTR3B, STK10, PTK7, MAP4K1, CTPS2, TIMP4, ABCA1, SRC, CMPK2, IRAK4, IRAK3, ENTPD1, RUNX1, RUNX2, HELLS, RUNX3, CSF1R, ERCC6L, TEC, AK1, MYO1G, ATAD2, BRIP1, MYO1F, ATAD5, PSMB8, PSMB9, DDX58, IKBKE, P2RX4, DNA2, P2RX7, PLK4, P2RX6, PLK3, PLK2, PLK1, MERTK, PIP4K2A, KIF23, KIF22, KIF24, FIGNL1, SLFN9, PPIP5K2, SLFN8, SLFN5, FES, SLFN3, SLFN4, ACSF2, SLFN1, SLFN2, TOR3A, KIF2C, MCM7, ATP8B1, ATP8B2, MKKS, CSK, SUCLA2, CDK15, CDK14, ATP8B4, KIF14, CDK1, ACADM, KIF11, PFKL, LIMK1, KIF15, PFKP, CDK6, PFKM, PBK, MCM3, UBE2C, PRKCD, MCM4, UBE2B, CDK2, MCM5, DAPK1, PRKCB, MCM6, TOR2A, ATP6V1A, LCK, BUB1B, H2-AA, MELK, CAMK1D, FRK, DCK, ASNS, KIF9, NAIP6, NAIP2, TAP2, KIF4, TAP1, DCLK1, CHD3, ACTB, ALPK1, FLT3, HCK, KIF18B, CENPE, SMC2, GSG2, SMC4, CYBB, GCK, TEP1, APAF1, JAK3, H2-DMA, CDK20	1916	1535	13288	1.27	1.05E-02	4.22E-04	1.30E-02
GOTERM_MF_FAT	GO:0001883~purine nucleoside binding	282	9.92	8.17E-06	ADCY3, KIFC1, ADCY7, AURKB, PRKG2, BTK, ACTG1, DDX11, OASL2, OASL1, MAP3K8, MAP2K6, PIK3CG, TYRO3, LIG1, MYH3, MYLK4, KIF5C, MYLK2, MYH7, WNK2, MYH9, RFC5, NME4, NME5, NAV1, ACAP1, DLD, NEK8, PDGFRA, MAPK7, EIF2AK2, CHKA, NEK2, PFKFB1, OAS3, UBA7, CHEK1, MYO9B, OAS2, NAGK, EPHB3, EPHB2, VRK1, MOV10, POLQ, DHX58, SPHK1, FOXRED2, TAOK3, UBE2L6, ABCG3, DDX4, RAD54L, ATP13A5, EPHA2, ABCG1, EPHA3, EPHA7, KCNJ8, GRK4, RAD54B, CLCN5, NUAK2, MYO7A, ATP10A, CAMKK1, CAMKK2, CKB, ACTR3, ACTR2, NOD2, MASTL, SIK1, TOP2A, TARSL2, CDC7, CDC6, LYN, PHKG1, NDUFA13, PKDCC, BBS12, RAD51, MAST3, PAPD4, SQLE, ADK, RIPK1, EIF4A1, STEAP2, STEAP1, MYO5A, ALDH18A1, CETN4, HK1, NLRP1A, ITM2C, IGSF10, GALK2, GALK1, PTK2B, CKMT2, HK3, BUB1, CERK, PAPSS1, PAPSS2, KIF21B, TRIP13, ACSL5, MYO5C, ACTC1, NOS1, SWAP70, PDK3, ATP1A3, AXL, ATP1A4, ACLY, RPS6KA6, RPS6KA4, RPS6KA1, FYN, KIF20B, ABCC3, CHTF18, GYK, CIT, KIF20A, RNASEL, PSTK, F13A1, PASK, TTK, PKMYT1, PI4K2B, MTHFD1L, PRKAR2B, NLRC4, NLRC3, MLKL, MATK, EGFR, CIITA, PDXK, MAP4K4, H2-OB, RIPK3, OAS1B, OAS1C, OAS1A, LRRK1, OAS1G, PRPS2, XDH, IFIH1, FGFR4, ABCA9, FGR, ASS1, ACTR3B, STK10, PTK7, MAP4K1, CTPS2, TIMP4, ABCA1, SRC, CMPK2, IRAK4, IRAK3, IIGP1, ENTPD1, RUNX1, RUNX2, HELLS, RUNX3, CSF1R, ERCC6L, TEC, AK1, MYO1G, ATAD2, BRIP1, MYO1F, ATAD5, PSMB8, PSMB9, DDX58, IKBKE, P2RX4, DNA2, P2RX7, PLK4, P2RX6, PLK3, PLK2, PLK1, MERTK, PIP4K2A, KIF23, KIF22, KIF24, FIGNL1, SLFN9, PPIP5K2, SLFN8, SLFN5, FES, SLFN3, SLFN4, ACSF2, SLFN1, SLFN2, TOR3A, KIF2C, MCM7, ATP8B1, RHOB, ATP8B2, MKKS, CSK, SUCLA2, CDK15, CDK14, ATP8B4, KIF14, CDK1, ACADM, KIF11, PFKL, LIMK1, KIF15, PFKP, CDK6, PFKM, PBK, MCM3, UBE2C, PRKCD, MCM4, UBE2B, CDK2, MCM5, DAPK1, PRKCB, MCM6, TOR2A, ATP6V1A, LCK, BUB1B, H2-AA, MELK, CAMK1D, FRK, DCK, ASNS, KIF9, NAIP6, NAIP2, TAP2, KIF4, TAP1, DCLK1, CHD3, ACTB, ALPK1, FLT3, HCK, KIF18B, CENPE, SMC2, GSG2, SMC4, CYBB, GCK, TEP1, APAF1, JAK3, H2-DMA, CDK20	1916	1548	13288	1.26	1.08E-02	4.16E-04	1.34E-02
GOTERM_MF_FAT	GO:0001882~nucleoside binding	283	9.96	1.00E-05	ADCY3, KIFC1, ADCY7, AURKB, PRKG2, BTK, ACTG1, DDX11, OASL2, OASL1, MAP3K8, MAP2K6, PIK3CG, TYRO3, LIG1, MYH3, MYLK4, KIF5C, MYLK2, MYH7, WNK2, MYH9, RFC5, NME4, NME5, NAV1, ACAP1, DLD, NEK8, PDGFRA, MAPK7, EIF2AK2, CHKA, NEK2, PFKFB1, OAS3, UBA7, CHEK1, MYO9B, OAS2, NAGK, EPHB3, EPHB2, VRK1, MOV10, POLQ, DHX58, SPHK1, FOXRED2, TAOK3, UBE2L6, ABCG3, DDX4, RAD54L, ATP13A5, EPHA2, ABCG1, EPHA3, EPHA7, KCNJ8, GRK4, RAD54B, CLCN5, NUAK2, MYO7A, ATP10A, CAMKK1, CAMKK2, CKB, ACTR3, ACTR2, NOD2, MASTL, SIK1, TOP2A, TARSL2, CDC7, CDC6, LYN, PHKG1, NDUFA13, PKDCC, BBS12, RAD51, MAST3, PAPD4, SQLE, ADK, RIPK1, EIF4A1, STEAP2, STEAP1, MYO5A, ALDH18A1, CETN4, POLA1, HK1, NLRP1A, ITM2C, IGSF10, GALK2, GALK1, PTK2B, CKMT2, HK3, BUB1, CERK, PAPSS1, PAPSS2, KIF21B, TRIP13, ACSL5, MYO5C, ACTC1, NOS1, SWAP70, PDK3, ATP1A3, AXL, ATP1A4, ACLY, RPS6KA6, RPS6KA4, RPS6KA1, FYN, KIF20B, ABCC3, CHTF18, GYK, CIT, KIF20A, RNASEL, PSTK, F13A1, PASK, TTK, PKMYT1, PI4K2B, MTHFD1L, PRKAR2B, NLRC4, NLRC3, MLKL, MATK, EGFR, CIITA, PDXK, MAP4K4, H2-OB, RIPK3, OAS1B, OAS1C, OAS1A, LRRK1, OAS1G, PRPS2, XDH, IFIH1, FGFR4, ABCA9, FGR, ASS1, ACTR3B, STK10, PTK7, MAP4K1, CTPS2, TIMP4, ABCA1, SRC, CMPK2, IRAK4, IRAK3, IIGP1, ENTPD1, RUNX1, RUNX2, HELLS, RUNX3, CSF1R, ERCC6L, TEC, AK1, MYO1G, ATAD2, BRIP1, MYO1F, ATAD5, PSMB8, PSMB9, DDX58, IKBKE, P2RX4, DNA2, P2RX7, PLK4, P2RX6, PLK3, PLK2, PLK1, MERTK, PIP4K2A, KIF23, KIF22, KIF24, FIGNL1, SLFN9, PPIP5K2, SLFN8, SLFN5, FES, SLFN3, SLFN4, ACSF2, SLFN1, SLFN2, TOR3A, KIF2C, MCM7, ATP8B1, RHOB, ATP8B2, MKKS, CSK, SUCLA2, CDK15, CDK14, ATP8B4, KIF14, CDK1, ACADM, KIF11, PFKL, LIMK1, KIF15, PFKP, CDK6, PFKM, PBK, MCM3, UBE2C, PRKCD, MCM4, UBE2B, CDK2, MCM5, DAPK1, PRKCB, MCM6, TOR2A, ATP6V1A, LCK, BUB1B, H2-AA, MELK, CAMK1D, FRK, DCK, ASNS, KIF9, NAIP6, NAIP2, TAP2, KIF4, TAP1, DCLK1, CHD3, ACTB, ALPK1, FLT3, HCK, KIF18B, CENPE, SMC2, GSG2, SMC4, CYBB, GCK, TEP1, APAF1, JAK3, H2-DMA, CDK20	1916	1558	13288	1.26	1.32E-02	4.75E-04	1.64E-02
Annotation Cluster 10	Enrichment Score: 6.7010035814563516											
Category	Term	Count	%	PValue	Genes	List Total	Pop Hits	Pop Total	Fold Enrichment	Bonferroni	Benjamini	FDR
GOTERM_BP_FAT	GO:0006897~endocytosis	60	2.11	4.54E-09	CD209A, MSR1, LDLR, MYO7A, WASF2, SYNJ1, MEGF10, CD47, DAB2, APP, NECAP2, LBP, CAP1, GHR, DBNL, CLN3, STXBP1, ARHGAP27, COLEC12, SIRPA, LDLRAP1, ELMO1, ZDHHC15, UNC13D, LRP12, RIN2, FKBP15, RIN1, STEAP2, ANKFY1, SH3GL1, RAB3A, CDC42SE1, NOSTRIN, PPT1, ABCA1, SLC11A1, DOCK1, FCER1G, MRC1, MICALL2, HCK, MRC2, STAB2, FCGR1, VAV1, FCGR3, CORO1C, FNBP1, P2RX7, LRP1, FCGR2B, RAB34, IRF8, LRP8, CLEC7A, MERTK, DNM1, CLCN5, VLDLR	2002	188	13588	2.17	1.84E-05	4.28E-07	8.45E-06
GOTERM_BP_FAT	GO:0010324~membrane invagination	60	2.11	4.54E-09	CD209A, MSR1, LDLR, MYO7A, WASF2, SYNJ1, MEGF10, CD47, DAB2, APP, NECAP2, LBP, CAP1, GHR, DBNL, CLN3, STXBP1, ARHGAP27, COLEC12, SIRPA, LDLRAP1, ELMO1, ZDHHC15, UNC13D, LRP12, RIN2, FKBP15, RIN1, STEAP2, ANKFY1, SH3GL1, RAB3A, CDC42SE1, NOSTRIN, PPT1, ABCA1, SLC11A1, DOCK1, FCER1G, MRC1, MICALL2, HCK, MRC2, STAB2, FCGR1, VAV1, FCGR3, CORO1C, FNBP1, P2RX7, LRP1, FCGR2B, RAB34, IRF8, LRP8, CLEC7A, MERTK, DNM1, CLCN5, VLDLR	2002	188	13588	2.17	1.84E-05	4.28E-07	8.45E-06
GOTERM_BP_FAT	GO:0006909~phagocytosis	24	0.84	7.78E-08	HCK, MYO7A, CDC42SE1, COLEC12, ABCA1, MEGF10, VAV1, SIRPA, FCGR1, FCGR3, ELMO1, CORO1C, CD47, SLC11A1, P2RX7, LRP1, UNC13D, DOCK1, FCGR2B, IRF8, FCER1G, LBP, CLEC7A, MERTK	2002	49	13588	3.32	3.16E-04	5.45E-06	1.45E-04
GOTERM_BP_FAT	GO:0016044~membrane organization	74	2.60	1.18E-07	CD209A, MSR1, LDLR, MYO7A, SYNJ1, WASF2, BNIP3, PMAIP1, GDNF, MEGF10, NRCAM, CD47, DAB2, APP, NECAP2, CAP1, LBP, GHR, CLN3, DBNL, CCDC88A, STXBP1, ARHGAP27, COLEC12, LPCAT2, LDLRAP1, SIRPA, TIMM8B, ELMO1, ZDHHC15, UNC13D, LRP12, RIN2, RIN1, FKBP15, STEAP2, ANKFY1, SH3GL1, BID, RAB3A, CDC42SE1, NOSTRIN, PPT1, ABCA1, CD9, SLC11A1, DOCK2, DOCK1, FCER1G, MRC1, MICALL2, VAV3, A4GALT, HCK, MRC2, LMNA, STAB2, VAV1, FCGR1, FCGR3, CORO1C, KCNN4, FNBP1, P2RX7, LRP1, FCGR2B, RAB34, IRF8, LRP8, CLEC7A, MERTK, DNM1, CLCN5, VLDLR	2002	272	13588	1.85	4.79E-04	7.86E-06	2.20E-04
Annotation Cluster 11	Enrichment Score: 6.369786736238554											
Category	Term	Count	%	PValue	Genes	List Total	Pop Hits	Pop Total	Fold Enrichment	Bonferroni	Benjamini	FDR
GOTERM_MF_FAT	GO:0030246~carbohydrate binding	93	3.27	8.84E-12	CD209A, KLRA17, SUSD2, PGLYRP1, FSTL1, POSTN, GPCPD1, OS9, NOD2, APP, MAN2B2, CD44, CD93, CLEC4E, APOE, CTGF, APOH, CLEC4A1, CLEC4A2, CLEC4A3, CLEC4D, MAN2B1, KLRD1, CLEC4A4, COLEC12, PFKM, CLEC4N, NCAM1, MAN2A1, SIGLEC1, TNFAIP6, EGFLAM, BGN, CLEC12A, CD33, LAMC2, CLEC4B1, VCAN, CLEC5A, SIGLECE, GALNT7, MAMDC2, ENPP1, GALNT6, CD248, FCNA, GALNT4, HSD17B12, KLRK1, CCL8, PF4, CALR, CLEC10A, ITGAM, CCL7, HMMR, CD69, COMP, CD22, PTN, CLEC2E, GPNMB, THBS1, SELPLG, THBS2, GALNT12, AGL, CLEC2I, FN1, KLRA2, MRC1, PTPRC, MAG, SELP, LGALS3, SELL, FBN1, MRC2, CCDC80, CHI3L3, STAB2, COL5A3, CLEC11A, COL5A1, LGALS9, LYVE1, GCK, STAB1, LAYN, GLT28D2, CLEC7A, KLRB1B, CD302	1916	317	13288	2.03	1.17E-08	1.17E-08	1.45E-08
GOTERM_MF_FAT	GO:0030247~polysaccharide binding	42	1.48	3.02E-07	ENPP1, MAMDC2, SUSD2, HSD17B12, PGLYRP1, CCL8, POSTN, PF4, FSTL1, ITGAM, CCL7, HMMR, NOD2, APP, CD44, APOE, CTGF, COMP, APOH, PTN, THBS1, GPNMB, THBS2, AGL, FN1, PTPRC, FBN1, CCDC80, CHI3L3, STAB2, COL5A3, COL5A1, NCAM1, TNFAIP6, LYVE1, EGFLAM, BGN, STAB1, LAYN, VCAN, LAMC2, CLEC7A	1916	128	13288	2.28	4.00E-04	2.86E-05	4.95E-04
GOTERM_MF_FAT	GO:0001871~pattern binding	42	1.48	3.02E-07	ENPP1, MAMDC2, SUSD2, HSD17B12, PGLYRP1, CCL8, POSTN, PF4, FSTL1, ITGAM, CCL7, HMMR, NOD2, APP, CD44, APOE, CTGF, COMP, APOH, PTN, THBS1, GPNMB, THBS2, AGL, FN1, PTPRC, FBN1, CCDC80, CHI3L3, STAB2, COL5A3, COL5A1, NCAM1, TNFAIP6, LYVE1, EGFLAM, BGN, STAB1, LAYN, VCAN, LAMC2, CLEC7A	1916	128	13288	2.28	4.00E-04	2.86E-05	4.95E-04
GOTERM_MF_FAT	GO:0005539~glycosaminoglycan binding	36	1.27	6.38E-06	MAMDC2, HSD17B12, PGLYRP1, CCL8, POSTN, PF4, FSTL1, ITGAM, CCL7, HMMR, NOD2, APP, CD44, APOE, CTGF, COMP, APOH, PTN, GPNMB, THBS1, THBS2, FN1, PTPRC, CCDC80, STAB2, COL5A3, COL5A1, NCAM1, TNFAIP6, LYVE1, BGN, EGFLAM, STAB1, LAYN, LAMC2, VCAN	1916	114	13288	2.19	8.42E-03	3.67E-04	1.04E-02
Annotation Cluster 12	Enrichment Score: 5.982599763257034											
Category	Term	Count	%	PValue	Genes	List Total	Pop Hits	Pop Total	Fold Enrichment	Bonferroni	Benjamini	FDR
GOTERM_BP_FAT	GO:0002822~regulation of adaptive immune response based on somatic recombination of immune rec	33	1.16	2.21E-13	IL27RA, C3, IL4RA, TNFSF13, CD1D1, IL7R, PNP, TGFB1, ADA, B2M, SLC11A1, NOD2, TAP2, HLX, PPP3CB, FCER1G, SPN, CD28, H2-K1, FCER1A, PTPN6, PTPRC, IL6, H2-M3, CENPF, CD40, H2-Q7, FCGR1, FCGR3, P2RX7, FCGR2B, TNFSF13B, SASH3	2002	56	13588	4.00	8.98E-10	4.73E-11	4.12E-10
GOTERM_BP_FAT	GO:0002819~regulation of adaptive immune response	33	1.16	2.21E-13	IL27RA, C3, IL4RA, TNFSF13, CD1D1, IL7R, PNP, TGFB1, ADA, B2M, SLC11A1, NOD2, TAP2, HLX, PPP3CB, FCER1G, SPN, CD28, H2-K1, FCER1A, PTPN6, PTPRC, IL6, H2-M3, CENPF, CD40, H2-Q7, FCGR1, FCGR3, P2RX7, FCGR2B, TNFSF13B, SASH3	2002	56	13588	4.00	8.98E-10	4.73E-11	4.12E-10
GOTERM_BP_FAT	GO:0002697~regulation of immune effector process	42	1.48	9.22E-13	RBP4, TNF, ADORA2B, IL27RA, C3, KLRK1, TNFSF13, CD1D1, IL7R, PNP, TGFB1, B2M, NOD2, SH2D1B1, TAP2, HMOX1, PPP3CB, FCER1G, LBP, LAG3, SPN, CD28, H2-K1, FCER1A, PTPRC, PTPN6, H2-M3, CENPF, CD40, FCGR1, H2-Q7, FOXP1, FCGR3, DDX58, CD37, P2RX7, FCGR2B, CD59B, ULBP1, PRAM1, KLRB1B, SASH3	2002	88	13588	3.24	3.74E-09	1.87E-10	1.72E-09
GOTERM_BP_FAT	GO:0002703~regulation of leukocyte mediated immunity	35	1.23	1.89E-11	ADORA2B, IL27RA, C3, KLRK1, TNFSF13, CD1D1, IL7R, PNP, TGFB1, B2M, NOD2, SH2D1B1, TAP2, HMOX1, PPP3CB, FCER1G, LAG3, SPN, CD28, H2-K1, FCER1A, PTPN6, PTPRC, H2-M3, CENPF, CD40, H2-Q7, FCGR1, FCGR3, P2RX7, FCGR2B, ULBP1, PRAM1, KLRB1B, SASH3	2002	70	13588	3.39	7.67E-08	2.84E-09	3.51E-08
GOTERM_BP_FAT	GO:0002706~regulation of lymphocyte mediated immunity	32	1.13	2.63E-10	IL27RA, C3, KLRK1, TNFSF13, CD1D1, IL7R, PNP, TGFB1, B2M, NOD2, SH2D1B1, TAP2, PPP3CB, FCER1G, LAG3, SPN, CD28, H2-K1, FCER1A, PTPN6, PTPRC, H2-M3, CENPF, CD40, H2-Q7, FCGR1, FCGR3, P2RX7, FCGR2B, ULBP1, KLRB1B, SASH3	2002	65	13588	3.34	1.07E-06	3.44E-08	4.89E-07
GOTERM_BP_FAT	GO:0002824~positive regulation of adaptive immune response based on somatic recombination of imm	21	0.74	3.65E-09	H2-K1, FCER1A, PTPRC, H2-M3, IL27RA, C3, TNFSF13, CD1D1, PNP, H2-Q7, FCGR1, ADA, B2M, FCGR3, SLC11A1, P2RX7, NOD2, TNFSF13B, TAP2, FCER1G, SASH3	2002	34	13588	4.19	1.48E-05	3.61E-07	6.79E-06
GOTERM_BP_FAT	GO:0002821~positive regulation of adaptive immune response	21	0.74	3.65E-09	H2-K1, FCER1A, PTPRC, H2-M3, IL27RA, C3, TNFSF13, CD1D1, PNP, H2-Q7, FCGR1, ADA, B2M, FCGR3, SLC11A1, P2RX7, NOD2, TNFSF13B, TAP2, FCER1G, SASH3	2002	34	13588	4.19	1.48E-05	3.61E-07	6.79E-06
GOTERM_BP_FAT	GO:0001914~regulation of T cell mediated cytotoxicity	11	0.39	4.49E-08	H2-K1, PTPRC, P2RX7, H2-M3, TAP2, PPP3CB, IL7R, CD1D1, H2-Q7, PNP, B2M	2002	11	13588	6.79	1.82E-04	3.57E-06	8.35E-05
GOTERM_BP_FAT	GO:0002709~regulation of T cell mediated immunity	14	0.49	6.12E-08	H2-K1, PTPRC, H2-M3, CENPF, IL7R, CD1D1, PNP, H2-Q7, B2M, P2RX7, TAP2, PPP3CB, SPN, SASH3	2002	18	13588	5.28	2.48E-04	4.52E-06	1.14E-04
GOTERM_BP_FAT	GO:0002699~positive regulation of immune effector process	23	0.81	9.79E-08	H2-K1, FCER1A, PTPRC, ADORA2B, H2-M3, C3, KLRK1, CD1D1, PNP, H2-Q7, FCGR1, FOXP1, FCGR3, B2M, CD37, P2RX7, NOD2, ULBP1, TAP2, FCER1G, LBP, LAG3, SASH3	2002	46	13588	3.39	3.98E-04	6.63E-06	1.82E-04
GOTERM_BP_FAT	GO:0001916~positive regulation of T cell mediated cytotoxicity	9	0.32	1.71E-06	H2-K1, PTPRC, P2RX7, H2-M3, TAP2, CD1D1, H2-Q7, PNP, B2M	2002	9	13588	6.79	6.92E-03	7.55E-05	3.18E-03
GOTERM_BP_FAT	GO:0002708~positive regulation of lymphocyte mediated immunity	19	0.67	4.31E-06	H2-K1, FCER1A, PTPRC, H2-M3, C3, KLRK1, CD1D1, PNP, H2-Q7, FCGR1, B2M, FCGR3, P2RX7, NOD2, TAP2, ULBP1, FCER1G, LAG3, SASH3	2002	40	13588	3.22	1.74E-02	1.81E-04	8.03E-03
GOTERM_BP_FAT	GO:0002705~positive regulation of leukocyte mediated immunity	19	0.67	4.31E-06	H2-K1, FCER1A, PTPRC, H2-M3, C3, KLRK1, CD1D1, PNP, H2-Q7, FCGR1, B2M, FCGR3, P2RX7, NOD2, TAP2, ULBP1, FCER1G, LAG3, SASH3	2002	40	13588	3.22	1.74E-02	1.81E-04	8.03E-03
GOTERM_BP_FAT	GO:0031341~regulation of cell killing	16	0.56	5.44E-06	H2-K1, PTPRC, H2-M3, KLRK1, IL7R, CD1D1, PNP, H2-Q7, B2M, P2RX7, SH2D1B1, TAP2, ULBP1, PPP3CB, KLRB1B, LAG3	2002	30	13588	3.62	2.19E-02	2.23E-04	1.01E-02

GOTERM_BP_FAT	GO:0001910~regulation of leukocyte mediated cytotoxicity	16	0.56	5.44E-06	H2-K1, PTPRC, H2-M3, KLRK1, IL7R, CD1D1, PNP, H2-Q7, B2M, P2RX7, SH2D1B1, TAP2, ULBP1, PPP3CB, KLRB1B, LAG3	2002	30	13588	3.62	2.19E-02	2.23E-04	1.01E-02
GOTERM_BP_FAT	GO:0002711~positive regulation of T cell mediated immunity	10	0.35	1.30E-05	H2-K1, PTPRC, P2RX7, H2-M3, TAP2, CD1D1, H2-Q7, PNP, SASH3, B2M	2002	13	13588	5.22	5.15E-02	4.77E-04	2.43E-02
Annotation Cluster 13	Enrichment Score: 5.973027397229028											
Category	Term	Count	%	PValue	Genes	List Total	Pop Hits	Pop Total	Fold Enrichment	Bonferroni	Benjamini	FDR
GOTERM_BP_FAT	GO:0043068~positive regulation of programmed cell death	69	2.43	1.84E-07	PDIA3, MMP9, IL18, BNIP3, PMAIP1, TGFB1, CASP6, GPX1, NLRC4, CASP3, CASP4, CASP9, APOE, BOK, HMOX1, TICAM1, POU4F1, NGFRAP1, CASP1, NDUFS3, MYC, LTB, SPN, RAB27A, CYCS, SKP2, NDUFA13, GZMB, GAL, PRKCD, BCL2L11, DAPK1, UNC13D, LCK, RIPK3, BID, CDK5R1, LST1, TNF, NFKBID, PML, NLRP1A, SP110, SRC, GCH1, ALDH1A1, SHISA5, PYCARD, BCL3, INPP5D, CD27, RUNX3, CD28, COL18A1, PTPRC, PTPN6, FCGR1, BRCA1, FCGR3, TRADD, NOTCH2, EPHA7, P2RX7, CDKN1A, BBC3, ENDOG, APAF1, IFI204, BARD1	2002	250	13588	1.87	7.48E-04	1.15E-05	3.43E-04
GOTERM_BP_FAT	GO:0010942~positive regulation of cell death	69	2.43	2.55E-07	PDIA3, MMP9, IL18, BNIP3, PMAIP1, TGFB1, CASP6, GPX1, NLRC4, CASP3, CASP4, CASP9, APOE, BOK, HMOX1, TICAM1, POU4F1, NGFRAP1, CASP1, NDUFS3, MYC, LTB, SPN, RAB27A, CYCS, SKP2, NDUFA13, GZMB, GAL, PRKCD, BCL2L11, DAPK1, UNC13D, LCK, RIPK3, BID, CDK5R1, LST1, TNF, NFKBID, PML, NLRP1A, SP110, SRC, GCH1, ALDH1A1, SHISA5, PYCARD, BCL3, INPP5D, CD27, RUNX3, CD28, COL18A1, PTPRC, PTPN6, FCGR1, BRCA1, FCGR3, TRADD, NOTCH2, EPHA7, P2RX7, CDKN1A, BBC3, ENDOG, APAF1, IFI204, BARD1	2002	252	13588	1.86	1.04E-03	1.48E-05	4.75E-04
GOTERM_BP_FAT	GO:0043065~positive regulation of apoptosis	68	2.39	2.98E-07	PDIA3, MMP9, IL18, BNIP3, PMAIP1, TGFB1, CASP6, GPX1, NLRC4, CASP3, CASP4, CASP9, APOE, BOK, HMOX1, TICAM1, POU4F1, NGFRAP1, CASP1, NDUFS3, MYC, LTB, SPN, RAB27A, CYCS, SKP2, NDUFA13, GZMB, GAL, PRKCD, BCL2L11, DAPK1, UNC13D, LCK, RIPK3, BID, CDK5R1, LST1, TNF, NFKBID, PML, NLRP1A, SP110, SRC, GCH1, ALDH1A1, SHISA5, PYCARD, BCL3, INPP5D, CD27, RUNX3, CD28, COL18A1, PTPRC, PTPN6, FCGR1, BRCA1, FCGR3, TRADD, NOTCH2, EPHA7, P2RX7, BBC3, ENDOG, APAF1, IFI204, BARD1	2002	248	13588	1.86	1.21E-03	1.70E-05	5.54E-04
GOTERM_BP_FAT	GO:0010941~regulation of cell death	127	4.47	4.83E-07	STIL, MMP9, IL18, PMAIP1, GDNF, PNP, TGFB1, NLRC4, CUL7, APOE, NGFRAP1, SPN, RAB27A, PIK3CG, ACTN4, MADD, SKP2, POLB, BCL2L11, NME5, HIF1A, UNC13D, CD59B, CX3CR1, RIPK3, NGF, TRAF1, IFIH1, CDK5R1, LST1, NFKBID, PPT1, SP110, SOX9, ADA, CD74, SRC, IRAK3, FCER1G, FAIM, BMF, HELLS, RUNX3, TRAF4, ANGPTL4, SPP1, COL18A1, PTPN6, TCF7, CARD9, SPHK1, HGF, GAS1, FCGR1, BRCA1, FCGR3, TRADD, NOTCH2, EPHA7, BCL2A1D, P2RX7, CDKN1A, BCL2A1B, BCL2A1A, BBC3, PLCG2, PDIA3, NUAK2, BNIP3, GLI3, KCNIP3, CASP6, GPX1, PCGF2, NOD2, CASP3, CASP4, CASP9, BOK, HMOX1, CASP8, TICAM1, POU4F1, CASP1, NDUFS3, MYC, LTB, CLN3, CYCS, NDUFA13, GZMB, GAL, PRKCD, UBE2B, DAPK1, SMO, CARD11, CARD14, TNFSF13B, BTG1, LCK, CAMK1D, BID, TNFRSF22, TNF, PRND, PML, NLRP1A, NLRP1B, GCH1, ALDH1A1, SHISA5, PYCARD, BCL3, INPP5D, CD27, CD28, PTPRC, IL2RB, IL6, CENPF, BIRC5, SNAI2, ENDOG, APAF1, BARD1, IFI204	2002	563	13588	1.53	1.96E-03	2.48E-05	8.98E-04
GOTERM_BP_FAT	GO:0042981~regulation of apoptosis	125	4.40	5.35E-07	STIL, MMP9, IL18, PMAIP1, GDNF, PNP, TGFB1, NLRC4, CUL7, APOE, NGFRAP1, SPN, RAB27A, PIK3CG, ACTN4, MADD, SKP2, POLB, BCL2L11, NME5, HIF1A, UNC13D, CX3CR1, RIPK3, NGF, TRAF1, IFIH1, CDK5R1, LST1, NFKBID, PPT1, SP110, SOX9, ADA, CD74, SRC, IRAK3, FCER1G, FAIM, BMF, HELLS, RUNX3, TRAF4, ANGPTL4, SPP1, COL18A1, PTPN6, TCF7, CARD9, SPHK1, HGF, GAS1, FCGR1, BRCA1, FCGR3, TRADD, NOTCH2, EPHA7, BCL2A1D, P2RX7, CDKN1A, BCL2A1B, BCL2A1A, BBC3, PDIA3, NUAK2, BNIP3, GLI3, KCNIP3, CASP6, GPX1, PCGF2, NOD2, CASP3, CASP4, CASP9, BOK, HMOX1, CASP8, TICAM1, POU4F1, CASP1, NDUFS3, MYC, LTB, CLN3, CYCS, NDUFA13, GZMB, GAL, PRKCD, UBE2B, DAPK1, SMO, CARD11, CARD14, TNFSF13B, BTG1, LCK, CAMK1D, TNFRSF22, BID, TNF, PRND, PML, NLRP1A, NLRP1B, GCH1, ALDH1A1, SHISA5, PYCARD, BCL3, INPP5D, CD27, CD28, PTPRC, IL2RB, IL6, CENPF, BIRC5, SNAI2, ENDOG, APAF1, BARD1, IFI204	2002	553	13588	1.53	2.17E-03	2.68E-05	9.96E-04
GOTERM_BP_FAT	GO:0043067~regulation of programmed cell death	126	4.43	6.20E-07	STIL, MMP9, IL18, PMAIP1, GDNF, PNP, TGFB1, NLRC4, CUL7, APOE, NGFRAP1, SPN, RAB27A, PIK3CG, ACTN4, MADD, SKP2, POLB, BCL2L11, NME5, HIF1A, UNC13D, CX3CR1, RIPK3, NGF, TRAF1, IFIH1, CDK5R1, LST1, NFKBID, PPT1, SP110, SOX9, ADA, CD74, SRC, IRAK3, FCER1G, FAIM, BMF, HELLS, RUNX3, TRAF4, ANGPTL4, SPP1, COL18A1, PTPN6, TCF7, CARD9, SPHK1, HGF, GAS1, FCGR1, BRCA1, FCGR3, TRADD, NOTCH2, EPHA7, BCL2A1D, P2RX7, CDKN1A, BCL2A1B, BCL2A1A, BBC3, PLCG2, PDIA3, NUAK2, BNIP3, GLI3, KCNIP3, CASP6, GPX1, PCGF2, NOD2, CASP3, CASP4, CASP9, BOK, HMOX1, CASP8, TICAM1, POU4F1, CASP1, NDUFS3, MYC, LTB, CLN3, CYCS, NDUFA13, GZMB, GAL, PRKCD, UBE2B, DAPK1, SMO, CARD11, CARD14, TNFSF13B, BTG1, LCK, CAMK1D, TNFRSF22, BID, TNF, PRND, PML, NLRP1A, NLRP1B, GCH1, ALDH1A1, SHISA5, PYCARD, BCL3, INPP5D, CD27, CD28, PTPRC, IL2RB, IL6, CENPF, BIRC5, SNAI2, ENDOG, APAF1, BARD1, IFI204	2002	560	13588	1.53	2.51E-03	3.07E-05	1.15E-03
Annotation Cluster 14	Enrichment Score: 5.779005193758813											
Category	Term	Count	%	PValue	Genes	List Total	Pop Hits	Pop Total	Fold Enrichment	Bonferroni	Benjamini	FDR
GOTERM_BP_FAT	GO:0042330~taxis	45	1.58	5.52E-11	NRP2, C3AR1, PLXNA3, CCL3, CCL2, S100A8, IL16, CYSLTR1, CXCL5, CCR1, SLC37A4, S100A9, CCL9, CCL8, CXCR2, ITGB2, PF4, FPR2, CCL5, CXCL12, CCL4, ITGAM, CCL7, CCL6, CCL22, DOCK2, RAC2, ROBO1, CKLF, CSF3R, IL1B, FCER1G, LBP, C5AR1, FCGR3, CCL12, CORO1A, CCR7, CXCL13, CXCL16, CX3CR1, CMTM7, CMTM3, CMTM6, PTAFR	2002	109	13588	2.80	2.24E-07	7.73E-09	1.03E-07
GOTERM_BP_FAT	GO:0006935~chemotaxis	45	1.58	5.52E-11	NRP2, C3AR1, PLXNA3, CCL3, CCL2, S100A8, IL16, CYSLTR1, CXCL5, CCR1, SLC37A4, S100A9, CCL9, CCL8, CXCR2, ITGB2, PF4, FPR2, CCL5, CXCL12, CCL4, ITGAM, CCL7, CCL6, CCL22, DOCK2, RAC2, ROBO1, CKLF, CSF3R, IL1B, FCER1G, LBP, C5AR1, FCGR3, CCL12, CORO1A, CCR7, CXCL13, CXCL16, CX3CR1, CMTM7, CMTM3, CMTM6, PTAFR	2002	109	13588	2.80	2.24E-07	7.73E-09	1.03E-07
GOTERM_BP_FAT	GO:0030595~leukocyte chemotaxis	15	0.53	6.50E-06	C5AR1, IL16, CCR1, S100A9, SLC37A4, ITGB2, PF4, ITGAM, FCGR3, CORO1A, CX3CR1, FCER1G, IL1B, CSF3R, LBP	2002	27	13588	3.77	2.60E-02	2.56E-04	1.21E-02
GOTERM_BP_FAT	GO:0060326~cell chemotaxis	15	0.53	6.50E-06	C5AR1, IL16, CCR1, S100A9, SLC37A4, ITGB2, PF4, ITGAM, FCGR3, CORO1A, CX3CR1, FCER1G, IL1B, CSF3R, LBP	2002	27	13588	3.77	2.60E-02	2.56E-04	1.21E-02
GOTERM_BP_FAT	GO:0007626~locomotory behavior	62	2.18	7.42E-06	NRP2, PLXNA3, S100A8, IL16, HEXA, HEXB, UCHL1, S100A9, CXCR2, CXCL12, APP, ROBO1, CSF3R, IL1B, LBP, SEPP1, C5AR1, CCR7, CX3CR1, CSTB, RASD2, CHL1, PTAFR, MYO5A, C3AR1, CCL3, CCL2, CYSLTR1, CXCL5, CCR1, SLC37A4, CCL9, CCL8, PPT1, PF4, ITGB2, FPR2, CCL5, CCL4, CCL7, ITGAM, CCL6, CCL22, DOCK2, PDE1B, RAC2, CKLF, FCER1G, APBA1, NTF5, ATP1A3, NPY1R, PARK7, FCGR3, CCL12, CORO1A, EPS8, CXCL13, CXCL16, CMTM7, CMTM3, CMTM6	2002	239	13588	1.76	2.97E-02	2.81E-04	1.38E-02
Annotation Cluster 15	Enrichment Score: 5.46846968893073											
Category	Term	Count	%	PValue	Genes	List Total	Pop Hits	Pop Total	Fold Enrichment	Bonferroni	Benjamini	FDR
GOTERM_BP_FAT	GO:0030029~actin filament-based process	54	1.90	1.26E-07	LIMA1, NUAK2, AIF1, S100A9, WASF2, ACTG1, TMSB15B1, CAP1, RHOG, FMNL1, DBNL, ARHGEF2, ACTN4, ACTN1, TMSB10, MYH9, SIRPA, WAS, FLNA, ELMO1, TNNT2, FMN1, CFL1, TMSB4X, DBN1, LCP1, MYO5A, TCAP, CNN3, MYL2, ARPC4, MYO9B, CALR, PFN2, EZR, RAC2, RAC3, FAT1, SH2B2, CNN2, WIPF1, INF2, ACTC1, RACGAP1, GAS7, CORO1C, EPB4.1L2, EPB4.1L3, CORO1A, EPB4.1L1, EPS8, ANTXR1, DIAP2, DIAP3	2002	176	13588	2.08	5.10E-04	8.09E-06	2.34E-04
GOTERM_BP_FAT	GO:0030036~actin cytoskeleton organization	51	1.79	2.28E-07	LIMA1, NUAK2, AIF1, S100A9, WASF2, ACTG1, TMSB15B1, CAP1, RHOG, FMNL1, DBNL, ARHGEF2, ACTN4, ACTN1, TMSB10, MYH9, SIRPA, WAS, FLNA, ELMO1, TNNT2, FMN1, CFL1, TMSB4X, DBN1, LCP1, TCAP, CNN3, MYL2, ARPC4, CALR, PFN2, EZR, RAC2, RAC3, FAT1, SH2B2, CNN2, INF2, ACTC1, RACGAP1, GAS7, EPB4.1L2, CORO1C, EPB4.1L3, CORO1A, EPB4.1L1, EPS8, ANTXR1, DIAP2, DIAP3	2002	165	13588	2.10	9.27E-04	1.36E-05	4.25E-04
GOTERM_BP_FAT	GO:0007010~cytoskeleton organization	79	2.78	6.03E-06	CEP72, LIMA1, NUAK2, AIF1, WASF2, S100A9, VILL, ACTG1, TUBB5, TMSB15B1, CAP1, TUBA1B, RHOG, FMNL1, DBNL, ARHGEF2, KIF11, ACTN4, ACTN1, NUSAP1, TMSB10, ESPL1, PLK1S1, MYH9, TACC3, WAS, SIRPA, UBE2B, FLNA, TACC1, THY1, ELMO1, FMN1, TNNT2, NAV1, TPPP, CFL1, TMSB4X, STMN1, HAUS8, DBN1, LCP1, MYL2, CNN3, TCAP, ARPC4, CALR, SPC25, PFN2, DOCK2, EZR, RAC2, RAC3, FAT1, FBXO5, CNN2, SH2B2, TES, INF2, ACTC1, CEP192, NUF2, BFSP2, CENPE, NDC80, BIRC5, RACGAP1, GAS7, BRCA1, CORO1C, EPB4.1L2, EPB4.1L3, CORO1A, EPB4.1L1, EPS8, LASP1, ANTXR1, DIAP2, DIAP3	2002	326	13588	1.64	2.42E-02	2.40E-04	1.12E-02
Annotation Cluster 16	Enrichment Score: 5.420191379823401											
Category	Term	Count	%	PValue	Genes	List Total	Pop Hits	Pop Total	Fold Enrichment	Bonferroni	Benjamini	FDR
GOTERM_MF_FAT	GO:0016651~oxidoreductase activity, acting on NADH or NADPH	24	0.84	1.30E-07	CYB5R3, NDUFB3, NDUFA5, NDUFB4, NDUFA2, GMPR2, NCF2, NDUFB8, NCF1, NDUFA7, NDUFC2, NDUFA13, CBR4, GMPR, ECSIT, NDUFAF1, POR, NDUFA12, NDUFS7, NDUFS4, NDUFS8, NDUFV2, DLD, NDUFS3	1916	51	13288	3.26	1.72E-04	1.72E-05	2.13E-04
GOTERM_MF_FAT	GO:0050136~NADH dehydrogenase (quinone) activity	14	0.49	5.84E-06	NDUFB3, NDUFB4, NDUFA2, NDUFB8, NDUFA7, NDUFC2, NDUFA13, NDUFAF1, NDUFA12, NDUFS7, NDUFS4, NDUFV2, NDUFS8, NDUFS3	1916	24	13288	4.05	7.70E-03	3.51E-04	9.55E-03
GOTERM_MF_FAT	GO:0003954~NADH dehydrogenase activity	14	0.49	5.84E-06	NDUFB3, NDUFB4, NDUFA2, NDUFB8, NDUFA7, NDUFC2, NDUFA13, NDUFAF1, NDUFA12, NDUFS7, NDUFS4, NDUFV2, NDUFS8, NDUFS3	1916	24	13288	4.05	7.70E-03	3.51E-04	9.55E-03
GOTERM_MF_FAT	GO:0008137~NADH dehydrogenase (ubiquinone) activity	14	0.49	5.84E-06	NDUFB3, NDUFB4, NDUFA2, NDUFB8, NDUFA7, NDUFC2, NDUFA13, NDUFAF1, NDUFA12, NDUFS7, NDUFS4, NDUFV2, NDUFS8, NDUFS3	1916	24	13288	4.05	7.70E-03	3.51E-04	9.55E-03
Annotation Cluster 17	Enrichment Score: 5.342784405964592											
Category	Term	Count	%	PValue	Genes	List Total	Pop Hits	Pop Total	Fold Enrichment	Bonferroni	Benjamini	FDR
GOTERM_BP_FAT	GO:0005996~monosaccharide metabolic process	67	2.36	4.84E-12	LDHA, GNPDA1, PHKB, G6PDX, PGD, PGAM1, PGAM2, ACN9, PDHB, MAN2B2, UGT1A7C, UGT1A6B, PPP1R1A, MAN2B1, MYC, B3GALTL, PFKL, PHKG1, PFKP, CHST2, CMAH, PFKM, PPP1R3A, FBP2, PGM2, MAN2A1, RENBP, PGM1, SERP1, MDH1, RBP4, TNF, PFKFB1, SLC37A4, PHKA1, FN3K, HK1, NAGK, GALK2, GALK1, TPI1, PPP1R3C, PPP1R3B, HK3, ENO2, ENO3, GALE, GNMT, AGL, ENO1, GPD2, ALDOART1, GPD1, GMDS, PDK3, BPGM, NPY1R, PCK2, MAN1C1, CPT1A, CSGALNACT1, AMDHD2, ATF3, PYGM, GCK, PYGL, FABP5, PYGB	2002	191	13588	2.38	1.97E-08	8.55E-10	9.01E-09
GOTERM_BP_FAT	GO:0019318~hexose metabolic process	60	2.11	4.06E-11	LDHA, GNPDA1, PHKB, G6PDX, PGD, PGAM1, PGAM2, ACN9, PDHB, MAN2B2, PPP1R1A, MAN2B1, MYC, B3GALTL, PFKL, PHKG1, PFKP, PFKM, FBP2, PPP1R3A, PGM2, MAN2A1, RENBP, PGM1, SERP1, MDH1, RBP4, TNF, PFKFB1, SLC37A4, PHKA1, HK1, GALK2, GALK1, TPI1, PPP1R3C, PPP1R3B, HK3, ENO2, ENO3, GALE, GNMT, AGL, ENO1, GPD2, ALDOART1, GPD1, GMDS, PDK3, BPGM, NPY1R, PCK2, MAN1C1, CPT1A, ATF3, PYGM, GCK, PYGL, FABP5, PYGB	2002	169	13588	2.41	1.65E-07	5.89E-09	7.56E-08
GOTERM_BP_FAT	GO:0006006~glucose metabolic process	48	1.69	1.60E-08	RBP4, LDHA, TNF, PHKB, PHKA1, SLC37A4, PGD, G6PDX, PGAM1, HK1, PGAM2, PDHB, ACN9, TPI1, PPP1R3C, PPP1R3B, HK3, PPP1R1A, ENO2, ENO3, GNMT, MYC, AGL, ENO1, GPD2, ALDOART1, GPD1, PFKL, PHKG1, PDK3, PFKP, BPGM, NPY1R, PFKM, FBP2, PPP1R3A, PCK2, CPT1A, PGM2, ATF3, PYGM, GCK, PYGL, PGM1, FABP5, SERP1, MDH1, PYGB	2002	140	13588	2.33	6.50E-05	1.38E-06	2.98E-05
Annotation Cluster 18	Enrichment Score: 5.294982405008022											
Category	Term	Count	%	PValue	Genes	List Total	Pop Hits	Pop Total	Fold Enrichment	Bonferroni	Benjamini	FDR
GOTERM_BP_FAT	GO:0002684~positive regulation of immune system process	87	3.06	1.98E-21	IL27RA, IL18, HPS1, TLR2, TNFSF14, PTPN22, TLR4, TNFSF13, CD1D1, C1QC, PNP, TGFB1, TLR8, B2M, CFP, NOD2, C1RB, MYD88, KLHL6, HLX, TICAM1, LBP, LAG3, SPN, H2-K1, ICOSL, LYN, C4B, CD40, NFAM1, THY1, C1QA, C1QB, CARD11, CD83, CD37, LAT2, CD80, TNFSF13B, ULBP1, LCK, H2-AA, ITGAL, ADORA2B, C3, IL4RA, UNC93B1, KLRK1, CACNB3, IL7R, CD74, ADA, IRAK3, SLC11A1, TAP2, FCER1G, CD4, IL2RG, SH2B2, INPP5D, THBS1, CD27, CLEC2I, CD28, FCER1A, PTPRC, PTPN6, SELP, IL6, CARD9, IKZF1, H2-M3, CFB, H2-Q7, FCGR1, FOXP1, FCGR3, BCL2A1D, CDKN1A, P2RX7, CORO1A, PLCG2, C1RL, CD79B, CLEC7A, H2-DMA, SASH3	2002	206	13588	2.87	8.05E-18	2.01E-18	3.69E-18
GOTERM_BP_FAT	GO:0050865~regulation of cell activation	65	2.29	9.41E-16	IL27RA, IL18, HPS1, TNFSF14, TLR4, TNFSF13, CD1D1, PNP, TGFB1, CASP3, MYD88, HLX, HMOX1, TICAM1, CSK, LAG3, SPN, ICOSL, TNFRSF14, NFAM1, CD40, GAL, THY1, CARD11, CD83, CD80, H2-OA, TNFSF13B, ULBP1, PRAM1, LCK, H2-AA, ITGAL, LST1, NFKBID, ADORA2B, IL4RA, IL7R, CD74, ADA, FCER1G, CD4, IL2RG, INPP5D, CD27, CLEC2I, CD28, FCER1A, PTPRC, SELP, PTPN6, IL6, IKZF1, PLEK, FLT3, CD276, CENPF, BTLA, LAT, CORO1A, CDKN1A, FCGR2B, CD274, H2-DMA, SASH3	2002	156	13588	2.83	3.61E-12	4.51E-13	1.65E-12
GOTERM_BP_FAT	GO:0002694~regulation of leukocyte activation	63	2.22	7.64E-15	IL27RA, IL18, HPS1, TNFSF14, TNFSF13, TLR4, CD1D1, PNP, TGFB1, CASP3, MYD88, HLX, HMOX1, TICAM1, CSK, LAG3, SPN, ICOSL, TNFRSF14, NFAM1, CD40, GAL, THY1, CARD11, CD83, CD80, H2-OA, TNFSF13B, ULBP1, PRAM1, LCK, H2-AA, ITGAL, LST1, NFKBID, ADORA2B, IL4RA, IL7R, CD74, ADA, FCER1G, CD4, IL2RG, INPP5D, CD27, CLEC2I, CD28, FCER1A, PTPRC, PTPN6, IL6, IKZF1, FLT3, CD276, CENPF, BTLA, LAT, CORO1A, CDKN1A, FCGR2B, CD274, H2-DMA, SASH3	2002	154	13588	2.78	3.11E-11	2.39E-12	1.43E-11
GOTERM_BP_FAT	GO:0051249~regulation of lymphocyte activation	58	2.04	2.19E-13	IL27RA, IL18, HPS1, TNFSF14, TNFSF13, TLR4, CD1D1, PNP, TGFB1, CASP3, MYD88, HLX, TICAM1, CSK, LAG3, SPN, ICOSL, TNFRSF14, NFAM1, CD40, GAL, THY1, CARD11, CD83, CD80, H2-OA, TNFSF13B, ULBP1, LCK, H2-AA, ITGAL, LST1, NFKBID, IL4RA, IL7R, ADA, CD74, CD4, IL2RG, INPP5D, CD27, CD28, CLEC2I, PTPRC, PTPN6, IL6, IKZF1, FLT3, CD276, CENPF, BTLA, LAT, CORO1A, CDKN1A, FCGR2B, CD274, H2-DMA, SASH3	2002	144	13588	2.73	8.89E-10	4.94E-11	4.08E-10

GOTERM_BP_FAT	GO:0050867~positive regulation of cell activation	44	1.55	1.12E-11	ITGAL, ADORA2B, IL18, IL4RA, HPS1, TNFSF14, TLR4, TNFSF13, CD1D1, IL7R, PNP, CD74, TGFB1, ADA, MYD88, HLX, TICAM1, FCER1G, CD4, IL2RG, INPP5D, CD27, SPN, CD28, FCER1A, PTPRC, SELP, ICOSL, IL6, IKZF1, PLEK, CD40, THY1, CARD11, CD83, CORO1A, CDKN1A, CD80, TNFSF13B, ULBP1, LCK, H2-AA, H2-DMA, SASH3	2002	101	13588	2.96	4.54E-08	1.75E-09	2.08E-08
GOTERM_BP_FAT	GO:0002696~positive regulation of leukocyte activation	42	1.48	9.60E-11	ITGAL, ADORA2B, IL18, IL4RA, HPS1, TNFSF14, TLR4, TNFSF13, CD1D1, IL7R, PNP, CD74, TGFB1, ADA, MYD88, HLX, TICAM1, FCER1G, CD4, IL2RG, INPP5D, CD27, SPN, CD28, FCER1A, PTPRC, ICOSL, IL6, IKZF1, CD40, THY1, CARD11, CD83, CORO1A, CDKN1A, CD80, TNFSF13B, ULBP1, LCK, H2-AA, H2-DMA, SASH3	2002	99	13588	2.88	3.90E-07	1.30E-08	1.79E-07
GOTERM_BP_FAT	GO:0050863~regulation of T cell activation	43	1.51	6.00E-10	ITGAL, NFKBID, IL18, IL4RA, TNFSF14, CD1D1, IL7R, PNP, CD74, TGFB1, ADA, CASP3, HLX, CD4, IL2RG, CSK, LAG3, CD27, SPN, CD28, CLEC2I, PTPRC, PTPN6, ICOSL, IL6, IKZF1, CD276, CENPF, TNFRSF14, THY1, BTLA, CARD11, LAT, CD83, CORO1A, CD80, H2-OA, TNFSF13B, CD274, LCK, H2-AA, H2-DMA, SASH3	2002	108	13588	2.70	2.44E-06	7.16E-08	1.12E-06
GOTERM_BP_FAT	GO:0051251~positive regulation of lymphocyte activation	39	1.37	1.07E-09	ITGAL, IL18, IL4RA, HPS1, TNFSF14, TNFSF13, TLR4, CD1D1, IL7R, PNP, CD74, TGFB1, ADA, MYD88, HLX, TICAM1, CD4, IL2RG, INPP5D, CD27, SPN, CD28, PTPRC, ICOSL, IL6, IKZF1, CD40, THY1, CARD11, CD83, CORO1A, CDKN1A, CD80, TNFSF13B, ULBP1, LCK, H2-AA, H2-DMA, SASH3	2002	94	13588	2.82	4.33E-06	1.24E-07	1.98E-06
GOTERM_BP_FAT	GO:0002683~negative regulation of immune system process	32	1.13	2.82E-08	ELF1, LST1, IL27RA, NFKBID, IL4RA, IL7R, TGFB1, ADA, CD74, GPX1, IRAK3, NOD2, CASP3, SH2D1B1, HLX, HMOX1, PPP3CB, INPP5D, LAG3, SPN, PTPRC, PTPN6, FLT3, CD276, TNFRSF14, GAL, THY1, BTLA, FCGR2B, CD59B, CD274, KLRB1B	2002	76	13588	2.86	1.14E-04	2.38E-06	5.24E-05
GOTERM_BP_FAT	GO:0050870~positive regulation of T cell activation	29	1.02	4.77E-08	ITGAL, IL18, IL4RA, TNFSF14, CD1D1, IL7R, PNP, ADA, CD74, HLX, CD4, IL2RG, SPN, CD27, CD28, PTPRC, ICOSL, IL6, IKZF1, THY1, CARD11, CD83, CORO1A, TNFSF13B, CD80, LCK, H2-AA, H2-DMA, SASH3	2002	66	13588	2.98	1.94E-04	3.73E-06	8.88E-05
GOTERM_BP_FAT	GO:0045621~positive regulation of lymphocyte differentiation	18	0.63	1.28E-06	PTPRC, IL6, IKZF1, IL4RA, CD1D1, IL7R, PNP, ADA, CD74, CD83, HLX, LCK, H2-AA, IL2RG, INPP5D, H2-DMA, CD27, SASH3	2002	34	13588	3.59	5.19E-03	5.78E-05	2.39E-03
GOTERM_BP_FAT	GO:0050670~regulation of lymphocyte proliferation	30	1.06	1.49E-06	ITGAL, LST1, IL18, TLR4, PNP, TGFB1, ADA, CASP3, MYD88, TICAM1, INPP5D, SPN, CD28, CLEC2I, PTPN6, PTPRC, ICOSL, CD276, TNFRSF14, CD40, GAL, BTLA, CARD11, CDKN1A, CORO1A, CD80, TNFSF13B, FCGR2B, CD274, SASH3	2002	80	13588	2.55	6.05E-03	6.66E-05	2.78E-03
GOTERM_BP_FAT	GO:0032944~regulation of mononuclear cell proliferation	30	1.06	1.49E-06	ITGAL, LST1, IL18, TLR4, PNP, TGFB1, ADA, CASP3, MYD88, TICAM1, INPP5D, SPN, CD28, CLEC2I, PTPN6, PTPRC, ICOSL, CD276, TNFRSF14, CD40, GAL, BTLA, CARD11, CDKN1A, CORO1A, CD80, TNFSF13B, FCGR2B, CD274, SASH3	2002	80	13588	2.55	6.05E-03	6.66E-05	2.78E-03
GOTERM_BP_FAT	GO:0045582~positive regulation of T cell differentiation	17	0.60	2.64E-06	PTPRC, IL6, IKZF1, IL4RA, CD1D1, IL7R, PNP, ADA, CD74, CD83, HLX, LCK, H2-AA, IL2RG, H2-DMA, CD27, SASH3	2002	32	13588	3.61	1.07E-02	1.14E-04	4.91E-03
GOTERM_BP_FAT	GO:0070663~regulation of leukocyte proliferation	30	1.06	2.67E-06	ITGAL, LST1, IL18, TLR4, PNP, TGFB1, ADA, CASP3, MYD88, TICAM1, INPP5D, SPN, CD28, CLEC2I, PTPN6, PTPRC, ICOSL, CD276, TNFRSF14, CD40, GAL, BTLA, CARD11, CDKN1A, CORO1A, CD80, TNFSF13B, FCGR2B, CD274, SASH3	2002	82	13588	2.48	1.08E-02	1.14E-04	4.96E-03
GOTERM_BP_FAT	GO:0045619~regulation of lymphocyte differentiation	24	0.84	3.06E-06	PTPN6, PTPRC, IL6, IKZF1, NFKBID, FLT3, IL4RA, NFAM1, CD1D1, IL7R, PNP, ADA, CD74, CARD11, CD83, H2-OA, HLX, LCK, H2-AA, IL2RG, INPP5D, H2-DMA, CD27, SASH3	2002	58	13588	2.81	1.24E-02	1.30E-04	5.70E-03
GOTERM_BP_FAT	GO:0050864~regulation of B cell activation	22	0.77	1.63E-05	PTPN6, PTPRC, IL27RA, FLT3, TNFSF13, TLR4, NFAM1, CD40, PNP, ADA, TGFB1, BTLA, CARD11, CASP3, CDKN1A, TNFSF13B, FCGR2B, TICAM1, IL2RG, INPP5D, SASH3, CD28	2002	55	13588	2.71	6.42E-02	5.82E-04	3.04E-02
GOTERM_BP_FAT	GO:0045580~regulation of T cell differentiation	20	0.70	2.26E-05	PTPRC, IL6, NFKBID, IKZF1, IL4RA, CD1D1, IL7R, PNP, ADA, CD74, CARD11, CD83, H2-OA, HLX, LCK, H2-AA, IL2RG, H2-DMA, CD27, SASH3	2002	48	13588	2.83	8.76E-02	7.90E-04	4.20E-02

Annotation Cluster 19 Enrichment Score: 5.038943249583482

Category	Term	Count	%	PValue	Genes	List Total	Pop Hits	Pop Total	Fold Enrichment	Bonferroni	Benjamini	FDR
GOTERM_BP_FAT	GO:0002237~response to molecule of bacterial origin	23	0.81	3.94E-07	C5AR1, CARD9, H2-M3, LY96, TLR1, TLR2, TLR4, STAT1, 1100001G20RIK, B2M, IRAK3, SLC11A1, NOD2, P2RX7, MYD88, KCNJ8, TAP2, TICAM1, PLCG2, IL1B, LBP, CD14, PTAFR	2002	49	13588	3.19	1.60E-03	2.19E-05	7.34E-04
GOTERM_BP_FAT	GO:0009617~response to bacterium	45	1.58	1.14E-05	TNF, IL27RA, FGR, TLR1, TLR2, PGLYRP1, TLR4, NLRP1A, SP110, B2M, IRAK3, CD47, TNFRSF1A, SLC11A1, GPX1, NLRC4, NOD2, MYD88, TAP2, TICAM1, BCL3, IL1B, FCER1G, LBP, SPN, H2-K1, LYZ2, CARD9, C5AR1, H2-M3, NCF1, LY96, HCK, MYO1F, STAT1, FCGR1, 1100001G20RIK, P2RX7, CCR5, STAB1, KCNJ8, PLCG2, IRF8, CD14, PTAFR	2002	157	13588	1.95	4.54E-02	4.22E-04	2.13E-02

Annotation Cluster 20 Enrichment Score: 4.8193315425957435

Category	Term	Count	%	PValue	Genes	List Total	Pop Hits	Pop Total	Fold Enrichment	Bonferroni	Benjamini	FDR
GOTERM_MF_FAT	GO:0008009~chemokine activity	18	0.63	6.51E-06	CXCL1, CCL3, CCL2, CXCL5, CXCL9, CCL9, CCL8, PF4, CCL5, CXCL12, CCL4, CCL7, CCL6, CCL12, CCL22, PPBP, CXCL13, CXCL16	1916	38	13288	3.29	8.59E-03	3.60E-04	1.07E-02
GOTERM_MF_FAT	GO:0042379~chemokine receptor binding	18	0.63	1.00E-05	CXCL1, CCL3, CCL2, CXCL5, CXCL9, CCL9, CCL8, PF4, CCL5, CXCL12, CCL4, CCL7, CCL6, CCL12, CCL22, PPBP, CXCL13, CXCL16	1916	39	13288	3.20	1.32E-02	4.91E-04	1.64E-02

Annotation Cluster 21 Enrichment Score: 4.555451893292316

Category	Term	Count	%	PValue	Genes	List Total	Pop Hits	Pop Total	Fold Enrichment	Bonferroni	Benjamini	FDR
GOTERM_BP_FAT	GO:0032943~mononuclear cell proliferation	19	0.67	1.49E-05	PTPRC, ITGAL, ELF4, ITGB2, IL7R, CXCL12, PRKCD, FKBP1B, TGFB1, ITGAM, SLC11A1, DOCK2, CD86, P2RX7, ITGAX, CXCR4, FYN, CD27, HELLS	2002	43	13588	3.00	5.86E-02	5.39E-04	2.77E-02
GOTERM_BP_FAT	GO:0070661~leukocyte proliferation	19	0.67	1.49E-05	PTPRC, ITGAL, ELF4, ITGB2, IL7R, CXCL12, PRKCD, FKBP1B, TGFB1, ITGAM, SLC11A1, DOCK2, CD86, P2RX7, ITGAX, CXCR4, FYN, CD27, HELLS	2002	43	13588	3.00	5.86E-02	5.39E-04	2.77E-02

Annotation Cluster 23 Enrichment Score: 4.213221844013901

Category	Term	Count	%	PValue	Genes	List Total	Pop Hits	Pop Total	Fold Enrichment	Bonferroni	Benjamini	FDR
GOTERM_MF_FAT	GO:0015077~monovalent inorganic cation transmembrane transporter activity	33	1.16	1.79E-07	SLC9A9, ATP5E, SLC36A1, COX7B, COX7C, ATP5G1, UQCRFS1, COX5A, COX5B, UQCRQ, UQCR10, UQCR11, COX6B1, ATP5L, ATP5O, COX6B2, ATP6V0D2, ATP5H, ATP5K, ATP5J, TCIRG1, COX7A2, COX8B, COX7A1, ATP5F1, ATP1A3, ATP1A4, COX6C, ATP6V1A, UQCRH, COX6A2, ATP5C1, UQCRB	1916	87	13288	2.63	2.37E-04	1.97E-05	2.93E-04
GOTERM_MF_FAT	GO:0015078~hydrogen ion transmembrane transporter activity	31	1.09	4.92E-07	SLC9A9, ATP5E, SLC36A1, COX7B, COX7C, ATP5G1, UQCRFS1, COX5A, COX5B, UQCRQ, UQCR10, UQCR11, COX6B1, ATP5L, ATP5O, COX6B2, ATP6V0D2, ATP5H, ATP5K, ATP5J, TCIRG1, COX7A2, COX8B, COX7A1, ATP5F1, COX6C, ATP6V1A, UQCRH, COX6A2, ATP5C1, UQCRB	1916	82	13288	2.62	6.51E-04	4.07E-05	8.05E-04

Annotation Cluster 25 Enrichment Score: 3.6226202982075844

Category	Term	Count	%	PValue	Genes	List Total	Pop Hits	Pop Total	Fold Enrichment	Bonferroni	Benjamini	FDR
GOTERM_BP_FAT	GO:0002274~myeloid leukocyte activation	20	0.70	5.44E-08	FYB, SBNO2, TLR1, MYO1F, TLR4, SFPI1, TGFB1, FCGR3, SLC11A1, LAT, LAT2, FCGR2B, LILRB3, TICAM1, CX3CR1, FCER1G, IRF4, LBP, RHOH, LCP2	2002	35	13588	3.88	2.21E-04	4.17E-06	1.01E-04

Annotation Cluster 26 Enrichment Score: 3.572727502857064

Category	Term	Count	%	PValue	Genes	List Total	Pop Hits	Pop Total	Fold Enrichment	Bonferroni	Benjamini	FDR
GOTERM_BP_FAT	GO:0001819~positive regulation of cytokine production	31	1.09	3.26E-10	TNF, ADORA2B, IL27RA, IL18, TLR2, KLRK1, TLR3, TLR4, TLR7, SLC11A1, NOD2, MYD88, TICAM1, PYCARD, IL1B, FCER1G, BCL3, LBP, CASP1, CARD9, H2-M3, CD40, H2-Q7, IFNAR1, DDX58, CD83, P2RX7, ULBP1, CLEC7A, CD14, SASH3	2002	62	13588	3.39	1.32E-06	4.13E-08	6.06E-07
GOTERM_BP_FAT	GO:0032680~regulation of tumor necrosis factor production	17	0.60	1.19E-07	CARD9, TLR1, TLR2, TLR3, TLR4, FCGR3, IRAK3, NOD2, MYD88, TICAM1, FCER1G, BCL3, LBP, CLEC7A, CD14, SPN, SASH3	2002	27	13588	4.27	4.84E-04	7.82E-06	2.22E-04
GOTERM_BP_FAT	GO:0032760~positive regulation of tumor necrosis factor production	12	0.42	5.29E-07	NOD2, CARD9, MYD88, TICAM1, TLR2, FCER1G, TLR3, TLR4, CLEC7A, LBP, SASH3, CD14	2002	15	13588	5.43	2.15E-03	2.69E-05	9.85E-04
GOTERM_BP_FAT	GO:0032755~positive regulation of interleukin-6 production	13	0.46	7.87E-07	TNF, CARD9, ADORA2B, TLR2, TLR3, TLR4, TLR7, P2RX7, NOD2, MYD88, TICAM1, IL1B, FCER1G	2002	18	13588	4.90	3.19E-03	3.76E-05	1.46E-03
GOTERM_BP_FAT	GO:0032675~regulation of interleukin-6 production	17	0.60	2.64E-06	TNF, CARD9, ADORA2B, TLR1, TLR2, TLR3, TLR4, TLR6, TLR7, IRAK3, P2RX7, NOD2, MYD88, TICAM1, FCER1G, IL1B, INPP5D	2002	32	13588	3.61	1.07E-02	1.14E-04	4.91E-03
GOTERM_BP_FAT	GO:0031349~positive regulation of defense response	23	0.81	8.42E-06	FCER1A, CARD9, ADORA2B, H2-M3, C3, TLR2, UNC93B1, KLRK1, TLR4, FCGR1, TLR8, FCGR3, TNFRSF1A, CD47, NOD2, MYD88, ULBP1, TICAM1, FCER1G, LBP, CLEC7A, LAG3, CD28	2002	57	13588	2.74	3.36E-02	3.14E-04	1.57E-02

Annotation Cluster 28 Enrichment Score: 3.357342543902644

Category	Term	Count	%	PValue	Genes	List Total	Pop Hits	Pop Total	Fold Enrichment	Bonferroni	Benjamini	FDR
GOTERM_MF_FAT	GO:0030695~GTPase regulator activity	91	3.20	6.25E-08	PREX1, IQGAP3, RASGEF1B, IQGAP2, ARHGAP18, ARHGAP15, IQGAP1, RGL1, ARHGAP22, HMHA1, SRPX, DMXL2, RINL, ARHGAP1, ARHGAP11A, DOCK10, DOCK11, ARHGAP9, ARHGEF2, MADD, ARHGEF19, ARHGAP28, RAB3IL1, ARHGAP27, PSD4, ECT2, WAS, THY1, ARHGAP25, ARHGAP30, MAP4K4, ARHGAP33, ACAP1, RIN2, SRGAP3, RIN1, RANGRF, RIN3, SRGAP2, FGD2, ARHGDIG, ARFGAP3, RALGPS2, TBC1D9, SIPA1, GPSM3, CYTH4, MAP4K1, MYO9B, ASAP3, ADAP1, PLEKHG4, PLEKHG3, DOCK2, PLEKHG2, TAGAP, DOCK1, RGS12, GMIP, RASGRP4, RASGRP1, RASAL3, IQSEC3, RASA3, ARHGDIA, FGD3, ARHGDIB, TBC1D2, TBC1D2B, VAV3, TBC1D10C, ABR, TBC1D10A, RGS18, DOCK7, DOCK8, VAV2, DOCK5, RACGAP1, RGS16, VAV1, RGS14, RGS1, ADAP2, RGS2, GRTP1, CHN2, CIT, SH3BP1, ARAP1, BCAR3	1916	361	13288	1.75	8.29E-05	1.18E-05	1.02E-04
GOTERM_MF_FAT	GO:0008047~enzyme activator activity	69	2.43	6.68E-08	GM2A, IQGAP3, ARHGAP18, IQGAP2, LTC4S, ARHGAP15, IQGAP1, HMHA1, ARHGAP22, RINL, APOE, ARHGAP1, ARHGAP11A, ARHGAP9, ARHGEF19, ARHGAP28, ARHGAP27, MMP14, 1100001G20RIK, THY1, ARHGAP25, ARHGAP30, ARHGAP33, ACAP1, RIN2, SRGAP3, RIN1, RIN3, SRGAP2, ARHGDIG, ARFGAP3, CDK5R1, TBC1D9, SIPA1, GPSM3, APOC2, MYO9B, ASAP3, TIMP2, ADAP1, DOCK2, RGS12, GMIP, ALOX5AP, RASAL3, PYCARD, RASA3, ARHGDIA, ARHGDIB, FN1, TBC1D2, GUCA1A, TBC1D2B, TBC1D10C, ABR, TBC1D10A, RGS18, RGS16, RACGAP1, RGS14, RGS1, ADAP2, RGS2, GRTP1, CHN2, APAF1, SH3BP1, ARAP1, MGST2	1916	249	13288	1.92	8.85E-05	1.11E-05	1.09E-04
GOTERM_MF_FAT	GO:0060589~nucleoside-triphosphatase regulator activity	91	3.20	1.40E-07	PREX1, IQGAP3, RASGEF1B, IQGAP2, ARHGAP18, ARHGAP15, IQGAP1, RGL1, ARHGAP22, HMHA1, SRPX, DMXL2, RINL, ARHGAP1, ARHGAP11A, DOCK10, DOCK11, ARHGAP9, ARHGEF2, MADD, ARHGEF19, ARHGAP28, RAB3IL1, ARHGAP27, PSD4, ECT2, WAS, THY1, ARHGAP25, ARHGAP30, MAP4K4, ARHGAP33, ACAP1, RIN2, SRGAP3, RIN1, RANGRF, RIN3, SRGAP2, FGD2, ARHGDIG, ARFGAP3, RALGPS2, TBC1D9, SIPA1, GPSM3, CYTH4, MAP4K1, MYO9B, ASAP3, ADAP1, PLEKHG4, PLEKHG3, DOCK2, PLEKHG2, TAGAP, DOCK1, RGS12, GMIP, RASGRP4, RASGRP1, RASAL3, IQSEC3, RASA3, ARHGDIA, FGD3, ARHGDIB, TBC1D2, TBC1D2B, VAV3, TBC1D10C, ABR, TBC1D10A, RGS18, DOCK7, DOCK8, VAV2, DOCK5, RACGAP1, RGS16, VAV1, RGS14, RGS1, ADAP2, RGS2, GRTP1, CHN2, CIT, SH3BP1, ARAP1, BCAR3	1916	367	13288	1.72	1.86E-04	1.69E-05	2.30E-04
GOTERM_MF_FAT	GO:0005096~GTPase activator activity	55	1.94	5.38E-07	IQGAP3, ARHGAP18, IQGAP2, ARHGAP15, IQGAP1, HMHA1, ARHGAP22, RINL, ARHGAP1, ARHGAP11A, ARHGAP9, ARHGEF19, ARHGAP28, ARHGAP27, THY1, ARHGAP25, ARHGAP30, ARHGAP33, ACAP1, SRGAP3, RIN2, RIN1, RIN3, SRGAP2, ARHGDIG, ARFGAP3, TBC1D9, GPSM3, SIPA1, MYO9B, ASAP3, ADAP1, DOCK2, RGS12, GMIP, RASAL3, RASA3, ARHGDIA, ARHGDIB, TBC1D2, TBC1D2B, TBC1D10C, ABR, TBC1D10A, RGS18, RGS16, RACGAP1, RGS14, ADAP2, RGS1, RGS2, GRTP1, CHN2, SH3BP1, ARAP1	1916	192	13288	1.99	7.13E-04	4.19E-05	8.81E-04

Annotation Cluster 31 Enrichment Score: 3.1451507850941565

Category	Term	Count	%	PValue	Genes	List Total	Pop Hits	Pop Total	Fold Enrichment	Bonferroni	Benjamini	FDR
GOTERM_BP_FAT	GO:0032755~positive regulation of interleukin-6 production	13	0.46	7.87E-07	TNF, CARD9, ADORA2B, TLR2, TLR3, TLR4, TLR7, P2RX7, NOD2, MYD88, TICAM1, IL1B, FCER1G	2002	18	13588	4.90	3.19E-03	3.76E-05	1.46E-03
GOTERM_BP_FAT	GO:0032675~regulation of interleukin-6 production	17	0.60	2.64E-06	TNF, CARD9, ADORA2B, TLR1, TLR2, TLR3, TLR4, TLR6, TLR7, IRAK3, P2RX7, NOD2, MYD88, TICAM1, FCER1G, IL1B, INPP5D	2002	32	13588	3.61	1.07E-02	1.14E-04	4.91E-03

Annotation Cluster 32 Enrichment Score: 3.1389357661073207

Category	Term	Count	%	PValue	Genes	List Total	Pop Hits	Pop Total	Fold Enrichment	Bonferroni	Benjamini	FDR
GOTERM_BP_FAT	GO:0006796~phosphate metabolic process	175	6.16	5.82E-06	RNASEL, PASK, STYX, PKMYT1, TTK, PTPN22, RORC, AURKB, PRKG2, TGFB1, BTK, PRKAR2B, APP, UQCR10, UQCR11, MDFIC, MAP3K8, MLKL, MAP2K6, MATK, PTPRJ, PIK3CG, EGFR, TYRO3, MADD, MYLK4, MYLK2, WNK2, PTPRN, PTPRO, MAP4K4, ACAP1, SLC37A2, DLD, NEK8, ATP5C1, PDGFRA, RIPK3, MAPK7, EIF2AK2, LRRK1, FGFR4, FGR, GNAI2, NEK2, STK10, SSH3, PTK7, ACP5, MAP4K1, CHEK1, EPHB3, SRC, CD74, EPHB2, IRAK4, IRAK3, VRK1, PTPLB, CSF1R, TEC, PTPN7, GPD2, MDFI, FCER1A, PTPN6, GPD1, PTPN18, TAOK3, HGF, EPHA2, EPHA3, IKBKE, EPHA7, DUSP28, P2RX7, PLK4, PLK3, PLK2, PLK1, GRK4, MERTK, ATP5E, IMPA2, NUAK2, SYNJ1, FES, CAMKK1, CAMKK2, CCNE1, SH2D1B1, ATP5L, ATP5O, MASTL, CSK, SIK1, ATP6V0D2, CDK15, ATP5H, CDK14, ATP5K, ATP5J, CDC7, CDK1, LYN, PHKG1, LIMK1, NDUFC2, CDK6, PKDCC, PBK, PRKCD, CDK2, DAPK1, PRKCB, MAST3, OSM, ATP6V1A, CCND1, PPM1E, UQCRH, RIPK1, PPM1H, CFL1, LCK, PPM1N, BUB1B, PPM1L, PPM1M, MELK, UQCRB, CAMK1D, FRK, NDUFB8, PML, PPM1A, ATP5G1, IGSF10, GALK2, GALK1, STAT4, PTK2B, PPP3CB, BUB1, DCLK1, TES, TCIRG1, PTPRC, ALPK1, PTPRE, FLT3, PDK3, HCK, PTPRA, NDUFA7, AXL, ATP5F1, CDKN3, GSG2, CDC25B, DUSP5, CCNB1, RPS6KA6, DUSP4, RPS6KA4, GCK, PTP4A3, RPS6KA1, FYN, GYK, JAK3, DUSP9, CIT, CDK20, DUSP6	2002	866	13588	1.37	2.33E-02	2.36E-04	1.08E-02

GOTERM_BP_FAT	GO:0006793~phosphorus metabolic process	175	6.16	5.82E-06	RNASEL, PASK, STYX, PKMYT1, TTK, PTPN22, RORC, AURKB, PRKG2, TGFB1, BTK, PRKAR2B, APP, UQCR10, UQCR11, MDFIC, MAP3K8, MLKL, MAP2K6, MATK, PTPRJ, PIK3CG, EGFR, TYRO3, MADD, MYLK4, MYLK2, WNK2, PTPRN, PTPRO, MAP4K4, ACAP1, SLC37A2, DLD, NEK8, ATP5C1, PDGFRA, RIPK3, MAPK7, EIF2AK2, LRRK1, FGFR4, FGR, GNAI2, NEK2, STK10, SSH3, PTK7, ACP5, MAP4K1, CHEK1, EPHB3, SRC, CD74, EPHB2, IRAK4, IRAK3, VRK1, PTPLB, CSF1R, TEC, PTPN7, GPD2, MDFI, FCER1A, PTPN6, GPD1, PTPN18, TAOK3, HGF, EPHA2, EPHA3, IKBKE, EPHA7, DUSP28, P2RX7, PLK4, PLK3, PLK2, PLK1, GRK4, MERTK, ATP5E, IMPA2, NUAK2, SYNJ1, FES, CAMKK1, CAMKK2, CCNE1, SH2D1B1, ATP5L, ATP5O, MASTL, CSK, SIK1, ATP6V0D2, CDK15, ATP5H, CDK14, ATP5K, ATP5J, CDC7, CDK1, LYN, PHKG1, LIMK1, NDUFC2, CDK6, PKDCC, PBK, PRKCD, CDK2, DAPK1, PRKCB, MAST3, OSM, ATP6V1A, CCND1, PPM1E, UQCRH, RIPK1, PPM1H, CFL1, LCK, PPM1N, BUB1B, PPM1L, PPM1M, MELK, UQCRB, CAMK1D, FRK, NDUFB8, PML, PPM1A, ATP5G1, IGSF10, GALK2, GALK1, STAT4, PTK2B, PPP3CB, BUB1, DCLK1, TES, TCIRG1, PTPRC, ALPK1, PTPRE, FLT3, PDK3, HCK, PTPRA, NDUFA7, AXL, ATP5F1, CDKN3, GSG2, CDC25B, DUSP5, CCNB1, RPS6KA6, DUSP4, RPS6KA4, GCK, PTP4A3, RPS6KA1, FYN, GYK, JAK3, DUSP9, CIT, CDK20, DUSP6	2002	866	13588	1.37	2.33E-02	2.36E-04	1.08E-02
Annotation Cluster 35	Enrichment Score: 2.5545766691173992											
Category	Term	Count	%	PValue	Genes	List Total	Pop Hits	Pop Total	Fold Enrichment	Bonferroni	Benjamini	FDR
GOTERM_BP_FAT	GO:0033559~unsaturated fatty acid metabolic process	19	0.67	6.23E-07	FCER1A, TBXAS1, PTGS2, NCF1, PDPN, FADS1, EPHX2, LTC4S, CD74, GGT5, TNFRSF1A, PTGIS, PTGES, ALOX5AP, ALOX5, HPGD, RNPEP, HPGDS, MGST2	2002	36	13588	3.58	2.53E-03	3.05E-05	1.16E-03
GOTERM_BP_FAT	GO:0006690~icosanoid metabolic process	17	0.60	7.22E-06	FCER1A, TBXAS1, PTGS2, NCF1, PDPN, LTC4S, CD74, GGT5, TNFRSF1A, PTGIS, PTGES, ALOX5AP, ALOX5, HPGD, RNPEP, HPGDS, MGST2	2002	34	13588	3.39	2.89E-02	2.76E-04	1.34E-02
Annotation Cluster 40	Enrichment Score: 2.3291168681773002											
Category	Term	Count	%	PValue	Genes	List Total	Pop Hits	Pop Total	Fold Enrichment	Bonferroni	Benjamini	FDR
GOTERM_BP_FAT	GO:0050777~negative regulation of immune response	14	0.49	2.33E-05	PTPRC, PTPN6, IL27RA, IL7R, TGFB1, GPX1, IRAK3, SH2D1B1, FCGR2B, CD59B, PPP3CB, INPP5D, KLRB1B, SPN	2002	26	13588	3.65	9.02E-02	8.00E-04	4.33E-02
Annotation Cluster 43	Enrichment Score: 2.1580019024363786											
Category	Term	Count	%	PValue	Genes	List Total	Pop Hits	Pop Total	Fold Enrichment	Bonferroni	Benjamini	FDR
GOTERM_BP_FAT	GO:0031341~regulation of cell killing	16	0.56	5.44E-06	H2-K1, PTPRC, H2-M3, KLRK1, IL7R, CD1D1, PNP, H2-Q7, B2M, P2RX7, SH2D1B1, TAP2, ULBP1, PPP3CB, KLRB1B, LAG3	2002	30	13588	3.62	2.19E-02	2.23E-04	1.01E-02
GOTERM_BP_FAT	GO:0001910~regulation of leukocyte mediated cytotoxicity	16	0.56	5.44E-06	H2-K1, PTPRC, H2-M3, KLRK1, IL7R, CD1D1, PNP, H2-Q7, B2M, P2RX7, SH2D1B1, TAP2, ULBP1, PPP3CB, KLRB1B, LAG3	2002	30	13588	3.62	2.19E-02	2.23E-04	1.01E-02
Annotation Cluster 44	Enrichment Score: 2.143499635516042											
Category	Term	Count	%	PValue	Genes	List Total	Pop Hits	Pop Total	Fold Enrichment	Bonferroni	Benjamini	FDR
GOTERM_BP_FAT	GO:0032101~regulation of response to external stimulus	33	1.16	1.87E-05	SBNO2, ADORA2B, IL16, C3, TLR4, CXCL12, ADA, GPX1, TNFRSF1A, CD47, NOD2, TNFRSF1B, AOAH, APOH, FCER1G, LBP, THBS1, SPN, CD28, ZFP36, FCER1A, SELP, CLN3, PLEK, CD276, AGER, FCGR1, FCGR3, ANXA2, CD59B, FCGR2B, NPY, FABP7	2002	103	13588	2.17	7.33E-02	6.62E-04	3.49E-02
Annotation Cluster 50	Enrichment Score: 2.0226420719661573											
Category	Term	Count	%	PValue	Genes	List Total	Pop Hits	Pop Total	Fold Enrichment	Bonferroni	Benjamini	FDR
GOTERM_MF_FAT	GO:0015077~monovalent inorganic cation transmembrane transporter activity	33	1.16	1.79E-07	SLC9A9, ATP5E, SLC36A1, COX7B, COX7C, ATP5G1, UQCRFS1, COX5A, COX5B, UQCRQ, UQCR10, UQCR11, COX6B1, ATP5L, ATP5O, COX6B2, ATP6V0D2, ATP5H, ATP5K, ATP5J, TCIRG1, COX7A2, COX8B, COX7A1, ATP5F1, ATP1A3, ATP1A4, COX6C, ATP6V1A, UQCRH, COX6A2, ATP5C1, UQCRB	1916	87	13288	2.63	2.37E-04	1.97E-05	2.93E-04
GOTERM_MF_FAT	GO:0015078~hydrogen ion transmembrane transporter activity	31	1.09	4.92E-07	SLC9A9, ATP5E, SLC36A1, COX7B, COX7C, ATP5G1, UQCRFS1, COX5A, COX5B, UQCRQ, UQCR10, UQCR11, COX6B1, ATP5L, ATP5O, COX6B2, ATP6V0D2, ATP5H, ATP5K, ATP5J, TCIRG1, COX7A2, COX8B, COX7A1, ATP5F1, COX6C, ATP6V1A, UQCRH, COX6A2, ATP5C1, UQCRB	1916	82	13288	2.62	6.51E-04	4.07E-05	8.05E-04

Table S1.F GO Terms upregulated 168 hours post injury

Annotation Cluster 1 Enrichment Score: 14.556398086357477

Category	Term	Count	%	PValue	Genes	List Total	Pop Hits	Pop Total	Fold Enrichment	Bonferroni	Benjamini	FDR
GOTERM_BP_FAT	GO:0006955~immune response	49	14.16	6.60E-23	LY86, TLR2, CD1D1, C1QC, TLR7, IL10, CFP, OASL2, AF251705, NCF1, C4B, H2-DMB1, C1QA, C1QB, IL18BP, CCR5, LILRB3, CX3CR1, LILRB4, CCR2, OAS1A, IL12B, OAS1G, CD300LB, CD300LD, CCL2, LST1, CCL9, CCL8, OAS2, CCL7, SLC11A1, GP49A, FCER1G, CD4, CD7, TLR13, MYO1F, CD300C, FCGR1, CD180, CCL17, FCGR3, CCL12, CD300A, PLCG2, IRF8, CLEC7A, XCL1, CD14	249	471	13588	5.68	1.03E-19	1.03E-19	1.10E-19
GOTERM_BP_FAT	GO:0006952~defense response	37	10.69	5.48E-14	CCL2, FGR, LY86, TLR2, CCL8, ITGB2, CD1D1, TLR7, C1QC, CCL7, IL10, CFP, SLC11A1, PYCARD, FCER1G, LYZ2, C4B, NCF1, HCK, CAMP, TLR13, SAA3, MYO1F, COTL1, FCGR1, CD180, FCGR3, CCL17, C1QA, C1QB, CCL12, CCR5, IRF8, CCR2, CLEC7A, CD14, ORM2	249	448	13588	4.51	8.55E-11	4.28E-11	9.15E-11
GOTERM_BP_FAT	GO:0006954~inflammatory response	26	7.51	4.19E-13	CCL2, LY86, TLR2, CCL8, ITGB2, C1QC, TLR7, CCL7, CFP, SLC11A1, PYCARD, NCF1, C4B, TLR13, SAA3, FCGR1, CD180, FCGR3, C1QA, CCL12, C1QB, CCR5, CCR2, CLEC7A, ORM2, CD14	249	225	13588	6.31	6.53E-10	2.18E-10	6.99E-10
GOTERM_BP_FAT	GO:0009611~response to wounding	29	8.38	3.92E-11	CCL2, LY86, TLR2, CCL8, ITGB2, C1QC, TLR7, CCL7, CFP, SLC11A1, PYCARD, TFPI2, PLEK, GATM, NCF1, C4B, TLR13, SAA3, FCGR1, CD180, FCGR3, C1QA, C1QB, CCL12, CCR5, CCR2, CLEC7A, ORM2, CD14	249	347	13588	4.56	6.12E-08	1.53E-08	6.55E-08

Annotation Cluster 2 Enrichment Score: 5.491123660755224

Category	Term	Count	%	PValue	Genes	List Total	Pop Hits	Pop Total	Fold Enrichment	Bonferroni	Benjamini	FDR
GOTERM_BP_FAT	GO:0042742~defense response to bacterium	13	3.76	6.20E-07	LYZ2, FGR, NCF1, CAMP, HCK, TLR2, MYO1F, FCGR1, IL10, SLC11A1, CCR5, IRF8, FCER1G	249	108	13588	6.57	9.66E-04	6.90E-05	1.03E-03
GOTERM_BP_FAT	GO:0009617~response to bacterium	15	4.34	1.08E-06	LYZ2, FGR, NCF1, CAMP, HCK, TLR2, MYO1F, FCGR1, IL10, SLC11A1, CCR5, IRF8, PLCG2, FCER1G, CD14	249	157	13588	5.21	1.69E-03	1.13E-04	1.81E-03

Annotation Cluster 3 Enrichment Score: 4.990727463947588

Category	Term	Count	%	PValue	Genes	List Total	Pop Hits	Pop Total	Fold Enrichment	Bonferroni	Benjamini	FDR
GOTERM_BP_FAT	GO:0001819~positive regulation of cytokine production	11	3.18	1.60E-07	CD83, SLC11A1, KLRE1, PYCARD, TLR2, FCER1G, CLEC7A, CASP1, TLR7, SASH3, CD14	249	62	13588	9.68	2.49E-04	2.27E-05	2.67E-04
GOTERM_BP_FAT	GO:0001817~regulation of cytokine production	15	4.34	2.44E-07	TLR2, CD1D1, TLR7, IL10, FCGR3, SLC11A1, CD83, HMOX1, KLRE1, PYCARD, FCER1G, CLEC7A, CASP1, SASH3, CD14	249	139	13588	5.89	3.80E-04	3.16E-05	4.07E-04

Annotation Cluster 4 Enrichment Score: 4.97611947115325

Category	Term	Count	%	PValue	Genes	List Total	Pop Hits	Pop Total	Fold Enrichment	Bonferroni	Benjamini	FDR
GOTERM_BP_FAT	GO:0042330~taxis	15	4.34	1.08E-08	C3AR1, CCL2, CCL9, CCL8, ITGB2, CCL7, CCL17, FCGR3, CCL12, CORO1A, RAC2, CX3CR1, FCER1G, XCR1, XCL1	249	109	13588	7.51	1.69E-05	1.88E-06	1.81E-05
GOTERM_BP_FAT	GO:0006935~chemotaxis	15	4.34	1.08E-08	C3AR1, CCL2, CCL9, CCL8, ITGB2, CCL7, CCL17, FCGR3, CCL12, CORO1A, RAC2, CX3CR1, FCER1G, XCR1, XCL1	249	109	13588	7.51	1.69E-05	1.88E-06	1.81E-05
GOTERM_MF_FAT	GO:0008009~chemokine activity	7	2.02	2.84E-05	CCL12, CCL2, CCL9, CCL8, XCL1, CCL7, CCL17	214	38	13288	11.44	9.14E-03	4.58E-03	3.84E-02
GOTERM_MF_FAT	GO:0042379~chemokine receptor binding	7	2.02	3.32E-05	CCL12, CCL2, CCL9, CCL8, XCL1, CCL7, CCL17	214	39	13288	11.14	1.07E-02	3.56E-03	4.48E-02

Annotation Cluster 5 Enrichment Score: 4.734364155773175

Category	Term	Count	%	PValue	Genes	List Total	Pop Hits	Pop Total	Fold Enrichment	Bonferroni	Benjamini	FDR
GOTERM_BP_FAT	GO:0001775~cell activation	24	6.94	1.31E-10	FYB, LST1, PLEK, MYO1F, RORC, ITGB2, SFPI1, CD1D1, WAS, FCGR3, CD48, SLC11A1, BCL2A1D, CD86, LILRB3, PLCG2, CX3CR1, KLRE1, FCER1G, CD4, IL12B, HELLS, RHOH, LCP2	249	246	13588	5.32	2.04E-07	4.08E-08	2.19E-07
GOTERM_BP_FAT	GO:0045321~leukocyte activation	22	6.36	5.46E-10	FYB, MYO1F, RORC, ITGB2, SFPI1, CD1D1, WAS, FCGR3, CD48, SLC11A1, BCL2A1D, CD86, LILRB3, PLCG2, CX3CR1, KLRE1, FCER1G, CD4, IL12B, HELLS, RHOH, LCP2	249	219	13588	5.48	8.52E-07	1.42E-07	9.12E-07
GOTERM_BP_FAT	GO:0002274~myeloid leukocyte activation	10	2.89	9.07E-09	FYB, SLC11A1, LILRB3, CX3CR1, MYO1F, FCER1G, SFPI1, LCP2, RHOH, FCGR3	249	35	13588	15.59	1.41E-05	1.77E-06	1.51E-05
GOTERM_BP_FAT	GO:0046649~lymphocyte activation	15	4.34	1.09E-05	RORC, ITGB2, SFPI1, CD1D1, WAS, CD48, SLC11A1, CD86, BCL2A1D, PLCG2, KLRE1, CD4, IL12B, HELLS, RHOH	249	191	13588	4.29	1.68E-02	8.46E-04	1.81E-02

Annotation Cluster 6 Enrichment Score: 4.102642809152643

Category	Term	Count	%	PValue	Genes	List Total	Pop Hits	Pop Total	Fold Enrichment	Bonferroni	Benjamini	FDR
GOTERM_BP_FAT	GO:0002684~positive regulation of immune system process	21	6.07	1.15E-09	C4B, TLR2, CD1D1, C1QC, FCGR1, FCGR3, CFP, C1QA, C1QB, SLC11A1, CD83, BCL2A1D, CORO1A, PLCG2, KLRE1, FCER1G, CD4, IL2RG, IL12B, CLEC7A, SASH3	249	206	13588	5.56	1.79E-06	2.56E-07	1.92E-06
GOTERM_BP_FAT	GO:0050778~positive regulation of immune response	16	4.62	2.64E-08	C4B, TLR2, CD1D1, C1QC, FCGR1, FCGR3, C1QA, CFP, C1QB, SLC11A1, BCL2A1D, PLCG2, KLRE1, FCER1G, CLEC7A, SASH3	249	136	13588	6.42	4.11E-05	4.11E-06	4.40E-05
GOTERM_BP_FAT	GO:0045087~innate immune response	13	3.76	5.60E-07	C4B, TLR13, TLR2, CD1D1, C1QC, FCGR1, TLR7, CD180, C1QA, CFP, SLC11A1, C1QB, CLEC7A	249	107	13588	6.63	8.73E-04	6.72E-05	9.35E-04
GOTERM_BP_FAT	GO:0048584~positive regulation of response to stimulus	16	4.62	1.60E-06	C4B, TLR2, CD1D1, C1QC, FCGR1, FCGR3, C1QA, CFP, C1QB, SLC11A1, BCL2A1D, PLCG2, KLRE1, FCER1G, CLEC7A, SASH3	249	186	13588	4.69	2.50E-03	1.56E-04	2.68E-03
GOTERM_BP_FAT	GO:0002252~immune effector process	13	3.76	3.24E-06	NCF1, C4B, MYO1F, C1QC, FCGR1, TLR7, FCGR3, C1QA, CFP, SLC11A1, C1QB, LILRB3, FCER1G	249	126	13588	5.63	5.04E-03	2.97E-04	5.41E-03
GOTERM_BP_FAT	GO:0002253~activation of immune response	11	3.18	3.59E-06	C1QA, CFP, C1QB, BCL2A1D, C4B, PLCG2, KLRE1, TLR2, FCER1G, CLEC7A, C1QC	249	86	13588	6.98	5.58E-03	3.11E-04	5.99E-03
GOTERM_BP_FAT	GO:0002443~leukocyte mediated immunity	11	3.18	4.91E-06	C1QA, C1QB, SLC11A1, NCF1, C4B, LILRB3, MYO1F, FCER1G, C1QC, FCGR1, FCGR3	249	89	13588	6.74	7.63E-03	4.03E-04	8.20E-03
GOTERM_BP_FAT	GO:0002250~adaptive immune response	10	2.89	2.17E-05	C1QA, C1QB, SLC11A1, IL18BP, C4B, LILRB3, FCER1G, C1QC, FCGR1, FCGR3	249	84	13588	6.50	3.33E-02	1.61E-03	3.63E-02
GOTERM_BP_FAT	GO:0002460~adaptive immune response based on somatic recombination of immune receptors built fr	10	2.89	2.17E-05	C1QA, C1QB, SLC11A1, IL18BP, C4B, LILRB3, FCER1G, C1QC, FCGR1, FCGR3	249	84	13588	6.50	3.33E-02	1.61E-03	3.63E-02

Annotation Cluster 7 Enrichment Score: 3.0962877308218895

Category	Term	Count	%	PValue	Genes	List Total	Pop Hits	Pop Total	Fold Enrichment	Bonferroni	Benjamini	FDR
GOTERM_BP_FAT	GO:0050778~positive regulation of immune response	16	4.62	2.64E-08	C4B, TLR2, CD1D1, C1QC, FCGR1, FCGR3, C1QA, CFP, C1QB, SLC11A1, BCL2A1D, PLCG2, KLRE1, FCER1G, CLEC7A, SASH3	249	136	13588	6.42	4.11E-05	4.11E-06	4.40E-05
GOTERM_BP_FAT	GO:0048584~positive regulation of response to stimulus	16	4.62	1.60E-06	C4B, TLR2, CD1D1, C1QC, FCGR1, FCGR3, C1QA, CFP, C1QB, SLC11A1, BCL2A1D, PLCG2, KLRE1, FCER1G, CLEC7A, SASH3	249	186	13588	4.69	2.50E-03	1.56E-04	2.68E-03

Annotation Cluster 12 Enrichment Score: 2.1332794229095495

Category	Term	Count	%	PValue	Genes	List Total	Pop Hits	Pop Total	Fold Enrichment	Bonferroni	Benjamini	FDR
GOTERM_BP_FAT	GO:0006909~phagocytosis	8	2.31	2.77E-05	SLC11A1, HCK, IRF8, FCER1G, CLEC7A, MEGF10, FCGR1, FCGR3	249	49	13588	8.91	4.23E-02	1.96E-03	4.62E-02

Annotation Cluster 13 Enrichment Score: 2.048358806290177

Category	Term	Count	%	PValue	Genes	List Total	Pop Hits	Pop Total	Fold Enrichment	Bonferroni	Benjamini	FDR
GOTERM_MF_FAT	GO:0019955~cytokine binding	11	3.18	1.38E-06	IL18BP, CCR5, CSF2RB2, IL10RA, CCR2, CX3CR1, CSF2RB, IL2RG, IL12B, XCR1, CSF2RA	214	88	13288	7.76	4.45E-04	4.45E-04	1.86E-03

Table S1.G GO Terms upregulated 336 hours post injury

Annotation Cluster 1 Enrichment Score: 14.556398086357477

Category	Term	Count	%	PValue	Genes	List Total	Pop Hits	Pop Total	Fold Enrichment	Bonferroni	Benjamini	FDR
GOTERM_BP_FAT	GO:0006955~immune response	49	14.16	6.60E-23	LY86, TLR2, CD1D1, C1QC, TLR7, IL10, CFP, OASL2, AF251705, NCF1, C4B, H2-DMB1, C1QA, C1QB, IL18BP, CCR5, LILRB3, CX3CR1, LILRB4, CCR2, OAS1A, IL12B, OAS1G, CD300LB, CD300LD, CCL2, LST1, CCL9, CCL8, OAS2, CCL7, SLC11A1, GP49A, FCER1G, CD4, CD7, TLR13, MYO1F, CD300C, FCGR1, CD180, CCL17, FCGR3, CCL12, CD300A, PLCG2, IRF8, CLEC7A, XCL1, CD14	249	471	13588	5.68	1.03E-19	1.03E-19	1.10E-19
GOTERM_BP_FAT	GO:0006952~defense response	37	10.69	5.48E-14	CCL2, FGR, LY86, TLR2, CCL8, ITGB2, CD1D1, TLR7, C1QC, CCL7, IL10, CFP, SLC11A1, PYCARD, FCER1G, LYZ2, C4B, NCF1, HCK, CAMP, TLR13, SAA3, MYO1F, COTL1, FCGR1, CD180, FCGR3, CCL17, C1QA, C1QB, CCL12, CCR5, IRF8, CCR2, CLEC7A, CD14, ORM2	249	448	13588	4.51	8.55E-11	4.28E-11	9.15E-11
GOTERM_BP_FAT	GO:0006954~inflammatory response	26	7.51	4.19E-13	CCL2, LY86, TLR2, CCL8, ITGB2, C1QC, TLR7, CCL7, CFP, SLC11A1, PYCARD, NCF1, C4B, TLR13, SAA3, FCGR1, CD180, FCGR3, C1QA, CCL12, C1QB, CCR5, CCR2, CLEC7A, ORM2, CD14	249	225	13588	6.31	6.53E-10	2.18E-10	6.99E-10
GOTERM_BP_FAT	GO:0009611~response to wounding	29	8.38	3.92E-11	CCL2, LY86, TLR2, CCL8, ITGB2, C1QC, TLR7, CCL7, CFP, SLC11A1, PYCARD, TFPI2, PLEK, GATM, NCF1, C4B, TLR13, SAA3, FCGR1, CD180, FCGR3, C1QA, C1QB, CCL12, CCR5, CCR2, CLEC7A, ORM2, CD14	249	347	13588	4.56	6.12E-08	1.53E-08	6.55E-08

Annotation Cluster 2 Enrichment Score: 5.491123660755224

Category	Term	Count	%	PValue	Genes	List Total	Pop Hits	Pop Total	Fold Enrichment	Bonferroni	Benjamini	FDR
GOTERM_BP_FAT	GO:0042742~defense response to bacterium	13	3.76	6.20E-07	LYZ2, FGR, NCF1, CAMP, HCK, TLR2, MYO1F, FCGR1, IL10, SLC11A1, CCR5, IRF8, FCER1G	249	108	13588	6.57	9.66E-04	6.90E-05	1.03E-03
GOTERM_BP_FAT	GO:0009617~response to bacterium	15	4.34	1.08E-06	LYZ2, FGR, NCF1, CAMP, HCK, TLR2, MYO1F, FCGR1, IL10, SLC11A1, CCR5, IRF8, PLCG2, FCER1G, CD14	249	157	13588	5.21	1.69E-03	1.13E-04	1.81E-03

Annotation Cluster 3 Enrichment Score: 4.990727463947588

Category	Term	Count	%	PValue	Genes	List Total	Pop Hits	Pop Total	Fold Enrichment	Bonferroni	Benjamini	FDR
GOTERM_BP_FAT	GO:0001819~positive regulation of cytokine production	11	3.18	1.60E-07	CD83, SLC11A1, KLRE1, PYCARD, TLR2, FCER1G, CLEC7A, CASP1, TLR7, SASH3, CD14	249	62	13588	9.68	2.49E-04	2.27E-05	2.67E-04
GOTERM_BP_FAT	GO:0001817~regulation of cytokine production	15	4.34	2.44E-07	TLR2, CD1D1, TLR7, IL10, FCGR3, SLC11A1, CD83, HMOX1, KLRE1, PYCARD, FCER1G, CLEC7A, CASP1, SASH3, CD14	249	139	13588	5.89	3.80E-04	3.16E-05	4.07E-04

Annotation Cluster 4 Enrichment Score: 4.97611947115325

Category	Term	Count	%	PValue	Genes	List Total	Pop Hits	Pop Total	Fold Enrichment	Bonferroni	Benjamini	FDR
GOTERM_BP_FAT	GO:0042330~taxis	15	4.34	1.08E-08	C3AR1, CCL2, CCL9, CCL8, ITGB2, CCL7, CCL17, FCGR3, CCL12, CORO1A, RAC2, CX3CR1, FCER1G, XCR1, XCL1	249	109	13588	7.51	1.69E-05	1.88E-06	1.81E-05
GOTERM_BP_FAT	GO:0006935~chemotaxis	15	4.34	1.08E-08	C3AR1, CCL2, CCL9, CCL8, ITGB2, CCL7, CCL17, FCGR3, CCL12, CORO1A, RAC2, CX3CR1, FCER1G, XCR1, XCL1	249	109	13588	7.51	1.69E-05	1.88E-06	1.81E-05
GOTERM_MF_FAT	GO:0008009~chemokine activity	7	2.02	2.84E-05	CCL12, CCL2, CCL9, CCL8, XCL1, CCL7, CCL17	214	38	13288	11.44	9.14E-03	4.58E-03	3.84E-02
GOTERM_MF_FAT	GO:0042379~chemokine receptor binding	7	2.02	3.32E-05	CCL12, CCL2, CCL9, CCL8, XCL1, CCL7, CCL17	214	39	13288	11.14	1.07E-02	3.56E-03	4.48E-02

Annotation Cluster 5 Enrichment Score: 4.734364155773175

Category	Term	Count	%	PValue	Genes	List Total	Pop Hits	Pop Total	Fold Enrichment	Bonferroni	Benjamini	FDR
GOTERM_BP_FAT	GO:0001775~cell activation	24	6.94	1.31E-10	FYB, LST1, PLEK, MYO1F, RORC, ITGB2, SFPI1, CD1D1, WAS, FCGR3, CD48, SLC11A1, BCL2A1D, CD86, LILRB3, PLCG2, CX3CR1, KLRE1, FCER1G, CD4, IL12B, HELLS, RHOH, LCP2	249	246	13588	5.32	2.04E-07	4.08E-08	2.19E-07
GOTERM_BP_FAT	GO:0045321~leukocyte activation	22	6.36	5.46E-10	FYB, MYO1F, RORC, ITGB2, SFPI1, CD1D1, WAS, FCGR3, CD48, SLC11A1, BCL2A1D, CD86, LILRB3, PLCG2, CX3CR1, KLRE1, FCER1G, CD4, IL12B, HELLS, RHOH, LCP2	249	219	13588	5.48	8.52E-07	1.42E-07	9.12E-07
GOTERM_BP_FAT	GO:0002274~myeloid leukocyte activation	10	2.89	9.07E-09	FYB, SLC11A1, LILRB3, CX3CR1, MYO1F, FCER1G, SFPI1, LCP2, RHOH, FCGR3	249	35	13588	15.59	1.41E-05	1.77E-06	1.51E-05
GOTERM_BP_FAT	GO:0046649~lymphocyte activation	15	4.34	1.09E-05	RORC, ITGB2, SFPI1, CD1D1, WAS, CD48, SLC11A1, CD86, BCL2A1D, PLCG2, KLRE1, CD4, IL12B, HELLS, RHOH	249	191	13588	4.29	1.68E-02	8.46E-04	1.81E-02

Annotation Cluster 6 Enrichment Score: 4.102642809152643

Category	Term	Count	%	PValue	Genes	List Total	Pop Hits	Pop Total	Fold Enrichment	Bonferroni	Benjamini	FDR
GOTERM_BP_FAT	GO:0002684~positive regulation of immune system process	21	6.07	1.15E-09	C4B, TLR2, CD1D1, C1QC, FCGR1, FCGR3, CFP, C1QA, C1QB, SLC11A1, CD83, BCL2A1D, CORO1A, PLCG2, KLRE1, FCER1G, CD4, IL2RG, IL12B, CLEC7A, SASH3	249	206	13588	5.56	1.79E-06	2.56E-07	1.92E-06
GOTERM_BP_FAT	GO:0050778~positive regulation of immune response	16	4.62	2.64E-08	C4B, TLR2, CD1D1, C1QC, FCGR1, FCGR3, C1QA, CFP, C1QB, SLC11A1, BCL2A1D, PLCG2, KLRE1, FCER1G, CLEC7A, SASH3	249	136	13588	6.42	4.11E-05	4.11E-06	4.40E-05
GOTERM_BP_FAT	GO:0045087~innate immune response	13	3.76	5.60E-07	C4B, TLR13, TLR2, CD1D1, C1QC, FCGR1, TLR7, CD180, C1QA, CFP, SLC11A1, C1QB, CLEC7A	249	107	13588	6.63	8.73E-04	6.72E-05	9.35E-04
GOTERM_BP_FAT	GO:0048584~positive regulation of response to stimulus	16	4.62	1.60E-06	C4B, TLR2, CD1D1, C1QC, FCGR1, FCGR3, C1QA, CFP, C1QB, SLC11A1, BCL2A1D, PLCG2, KLRE1, FCER1G, CLEC7A, SASH3	249	186	13588	4.69	2.50E-03	1.56E-04	2.68E-03
GOTERM_BP_FAT	GO:0002252~immune effector process	13	3.76	3.24E-06	NCF1, C4B, MYO1F, C1QC, FCGR1, TLR7, FCGR3, C1QA, CFP, SLC11A1, C1QB, LILRB3, FCER1G	249	126	13588	5.63	5.04E-03	2.97E-04	5.41E-03
GOTERM_BP_FAT	GO:0002253~activation of immune response	11	3.18	3.59E-06	C1QA, CFP, C1QB, BCL2A1D, C4B, PLCG2, KLRE1, TLR2, FCER1G, CLEC7A, C1QC	249	86	13588	6.98	5.58E-03	3.11E-04	5.99E-03
GOTERM_BP_FAT	GO:0002443~leukocyte mediated immunity	11	3.18	4.91E-06	C1QA, C1QB, SLC11A1, NCF1, C4B, LILRB3, MYO1F, FCER1G, C1QC, FCGR1, FCGR3	249	89	13588	6.74	7.63E-03	4.03E-04	8.20E-03
GOTERM_BP_FAT	GO:0002250~adaptive immune response	10	2.89	2.17E-05	C1QA, C1QB, SLC11A1, IL18BP, C4B, LILRB3, FCER1G, C1QC, FCGR1, FCGR3	249	84	13588	6.50	3.33E-02	1.61E-03	3.63E-02
GOTERM_BP_FAT	GO:0002460~adaptive immune response based on somatic recombination of immune receptors built fr	10	2.89	2.17E-05	C1QA, C1QB, SLC11A1, IL18BP, C4B, LILRB3, FCER1G, C1QC, FCGR1, FCGR3	249	84	13588	6.50	3.33E-02	1.61E-03	3.63E-02

Annotation Cluster 7 Enrichment Score: 3.0962877308218895

Category	Term	Count	%	PValue	Genes	List Total	Pop Hits	Pop Total	Fold Enrichment	Bonferroni	Benjamini	FDR
GOTERM_BP_FAT	GO:0050778~positive regulation of immune response	16	4.62	2.64E-08	C4B, TLR2, CD1D1, C1QC, FCGR1, FCGR3, C1QA, CFP, C1QB, SLC11A1, BCL2A1D, PLCG2, KLRE1, FCER1G, CLEC7A, SASH3	249	136	13588	6.42	4.11E-05	4.11E-06	4.40E-05
GOTERM_BP_FAT	GO:0048584~positive regulation of response to stimulus	16	4.62	1.60E-06	C4B, TLR2, CD1D1, C1QC, FCGR1, FCGR3, C1QA, CFP, C1QB, SLC11A1, BCL2A1D, PLCG2, KLRE1, FCER1G, CLEC7A, SASH3	249	186	13588	4.69	2.50E-03	1.56E-04	2.68E-03

Annotation Cluster 12 Enrichment Score: 2.1332794229095495

Category	Term	Count	%	PValue	Genes	List Total	Pop Hits	Pop Total	Fold Enrichment	Bonferroni	Benjamini	FDR
GOTERM_BP_FAT	GO:0006909~phagocytosis	8	2.31	2.77E-05	SLC11A1, HCK, IRF8, FCER1G, CLEC7A, MEGF10, FCGR1, FCGR3	249	49	13588	8.91	4.23E-02	1.96E-03	4.62E-02

Annotation Cluster 13 Enrichment Score: 2.048358806290177

Category	Term	Count	%	PValue	Genes	List Total	Pop Hits	Pop Total	Fold Enrichment	Bonferroni	Benjamini	FDR
GOTERM_MF_FAT	GO:0019955~cytokine binding	11	3.18	1.38E-06	IL18BP, CCR5, CSF2RB2, IL10RA, CCR2, CX3CR1, CSF2RB, IL2RG, IL12B, XCR1, CSF2RA	214	88	13288	7.76	4.45E-04	4.45E-04	1.86E-03

Table S1.H GO Terms upregulated 504 hours post injury

Annotation Cluster 1 Enrichment Score: 9.147013030129218

Category	Term	Count	%	PValue	Genes	List Total	Pop Hits	Pop Total	Fold Enrichment	Bonferroni	Benjamini	FDR
GOTERM_BP_FAT	GO:0006955~immune response	55	7.21	3.31E-12	LY86, TLR2, H2-D1, C1QC, TLR7, TGFB1, B2M, CFP, C1RA, CLEC4A2, SPON2, DLG1, H2-K1, NCF1, H2-DMB1, SERPING1, CLEC4N, C1QA, C1QB, LAT2, CCR5, LILRB3, LILRB4, CCR2, H2-AA, PTMS, CD300LB, LCP1, CD300LD, LST1, CCL2, CXCL9, CCL9, CCL8, PF4, C1S, CD74, CCL6, SLC11A1, FCER1G, PTPN6, PTPRC, CFB, TLR13, MYO1F, H2-AB1, FCGR1, FCGR3, CCL12, ABCC9, FCGR2B, CD300A, H2-EB1, CLEC7A, CD14	550	471	13588	2.88	8.17E-09	2.72E-09	5.82E-09
GOTERM_BP_FAT	GO:0009611~response to wounding	44	5.77	5.00E-11	CCL2, LY86, TLR2, CXCL9, CCL8, ITGB2, PF4, C1S, C1QC, MDK, TLR7, TGFB1, CFP, C1RA, SLC11A1, GPX1, CD44, BCL2, PYCARD, TFPI2, ENTPD2, FN1, PTPN6, GATM, PLEK, CFB, NCF1, TLR13, SERPING1, ANXA5, FCGR1, FCGR3, C1QA, C1QB, CCL12, CCR5, NUPR1, STAB1, CCR2, PLA2G7, CLEC7A, CD14, IGFBP4, LCP1	550	347	13588	3.13	1.24E-07	2.06E-08	8.80E-08
GOTERM_BP_FAT	GO:0006952~defense response	51	6.68	5.98E-11	LY86, H2-D1, TLR2, C1QC, TLR7, TGFB1, B2M, CFP, C1RA, CD44, DDAH2, H2-K1, NCF1, SERPING1, CLIC1, C1QA, C1QB, CCR5, CCR2, PLA2G7, H2-AA, CCL2, CXCL9, CCL8, ITGB2, C1S, CD74, SLC11A1, BCL2, PYCARD, FCER1G, FN1, PTPN6, PTPRC, LYZ2, LYZ1, CFB, TLR13, MYO1F, COTL1, FCGR1, FCGR3, CCL12, ABCC9, PENK, FCGR2B, NUPR1, STAB1, CLEC7A, IGFBP4, CD14	550	448	13588	2.81	1.48E-07	2.11E-08	1.05E-07
GOTERM_BP_FAT	GO:0006954~inflammatory response	33	4.33	5.00E-10	CCL2, LY86, TLR2, CXCL9, CCL8, ITGB2, C1S, TLR7, C1QC, TGFB1, CFP, C1RA, SLC11A1, CD44, PYCARD, FN1, CFB, NCF1, TLR13, SERPING1, FCGR1, FCGR3, C1QA, C1QB, CCL12, NUPR1, CCR5, STAB1, CCR2, PLA2G7, CLEC7A, CD14, IGFBP4	550	225	13588	3.62	1.24E-06	1.37E-07	8.81E-07
GOTERM_BP_FAT	GO:0002252~immune effector process	24	3.15	1.06E-09	PTPN6, PTPRC, CFB, NCF1, MYO1F, SERPING1, C1S, TLR7, C1QC, FCGR1, CD74, FCGR3, CFP, C1QA, C1QB, SLC11A1, C1RA, LAT2, ABCC9, FCGR2B, LILRB3, FCER1G, H2-AA, DLG1	550	126	13588	4.71	2.63E-06	2.39E-07	1.87E-06
GOTERM_BP_FAT	GO:0045087~innate immune response	16	2.10	2.51E-05	PTPN6, CFB, TLR13, TLR2, SERPING1, C1S, C1QC, FCGR1, TLR7, TGFB1, CFP, C1QA, C1QB, SLC11A1, C1RA, CLEC7A	550	107	13588	3.69	6.01E-02	1.82E-03	4.41E-02

Annotation Cluster 2 Enrichment Score: 7.651275481859161

Category	Term	Count	%	PValue	Genes	List Total	Pop Hits	Pop Total	Fold Enrichment	Bonferroni	Benjamini	FDR
GOTERM_MF_FAT	GO:0030246~carbohydrate binding	39	5.11	7.97E-10	GLG1, GALNT5, CD248, CCL8, POSTN, PF4, FSTL1, GPCPD1, CLEC10A, MDK, CD44, APOE, PTN, CLEC4A1, CLEC4A2, CLEC4A3, GPNMB, THBS2, FN1, LPHN1, MRC1, PTPRC, LGALS3, CCDC80, STAB2, COL5A3, CLEC4N, COL5A1, BGN, ADAMTS8, GCK, CLEC12A, STAB1, CLEC3B, CD33, CLEC3A, FBLN7, VCAN, CLEC7A	523	317	13288	3.13	5.24E-07	5.24E-07	1.19E-06
GOTERM_MF_FAT	GO:0005539~glycosaminoglycan binding	21	2.75	1.65E-08	PTPRC, CCDC80, CCL8, POSTN, FSTL1, PF4, STAB2, COL5A3, MDK, COL5A1, ADAMTS8, BGN, CD44, STAB1, APOE, FBLN7, PTN, VCAN, GPNMB, THBS2, FN1	523	114	13288	4.68	1.08E-05	5.42E-06	2.46E-05
GOTERM_MF_FAT	GO:0001871~pattern binding	22	2.88	2.47E-08	PTPRC, CCDC80, CCL8, POSTN, FSTL1, PF4, STAB2, COL5A3, MDK, COL5A1, ADAMTS8, BGN, CD44, STAB1, APOE, FBLN7, PTN, VCAN, CLEC7A, GPNMB, THBS2, FN1	523	128	13288	4.37	1.63E-05	5.42E-06	3.70E-05
GOTERM_MF_FAT	GO:0030247~polysaccharide binding	22	2.88	2.47E-08	PTPRC, CCDC80, CCL8, POSTN, FSTL1, PF4, STAB2, COL5A3, MDK, COL5A1, ADAMTS8, BGN, CD44, STAB1, APOE, FBLN7, PTN, VCAN, CLEC7A, GPNMB, THBS2, FN1	523	128	13288	4.37	1.63E-05	5.42E-06	3.70E-05
GOTERM_MF_FAT	GO:0008201~heparin binding	16	2.10	6.92E-07	PTPRC, CCDC80, CCL8, FSTL1, PF4, POSTN, COL5A3, MDK, COL5A1, ADAMTS8, APOE, FBLN7, PTN, GPNMB, THBS2, FN1	523	83	13288	4.90	4.55E-04	1.14E-04	1.03E-03

Annotation Cluster 3 Enrichment Score: 6.147770208479148

Category	Term	Count	%	PValue	Genes	List Total	Pop Hits	Pop Total	Fold Enrichment	Bonferroni	Benjamini	FDR
GOTERM_BP_FAT	GO:0002478~antigen processing and presentation of exogenous peptide antigen	13	1.70	1.51E-11	H2-K1, UNC93B1, IFI30, H2-DMB1, H2-AB1, FCGR1, CD74, B2M, FCGR3, FCGR2B, H2-EB1, H2-AA, FCER1G	550	23	13588	13.96	3.74E-08	9.36E-09	2.67E-08
GOTERM_BP_FAT	GO:0048002~antigen processing and presentation of peptide antigen	15	1.97	2.78E-11	H2-K1, UNC93B1, H2-D1, IFI30, H2-DMB1, H2-AB1, FCGR1, CD74, B2M, FCGR3, SLC11A1, FCGR2B, H2-EB1, FCER1G, H2-AA	550	35	13588	10.59	6.88E-08	1.38E-08	4.90E-08
GOTERM_BP_FAT	GO:0019884~antigen processing and presentation of exogenous antigen	13	1.70	2.83E-10	H2-K1, UNC93B1, IFI30, H2-DMB1, H2-AB1, FCGR1, CD74, B2M, FCGR3, FCGR2B, H2-EB1, H2-AA, FCER1G	550	28	13588	11.47	7.00E-07	8.74E-08	4.98E-07
GOTERM_BP_FAT	GO:0019886~antigen processing and presentation of exogenous peptide antigen via MHC class II	9	1.18	6.52E-08	FCGR2B, H2-EB1, UNC93B1, H2-AA, FCER1G, H2-DMB1, IFI30, H2-AB1, CD74	550	16	13588	13.90	1.61E-04	1.08E-05	1.15E-04
GOTERM_BP_FAT	GO:0002495~antigen processing and presentation of peptide antigen via MHC class II	9	1.18	6.52E-08	FCGR2B, H2-EB1, UNC93B1, H2-AA, FCER1G, H2-DMB1, IFI30, H2-AB1, CD74	550	16	13588	13.90	1.61E-04	1.08E-05	1.15E-04
GOTERM_BP_FAT	GO:0002504~antigen processing and presentation of peptide or polysaccharide antigen via MHC class I	9	1.18	3.44E-07	FCGR2B, H2-EB1, UNC93B1, H2-AA, FCER1G, H2-DMB1, IFI30, H2-AB1, CD74	550	19	13588	11.70	8.50E-04	5.00E-05	6.06E-04
GOTERM_BP_FAT	GO:0019882~antigen processing and presentation	15	1.97	9.34E-06	H2-K1, UNC93B1, H2-D1, IFI30, H2-DMB1, H2-AB1, FCGR1, CD74, B2M, FCGR3, SLC11A1, FCGR2B, H2-EB1, FCER1G, H2-AA	550	87	13588	4.26	2.28E-02	7.45E-04	1.65E-02

Annotation Cluster 4 Enrichment Score: 5.201812368353064

Category	Term	Count	%	PValue	Genes	List Total	Pop Hits	Pop Total	Fold Enrichment	Bonferroni	Benjamini	FDR
GOTERM_BP_FAT	GO:0002684~positive regulation of immune system process	31	4.06	9.83E-10	ITGAL, STAT5B, UNC93B1, TLR2, CACNB3, C1S, C1QC, TGFB1, CD74, B2M, CFP, C1RA, SLC11A1, HLX, FCER1G, H2-K1, PTPN6, PTPRC, CFB, SERPING1, FCGR1, FCGR3, THY1, C1QA, C1QB, CD83, CDKN1A, LAT2, CORO1A, H2-AA, CLEC7A	550	206	13588	3.72	2.43E-06	2.43E-07	1.73E-06
GOTERM_BP_FAT	GO:0002252~immune effector process	24	3.15	1.06E-09	PTPN6, PTPRC, CFB, NCF1, MYO1F, SERPING1, C1S, TLR7, C1QC, FCGR1, CD74, FCGR3, CFP, C1QA, C1QB, SLC11A1, C1RA, LAT2, ABCC9, FCGR2B, LILRB3, FCER1G, H2-AA, DLG1	550	126	13588	4.71	2.63E-06	2.39E-07	1.87E-06
GOTERM_BP_FAT	GO:0002443~leukocyte mediated immunity	20	2.62	1.98E-09	PTPN6, NCF1, CFB, MYO1F, SERPING1, C1S, C1QC, FCGR1, CD74, FCGR3, C1QA, C1QB, SLC11A1, C1RA, LAT2, FCGR2B, LILRB3, FCER1G, H2-AA, DLG1	550	89	13588	5.55	4.90E-06	4.08E-07	3.49E-06
GOTERM_BP_FAT	GO:0050778~positive regulation of immune response	24	3.15	4.98E-09	H2-K1, PTPN6, PTPRC, CFB, STAT5B, TLR2, UNC93B1, CACNB3, SERPING1, C1S, C1QC, FCGR1, FCGR3, THY1, B2M, CFP, C1QA, C1QB, SLC11A1, C1RA, LAT2, FCER1G, H2-AA, CLEC7A	550	136	13588	4.36	1.23E-05	9.47E-07	8.77E-06
GOTERM_BP_FAT	GO:0002449~lymphocyte mediated immunity	17	2.23	4.64E-08	PTPN6, CFB, SERPING1, C1S, C1QC, FCGR1, CD74, FCGR3, C1QA, C1QB, SLC11A1, C1RA, FCGR2B, LILRB3, FCER1G, H2-AA, DLG1	550	76	13588	5.53	1.15E-04	8.19E-06	8.17E-05
GOTERM_BP_FAT	GO:0002253~activation of immune response	17	2.23	2.85E-07	PTPN6, PTPRC, CFB, UNC93B1, TLR2, SERPING1, CACNB3, C1S, C1QC, THY1, CFP, C1QA, C1QB, C1RA, LAT2, FCER1G, CLEC7A	550	86	13588	4.88	7.03E-04	4.40E-05	5.01E-04
GOTERM_BP_FAT	GO:0048584~positive regulation of response to stimulus	25	3.28	4.79E-07	STAT5B, TLR2, UNC93B1, CACNB3, C1S, C1QC, B2M, CFP, C1RA, SLC11A1, FCER1G, H2-K1, PTPN6, PTPRC, CFB, SERPING1, FCGR1, THY1, FCGR3, C1QA, C1QB, LAT2, HIPK2, H2-AA, CLEC7A	550	186	13588	3.32	1.18E-03	6.58E-05	8.43E-04
GOTERM_BP_FAT	GO:0002460~adaptive immune response based on somatic recombination of immune receptors built fr	16	2.10	1.16E-06	CFB, SERPING1, C1S, C1QC, FCGR1, CD74, FCGR3, C1QA, C1QB, SLC11A1, C1RA, FCGR2B, LILRB3, FCER1G, H2-AA, DLG1	550	84	13588	4.71	2.86E-03	1.43E-04	2.04E-03
GOTERM_BP_FAT	GO:0002250~adaptive immune response	16	2.10	1.16E-06	CFB, SERPING1, C1S, C1QC, FCGR1, CD74, FCGR3, C1QA, C1QB, SLC11A1, C1RA, FCGR2B, LILRB3, FCER1G, H2-AA, DLG1	550	84	13588	4.71	2.86E-03	1.43E-04	2.04E-03
GOTERM_BP_FAT	GO:0019724~B cell mediated immunity	14	1.83	1.58E-06	CFB, SERPING1, C1S, C1QC, FCGR1, CD74, FCGR3, C1QA, C1RA, C1QB, FCGR2B, LILRB3, H2-AA, FCER1G	550	65	13588	5.32	3.90E-03	1.86E-04	2.78E-03
GOTERM_BP_FAT	GO:0016064~immunoglobulin mediated immune response	13	1.70	6.80E-06	CFB, SERPING1, C1S, C1QC, FCGR1, CD74, FCGR3, C1QA, C1RA, C1QB, FCGR2B, H2-AA, FCER1G	550	63	13588	5.10	1.67E-02	5.80E-04	1.20E-02
GOTERM_BP_FAT	GO:0045087~innate immune response	16	2.10	2.51E-05	PTPN6, CFB, TLR13, TLR2, SERPING1, C1S, C1QC, FCGR1, TLR7, TGFB1, CFP, C1QA, C1QB, SLC11A1, C1RA, CLEC7A	550	107	13588	3.69	6.01E-02	1.82E-03	4.41E-02

Annotation Cluster 5 Enrichment Score: 4.873233284041742

Category	Term	Count	%	PValue	Genes	List Total	Pop Hits	Pop Total	Fold Enrichment	Bonferroni	Benjamini	FDR
GOTERM_BP_FAT	GO:0060348~bone development	18	2.36	5.02E-06	IBSP, IMPAD1, BMP1, MGP, FHL2, SMAD3, IGF2, MMP14, MMP2, HDAC4, BCL2, ANKRD11, SORT1, PTN, MYOG, COL1A1, GPNMB, SPP1	550	118	13588	3.77	1.23E-02	4.60E-04	8.85E-03
GOTERM_BP_FAT	GO:0001503~ossification	17	2.23	5.14E-06	IBSP, IMPAD1, BMP1, MGP, SMAD3, FHL2, IGF2, MMP14, MMP2, HDAC4, BCL2, SORT1, PTN, MYOG, COL1A1, GPNMB, SPP1	550	106	13588	3.96	1.26E-02	4.54E-04	9.05E-03

Annotation Cluster 6 Enrichment Score: 3.6600379156900367

Category	Term	Count	%	PValue	Genes	List Total	Pop Hits	Pop Total	Fold Enrichment	Bonferroni	Benjamini	FDR
GOTERM_BP_FAT	GO:0006979~response to oxidative stress	15	1.97	9.34E-06	PXDN, GATM, ATOX1, CLU, PRND, MYH7, MMP14, GPX1, UCP2, APOE, HMOX1, BCL2, CYGB, GPX8, COL1A1	550	87	13588	4.26	2.28E-02	7.45E-04	1.65E-02

Annotation Cluster 7 Enrichment Score: 3.05794582612324

Category	Term	Count	%	PValue	Genes	List Total	Pop Hits	Pop Total	Fold Enrichment	Bonferroni	Benjamini	FDR
GOTERM_BP_FAT	GO:0060537~muscle tissue development	19	2.49	9.01E-06	MYL6, ACTC1, RXRA, TNC, ELN, DICER1, FHL2, HOMER1, TGFB1, TNNT2, GPX1, COL19A1, EP300, TSC1, HLX, MYOG, PAK1, CHRNA1, DLG1	550	136	13588	3.45	2.20E-02	7.43E-04	1.59E-02
GOTERM_BP_FAT	GO:0014706~striated muscle tissue development	18	2.36	1.37E-05	MYL6, ACTC1, RXRA, TNC, ELN, DICER1, FHL2, HOMER1, TGFB1, TNNT2, GPX1, COL19A1, EP300, TSC1, HLX, MYOG, PAK1, CHRNA1	550	127	13588	3.50	3.33E-02	1.06E-03	2.41E-02

Annotation Cluster 8 Enrichment Score: 2.99846280110976

Category	Term	Count	%	PValue	Genes	List Total	Pop Hits	Pop Total	Fold Enrichment	Bonferroni	Benjamini	FDR
GOTERM_BP_FAT	GO:0001775~cell activation	28	3.67	2.38E-06	ITGAL, LST1, STAT5B, RORC, PF4, SFPI1, ITGB2, TGFB1, CD74, CD48, SLC11A1, CXCR4, BCL2, FCER1G, ENTPD2, DLG1, FYB, PTPRC, PLEK, SMAD3, MYO1F, FCGR3, LAT2, CD86, FCGR2B, LILRB3, LCP1, LCP2	550	246	13588	2.81	5.86E-03	2.45E-04	4.19E-03
GOTERM_BP_FAT	GO:0045321~leukocyte activation	24	3.15	2.70E-05	FYB, PTPRC, ITGAL, STAT5B, MYO1F, SMAD3, RORC, ITGB2, SFPI1, TGFB1, CD74, FCGR3, CD48, SLC11A1, LAT2, CD86, FCGR2B, CXCR4, LILRB3, BCL2, FCER1G, LCP1, DLG1, LCP2	550	219	13588	2.71	6.46E-02	1.91E-03	4.76E-02

Annotation Cluster 13 Enrichment Score: 2.2379834754282384

Category	Term	Count	%	PValue	Genes	List Total	Pop Hits	Pop Total	Fold Enrichment	Bonferroni	Benjamini	FDR
GOTERM_BP_FAT	GO:0051130~positive regulation of cellular component organization	19	2.49	1.87E-06	LST1, PLEK, CDH4, SIRPA, FCGR1, TGFB1, FCGR3, SLC11A1, MIB1, GPC3, FCGR2B, TIAM1, TPPP, MAPT, CFL1, FCER1G, CLEC7A, PTMS, DLG1	550	122	13588	3.85	4.62E-03	2.10E-04	3.30E-03

Table S1.I GO Terms upregulated 672 hours post injury

Annotation Cluster 1 Enrichment Score: 9.542765917628339

Category	Term	Count	%	PValue	Genes	List Total	Pop Hits	Pop Total	Fold Enrichment	Bonferroni	Benjamini	FDR
GOTERM_MF_FAT	GO:0005539~glycosaminoglycan binding	23	3.40	8.56E-11	PTPRC, CCDC80, CCL8, CHODL, POSTN, PF4, FSTL1, STAB2, DCN, COL5A1, ABI3BP, NCAM1, ADAMTS8, BGN, CD44, APOE, COMP, CFH, FBLN7, GPNMB, FGF2, THBS2, FN1	478	114	13288	5.61	5.13E-08	2.57E-08	1.26E-07
GOTERM_MF_FAT	GO:0030247~polysaccharide binding	24	3.55	1.44E-10	PTPRC, CCDC80, CCL8, CHODL, POSTN, PF4, FSTL1, STAB2, DCN, COL5A1, ABI3BP, NCAM1, ADAMTS8, BGN, CD44, APOE, COMP, CFH, FBLN7, CLEC7A, GPNMB, FGF2, THBS2, FN1	478	128	13288	5.21	8.65E-08	2.88E-08	2.13E-07
GOTERM_MF_FAT	GO:0001871~pattern binding	24	3.55	1.44E-10	PTPRC, CCDC80, CCL8, CHODL, POSTN, PF4, FSTL1, STAB2, DCN, COL5A1, ABI3BP, NCAM1, ADAMTS8, BGN, CD44, APOE, COMP, CFH, FBLN7, CLEC7A, GPNMB, FGF2, THBS2, FN1	478	128	13288	5.21	8.65E-08	2.88E-08	2.13E-07
GOTERM_MF_FAT	GO:0030246~carbohydrate binding	38	5.62	2.31E-10	EMCN, GALNT5, CCL8, POSTN, FSTL1, PF4, DCN, CLEC10A, ABI3BP, CD44, APOE, COMP, CFH, CLEC4A2, CLEC4A3, GPNMB, FGF2, SELPLG, THBS2, FN1, LPHN1, MRC1, PTPRC, LGALS3, CCDC80, CHODL, COLEC12, STAB2, COL5A1, CLEC11A, NCAM1, BGN, ADAMTS8, GCK, CLEC12A, CLEC3B, FBLN7, CLEC7A	478	317	13288	3.33	1.39E-07	3.47E-08	3.42E-07
GOTERM_MF_FAT	GO:0008201~heparin binding	18	2.66	4.69E-09	PTPRC, CCDC80, CCL8, POSTN, FSTL1, PF4, ABI3BP, COL5A1, NCAM1, ADAMTS8, APOE, COMP, FBLN7, CFH, GPNMB, FGF2, THBS2, FN1	478	83	13288	6.03	2.82E-06	5.63E-07	6.93E-06

Annotation Cluster 2 Enrichment Score: 6.07655836906391

Category	Term	Count	%	PValue	Genes	List Total	Pop Hits	Pop Total	Fold Enrichment	Bonferroni	Benjamini	FDR
GOTERM_BP_FAT	GO:0006955~immune response	45	6.66	8.11E-09	LY86, H2-D1, CCL9, CXCL9, ACP5, CCL8, PF4, C1S, CD1D1, C1QC, CD74, CCL6, B2M, CFP, C1RA, GP49A, CFH, FCER1G, CLEC4A2, AF251705, C2, SPON2, CFD, PTPRC, CFB, IGJ, H2-DMB1, COLEC12, H2-AB1, SERPING1, FCGR3, C1QA, CCL11, C1QB, CD55, FCGR2B, LILRB3, CCR2, H2-EB1, LILRB4, IL12A, H2-AA, CLEC7A, PTMS, GBP2, LCP1	494	471	13588	2.63	1.79E-05	3.57E-06	1.41E-05
GOTERM_BP_FAT	GO:0009611~response to wounding	37	5.47	1.35E-08	LY86, F13A1, CXCL9, GJA1, CCL8, PF4, C1S, C1QC, TRF, CFP, C1RA, GPX1, CD44, BCL2, CFH, C2, CFD, ENTPD2, FN1, PLEK, CFB, SERPING1, ANXA5, FCGR3, C1QA, CCL11, C1QB, CD55, SERPINA3N, NUPR1, F3, CCR2, PLA2G7, ID3, CLEC7A, PROS1, LCP1	494	347	13588	2.93	2.96E-05	4.94E-06	2.34E-05
GOTERM_BP_FAT	GO:0002526~acute inflammatory response	17	2.51	2.70E-08	CFB, SERPING1, C1S, C1QC, TRF, FCGR3, CFP, C1QA, C1QB, C1RA, CD55, SERPINA3N, NUPR1, CFH, C2, CFD, FN1	494	81	13588	5.77	5.93E-05	7.42E-06	4.69E-05
GOTERM_BP_FAT	GO:0002541~activation of plasma proteins involved in acute inflammatory response	12	1.78	3.39E-08	C1QA, CFP, C1QB, C1RA, CD55, CFB, CFH, SERPING1, C2, C1S, CFD, C1QC	494	36	13588	9.17	7.46E-05	8.29E-06	5.89E-05
GOTERM_BP_FAT	GO:0006956~complement activation	12	1.78	3.39E-08	C1QA, CFP, C1QB, C1RA, CD55, CFB, CFH, SERPING1, C2, C1S, CFD, C1QC	494	36	13588	9.17	7.46E-05	8.29E-06	5.89E-05
GOTERM_BP_FAT	GO:0006959~humoral immune response	14	2.07	4.58E-08	CFB, IGJ, SERPING1, C1S, C1QC, C1QA, CFP, C1QB, C1RA, CD55, CCR2, CFH, C2, CFD	494	54	13588	7.13	1.01E-04	1.01E-05	7.96E-05
GOTERM_BP_FAT	GO:0019724~B cell mediated immunity	15	2.22	6.35E-08	CFB, SERPING1, C1S, C1QC, CD74, FCGR3, C1QA, C1QB, C1RA, CD55, FCGR2B, LILRB3, H2-AA, FCER1G, C2	494	65	13588	6.35	1.40E-04	1.17E-05	1.10E-04
GOTERM_BP_FAT	GO:0050778~positive regulation of immune response	21	3.11	9.50E-08	PTPRC, CFB, UNC93B1, SERPING1, C1S, CD1D1, C1QC, PNP, FCGR3, B2M, CFP, C1QA, C1QB, C1RA, CD55, CFH, FCER1G, H2-AA, C2, CLEC7A, CFD	494	136	13588	4.25	2.09E-04	1.61E-05	1.65E-04
GOTERM_BP_FAT	GO:0002684~positive regulation of immune system process	26	3.85	1.19E-07	UNC93B1, C1S, CD1D1, C1QC, PNP, CD74, B2M, CFP, C1RA, CFH, FCER1G, C2, CFD, PTPRC, CFB, SERPING1, FCGR3, C1QA, C1QB, CD83, CDKN1A, CORO1A, CD55, IL12A, H2-AA, CLEC7A	494	206	13588	3.47	2.62E-04	1.87E-05	2.07E-04
GOTERM_BP_FAT	GO:0016064~immunoglobulin mediated immune response	14	2.07	3.21E-07	CFB, SERPING1, C1S, C1QC, CD74, FCGR3, C1QA, C1QB, C1RA, CD55, FCGR2B, H2-AA, FCER1G, C2	494	63	13588	6.11	7.06E-04	4.42E-05	5.58E-04
GOTERM_BP_FAT	GO:0002253~activation of immune response	16	2.37	4.08E-07	PTPRC, CFB, UNC93B1, SERPING1, C1S, C1QC, CFP, C1QA, C1QB, C1RA, CD55, CFH, FCER1G, CLEC7A, C2, CFD	494	86	13588	5.12	8.99E-04	5.29E-05	7.10E-04
GOTERM_BP_FAT	GO:0002449~lymphocyte mediated immunity	15	2.22	4.96E-07	CFB, SERPING1, C1S, C1QC, CD74, FCGR3, C1QA, C1QB, C1RA, CD55, FCGR2B, LILRB3, H2-AA, FCER1G, C2	494	76	13588	5.43	0.001092239	6.07E-05	8.63E-04
GOTERM_BP_FAT	GO:0002252~immune effector process	19	2.81	6.47E-07	PTPRC, CFB, SERPING1, C1S, C1QC, CD74, FCGR3, CFP, C1QA, C1QB, C1RA, CD55, FCGR2B, LILRB3, CFH, FCER1G, H2-AA, C2, CFD	494	126	13588	4.15	0.001424535	7.50E-05	0.001125567
GOTERM_BP_FAT	GO:0006952~defense response	39	5.77	1.02E-06	LY86, H2-D1, CXCL9, CCL8, C1S, CD1D1, C1QC, CD74, TRF, B2M, CFP, C1RA, CD44, BCL2, CFH, FCER1G, C2, CFD, FN1, PTPRC, LYZ2, LYZ1, NGP, CFB, SERPING1, CLIC1, FCGR3, CCL11, C1QA, C1QB, CD55, SERPINA3N, PENK, NUPR1, FCGR2B, CCR2, PLA2G7, H2-AA, CLEC7A	494	448	13588	2.39	0.002237628	1.07E-04	0.00176873
GOTERM_BP_FAT	GO:0048584~positive regulation of response to stimulus	23	3.40	1.06E-06	PTPRC, CFB, UNC93B1, SERPING1, C1S, CD1D1, C1QC, PNP, FCGR3, B2M, CFP, C1QA, C1QB, C1RA, CD55, HIPK2, CFH, FCER1G, FABP4, H2-AA, C2, CLEC7A, CFD	494	186	13588	3.40	0.002340331	1.06E-04	0.001850005
GOTERM_BP_FAT	GO:0002250~adaptive immune response	15	2.22	1.76E-06	CFB, SERPING1, C1S, C1QC, CD74, FCGR3, C1QA, C1QB, C1RA, CD55, FCGR2B, LILRB3, H2-AA, FCER1G, C2	494	84	13588	4.91	0.003860339	1.61E-04	0.003053862
GOTERM_BP_FAT	GO:0002460~adaptive immune response based on somatic recombination of immune receptors built f	15	2.22	1.76E-06	CFB, SERPING1, C1S, C1QC, CD74, FCGR3, C1QA, C1QB, C1RA, CD55, FCGR2B, LILRB3, H2-AA, FCER1G, C2	494	84	13588	4.91	0.003860339	1.61E-04	0.003053862
GOTERM_BP_FAT	GO:0006954~inflammatory response	25	3.70	2.23E-06	LY86, CXCL9, CCL8, C1S, C1QC, TRF, CFP, C1RA, CD44, CFH, C2, CFD, FN1, CFB, SERPING1, FCGR3, CCL11, C1QA, C1QB, SERPINA3N, CD55, NUPR1, CCR2, PLA2G7, CLEC7A	494	225	13588	3.06	0.004909066	1.97E-04	0.003885524
GOTERM_BP_FAT	GO:0051605~protein maturation by peptide bond cleavage	13	1.92	3.18E-06	CFB, SERPING1, C1S, MMP14, C1QC, C1QA, CFP, C1RA, C1QB, CD55, CFH, C2, CFD	494	65	13588	5.50	0.006979556	2.59E-04	0.005530022
GOTERM_BP_FAT	GO:0002443~leukocyte mediated immunity	15	2.22	3.58E-06	CFB, SERPING1, C1S, C1QC, CD74, FCGR3, C1QA, C1QB, C1RA, CD55, FCGR2B, LILRB3, H2-AA, FCER1G, C2	494	89	13588	4.64	0.007847113	2.81E-04	0.006220095
GOTERM_BP_FAT	GO:0006958~complement activation, classical pathway	9	1.33	8.20E-06	C1QA, C1QB, C1RA, CD55, CFB, SERPING1, C2, C1S, C1QC	494	30	13588	8.25	0.017900139	5.64E-04	0.014260434
GOTERM_BP_FAT	GO:0051604~protein maturation	15	2.22	8.89E-06	CFB, SERPING1, C1S, MMP14, C1QC, SERPINH1, CFP, C1QA, C1QB, C1RA, CD55, CFH, C2, CASP1, CFD	494	96	13588	4.30	0.01938729	5.93E-04	0.015456775
GOTERM_BP_FAT	GO:0016485~protein processing	14	2.07	2.06E-05	CFB, SERPING1, C1S, MMP14, C1QC, C1QA, CFP, C1QB, C1RA, CD55, CFH, C2, CASP1, CFD	494	90	13588	4.28	0.044356591	0.001259496	0.035816753
GOTERM_BP_FAT	GO:0002455~humoral immune response mediated by circulating immunoglobulin	9	1.33	2.81E-05	C1QA, C1QB, C1RA, CD55, CFB, SERPING1, C2, C1S, C1QC	494	35	13588	7.07	0.060026252	0.001671664	0.048865114

Annotation Cluster 3 Enrichment Score: 4.451771606128764

Category	Term	Count	%	PValue	Genes	List Total	Pop Hits	Pop Total	Fold Enrichment	Bonferroni	Benjamini	FDR
GOTERM_BP_FAT	GO:0001501~skeletal system development	29	4.29	1.71E-06	IBSP, RBP4, FGFRL1, ACP5, ZBTB16, MMP2, IGSF10, BCL2, MYOG, GPNMB, RUNX1, COL11A1, MDFI, BMP1, LGALS3, IGF1, MGP, TBX1, IGF2, MMP14, COL5A2, MMP13, HOXC10, CLEC3B, KAZALD1, UBC, PDGFRA, COL1A1, IGFBP3	494	285	13588	2.80	0.003748538	1.63E-04	0.002965253
GOTERM_BP_FAT	GO:0060348~bone development	17	2.51	5.42E-06	IBSP, BMP1, MGP, ACP5, IGF1, IGF2, MMP14, MMP2, MMP13, IGSF10, KAZALD1, BCL2, UBC, MYOG, COL1A1, GPNMB, IGFBP3	494	118	13588	3.96	0.011867479	4.12E-04	0.009425814
GOTERM_BP_FAT	GO:0001503~ossification	16	2.37	6.24E-06	IBSP, BMP1, MGP, IGF1, IGF2, MMP14, MMP2, MMP13, IGSF10, KAZALD1, BCL2, UBC, MYOG, COL1A1, GPNMB, IGFBP3	494	106	13588	4.15	0.01364875	4.43E-04	0.010850285

Annotation Cluster 4 Enrichment Score: 3.45545827605697

Category	Term	Count	%	PValue	Genes	List Total	Pop Hits	Pop Total	Fold Enrichment	Bonferroni	Benjamini	FDR
GOTERM_MF_FAT	GO:0005509~calcium ion binding	73	10.80	3.05E-12	S100A4, S100A6, PXDN, LTBP1, S100A8, AIF1, F13A1, FAM20C, S100A9, SYT3, FSTL1, MMP3, MMP2, C1RA, PLCB4, SLC25A25, GSN, AIF1L, EEF2K, SPON2, PLS3, KCNMA1, CACNG7, MGP, PADI2, COLEC12, MMP14, MMP13, PLCE1, NUCB2, MFAP4, PROS1, LCP1, AOC3, MYL4, FKBP7, MYL2, EFCAB6, GALNT5, C1S, CLEC10A, CDH4, TPM4, DAGLA, ATN1, COMP, MAN1A, EGF, THBS2, ENTPD2, THBS3, MRC1, BMP1, ANXA1, S100A11, S100A10, NID1, SPARC, ITGA4, ANXA5, ANXA4, ITPR1, S100A13, ANXA2, FBLN1, CDH15, SULF2, EMR1, CLEC3B, FBLN7, RCN3, VLDLR, CDH11	478	840	13288	2.42	1.83E-09	1.83E-09	4.51E-09

Table S2.A KEGG pathways significant at 3 hours

Category	Term	Count	%	PValue	Genes	List Total	Pop Hits	Pop Total	Fold Enrichment	Bonferroni	Benjamini	FDR
KEGG_PATHWAY	mmu04060:Cytokine-cytokine receptor interaction	49	4.52	2.96E-10	OSMR, CXCR2, IL17RA, TGFB2, CXCR4, IL1RAP, CSF3R, CSF2RB, IL1B, FAS, IL13RA1, CSF2RA, EGFR, LTBR, INHBB, OSM, TNFRSF10B, PPBP, CCR2, PDGFRA, CXCL1, CSF3, IL1R2, CCL3, IL1R1, CCL2, CSF2RB2, TNFRSF12A, CSF1, CCR1, IL4RA, CXCL2, CXCL9, CCL9, PF4, CNTFR, CCL7, CCL6, CCL24, TNFRSF1A, TNFRSF1B, IL10RA, BMP2, TGFBR1, TGFBR2, CCL11, CXCL14, CXCL16, BMPR1B	434	244	5738	2.66	4.88E-08	4.88E-08	3.59E-07
KEGG_PATHWAY	mmu04512:ECM-receptor interaction	25	2.31	4.36E-09	TNC, COL3A1, SDC4, SDC2, CD44, COMP, COL6A2, COL6A1, COL11A2, THBS1, THBS2, THBS3, THBS4, SPP1, FN1, TNXB, COL4A1, ITGA3, ITGA4, COL5A2, LAMA4, ITGA5, COL1A2, LAMC2, COL1A1	434	83	5738	3.98	7.20E-07	3.60E-07	5.30E-06
KEGG_PATHWAY	mmu04610:Complement and coagulation cascades	22	2.03	7.69E-08	PLAT, C7, C5AR1, MASP1, C3, C4B, CFB, F13A1, SERPING1, C1S, C1QC, PLAUR, C1QA, C1QB, C1RA, THBD, F3, SERPINE1, CFH, CFD, PROS1, PLAU	434	75	5738	3.88	1.27E-05	4.23E-06	9.33E-05
KEGG_PATHWAY	mmu04510:Focal adhesion	37	3.41	4.52E-07	MYL2, PGF, TNC, COL3A1, RAC2, COMP, COL6A2, COL6A1, PIK3R5, ZYX, PAK1, COL11A2, THBS1, THBS2, THBS3, FN1, SPP1, THBS4, ACTB, EGFR, COL4A1, TNXB, ACTN1, ITGA3, ITGA4, FLNC, BIRC3, FLNB, COL5A2, LAMA4, ITGA5, FYN, PDGFRA, COL1A2, RAP1B, LAMC2, COL1A1	434	198	5738	2.47	7.45E-05	1.86E-05	5.48E-04
KEGG_PATHWAY	mmu04210:Apoptosis	22	2.03	1.18E-06	CFLAR, IL1R1, CSF2RB2, RELA, NFKBIA, NFKB1, BIRC3, PRKAR2B, TNFRSF1A, IRAK3, TNFRSF10B, MYD88, RIPK1, IL1RAP, CASP8, CASP12, IL1B, CSF2RB, PIK3R5, FAS, APAF1, MAP3K14	434	87	5738	3.34	1.95E-04	3.89E-05	1.43E-03
KEGG_PATHWAY	mmu04062:Chemokine signaling pathway	33	3.04	4.28E-06	CXCL1, ADCY4, CCL3, CCL2, GNAI3, CCR1, CXCL2, CCL9, CXCL9, NFKBIA, NFKB1, CXCR2, PF4, GNG11, CCL7, CCL6, CCL24, RAC2, TIAM1, PTK2B, CXCR4, PIK3R5, PAK1, LYN, RELA, STAT3, STAT2, CCL11, PPBP, CXCL14, CXCL16, CCR2, RAP1B	434	182	5738	2.40	7.05E-04	1.18E-04	5.19E-03
KEGG_PATHWAY	mmu04010:MAPK signaling pathway	41	3.78	1.37E-05	IL1R2, IL1R1, FGFR3, MRAS, DUSP10, HSPA1A, NFKB1, HSPA1B, TGFB2, FOS, TNFRSF1A, BDNF, RAC2, HSPA2, MAP3K2, MAP3K1, JUND, MAP3K8, IL1B, FAS, PAK1, RAPGEF2, MYC, EGFR, RELA, TGFBR1, TGFBR2, FLNC, FLNB, DUSP5, MAP4K4, DUSP2, GADD45G, NTRK2, PDGFRA, HSPB1, RAP1B, GADD45B, MAP3K14, GADD45A, CD14, PLA2G4E	434	265	5738	2.05	2.26E-03	3.23E-04	1.66E-02

Table S2.B KEGG pathways significant at 10 hours

Category	Term	Count	%	PValue	Genes	List Total	Pop Hits	Pop Total	Fold Enrichment	Bonferroni	Benjamini	FDR
KEGG_PATHWAY	mmu00190:Oxidative phosphorylation	80	2.10	8.69E-22	ATP5D, UQCRC2, ATP5E, COX11, UQCRC1, COX10, ATP5B, CYC1, NDUFAB1, UQCRFS1, COX5A, COX5B, UQCRQ, NDUFS7, NDUFS6, NDUFS5, UQCR10, UQCR11, NDUFS4, NDUFS8, ATP5L, ATP5O, NDUFS3, ATP5H, NDUFS1, ATP5K, ATP5J, COX15, NDUFB10, NDUFC2, COX4I2, COX4I1, ATP6V1H, NDUFC1, NDUFA10, COX6C, NDUFA11, ATP6V1A, UQCRH, ATP5C1, UQCRB, NDUFB3, NDUFB4, NDUFB5, NDUFB6, NDUFB7, NDUFB8, NDUFB9, COX7B, COX7C, ATP6V1B2, ATP5G1, COX7A2L, ATP5G3, NDUFB2, COX6B1, NDUFA4, NDUFA5, NDUFA2, ATP5J2, COX7A2, NDUFA3, NDUFA8, COX7A1, COX8B, NDUFA9, NDUFA6, NDUFA7, ATP5F1, NDUFA1, PPA2, SDHA, NDUFV3, SDHB, ATP6V0E2, NDUFV1, SDHC, NDUFV2, SDHD, ATP5A1	1276	130	5738	2.77	1.64E-19	1.64E-19	1.08E-18
KEGG_PATHWAY	mmu05012:Parkinson's disease	80	2.10	6.54E-21	ATP5D, UQCRC2, ATP5E, SNCAIP, UQCRC1, ATP5B, UBE2G1, CYC1, SNCA, NDUFAB1, UQCRFS1, COX5A, COX5B, UQCRQ, NDUFS7, NDUFS6, NDUFS5, CASP3, UQCR10, UQCR11, NDUFS4, NDUFS8, ATP5O, NDUFS3, ATP5H, NDUFS1, ATP5J, NDUFB10, SLC25A4, SLC25A5, CYCS, NDUFC2, COX4I2, COX4I1, NDUFC1, NDUFA10, COX6C, UQCRH, UBC, ATP5C1, UBB, UQCRB, NDUFB3, NDUFB4, NDUFB5, NDUFB6, NDUFB7, NDUFB8, NDUFB9, COX7B, COX7C, ATP5G1, COX7A2L, ATP5G3, NDUFB2, COX6B1, NDUFA4, NDUFA5, NDUFA2, COX7A2, NDUFA3, NDUFA8, COX7A1, COX8B, NDUFA9, NDUFA6, NDUFA7, ATP5F1, UBE2L6, VDAC2, VDAC3, NDUFA1, SDHA, NDUFV3, SDHB, NDUFV1, SDHC, NDUFV2, SDHD, APAF1, ATP5A1	1276	133	5738	2.70	1.24E-18	6.18E-19	8.12E-18
KEGG_PATHWAY	mmu05016:Huntington's disease	95	2.49	1.19E-18	ATP5D, UQCRC2, ATP5E, CLTB, UQCRC1, ATP5B, CYC1, NDUFAB1, REST, CLTC, COX5A, UQCRFS1, COX5B, UQCRQ, NDUFS7, GPX1, NDUFS6, AP2B1, NDUFS5, CASP3, UQCR10, UQCR11, NDUFS4, PLCB4, CASP8, NDUFS8, CREB3L2, ATP5O, NDUFS3, ATP5H, NDUFS1, ATP5J, NDUFB10, SLC25A4, SLC25A5, TAF4B, CYCS, NDUFC2, COX4I2, COX4I1, NDUFC1, NDUFA10, COX6C, UQCRH, ATP5C1, UQCRB, NDUFB3, NDUFB4, NDUFB5, NDUFB6, NDUFB7, NDUFB8, NDUFB9, COX7B, COX7C, ATP5G1, COX7A2L, ATP5G3, NDUFB2, POLR2A, TFAM, COX6B1, TGM2, TRP53, NDUFA4, NDUFA5, NDUFA2, COX7A2, NDUFA3, NDUFA8, COX7A1, COX8B, HTT, NDUFA9, DNALC1, NDUFA6, CREBBP, NDUFA7, ATP5F1, VDAC2, VDAC3, NDUFA1, SOD2, NDUFV3, SDHA, SDHB, SP1, BBC3, NDUFV1, SDHC, BAX, NDUFV2, SDHD, APAF1, ATP5A1	1276	183	5738	2.33	2.24E-16	7.48E-17	1.47E-15
KEGG_PATHWAY	mmu05010:Alzheimer's disease	93	2.44	1.09E-17	ATP5D, UQCRC2, ATP5E, UQCRC1, ATP5B, SNCA, CYC1, NDUFAB1, UQCRFS1, COX5A, COX5B, UQCRQ, NDUFS7, NDUFS6, NDUFS5, CASP3, UQCR10, UQCR11, NDUFS4, PLCB4, CASP8, NDUFS8, ATP5O, FAS, NDUFS3, ATP5H, NDUFS1, ATP5J, NDUFB10, ADAM10, CYCS, NDUFC2, COX4I2, COX4I1, NDUFC1, NDUFA10, CDK5, COX6C, UQCRH, BACE1, ATP5C1, EIF2AK3, UQCRB, BID, NDUFB3, NDUFB4, NDUFB5, CDK5R1, NDUFB6, NDUFB7, NDUFB8, NDUFB9, COX7B, COX7C, ATP5G1, COX7A2L, ATP5G3, NDUFB2, TNFRSF1A, COX6B1, GAPDH, NDUFA4, LPL, NDUFA5, NDUFA2, COX7A2, NDUFA3, NOS1, NDUFA8, COX7A1, COX8B, NDUFA9, NDUFA6, NDUFA7, ATP5F1, CAPN2, NDUFA1, ITPR2, ATF6, SDHA, NDUFV3, SDHB, LRP1, ATP2A2, ATP2A3, NDUFV1, SDHC, NDUFV2, CASP12, SDHD, APAF1, ATP5A1, CALM2	1276	182	5738	2.30	2.07E-15	5.17E-16	1.36E-14
KEGG_PATHWAY	mmu04510:Focal adhesion	82	2.15	1.46E-09	PDGFB, PDGFA, PGF, BCAR1, CHAD, VCL, ACTG1, ARHGAP5, PAK2, PAK4, SHC1, ZYX, COL11A2, COL11A1, PRKCA, EGFR, PARVG, ACTN4, BRAF, ROCK1, ROCK2, ACTN1, ACTN2, FLNC, FLNB, FLNA, VASP, VEGFB, CCND1, CCND2, JUN, VEGFA, COL1A2, PDGFRA, LAMC2, LAMC1, COL1A1, CAV3, IBSP, MYL2, TNC, COL3A1, COL2A1, ITGB1, PXN, MYL9, IGF1R, DOCK1, LAMB2, RAC2, COMP, ITGB7, ITGB6, COL6A2, PPP1R12A, COL6A1, PIK3R5, THBS1, THBS2, PIK3R1, THBS3, FN1, THBS4, SPP1, PIK3R2, ACTB, COL4A2, COL4A1, FLT1, TNXB, MET, BIRC3, CAPN2, COL5A2, VAV1, KDR, VWF, LAMA4, ITGA5, GRLF1, RAP1B, DIAP1	1276	198	5738	1.86	2.76E-07	5.53E-08	1.82E-06
KEGG_PATHWAY	mmu00020:Citrate cycle (TCA cycle)	23	0.60	8.98E-09	DLST, ACO2, SUCLG1, CS, IDH3B, ACLY, DLAT, OGDH, PDHB, IDH3A, SDHA, SDHB, IDH3G, SDHC, DLD, SDHD, IDH2, IDH1, PDHA1, FH1, SUCLA2, MDH2, MDH1	1276	31	5738	3.34	1.70E-06	2.83E-07	1.11E-05
KEGG_PATHWAY	mmu04260:Cardiac muscle contraction	37	0.97	2.11E-06	UQCRC2, ATP1B1, UQCRC1, MYL2, ATP1B3, TNNC1, MYL3, ATP1B4, CYC1, COX7B, COX7C, CACNB1, COX7A2L, UQCRFS1, COX5A, COX5B, UQCRQ, TPM4, TPM3, UQCR10, UQCR11, COX6B1, ACTC1, SLC8A1, COX7A2, COX8B, COX7A1, COX4I2, COX4I1, MYH7, ATP1A1, COX6C, CACNA2D4, ATP2A2, UQCRH, UQCRB, SLC9A1	1276	78	5738	2.13	3.99E-04	5.70E-05	0.002622723
KEGG_PATHWAY	mmu04512:ECM-receptor interaction	38	1.00	4.27E-06	IBSP, TNC, NPNT, COL3A1, COL2A1, SDC4, ITGB1, SDC2, CHAD, LAMB2, CD44, COMP, ITGB7, ITGB6, COL6A2, COL6A1, AGRN, COL11A2, THBS1, COL11A1, THBS2, THBS3, FN1, SPP1, THBS4, COL4A2, COL4A1, TNXB, COL5A2, VWF, LAMA4, SDC1, CD36, ITGA5, COL1A2, LAMC2, LAMC1, COL1A1	1276	83	5738	2.06	8.06E-04	1.01E-04	0.005297508
KEGG_PATHWAY	mmu00280:Valine, leucine and isoleucine degradation	25	0.66	7.94E-06	BCAT2, EHHADH, ECHS1, ACAT1, HADHA, HIBADH, AUH, HADHB, MCCC2, MUT, MCEE, OXCT1, MCCC1, ACAD8, HADH, HMGCL, BCKDHA, ACAA2, ACADM, ACADS, DBT, DLD, AOX1, HIBCH, PCCA	1276	46	5738	2.44	0.001499078	1.67E-04	0.009856236
KEGG_PATHWAY	mmu04010:MAPK signaling pathway	90	2.36	9.02E-06	FGF7, PDGFB, PDGFA, TGFB3, NFKB2, TGFB1, PRKX, TGFB2, MAP3K6, FOS, CASP3, MAP3K5, PAK2, B230120H23RIK, MAP3K8, FAS, RAPGEF2, FGF2, MYC, CHUK, MAP2K6, PRKCA, EGFR, BRAF, RELA, RELB, PTPRR, ECSIT, FLNC, FLNB, FLNA, STK3, MAP4K3, MAP4K4, RASGRF2, ARRB2, RRAS2, JUN, GADD45G, PDGFRA, HSPB1, MAPK7, MAP3K14, GADD45B, GADD45A, IL1R2, IL1R1, FGFR3, MKNK2, DUSP10, MAP4K2, CACNB1, HSPA1A, GNG12, HSPA1B, TNFRSF1A, KRAS, HSPA2, RASGRP3, RAC2, ELK4, MAP3K2, PLA2G12A, JUND, RASGRP2, HSPA8, RASA2, PTPN7, TRP53, TGFBR1, TGFBR2, NR4A1, MAPK11, TAB1, CACNA2D4, RPS6KA5, DUSP5, NRAS, DUSP4, ATF4, PLA2G4A, RPS6KA3, DUSP2, RPS6KA4, DUSP1, RPS6KA2, NTRK2, RAP1B, PLA2G4B, CD14, DUSP7	1276	265	5738	1.53	0.001703641	1.70E-04	0.011202286
KEGG_PATHWAY	mmu04670:Leukocyte transendothelial migration	47	1.23	3.16E-05	ITGAL, GNAI3, MYL2, GNAI2, MMP9, BCAR1, CLDN5, ITGB2, ITGB1, MMP2, PXN, ITGAM, VCL, MYL9, ACTG1, ARHGAP5, EZR, RAC2, PTK2B, PIK3R5, MSN, PIK3R1, MLLT4, PIK3R2, ACTB, PRKCA, ICAM1, ACTN4, ROCK1, NCF2, NCF1, ROCK2, NCF4, ACTN1, ACTN2, MAPK11, CTNNA1, VAV1, VASP, THY1, CYBA, CYBB, RASSF5, PLCG2, GRLF1, RAP1B, JAM2	1276	119	5738	1.78	0.005957607	5.43E-04	0.039252385
KEGG_PATHWAY	mmu05200:Pathways in cancer	103	2.70	3.93E-05	PDGFB, PGF, PDGFA, MMP9, TGFB3, FOXO1, MMP2, TGFB1, TGFB2, SLC2A1, RALB, CSF3R, RARA, FAS, RARB, EGFR, RET, BCR, BRAF, RELA, RXRG, CTNNA1, VEGFB, JUP, HIF1A, JUN, VEGFA, PDGFRA, LAMC2, LAMC1, WNT5A, FGFR3, SFPI1, BCL2L1, ITGB1, KRAS, LAMB2, RAC2, FH1, RUNX1, AXIN2, CSF1R, FN1, AXIN1, TRP53, BMP4, TCF7, BMP2, EPAS1, TGFBR1, MET, CREBBP, TGFBR2, SMAD3, CBLB, LAMA4, CDKN1A, PLCG2, PPARD, FGF7, NFKB2, FOS, CCNE1, CASP3, CASP8, FGF2, MYC, CSF2RA, CHUK, PRKCA, AR, HSP90AA1, CYCS, CDK4, SMO, CCND1, MDM2, WNT11, WNT9A, BID, PML, KIT, TPM3, IGF1R, PIK3R5, PIK3R1, PIK3R2, CEBPA, IL6, COL4A2, COL4A1, MSH2, FZD1, BIRC3, FZD5, STAT3, RALGDS, NRAS, HSP90B1, RASSF5, BAX, RASSF1, JAK1	1276	323	5738	1.43	0.007403353	6.19E-04	0.04881099

Table S2.C KEGG pathways significant at 24 hours

Category	Term	Count	%	PValue	Genes	List Total	Pop Hits	Pop Total	Fold Enrichment	Bonferroni	Benjamini	FDR
KEGG_PATHWAY	mmu00190:Oxidative phosphorylation	76	1.88	2.71E-17	ATP5D, UQCRC2, ATP5E, COX11, COX10, UQCRFS1, COX5A, COX5B, NDUFS7, NDUFS6, NDUFS5, UQCR10, UQCR11, NDUFS4, NDUFS8, ATP5L, ATP5O, NDUFS3, ATP5H, COX17, NDUFS1, ATP5K, ATP5J, NDUFB10, NDUFC2, ATP6V1H, NDUFC1, NDUFA10, ATP6V1D, COX6C, NDUFA11, ATP6V1A, UQCRH, ATP5C1, UQCRB, NDUFB3, NDUFB4, NDUFB5, NDUFB6, NDUFB7, NDUFB8, NDUFB9, COX7B, COX7C, ATP5G2, ATP6V1B2, ATP5G1, ATP6V1G1, COX7A2L, NDUFB2, ATP6V0E, COX6B1, TCIRG1, NDUFA4, NDUFA5, NDUFA2, ATP5J2, COX7A2, NDUFA3, NDUFA8, COX7A1, COX8B, NDUFA9, NDUFA6, NDUFA7, ATP5F1, LHPP, NDUFA1, PPA2, NDUFV3, ATP6V0E2, ATP6V1E1, SDHC, NDUFV2, SDHD, COX6A2	1355	130	5738	2.48	5.15E-15	5.15E-15	3.37E-14
KEGG_PATHWAY	mmu05010:Alzheimer's disease	87	2.15	1.15E-12	ATP5D, UQCRC2, ATP5E, IDE, COX5A, UQCRFS1, COX5B, NDUFS7, NDUFS6, NDUFS5, APP, CASP3, UQCR10, UQCR11, NDUFS4, CASP9, APOE, CASP8, NDUFS8, IL1B, ATP5O, NDUFS3, ATP5H, PLCB2, NDUFS1, ATP5J, NDUFB10, ADAM10, CYCS, NDUFC2, FADD, NDUFC1, NDUFA10, COX6C, UQCRH, ATP5C1, ERN1, GM12070, EIF2AK3, UQCRB, BID, NDUFB3, NDUFB4, HSD17B10, NDUFB5, NDUFB6, NDUFB7, NDUFB8, NDUFB9, COX7B, COX7C, MME, ATP5G2, ATP5G1, COX7A2L, NDUFB2, TNFRSF1A, COX6B1, PPP3CB, PPP3CA, GAPDH, NDUFA4, NDUFA5, NDUFA2, COX7A2, NDUFA3, NOS1, NDUFA8, COX7A1, COX8B, NDUFA9, NDUFA6, NDUFA7, ATP5F1, CAPN2, NDUFA1, ITPR1, CAPN1, ATF6, NDUFV3, LRP1, ATP2A2, SDHC, NDUFV2, SDHD, COX6A2, CALM3, APAF1, CALM2	1355	182	5738	2.02	2.19E-10	1.09E-10	1.43E-09
KEGG_PATHWAY	mmu05012:Parkinson's disease	69	1.70	3.01E-12	ATP5D, UQCRC2, ATP5E, UBE2G1, UQCRFS1, COX5A, COX5B, NDUFS7, NDUFS6, NDUFS5, UQCR10, CASP3, UQCR11, NDUFS4, CASP9, NDUFS8, ATP5O, NDUFS3, ATP5H, NDUFS1, ATP5J, NDUFB10, SLC25A5, CYCS, NDUFC2, NDUFC1, NDUFA10, COX6C, UQCRH, ATP5C1, UQCRB, NDUFB3, NDUFB4, NDUFB5, NDUFB6, NDUFB7, NDUFB8, NDUFB9, COX7B, COX7C, ATP5G2, COX7A2L, ATP5G1, NDUFB2, COX6B1, NDUFA4, NDUFA5, NDUFA2, COX7A2, NDUFA3, NDUFA8, COX7A1, COX8B, NDUFA9, NDUFA6, NDUFA7, ATP5F1, UBE2L6, VDAC2, VDAC3, NDUFA1, PARK7, VDAC1, NDUFV3, SDHC, NDUFV2, SDHD, COX6A2, APAF1	1355	133	5738	2.20	5.72E-10	1.91E-10	3.74E-09
KEGG_PATHWAY	mmu05016:Huntington's disease	86	2.12	5.05E-12	ATP5D, UQCRC2, ATP5E, CLTA, PPARG, COX5A, UQCRFS1, COX5B, NDUFS7, GPX1, NDUFS6, AP2B1, NDUFS5, CASP3, UQCR10, UQCR11, NDUFS4, CASP9, CASP8, NDUFS8, ATP5O, NDUFS3, ATP5H, PLCB2, NDUFS1, ATP5J, NDUFB10, SLC25A5, TAF4B, CYCS, NDUFC2, NDUFC1, NDUFA10, COX6C, UQCRH, ATP5C1, UQCRB, POLR2H, NDUFB3, NDUFB4, NDUFB5, NDUFB6, NDUFB7, NDUFB8, POLR2K, NDUFB9, POLR2I, COX7B, COX7C, ATP5G2, ATP5G1, COX7A2L, NDUFB2, POLR2A, TFAM, COX6B1, TGM2, TRP53, NDUFA4, NDUFA5, NDUFA2, COX7A2, NDUFA3, NDUFA8, COX8B, COX7A1, HTT, NDUFA9, NDUFA6, NDUFA7, ATP5F1, VDAC2, VDAC3, NDUFA1, ITPR1, VDAC1, SOD2, NDUFV3, HDAC1, BBC3, SDHC, BAX, NDUFV2, SDHD, COX6A2, APAF1	1355	183	5738	1.99	9.59E-10	2.40E-10	6.27E-09
KEGG_PATHWAY	mmu04210:Apoptosis	45	1.11	3.39E-08	BID, IL1R1, CSF2RB2, NFKBIA, BCL2L1, PRKX, AKT1, IRAK4, PRKAR2B, IRAK3, TNFRSF1A, CASP6, CASP3, MYD88, CASP9, BCL2, IL1RAP, CASP8, PPP3CB, CSF2RB, IL1B, PRKACA, PIK3R5, PPP3CA, CHUK, AKT2, IRAK2, TRP53, PIK3CG, RELA, CYCS, ENDOD1, FADD, CAPN2, BIRC3, TRADD, CAPN1, TNFRSF10B, ENDOG, RIPK1, BAX, PRKAR1A, APAF1, MAP3K14, IL3RA	1355	87	5738	2.19	6.44E-06	1.29E-06	4.21E-05
KEGG_PATHWAY	mmu04920:Adipocytokine signaling pathway	35	0.86	1.12E-06	PRKAG3, NFKBIE, STK11, NFKBIA, CAMKK1, CAMKK2, AKT1, TNFRSF1A, TNFRSF1B, SLC2A4, SLC2A1, PRKAA1, PRKAA2, ACSL3, CHUK, ACSL6, ACSL5, AKT2, CPT1B, SOCS3, RXRA, RELA, PRKAB2, PRKAB1, RXRG, ADIPOR1, ACACB, ADIPOQ, IRS1, CPT1A, STAT3, TRADD, PRKCQ, NPY, JAK2	1355	67	5738	2.21	2.13E-04	3.55E-05	0.001393144
KEGG_PATHWAY	mmu04666:Fc gamma R-mediated phagocytosis	44	1.09	6.54E-06	MARCKSL1, WASF2, ASAP1, ARPC4, ARF6, ARPC5, AKT1, DOCK2, RAC2, ARPC3, GAB2, ARPC2, GSN, PIKFYVE, PIK3R5, INPP5D, PAK1, AKT2, PRKCA, PIK3CG, PTPRC, VAV3, LYN, PPAP2C, LIMK1, NCF1, HCK, SPHK1, PRKCE, FCGR1, WAS, VAV1, PRKCD, VASP, PRKCB, ARPC1B, PLA2G4A, FCGR2B, ARPC5L, CFL1, PLCG2, SCIN, MARCKS, PLA2G4E	1355	98	5738	1.90	0.001241374	1.77E-04	0.00812502
KEGG_PATHWAY	mmu04062:Chemokine signaling pathway	70	1.73	7.95E-06	ADCY2, ADCY7, PREX1, STAT5B, CXCR2, PRKX, AKT1, TIAM2, CXCR4, TIAM1, RHOA, PRKACA, SHC1, PAK1, CSK, PLCB2, CHUK, AKT2, PIK3CG, LYN, NCF1, RELA, WAS, PRKCD, HRAS1, PRKCB, ELMO1, GNB2, PPBP, CCR5, ARRB2, GNB1, CCR2, CX3CR1, GNB5, CXCL1, PARD3, CCL3, GNAI3, CCL2, GNAI2, FGR, CCR1, CCL9, NFKBIA, CCL8, PF4, CCL7, CCL6, CCL24, CCL25, DOCK2, KRAS, RAC2, PTK2B, SOS2, PIK3R5, VAV3, HCK, VAV1, STAT3, STAT2, NRAS, CCL12, CXCL14, GNG10, CXCL16, JAK2, RAP1B, GRK5	1355	182	5738	1.63	0.001508532	1.89E-04	0.009874856
KEGG_PATHWAY	mmu04115:p53 signaling pathway	34	0.84	8.49E-06	BID, STEAP3, CHEK1, SFN, RRM2B, PMAIP1, CCNG1, SESN2, CCNG2, CCNE1, CASP3, CASP9, SHISA5, SERPINE1, CASP8, THBS1, TRP53, CDK1, CYCS, CDK6, CDK4, CDK2, RFWD2, CCND1, CDKN1A, EI24, BBC3, CCND2, CD82, RRM2, BAX, GADD45G, APAF1, GADD45A	1355	69	5738	2.09	0.001611612	1.79E-04	0.010550125
KEGG_PATHWAY	mmu00230:Purine metabolism	62	1.53	1.10E-05	GDA, ADCY2, GMPR2, ADCY7, PDE3B, PNP, PRIM1, NT5M, NT5C3, PDE4A, PRIM2, NT5C2, IMPDH1, NT5E, NT5C, NUDT2, POLR1E, NUDT5, PDE4D, PDE10A, POLR1B, GMPR, NME1, ADK, RRM2, ADSL, PRPS2, XDH, POLR2H, ADSS, ENPP1, POLR2K, NT5C1A, POLR2I, POLA1, DCK, RRM2B, PFAS, ADA, POLR2A, POLE2, PDE1B, ATIC, NUDT9, ENTPD4, ENTPD1, PAPSS1, PAPSS2, ADSSL1, AK1, POLR3GL, NPR2, AMPD2, AMPD3, APRT, POLR3D, AMPD1, POLD3, PDE7B, PDE2A, POLD1, PAICS	1355	157	5738	1.67	0.00208012	2.08E-04	0.013620114
KEGG_PATHWAY	mmu05200:Pathways in cancer	109	2.69	2.31E-05	PTGS2, PGF, MMP9, STAT5B, FGF13, TGFB1, WNT4, SLC2A1, RALB, CSF3R, RARA, RARB, EGFR, PIK3CG, BCR, RXRA, RELA, RXRG, SKP2, FADD, CTNNA1, STK4, CTNNA3, HRAS1, VEGFB, CCDC6, HIF1A, VEGFA, PDGFRA, LAMC2, TRAF1, NFKBIA, SFPI1, BCL2L1, ITGB1, RBX1, LAMB2, KRAS, RAC2, FH1, RUNX1, CSF1R, FN1, TRP53, BMP2, EPAS1, MAP2K2, SMAD3, ITGA3, CDKN1A, CDKN1B, HDAC1, PLCG2, E2F1, PPARG, NFKB2, AKT1, ACVR1B, CCNE1, FOS, CASP3, CASP9, CASP8, RHOA, MYC, CHUK, CSF2RA, AKT2, PRKCA, AR, HSP90AA1, CYCS, CDK6, CDK4, CDK2, PRKCB, DAPK1, SMO, CCND1, WNT11, WNT9A, BID, CKS1B, PML, EGLN2, KIT, ZBTB16, TPM3, IGF1R, BCL2, SOS2, PIK3R5, CEBPA, IL6, COL4A2, COL4A1, VHL, CBL, BIRC3, FZD5, FZD4, STAT3, FZD7, NRAS, HSP90B1, RASSF5, BAX, RASSF1, ARAF	1355	323	5738	1.43	0.004375503	3.99E-04	0.028680546

Table S2.D KEGG pathways significant at 48 hours

Category	Term	Count	%	PValue	Genes	List Total	Pop Hits	Pop Total	Fold Enrichment	Bonferroni	Benjamini	FDR
KEGG_PATHWAY	mmu00280:Valine, leucine and isoleucine degradation	28	1.30	2.79E-12	BCAT1, ACADSB, BCAT2, ECHS1, ACAT1, HIBADH, HADHA, AUH, HADHB, MCCC2, MUT, IVD, MCCC1, MCEE, OXCT1, HADH, BCKDHA, ACAA2, ALDH6A1, ACADM, ACADS, BCKDHB, DBT, DLD, ALDH2, PCCB, ALDH9A1, PCCA	820	46	5738	4.26	4.99E-10	4.99E-10	3.43E-09
KEGG_PATHWAY	mmu05010:Alzheimer's disease	55	2.56	4.10E-08	UQCRC2, UQCRC1, CYC1, NDUFAB1, UQCRFS1, NDUFS7, CASP3, NDUFS4, UQCR11, CASP9, APOE, NDUFS8, CASP8, IL1B, NDUFS3, PLCB2, NDUFS2, NDUFS1, ADAM10, NDUFB10, CYCS, NDUFC1, NDUFA10, ATP5C1, UQCRB, NDUFB3, BID, CDK5R1, TM4SF19, NDUFB8, NDUFB9, NDUFB2, TNFRSF1A, PPP3CB, PPP3CA, LPL, NDUFA5, NOS1, COX7A1, COX8B, NDUFA8, NDUFA9, NDUFA6, CACNA1S, SDHA, NDUFV3, SDHB, LRP1, ATP2A2, NDUFV1, SDHC, NDUFV2, COX6A2, COX6A1, APAF1	820	182	5738	2.11	7.33E-06	3.67E-06	5.04E-05
KEGG_PATHWAY	mmu05012:Parkinson's disease	44	2.05	6.92E-08	UQCRC2, NDUFB3, UQCRC1, NDUFB8, NDUFB9, CYC1, UCHL1, NDUFAB1, PINK1, UQCRFS1, NDUFB2, NDUFS7, CASP3, UQCR11, NDUFS4, CASP9, NDUFS8, NDUFS3, NDUFS2, NDUFS1, NDUFA5, NDUFB10, NDUFA8, COX8B, COX7A1, NDUFA9, SLC25A5, NDUFA6, CYCS, NDUFC1, NDUFA10, VDAC3, VDAC1, NDUFV3, SDHA, SDHB, NDUFV1, SDHC, NDUFV2, COX6A2, ATP5C1, COX6A1, APAF1, UQCRB	820	133	5738	2.31	1.24E-05	4.13E-06	8.52E-05
KEGG_PATHWAY	mmu04670:Leukocyte transendothelial migration	39	1.81	5.77E-07	ITGAL, GNAI3, MYL2, GNAI2, MMP9, ITGB2, ITGAM, VCL, ACTG1, VCAM1, EZR, RAC2, PTK2B, CXCR4, RHOA, PIK3R5, MSN, RHOH, ACTB, PRKCA, PIK3CG, ICAM1, VAV3, NCF2, ACTN4, NCF1, NCF4, ACTN1, MYL12B, VAV1, VASP, THY1, PRKCB, CYBA, CYBB, RASSF5, MAPK12, PLCG2, RAP1B	820	119	5738	2.29	1.03E-04	2.58E-05	7.10E-04
KEGG_PATHWAY	mmu00190:Oxidative phosphorylation	41	1.91	8.65E-07	UQCRC2, NDUFB3, UQCRC1, NDUFB8, NDUFB9, CYC1, NDUFAB1, UQCRFS1, NDUFB2, NDUFS7, ATP6V0E, UQCR11, NDUFS4, NDUFS8, NDUFS3, NDUFS2, NDUFS1, ATP5K, TCIRG1, NDUFA5, NDUFB10, NDUFA8, COX8B, COX7A1, NDUFA9, NDUFA6, ATP6V1H, NDUFC1, NDUFA10, NDUFV3, SDHA, SDHB, ATP6V1A, ATP6V0E2, NDUFV1, SDHC, NDUFV2, COX6A2, ATP5C1, COX6A1, UQCRB	820	130	5738	2.21	1.55E-04	3.10E-05	0.001065029
KEGG_PATHWAY	mmu00020:Citrate cycle (TCA cycle)	17	0.79	9.45E-07	SUCLG1, CS, IDH3B, ACLY, DLAT, OGDH, IDH3A, SDHA, SDHB, IDH3G, SDHC, DLD, IDH2, PDHA1, FH1, SUCLA2, MDH1	820	31	5738	3.84	1.69E-04	2.82E-05	0.001162609
KEGG_PATHWAY	mmu04666:Fc gamma R-mediated phagocytosis	33	1.53	2.63E-06	MARCKSL1, ARPC4, ARF6, ARPC5, DOCK2, RAC2, ARPC3, GAB2, ARPC2, PIK3R5, INPP5D, PIK3CG, PRKCA, PTPRC, VAV3, LYN, PPAP2C, NCF1, LIMK1, HCK, SPHK1, VAV1, PRKCD, WAS, FCGR1, VASP, PRKCB, ARPC1B, PLA2G4A, FCGR2B, CFL1, PLCG2, MARCKS	820	98	5738	2.36	4.71E-04	6.72E-05	0.0032361
KEGG_PATHWAY	mmu04062:Chemokine signaling pathway	49	2.28	9.62E-06	ADCY7, STAT5B, CXCR2, PRKX, CXCR4, RHOA, CSK, PLCB2, PIK3CG, LYN, NCF1, PRKCD, WAS, ELMO1, PRKCB, CCR5, PPBP, ARRB2, GNB1, CX3CR1, CCR2, GNAI3, CCL2, GNAI2, FGR, CCR1, CCL9, CCL8, PF4, CCL4, CCL7, CCL6, DOCK2, RAC2, PTK2B, SOS2, PIK3R5, VAV3, HCK, VAV1, STAT3, NRAS, ADCY9, CXCL14, CXCL13, CXCL16, GNG10, JAK2, RAP1B	820	182	5738	1.88	0.001720856	2.15E-04	0.011840157
KEGG_PATHWAY	mmu05016:Huntington's disease	49	2.28	1.13E-05	UQCRC2, CLTA, UQCRC1, CYC1, NDUFAB1, UQCRFS1, NDUFS7, GPX1, CASP3, NDUFS4, UQCR11, CASP9, NDUFS8, CASP8, NDUFS3, PLCB2, NDUFS2, NDUFS1, NDUFB10, SLC25A5, CYCS, NDUFC1, NDUFA10, ATP5C1, UQCRB, NDUFB3, NDUFB8, NDUFB9, NDUFB2, TRP53, NDUFA5, COX7A1, COX8B, NDUFA8, NDUFA9, NDUFA6, VDAC3, SOD2, VDAC1, SDHA, NDUFV3, SDHB, BAX, SDHC, NDUFV1, NDUFV2, COX6A2, COX6A1, APAF1	820	183	5738	1.87	0.002020134	2.25E-04	0.013901248
KEGG_PATHWAY	mmu00640:Propanoate metabolism	15	0.70	2.05E-05	ALDH6A1, LDHB, ACADM, SUCLG1, ECHS1, ACAT1, HADHA, MUT, ACSS1, MCEE, ALDH2, SUCLA2, PCCB, ALDH9A1, PCCA	820	30	5738	3.50	0.003656954	3.66E-04	0.025184004

Table S2.E KEGG pathways significant at 72 hours

Category	Term	Count	%	PValue	Genes	List Total	Pop Hits	Pop Total	Fold Enrichment	Bonferroni	Benjamini	FDR
KEGG_PATHWAY	mmu05010:Alzheimer's disease	125	1.73	1.05E-16	IDE, SNCA, NDUFAB1, COX5A, COX5B, APP, UQCR10, UQCR11, APOE, IL1B, PSENEN, FADD, COX6C, BACE2, MAPK3, ATP5C1, GM12070, EIF2AK3, HSD17B10, CDK5R1, COX7B, COX7C, MME, COX6B1, COX6B2, LPL, COX8B, COX8A, CACNA1S, NDUFV3, ATP2A2, ATP2A3, NDUFV1, NDUFV2, COX6A2, COX6A1, CACNA1D, ATP5D, UQCRC2, ATP5E, UQCRC1, ATP5B, CYC1, UQCRFS1, UQCRQ, NDUFS7, NDUFS6, NDUFS5, CASP3, PLCB3, NDUFS4, CASP9, CASP7, MAPT, CASP8, NDUFS8, ATP5O, NDUFS3, PLCB1, NDUFS2, ATP5H, PLCB2, NDUFS1, ATP5J, NDUFB10, ADAM10, CYCS, NDUFC2, COX4I2, COX4I1, NDUFC1, NDUFA10, GNAQ, UQCRH, PSEN2, ADAM17, UQCRB, BID, NDUFB3, NDUFB4, NDUFB5, NDUFB6, TNF, NDUFB7, NDUFB8, NDUFB9, APH1C, PPP3R1, ATP5G2, ATP5G1, COX7A2L, ATP5G3, NDUFB2, TNFRSF1A, PPP3CB, PPP3CC, PPP3CA, GAPDH, NDUFA4, NDUFA5, NDUFA2, NOS1, COX7A2, NDUFA3, COX7A1, NDUFA8, NDUFA9, NDUFA6, NDUFA7, ATP5F1, CAPN2, ITPR3, NDUFA1, ITPR1, CAPN1, ITPR2, NAE1, SDHA, SDHB, LRP1, GSK3B, SDHC, SDHD, CALM3, ATP5A1, APAF1	2203	182	5738	1.79	2.14E-14	2.14E-14	1.33E-13
KEGG_PATHWAY	mmu00190:Oxidative phosphorylation	95	1.32	2.12E-15	ATP5D, UQCRC2, ATP5E, COX11, UQCRC1, COX10, ATP5B, CYC1, NDUFAB1, COX5A, UQCRFS1, COX5B, UQCRQ, NDUFS7, NDUFS6, NDUFS5, UQCR10, UQCR11, NDUFS4, NDUFS8, ATP5L, ATP5O, ATP6V0D1, NDUFS3, NDUFS2, ATP6V0D2, ATP5H, COX17, NDUFS1, ATP5K, ATP5J, NDUFB10, NDUFC2, COX4I2, COX4I1, ATP6V1H, NDUFC1, NDUFA10, ATP6V1D, COX6C, NDUFA11, ATP6V1A, UQCRH, ATP5C1, UQCRB, NDUFB3, NDUFB4, NDUFB5, NDUFB6, NDUFB7, NDUFB8, NDUFB9, COX7B, COX7C, ATP5G2, ATP6V1B2, ATP5G1, ATP6V0C-PS2, ATP6V1G1, COX7A2L, ATP5G3, NDUFB2, ATP6V0E, COX6B1, COX6B2, TCIRG1, NDUFA4, NDUFA5, NDUFA2, ATP5J2, COX7A2, NDUFA3, NDUFA8, COX7A1, COX8B, NDUFA9, NDUFA6, COX8A, NDUFA7, ATP5F1, LHPP, NDUFA1, PPA2, SDHA, NDUFV3, SDHB, ATP6V0E2, NDUFV1, ATP6V1E1, SDHC, NDUFV2, SDHD, COX6A2, COX6A1, ATP5A1	2203	130	5738	1.90	4.07E-13	2.04E-13	2.63E-12
KEGG_PATHWAY	mmu05016:Huntington's disease	123	1.71	3.38E-15	NDUFAB1, COX5A, COX5B, BDNF, UQCR10, UQCR11, CREB3L1, CREB3L4, CREB3L3, RCOR1, PPARGC1A, COX6C, ATP5C1, COX7B, COX7C, COX6B1, COX6B2, HIP1, TRP53, COX8B, HTT, COX8A, VDAC2, VDAC3, VDAC1, NDUFV3, HDAC2, HDAC1, BBC3, NDUFV1, PPID, NDUFV2, COX6A2, COX6A1, ATP5D, UQCRC2, ATP5E, CLTA, UQCRC1, CLTB, AP2S1, ATP5B, CYC1, PPARG, CLTC, UQCRFS1, UQCRQ, NDUFS7, NDUFS6, GPX1, AP2B1, NDUFS5, CASP3, PLCB3, NDUFS4, CASP9, CASP8, NDUFS8, DLG4, ATP5O, NDUFS3, PLCB1, NDUFS2, ATP5H, PLCB2, NDUFS1, ATP5J, NDUFB10, SLC25A4, SLC25A5, CYCS, NDUFC2, COX4I2, COX4I1, NDUFC1, NDUFA10, GNAQ, UQCRH, UQCRB, NDUFB3, NDUFB4, NDUFB5, NDUFB6, NDUFB7, POLR2L, NDUFB8, POLR2K, NDUFB9, POLR2J, POLR2I, ATP5G2, ATP5G1, COX7A2L, ATP5G3, POLR2C, NDUFB2, POLR2A, TFAM, NDUFA4, NDUFA5, NDUFA2, COX7A2, NDUFA3, NDUFA8, COX7A1, NDUFA9, NDUFA6, NDUFA7, ATP5F1, CREB5, NDUFA1, ITPR1, SOD2, SDHA, SDHB, AP2A2, SP1, IFT57, BAX, SDHC, SDHD, APAF1, ATP5A1	2203	183	5738	1.75	6.43E-13	2.14E-13	4.15E-12
KEGG_PATHWAY	mmu05012:Parkinson's disease	94	1.30	8.90E-14	ATP5D, UQCRC2, ATP5E, UQCRC1, ATP5B, UBE2G1, SNCA, UCHL1, CYC1, NDUFAB1, PINK1, COX5A, UQCRFS1, COX5B, UQCRQ, NDUFS7, NDUFS6, NDUFS5, CASP3, UQCR10, UQCR11, NDUFS4, HTRA2, CASP9, NDUFS8, ATP5O, NDUFS3, NDUFS2, ATP5H, NDUFS1, ATP5J, NDUFB10, SLC25A4, SLC25A5, CYCS, NDUFC2, COX4I2, COX4I1, NDUFC1, NDUFA10, COX6C, UQCRH, UBC, ATP5C1, UBA52, UQCRB, NDUFB3, NDUFB4, NDUFB5, NDUFB6, NDUFB7, NDUFB8, NDUFB9, COX7B, UBA7, COX7C, ATP5G2, ATP5G1, COX7A2L, ATP5G3, NDUFB2, COX6B1, COX6B2, NDUFA4, NDUFA5, NDUFA2, COX7A2, NDUFA3, NDUFA8, COX7A1, COX8B, NDUFA9, NDUFA6, COX8A, NDUFA7, ATP5F1, UBE2L6, VDAC2, VDAC3, NDUFA1, PARK7, VDAC1, SDHA, NDUFV3, SDHB, NDUFV1, SDHC, PPID, NDUFV2, SDHD, COX6A2, SLC18A2, COX6A1, APAF1, ATP5A1	2203	133	5738	1.84	1.72E-11	4.29E-12	1.11E-10
KEGG_PATHWAY	mmu00020:Citrate cycle (TCA cycle)	28	0.39	2.69E-08	OGDH, PDHB, IDH3G, IDH2, IDH1, FH1, CSL, PDHA1, SUCLA2, DLST, ACO2, SUCLG2, ACO1, SUCLG1, CS, IDH3B, ACLY, DLAT, PCK2, IDH3A, SDHA, SDHB, PCX, SDHC, DLD, SDHD, MDH2, MDH1	2203	31	5738	2.35	5.20E-06	1.04E-06	3.36E-05
KEGG_PATHWAY	mmu04620:Toll-like receptor signaling pathway	66	0.92	2.78E-08	TBK1, TLR1, TIRAP, TLR2, TLR3, NFKB1, TLR4, TLR5, TLR6, TLR7, TLR8, TLR9, AKT1, MAP3K7, FOS, MYD88, TICAM1, CASP8, MAP3K8, TICAM2, IL1B, PIK3CA, LBP, MAP2K7, AKT3, MAP2K6, AKT2, PIK3CG, LY96, RELA, PIK3CD, FADD, CD40, IFNAR1, IFNAR2, CD86, CD80, JUN, RIPK1, MAPK3, IL12A, MAPK9, MAPK8, CCL3, TNF, CXCL9, NFKBIA, CCL5, CCL4, IRAK4, PIK3R5, SPP1, IL6, MAP2K2, MAP2K3, MAP2K4, MAPK11, STAT1, TAB2, IKBKE, IRF5, MAPK12, IRF7, MAPK14, IKBKB, CD14	2203	99	5738	1.74	5.37E-06	8.96E-07	3.47E-05
KEGG_PATHWAY	mmu04142:Lysosome	76	1.05	3.39E-08	SGSH, ARSB, CLTA, CLTB, GM2A, AP1B1, HEXA, LGMN, HEXB, AP3S1, CLTC, ASAH1, GLB1, AP1S3, AP1S1, AP1S2, ATP6V0D1, MAN2B1, ATP6V0D2, AP3B1, CLN3, HYAL1, CTSZ, ATP6V1H, NAPSA, CTSS, M6PR, DNASE2A, CTSW, GNS, CTSL, CTSK, CTSO, GAA, CTSD, NEU1, CTSC, CTSB, CTSH, CTSF, AP1M1, NAGLU, MFSD8, ACP5, PPT1, CTSA, ACP2, ATP6V0C-PS2, SLC11A1, CD68, NAGPA, LAPTM5, AP3M2, GALNS, NAGA, GALC, AP3D1, ENTPD4, GBA, LAPTM4B, TCIRG1, ABCB9, PLA2G15, LIPA, PSAP, GUSB, CD63, FUCA1, MANBA, SLC17A5, LAMP2, GLA, ARSA, SMPD1, CTNS, GGA2	2203	119	5738	1.66	6.53E-06	9.33E-07	4.22E-05
KEGG_PATHWAY	mmu04621:NOD-like receptor signaling pathway	46	0.64	4.13E-08	CXCL1, TNF, CCL2, IL18, NFKBIB, CCL8, NFKBIA, NFKB1, NLRP1A, NLRP1B, CCL5, CCL7, NAIP6, MAP3K7, NLRC4, NOD2, NOD1, MEFV, NAIP2, CASP8, NAIP5, PSTPIP1, PYCARD, IL1B, CASP1, IL6, HSP90AA1, CARD9, RELA, MAPK11, CARD6, SUGT1, BIRC3, BIRC2, TAB2, CCL12, HSP90B1, MAPK12, MAPK14, MAPK3, RIPK2, MAPK9, MAPK8, TNFAIP3, TRIP6, IKBKB	2203	62	5738	1.93	7.98E-06	9.97E-07	5.15E-05
KEGG_PATHWAY	mmu04062:Chemokine signaling pathway	106	1.47	6.48E-08	ADCY3, ADCY1, ADCY2, ADCY7, STAT5B, CXCL12, PRKX, PIK3CA, GNG2, GNG5, GNG7, PIK3CG, ROCK1, ROCK2, NCF1, RELA, PIK3CD, HRAS1, CCR7, PPBP, CCR5, ARRB2, CCR2, CX3CR1, MAPK3, CCL3, GNAI3, CCL2, GNAI2, FGR, GRB2, NFKBIB, CCL9, CCL8, NFKBIA, CX3CL1, CCL5, CCL4, CCL7, CCL6, DOCK2, RAC2, VAV3, VAV2, VAV1, CXCL14, CXCL13, CXCL16, GRK6, GRK4, RAP1B, GRK5, IKBKB, PREX1, NFKB1, CXCR2, CXCR3, AKT1, PLCB3, CXCR4, RHOA, SHC1, PLCB1, CSK, PLCB2, AKT3, AKT2, LYN, WAS, PRKCD, PRKCB, ELMO1, GNB2, GNB1, GNB5, GNB4, GNB3, CXCL1, PARD3, CXCL5, CCR1, CXCL9, PF4, GNG11, GNG12, CCL28, CCL24, CCL25, CCL22, PTK2B, SOS1, SOS2, PIK3R5, HCK, RAF1, STAT1, STAT3, STAT2, NRAS, CCL12, ADCY9, GSK3A, GSK3B, GNG10, JAK2, JAK3	2203	182	5738	1.52	1.25E-05	1.39E-06	8.08E-05
KEGG_PATHWAY	mmu04010:MAPK signaling pathway	144	2.00	1.00E-07	MEF2C, FGF18, PDGFA, GNA12, FGF13, TGFB1, PRKX, TGFB2, MAP3K7, MAP3K6, BDNF, MAP3K4, B230120H23RIK, MAP3K8, IL1B, RRAS, MAP2K7, MAP2K6, EGFR, RELA, RELB, PTPRR, MECOM, STK4, STK3, HRAS1, MAP4K4, ARRB2, JUN, MAPK3, PDGFRA, HSPB1, MAPK9, MAPK8, MAPK7, STMN1, PLA2G2E, NGF, PPP5C, IL1R2, FGFR4, IL1R1, FGFR3, GRB2, MAPKAPK5, CACNB1, DUSP10, MAP4K1, CACNB3, MAPKAPK2, HSPA1B, SRF, HSPA1L, RAC2, DUSP14, RAC3, JUND, DUSP16, EGF, PTPN7, TRP53, CACNA2D1, NTF5, NTF3, MAP2K2, MAP2K3, TGFBR1, TGFBR2, MAP2K4, TAOK3, NR4A1, CACNA1S, CACNA2D4, ATF4, PLA2G4A, RAP1B, IKBKB, CACNA1D, CACNA1A, PLA2G4E, FGF6, NFKB1, NFKB2, DAXX, AKT1, ACVR1B, FOS, MAX, CASP3, PAK2, MAPT, MYC, AKT3, AKT2, PRKCA, CACNG7, CACNG6, CACNG1, ECSIT, FLNB, FLNA, PRKCB, GADD45G, PLA2G6, MAP3K14, GADD45A, MAP3K11, TNF, PPM1A, PPP3R1, GNG12, PPM1B, TNFRSF1A, RASGRP3, RASGRP4, MAP3K2, PLA2G12A, SOS1, MAP3K1, RASGRP1, SOS2, PPP3CB, PPP3CC, NFATC4, PPP3CA, NFATC2, RAF1, MAPK11, TAB2, CDC25B, RPS6KA5, DUSP5, RPS6KA6, DUSP4, NRAS, RPS6KA4, DUSP2, MAPK12, RPS6KA1, RPS6KA2, MAPK14, DUSP9, DUSP8, CD14, DUSP6	2203	265	5738	1.42	1.93E-05	1.93E-06	1.25E-04
KEGG_PATHWAY	mmu04920:Adipocytokine signaling pathway	48	0.67	1.16E-07	PRKAG3, PPARA, TNF, NFKBIE, STK11, PRKAG1, NFKBIB, PRKAG2, NFKBIA, NFKB1, CAMKK1, CAMKK2, AKT1, TNFRSF1A, TNFRSF1B, ACSL1, SLC2A4, SLC2A1, PRKAA2, ACSL3, ACSL6, AKT3, ACSL5, AKT2, CPT1C, CPT1B, SOCS3, RELA, RXRA, PRKAB2, RXRG, ADIPOR2, PRKAB1, ADIPOR1, ACACB, PCK2, PPARGC1A, IRS1, CPT1A, STAT3, TRADD, PTPN11, PRKCQ, NPY, MAPK9, MAPK8, JAK2, IKBKB	2203	67	5738	1.87	2.23E-05	2.03E-06	1.44E-04
KEGG_PATHWAY	mmu04210:Apoptosis	58	0.80	2.24E-07	NFKB1, PRKX, AKT1, PRKAR2B, CASP6, CASP3, PRKAR2A, MYD88, CASP9, CASP7, IL1RAP, CASP8, EXOG, CSF2RB, IL1B, PIK3CA, AKT3, AKT2, PIK3CG, IRAK2, AIFM1, DFFA, RELA, PIK3CD, CYCS, FADD, TNFRSF10B, RIPK1, MAP3K14, NGF, BID, IL1R1, TNF, CSF2RB2, PPP3R1, NFKBIA, BCL2L1, IRAK4, IRAK3, TNFRSF1A, BCL2, PPP3CB, PPP3CC, PIK3R5, PPP3CA, TRP53, ENDOD1, CAPN2, BIRC3, BIRC2, TRADD, CAPN1, TNFSF10, ENDOG, BAX, APAF1, IKBKB, IL3RA	2203	87	5738	1.74	4.33E-05	3.61E-06	2.79E-04
KEGG_PATHWAY	mmu04260:Cardiac muscle contraction	53	0.73	3.33E-07	UQCRC2, ATP1B1, UQCRC1, SLC9A6, ATP1B3, ATP1B2, CYC1, UQCRFS1, COX5A, UQCRQ, COX5B, UQCR10, UQCR11, CACNG7, CACNG6, COX4I2, COX4I1, MYH7, CACNG1, COX6C, TNNT2, UQCRH, UQCRB, FXYD2, MYL2, MYL3, COX7B, COX7C, CACNB1, CACNB3, COX7A2L, TPM2, TPM4, TPM3, COX6B1, COX6B2, CACNA2D1, ACTC1, COX7A2, COX7A1, COX8B, COX8A, ATP1A3, ATP1A1, ATP1A2, TNNI3, CACNA1S, CACNA2D4, ATP2A2, COX6A2, COX6A1, CACNA1D, SLC9A1	2203	78	5738	1.77	6.42E-05	4.94E-06	4.14E-04
KEGG_PATHWAY	mmu00280:Valine, leucine and isoleucine degradation	35	0.49	9.64E-07	BCAT1, HSD17B10, ACADSB, BCAT2, EHHADH, ECHS1, ACAT2, ACAT1, HIBADH, HADHA, ALDH3A2, HADHB, AUH, MCCC2, MUT, IVD, MCCC1, MCEE, OXCT1, ACAD8, HADH, BCKDHA, ACAA2, ALDH6A1, ACADM, ACADS, BCKDHB, DBT, ALDH1B1, DLD, AOX1, HIBCH, PCCB, PCCA, ALDH9A1	2203	46	5738	1.98	1.86E-04	1.33E-05	0.001200903
KEGG_PATHWAY	mmu04662:B cell receptor signaling pathway	53	0.73	1.10E-06	NFKB1, BTK, AKT1, FOS, PIK3CA, PIK3AP1, AKT3, AKT2, PIK3CG, BCL10, LYN, RELA, PIK3CD, HRAS1, PRKCB, CARD11, LILRB3, JUN, MAPK3, IFITM1, GRB2, NFKBIE, NFKBIB, PPP3R1, NFKBIA, RAC2, DAPP1, RASGRP3, RAC3, SOS1, SOS2, PPP3CB, CD22, PPP3CC, PIK3R5, NFATC4, PPP3CA, INPP5D, NFATC2, BLNK, PTPN6, VAV3, MAP2K2, RAF1, MALT1, VAV2, VAV1, NRAS, FCGR2B, GSK3B, PLCG2, CD79B, IKBKB	2203	80	5738	1.73	2.13E-04	1.42E-05	0.001375792
KEGG_PATHWAY	mmu05200:Pathways in cancer	166	2.30	1.22E-06	FGF18, PTGS2, PGF, PDGFA, MMP9, STAT5B, FGF13, MMP2, TGFB1, TGFB2, CUL2, WNT4, CDKN2B, SLC2A1, RALB, RALA, CSF3R, PIK3CA, RARA, RARB, PIK3CG, EGFR, BCR, RELA, RXRA, PIK3CD, SKP2, RXRG, FADD, MECOM, CTNNA1, STK4, CTNNA3, HRAS1, VEGFB, CCDC6, HIF1A, PIAS3, JUN, MAPK3, VEGFA, PDGFRA, MAPK9, PIAS2, LAMC2, MAPK8, WNT5A, TRAF1, FGFR3, GRB2, NFKBIA, SFPI1, BCL2L1, RBX1, LAMB2, RAC2, RAC3, ITGAV, FH1, EGF, RUNX1, TRAF5, TRAF4, CSF1R, FN1, TRP53, DVL2, TCF7, EPAS1, MAP2K2, TGFBR1, TGFBR2, SMAD4, SMAD3, BRCA2, ITGA3, HGF, DVL1, CDKN1A, LAMA4, HDAC2, ITGA6, HDAC1, PLCG2, IKBKB, FGF6, E2F1, E2F2, PPARG, NFKB1, NFKB2, GLI3, AKT1, ACVR1B, CCNE1, FOS, MAX, CASP3, CASP9, CASP8, RHOA, MYC, AKT3, CSF2RA, AKT2, PRKCA, CTBP1, AR, HSP90AA1, CYCS, CDK6, CDK4, CDK2, DAPK1, RAD51, PRKCB, SMO, CCND1, MDM2, WNT11, WNT9A, BID, CKS1B, PML, EGLN2, ZBTB16, TCF7L2, TPM3, BCL2, SOS1, SOS2, PIK3R5, FLT3L, FIGF, CEBPA, FZD9, IL6, COL4A1, MSH3, MSH2, VHL, FLT3, CBL, FZD1, RAF1, IGF1, BIRC5, FZD2, APPL1, STAT1, FZD5, BIRC3, FZD4, BIRC2, RALGDS, FZD7, STAT3, FZD6, NRAS, RASSF5, HSP90B1, RASSF1, GSK3B, BAX, ARAF, TCEB1	2203	323	5738	1.34	2.35E-04	1.47E-05	0.001518548
KEGG_PATHWAY	mmu04510:Focal adhesion	107	1.48	7.61E-06	PGF, PDGFA, VTN, MYL10, ACTG1, PIK3CA, PDGFC, EGFR, PIK3CG, ROCK1, ACTN4, ROCK2, PIK3CD, ACTN1, MYLK2, ACTN3, PPP1CB, HRAS1, VEGFB, JUN, VEGFA, MAPK3, PDGFRA, COL1A2, MAPK9, LAMC2, MAPK8, COL1A1, CAV3, MYL2, GRB2, ITGA11, ITGB3, SRC, MYL9, DOCK1, LAMB2, RAC2, RAC3, ITGAV, ITGB7, ITGB6, EGF, FN1, SPP1, VAV3, ITGA3, MYL12B, ITGA4, HGF, VAV2, VAV1, KDR, ITGA9, LAMA4, ITGA6, ITGA5, ITGA7, RAP1B, TLN2, PIP5K1C, AKT1, ARHGAP5, PAK2, PAK4, RHOA, SHC1, ZYX, AKT3, AKT2, PRKCA, PARVG, FLNB, FLNA, VASP, PRKCB, CCND1, CCND3, CCND2, PARVA, TNC, COL3A1, SOS1, COMP, BCL2, SOS2, COL6A2, COL6A1, PIK3R5, TNN, THBS1, FIGF, THBS2, THBS3, ACTB, COL4A1, FLT1, RAF1, IGF1, BIRC3, CAPN2, COL5A3, COL5A2, BIRC2, COL5A1, FYN, GSK3B	2203	198	5738	1.41	0.001467105	8.64E-05	0.009478696

KEGG_PATHWAY	mmu05212:Pancreatic cancer	47	0.65	9.20E-06	E2F1, E2F2, PGF, NFKB1, BCL2L1, TGFB1, TGFB2, AKT1, ACVR1B, RAC2, CASP9, RAC3, RALB, RALA, PIK3CA, PIK3R5, EGF, FIGF, AKT3, AKT2, PIK3CG, TRP53, EGFR, PLD1, RELA, ARHGEF6, TGFBR1, PIK3CD, TGFBR2, SMAD4, RAF1, BRCA2, SMAD3, CDK6, STAT1, CDK4, STAT3, RALGDS, RAD51, VEGFB, CCND1, ARAF, VEGFA, MAPK3, MAPK9, MAPK8, IKBKB	2203	72	5738	1.70	0.001774079	9.86E-05	0.011463648
KEGG_PATHWAY	mmu05210:Colorectal cancer	53	0.73	2.43E-05	TGFB1, TGFB2, AKT1, FOS, ACVR1B, CASP3, CASP9, PIK3CA, MYC, AKT3, AKT2, EGFR, PIK3CG, PIK3CD, CYCS, CCND1, JUN, MAPK3, PDGFRA, MAPK9, MAPK8, GRB2, TCF7L2, RAC2, RAC3, SOS1, BCL2, SOS2, PIK3R5, DVL2, FZD9, TRP53, TCF7, MSH3, MSH2, TGFBR1, TGFBR2, SMAD4, FZD1, RAF1, SMAD3, BIRC5, FZD2, FZD5, APPL1, FZD4, FZD7, RALGDS, FZD6, DVL1, BAX, GSK3B, ARAF	2203	86	5738	1.61	0.004670485	2.46E-04	0.030220506
KEGG_PATHWAY	mmu05220:Chronic myeloid leukemia	48	0.67	2.60E-05	E2F1, E2F2, GRB2, STAT5B, NFKBIA, NFKB1, BCL2L1, TGFB1, TGFB2, AKT1, ACVR1B, GAB2, SOS1, SOS2, PIK3CA, SHC1, PIK3R5, RUNX1, MYC, AKT3, AKT2, PIK3CG, TRP53, CTBP1, BCR, MAP2K2, RELA, TGFBR1, CBL, PIK3CD, TGFBR2, SMAD4, RAF1, SMAD3, CDK6, MECOM, CDK4, PTPN11, HRAS1, NRAS, CCND1, CDKN1A, HDAC2, HDAC1, ARAF, MAPK3, MDM2, IKBKB	2203	76	5738	1.65	0.005013764	2.51E-04	0.032446929

Table S2.F KEGG pathways significant at 168 hours

Category	Term	Count	%	PValue	Genes	List Total	Pop Hits	Pop Total	Fold Enrichment	Bonferroni	Benjamini	FDR
KEGG_PATHWAY	mmu04062:Chemokine signaling pathway	18	0.65	4.47E-07	CCL2, FGR, NCF1, HCK, CCL9, CCL8, WAS, CCL7, CCL17, PRKCB, CCL12, CCR5, RAC2, CCR2, CX3CR1, PIK3R5, XCL1, XCR1	129	182	5738	4.40	4.78E-05	4.78E-05	5.02E-04
KEGG_PATHWAY	mmu04670:Leukocyte transendothelial migration	14	0.50	1.93E-06	NCF2, NCF1, NCF4, ITGB2, PRKCB, VCAM1, CYBA, CYBB, EZR, RAC2, PLCG2, CLDN2, PIK3R5, RHOH	129	119	5738	5.23	2.06E-04	1.03E-04	0.002168346
KEGG_PATHWAY	mmu04650:Natural killer cell mediated cytotoxicity	13	0.47	1.46E-05	BID, FCGR4, ITGB2, NCR1, PRKCB, FCGR3, CD48, RAC2, PLCG2, FCER1G, PIK3R5, LCP2, TYROBP	129	122	5738	4.74	0.001563801	5.22E-04	0.016439609
KEGG_PATHWAY	mmu05322:Systemic lupus erythematosus	12	0.43	1.55E-05	C1QA, C1QB, C7, CD86, C4B, FCGR4, H2-DMB1, HIST1H4I, C1QC, FCGR1, IL10, FCGR3	129	103	5738	5.18	0.001652768	4.13E-04	0.017375573
KEGG_PATHWAY	mmu04060:Cytokine-cytokine receptor interaction	18	0.65	2.48E-05	CCL2, CSF2RB2, CCL9, CCL8, IL10, CCL7, CCL17, CCL12, CCR5, IL10RA, CCR2, CX3CR1, CSF2RB, IL2RG, IL12B, XCL1, XCR1, CSF2RA	129	244	5738	3.28	0.00265068	5.31E-04	0.027879105

Table S2.G KEGG pathways significant at 336 hours

Category	Term	Count	%	PValue	Genes	List Total	Pop Hits	Pop Total	Fold Enrichment	Bonferroni	Benjamini	FDR
KEGG_PATHWAY	mmu04512:ECM-receptor interaction	18	2.36	7.85E-08	IBSP, TNC, COL3A1, ITGB5, ITGA4, COL5A3, COL5A2, SDC2, COL5A1, CD44, COL1A2, COL6A2, COL6A1, COL1A1, THBS2, THBS3, SPP1, FN1	254	83	5738	4.90	1.08E-05	1.08E-05	9.24E-05
KEGG_PATHWAY	mmu05416:Viral myocarditis	16	2.10	1.24E-05	H2-K1, ITGAL, MYH3, H2-D1, H2-DMB1, ITGB2, MYH7, H2-AB1, MYH8, CD86, H2-EB1, SGCD, H2-AA, MYH13, MYH14, ABL2	254	94	5738	3.85	0.001713391	8.57E-04	0.014623985
KEGG_PATHWAY	mmu04062:Chemokine signaling pathway	23	3.01	1.34E-05	PARD3, CCL2, ADCY7, NCF1, STAT5B, CXCL9, CCL9, CCL8, GNG11, PF4, CCL6, CCL12, CCR5, ADCY9, TIAM1, CXCR4, GNG10, CXCL16, GSK3B, CCR2, SOS2, PAK1, SHC2	254	182	5738	2.85	0.001841954	6.14E-04	0.015722211
KEGG_PATHWAY	mmu04510:Focal adhesion	24	3.15	1.64E-05	IBSP, FLT1, TNC, COL3A1, ITGB5, ITGA4, COL5A3, COL5A2, COL5A1, GSK3B, BCL2, SOS2, COL6A2, GRLF1, COL1A2, COL6A1, COL1A1, PAK1, EGF, SHC2, THBS2, THBS3, SPP1, FN1	254	198	5738	2.74	0.002258263	5.65E-04	0.019279342

Table S2.H KEGG pathways significant at 504 hours

Table S2.H KEGG pathways significant at 504 hours

Category	Term	Count	%	PValue	Genes	List Total	Pop Hits	Pop Total	Fold Enrichment	Bonferroni	Benjamini	FDR
KEGG_PATHWAY	mmu04512:ECM-receptor interaction	18	2.36	7.85E-08	IBSP, TNC, COL3A1, ITGB5, ITGA4, COL5A3, COL5A2, SDC2, COL5A1, CD44, COL1A2, COL6A2, COL6A1, COL1A1, THBS2, THBS3, SPP1, FN1	254	83	5738	4.90	1.08E-05	1.08E-05	9.24E-05
KEGG_PATHWAY	mmu05416:Viral myocarditis	16	2.10	1.24E-05	H2-K1, ITGAL, MYH3, H2-D1, H2-DMB1, ITGB2, MYH7, H2-AB1, MYH8, CD86, H2-EB1, SGCD, H2-AA, MYH13, MYH14, ABL2	254	94	5738	3.85	0.001713391	8.57E-04	0.014623985
KEGG_PATHWAY	mmu04062:Chemokine signaling pathway	23	3.01	1.34E-05	PARD3, CCL2, ADCY7, NCF1, STAT5B, CXCL9, CCL9, CCL8, GNG11, PF4, CCL6, CCL12, CCR5, ADCY9, TIAM1, CXCR4, GNG10, CXCL16, GSK3B, CCR2, SOS2, PAK1, SHC2	254	182	5738	2.85	0.001841954	6.14E-04	0.015722211
KEGG_PATHWAY	mmu04510:Focal adhesion	24	3.15	1.64E-05	IBSP, FLT1, TNC, COL3A1, ITGB5, ITGA4, COL5A3, COL5A2, COL5A1, GSK3B, BCL2, SOS2, COL6A2, GRLF1, COL1A2, COL6A1, COL1A1, PAK1, EGF, SHC2, THBS2, THBS3, SPP1, FN1	254	198	5738	2.74	0.002258263	5.65E-04	0.019279342

Table S2.I KEGG pathways significant at 672 hours

Category	Term	Count	%	PValue	Genes	List Total	Pop Hits	Pop Total	Fold Enrichment	Bonferroni	Benjamini	FDR
KEGG_PATHWAY	mmu04512:ECM-receptor interaction	17	2.51	7.58E-08	IBSP, TNC, COL3A1, ITGA4, COL5A2, SDC2, COL5A1, CD44, COMP, COL1A2, COL6A2, COL6A1, COL1A1, THBS2, COL11A1, THBS3, FN1	223	83	5738	5.27	1.08E-05	1.08E-05	8.96E-05
KEGG_PATHWAY	mmu04510:Focal adhesion	26	3.85	1.21E-07	IBSP, CAV2, CAV1, MYL2, TNC, COL3A1, BCL2, COMP, COL6A2, COL6A1, PAK1, EGF, COL11A1, THBS2, THBS3, FN1, VAV3, IGF1, ITGA4, COL5A2, COL5A1, PDGFRA, COL1A2, GRLF1, COL1A1, PARVA	223	198	5738	3.38	1.72E-05	8.62E-06	1.44E-04
KEGG_PATHWAY	mmu04610:Complement and coagulation cascades	15	2.22	7.70E-07	C3AR1, CFB, F13A1, SERPING1, C1S, C1QC, C1QA, C1QB, C1RA, CD55, F3, CFH, C2, CFD, PROS1	223	75	5738	5.15	1.09E-04	3.64E-05	9.10E-04

Table S3. Significant GO clusters identified from the 168h time point that were obtained from differentially expressed genes that were determined to contribute the largest variance at that time point.

Cluster 1: Enrichment Score: 11.54				
**Annotation**	**GO Term**	**Gene Count**	**P-value**	**FDR**
GOTERM_BP_FAT	vasculature development	85	5.20E-14	9.70E-11
GOTERM_BP_FAT	blood vessel development	83	1.00E-13	1.90E-10
GOTERM_BP_FAT	blood vessel morphogenesis	69	4.30E-12	8.00E-09
GOTERM_BP_FAT	angiogenesis	48	3.00E-09	5.60E-06
**Cluster 2: Enrichment Score: 11.31**				
**Annotation**	**GO Term**	**Gene Count**	**P-value**	**FDR**
GOTERM_CC_FAT	extracellular matrix	100	2.50E-14	3.60E-11
SP_PIR_KEYWORD	extracellular matrix	76	7.40E-14	1.10E-10
GOTERM_CC_FAT	proteinaceous extracellular m	96	9.40E-14	1.40E-10
GOTERM_CC_FAT	extracellular region part	179	1.10E-09	1.60E-06
GOTERM_CC_FAT	extracellular matrix part	37	1.50E-08	2.20E-05
**Cluster 3: Enrichment Score: 9.38**				
**Annotation**	**GO Term**	**Gene Count**	**P-value**	**FDR**
KEGG_PATHWAY	lysosome	53	2.40E-12	3.00E-09
SP_PIR_KEYWORD	lysosome	52	5.60E-10	8.30E-07
GOTERM_CC_FAT	lysosome	60	1.00E-09	1.50E-06
GOTERM_CC_FAT	lytic vacuole	60	1.30E-09	1.90E-06
GOTERM_CC_FAT	vacuole	64	6.80E-09	9.90E-06
**Cluster 4: Enrichment Score: 7.85**				
**Annotation**	**GO Term**	**Gene Count**	**P-value**	**FDR**
GOTERM_BP_FAT	vesicle-mediated transport	121	2.70E-10	5.00E-07
GOTERM_BP_FAT	membrane invagination	61	2.10E-09	3.90E-06
GOTERM_BP_FAT	endocytosis	61	2.10E-09	3.90E-06
GOTERM_BP_FAT	membrane organization	76	3.10E-08	5.70E-05
SP_PIR_KEYWORD	endocytosis	31	1.60E-05	2.40E-02
**Cluster 5: Enrichment Score: 7.18**				
**Annotation**	**GO Term**	**Gene Count**	**P-value**	**FDR**
GOTERM_BP_FAT	actin filament-based process	61	1.10E-10	2.00E-07
GOTERM_BP_FAT	actin cytoskeleton organizati	57	5.60E-10	1.00E-06
GOTERM_BP_FAT	actin filament organization	23	6.70E-06	1.20E-02
GOTERM_BP_FAT	cytoskeleton organization	76	4.80E-05	8.80E-02
